# Mice and Men: Their Promoter Properties

**DOI:** 10.1371/journal.pgen.0020054

**Published:** 2006-04-28

**Authors:** Vladimir B Bajic, Sin Lam Tan, Alan Christoffels, Christian Schönbach, Leonard Lipovich, Liang Yang, Oliver Hofmann, Adele Kruger, Winston Hide, Chikatoshi Kai, Jun Kawai, David A Hume, Piero Carninci, Yoshihide Hayashizaki

**Affiliations:** 1 Knowledge Extraction Laboratory, Institute for Infocomm Research, Singapore; 2 South African National Bioinformatics Institute, University of the Western Cape, Bellville, South Africa; 3 Temasek Life Sciences Laboratory, National University of Singapore, Singapore; 4 School of Biological Sciences, Nanyang Technological University, Singapore; 5 Immunoinformatics Research Team, Advanced Genome Information Technology Research Group, RIKEN Genomic Sciences Center, RIKEN Yokohama Institute, Yokohama, Japan; 6 Genome Institute of Singapore, Singapore; 7 Department of Obstetrics and Gynecology, National University Hospital, National University of Singapore, Singapore; 8 Genome Exploration Research Group (Genome Network Project Core Group), RIKEN Genomic Sciences Center, RIKEN Yokohama Institute, Yokohama, Japan; 9 Genome Science Laboratory, Discovery Research Institute, RIKEN Wako Institute, Wako, Japan; 10 Australian Research Council Special Research Centre for Functional and Applied Genomics, Institute for Molecular Bioscience, University of Queensland, Brisbane, Queensland, Australia; The Jackson Laboratory, US; MRC-Harwell, UK; NHGRI-NIH, US; Lawrence Livermore National Laboratory, US; The Jackson Laboratory, US

## Abstract

Using the two largest collections of Mus musculus and Homo sapiens transcription start sites (TSSs) determined based on CAGE tags, ditags, full-length cDNAs, and other transcript data, we describe the compositional landscape surrounding TSSs with the aim of gaining better insight into the properties of mammalian promoters. We classified TSSs into four types based on compositional properties of regions immediately surrounding them. These properties highlighted distinctive features in the extended core promoters that helped us delineate boundaries of the transcription initiation domain space for both species. The TSS types were analyzed for associations with initiating dinucleotides, CpG islands, TATA boxes, and an extensive collection of statistically significant *cis*-elements in mouse and human. We found that different TSS types show preferences for different sets of initiating dinucleotides and *cis*-elements. Through Gene Ontology and eVOC categories and tissue expression libraries we linked TSS characteristics to expression. Moreover, we show a link of TSS characteristics to very specific genomic organization in an example of immune-response-related genes (GO:0006955). Our results shed light on the global properties of the two transcriptomes not revealed before and therefore provide the framework for better understanding of the transcriptional mechanisms in the two species, as well as a framework for development of new and more efficient promoter- and gene-finding tools.

## Synopsis

Tens of thousands of mammalian genes are expressed in various cells at different times, controlled mainly at the promoter level through the interaction of transcription factors with *cis*-elements. The authors analyzed properties of a large collection of experimental mouse *(Mus musculus)* and human *(Homo sapiens)* transcription start sites (TSSs). They defined four types of TSSs based on the compositional properties of surrounding regions and showed that (a) the regions surrounding TSSs are much richer in properties than previously thought, (b) the four TSSs types are associated with distinct groups of *cis*-elements and initiating dinucleotides, (c) the regions upstream of TSSs are distinctly different from the downstream ones in terms of the associated *cis*-elements, and (d) mouse and human TSS properties relative to CpG islands (CGIs) and TATA box elements suggest species-specific adaptation. The authors linked TSS characteristics to gene expression through categories defined by the Gene Ontology and eVOC classifications and tissue expression libraries. They provided examples of the preference of immune response genes for TSS types and specific genomic organization. Their results shed light on the fine compositional properties of TSSs in mammals and could lead to better design of promoter- and gene-finding tools, better annotation of promoters by *cis*-elements, and better regulatory network reconstructions. These areas represent some of the focal topics of bioinformatics and genomics research that are of interest to a wide range of life scientists.

## Introduction

The computational identification and functional analysis of mammalian promoters has, to date, been constrained by the relatively small datasets of experimentally confirmed transcription start sites (TSSs). For example, promoters within dbTSS were recently updated with the mapping of 195,446 FANTOM2 mouse full-length cDNA sequences to 6,875 RefSeq mouse genes [1,2]. Functional analyses of these mammalian promoters have been restricted to shared transcription factor binding sites (TFBSs) between human and mouse datasets [2]. Using the same collection of promoters contained in dbTSS, Aerts et al. embarked on a characterization of promoters by extending their study to Drosophila melanogaster and Fugu rubripes [3]. Further characterization of mammalian promoters is dependent on the availability of experimentally verified TSSs that would complement and extend existing datasets represented by the FANTOM collection, dbTSS, the H-Invitational database [4], and RefSeq. The latest effort of the FANTOM3 consortium [5] has provided the scientific community with the largest collection of transcriptome data for Mus musculus (mouse), and has complemented this with CAGE tags of Homo sapiens (human). Based on these data we provide a comprehensive comparative analysis of mouse and human promoters that results in a number of new insights that help us to better understand the transcriptional scenario in these two species.

GC properties are well-known global factors that influence promoter characteristics and gene expression [3,6–9]. In addition, GC characteristics influence important DNA properties such as the “bendability” and curvature of the DNA helix and consequently influence the interplay of DNA and chromatin, which impacts transcription. We set out to characterize the regions immediately surrounding TSSs based on such compositional properties. Our determination of tentative TSS locations has been based on the use of CAGE tags [10] and ditags [11] enriched with additional independent pieces of evidence of transcript existence including 5′ expressed sequence tags, long 5′-SAGE, and the 5′ ends of fully sequenced cDNAs from full-length libraries.

In this study, we report several distinctive features in the extended core promoters that helped us delineate the boundaries of the transcription initiation domain space for both mouse and human, as well as delineate species-specific characteristics within that space. We describe the association of TSS types with the initiating dinucleotide, CGIs, TATA boxes, and an extensive collection of statistically significant *cis*-elements in mouse and human, and correlate TSS properties with expression data through comparison with Gene Ontology (GO) [12] and eVOC [13] categories, tissue expression libraries, and specific genome organization.

## Results/Discussion

### GC Content and TSS Types

**Figure 1:**
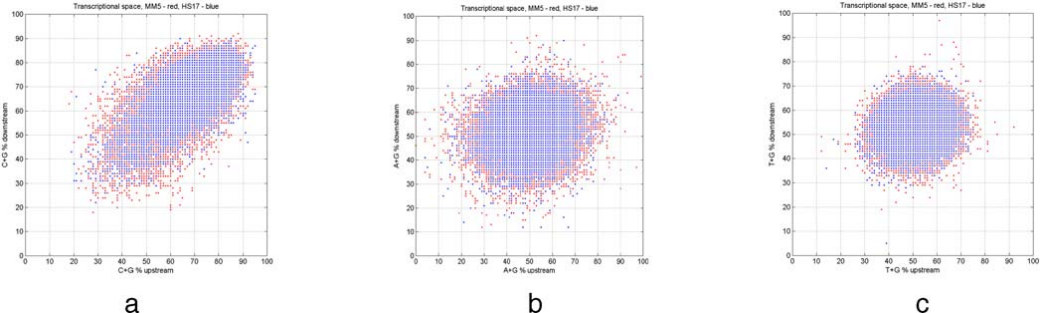
Transcription Initiation Domains for Mouse and Human Distribution of mouse (red) TSSs overlapped by human (blue) TSSs based on (A) C + G content, (B) A + G content, and (C) T + G content. Nucleotide content is determined for upstream [−100, −1] and downstream [+1, +100] regions relative to the TSS. The distribution of TSS locations is more or less random when viewed in terms of A + G content (B) or T + G content (C). Strong polarization of distributions is evident only in the G + C case (A).

We considered TSS properties based on the GC characteristics of the segments immediately upstream and downstream of experimentally estimated TSSs. We split TSSs into four distinct classes based on the GC content upstream and downstream of the TSS, as shown in Table 1 (see Materials and Methods). These four tentative TSS types have been used as a tool to investigate different promoter features in mouse and human. Two TSS types do not differ in GC richness between the upstream and downstream regions. They are GC-rich (GC-GC, type A) or AT-rich (AT-AT, type D) both upstream and downstream. The other two are GC-rich upstream and AT-rich downstream (GC-AT, type B) and, vice versa, AT-rich upstream and GC-rich downstream (AT-GC, type C). The distributions of TSS positions in the case of mouse and human are depicted in Figure 1. A strong polarization of the TSS distribution exists, with TSS types A and D being most prevalent (Figure 1A). The number of TSSs in each of the TSS types remains almost unchanged if the length of the upstream and downstream regions changes (Figure S1); it also only gradually changes with a change of threshold for GC richness (Figure S1). These findings suggest robustness of our TSS classification.

**Table 1:**
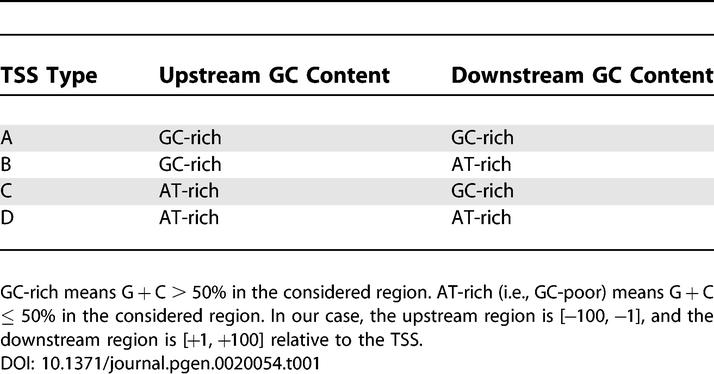
Four TSS Types Defined Based on the GC Content Upstream and Downstream of the TSS

### Are Two TSS Types (GC-Rich and AT-Rich) Sufficient to Consider?

**Figure 2:**
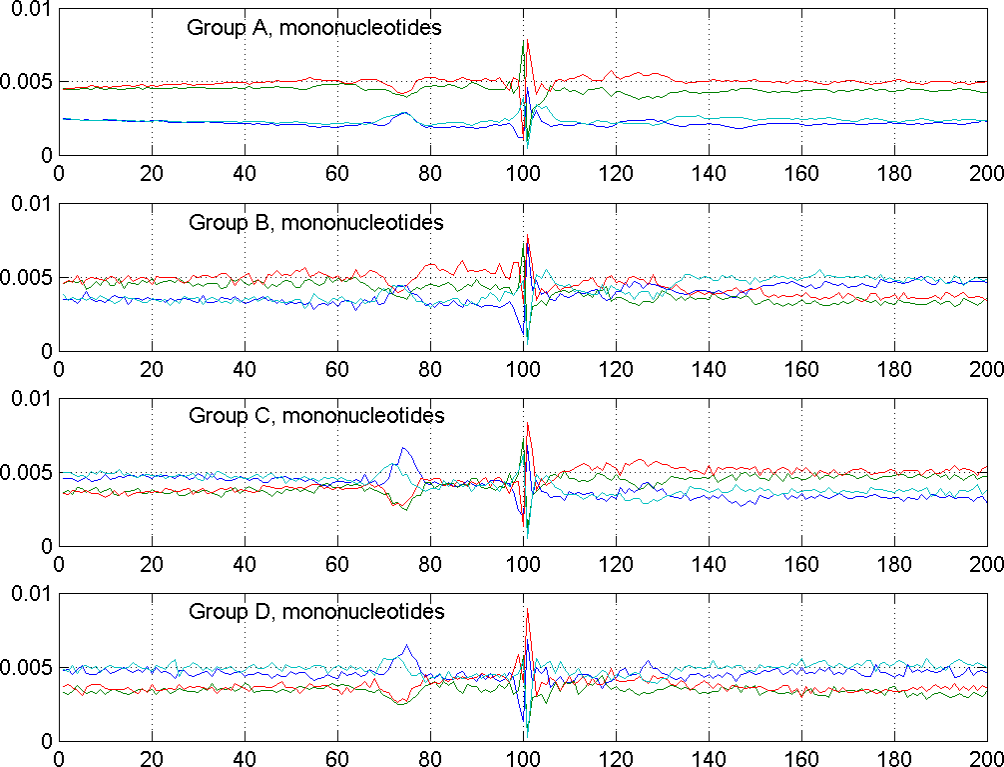
Distribution of Mononucleotides in Mouse Promoters in the Region Surrounding the TSS The nucleotides adenine, cytosine, guanine, and thymine are represented by blue, green, red, and light blue, respectively. The TSS types that are GC-poor upstream (C and D) show very characteristic enrichment in adenine and thymine nucleotides around [−35, −20], suggesting a potential dominant influence of TATA box and similar AT-rich elements in transcription initiation in these types. In type B and A TSSs, this influence does not seem to be dominant, but the presence of such elements is suggested by a significant reduction of the GC content in the [−35, −20] region. In principle, one could attempt to link the types of AT-rich upstream elements with initiating dinucleotides characteristic of different TSS types.

Promoters are usually classified as either GC-rich or AT-rich, without separating such properties into upstream and downstream characteristics relative to the TSS [3]. In our study we observed that many of the TSSs that are not evidently GC-rich (both upstream and downstream of the TSS) have changing GC content when going from upstream to downstream regions (Figure 2). The types of patterns were AT→GC, AT→AT, and GC→AT, containing 1,911, 1,528, and 1,440 instances, respectively, in our mouse TSS dataset. We found it reasonable to assign the TSSs with a change of GC content around the TSS (AT→GC and GC→AT) to different classes because they represent about 2/3 of all non-GC-GC types. We use this profiling of TSS characteristics as a methodological convenience. However, the biological justification for this relies on the fact that many *cis*-elements have a preference for GC-rich or AT-rich domains, as found in studies of promoter groups [14,15]. Thus, considering separately the GC-rich (AT-rich) upstream and downstream segments around TSSs provides an opportunity to analyze different groups of binding sites that may confer different transcription initiation scenarios.

An essential support for the biological relevance of our introduced TSS classification relies on the fact that some eukaryotic genomes have dominant TSS characteristics of the classes we defined. For example, based on the work of Aerts et al. [3], TSS types B and C appear prevalent in F. rubripes and type D in *D. melanogaster,* while type A is characteristic of the human genome. There are other ways to classify promoters using certain functional rather than compositional properties. Kadonaga [16] used the presence of functional core promoter elements (PEs) such as TATA boxes, initiators, and downstream promoter elements (DPEs) to classify promoters into several types. A different approach was used by Kim et al. [17]: the properties of preinitiation complex binding to promoter and the observed transcript expression state were used to define four promoter groups.

We found through several sources of evidence that expanding a crude classification of GC-rich and AT-rich TSSs by two additional subclasses makes biological sense and presents certain fine details more explicitly than is possible if all TSSs are lumped into only two (GC-rich and AT-rich) classes. Very obvious examples of such details, in addition to largely different compositions of the putative *cis*-elements that reside in the upstream and downstream regions, are (a) specialized, but different, initiating dinucleotides overrepresented in a statistically significant manner in TSSs of different types, (b) clear differences in the surrounding environment of the initiating dinucleotides between the four TSS types, and (c) different preferences of some functional gene groups for particular TSS types. These features cannot be observed if the groups are lumped.

### GC Content of TSS Surroundings Reflects Types of Putative *cis*-Elements

**Figure 3:**
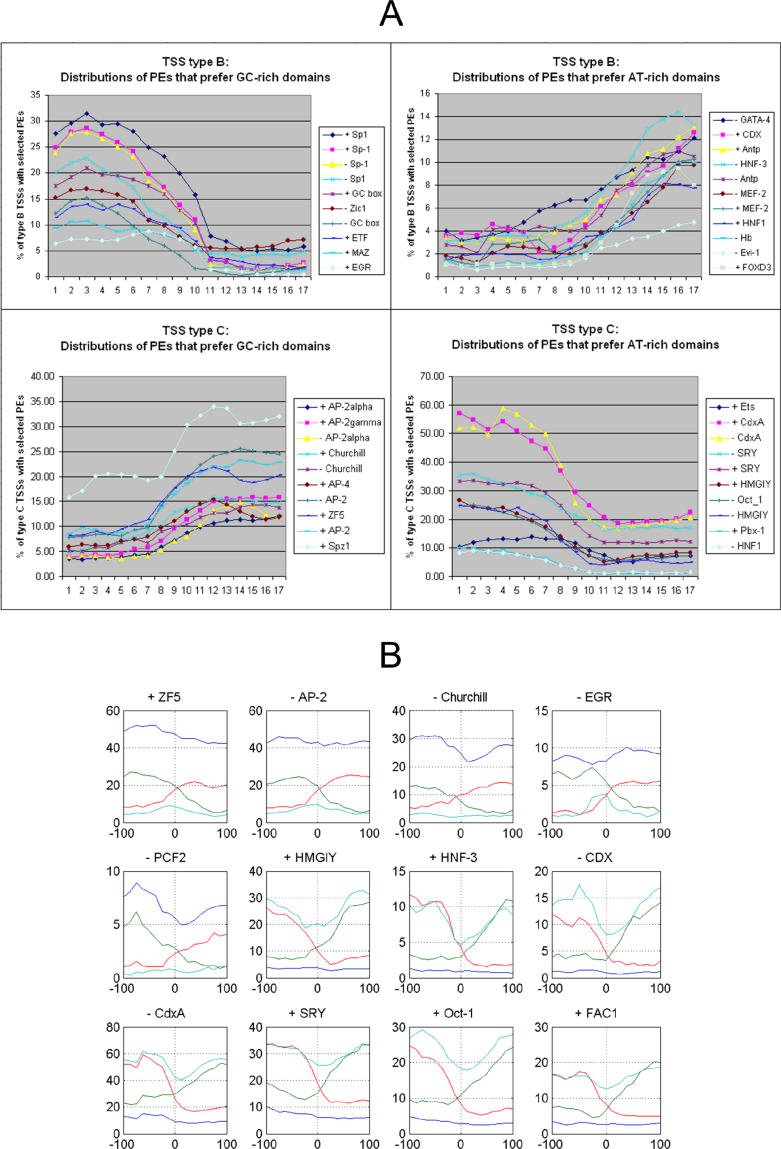
Distribution of Densities of Selected PEs in Promoters of the Four TSS Types in Mouse The density of PEs is calculated from the region covering [−100, +100] relative to the TSS. Density is determined for bins of length 50 bp and shifted by 10 bp. In total, there are 17 bins. The vertical axis shows the percentage of TSSs of the considered type that contain the PE. (A) Distribution of selected PEs that prefer GC-rich (left) and AT-rich (right) domains in type B (above) and type C (below) TSS groups. Bin number 9 is centered around the TSS. It can be seen that groups of PEs change significantly in their concentrations in transition from upstream to downstream regions and characterize two distinct TSS types (B and C). (B) Distribution of selected PEs across all four TSS types. Blue, green, red, and light blue correspond to distributions characterized by type A, B, C, and D TSSs. The first five PEs are those that prefer GC-rich regions, and the last seven PEs prefer AT-rich regions (the plus or minus sign in front of the TFBS symbol denotes the strand where the TFBS is found).

By considering the GC content upstream and downstream separately, we allowed for one more degree of freedom in observing global TSS properties. Here we denote a PE as a TFBS and the strand where it is found. Many PEs have preferences for either GC-rich or GC-poor regions [14,15]. For example, the well-known TATA box element, being AT-rich, will be found more frequently in AT-rich regions, while the Sp1-binding sites, being GC-rich, will be found more frequently in GC-rich regions. Thus, the four TSS types that we consider could be correlated in a global manner with the potential PEs that may control the respective genes. Support for the influence of potential PEs on specific TSS types is obtained from the distributions of PE densities (Figure 3). Density distributions of selected PEs that prefer GC-rich (AT-rich) domains in type B and type C TSSs are depicted in Figure 3A. We observe that PE groups change their concentrations significantly in transition from upstream to downstream regions. Moreover, in Figure 3B we present distributions for selected PEs across all four TSS types. The first five PEs in Figure 3B (+ZF5, −AP-2, −Churchill, −EGR, and −PCF2) are those that prefer GC-rich regions (the plus and minus signs in front of the TFBS symbols denotes the strand where the TFBS is found). It is interesting to observe that these PEs occur in high concentrations in the type A group (GC-GC), occur in considerably lower concentrations in type D (AT-AT), and follow the change of GC content in types B and C. We observe the converse for the remaining seven PEs, which prefer AT-rich regions. These properties suggest that the four TSS types selectively associate with different groups of PEs.

### Upstream and Downstream Regions Are Different: Enrichment by Specific PEs

**Figure 4:**
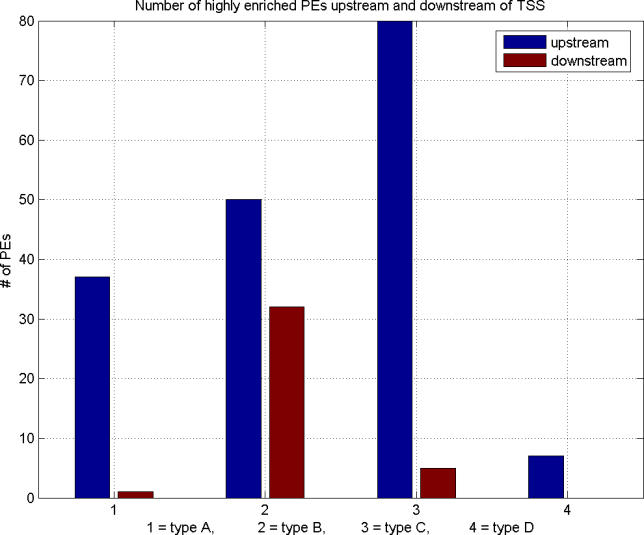
Distribution of Selected Groups of PEs That Are Highly Enriched (at Least 3-Fold) Upstream or Downstream of the TSS The upstream region considered covers [−100, −1], while the downstream region covers [+1, +100] relative to the TSS. In all TSS types, the upstream region contains significantly more enriched PEs than the downstream region.

We analyzed the preference of upstream and downstream regions in the four TSS types for significantly enriched (at least 3-fold) PEs in one region as opposed to the other region. The results are presented in Figure 4. To our surprise, we found that for all TSS types the number of enriched PEs in the upstream region is much higher than in the downstream region. In three types (A, C, and D) the number of PEs in the downstream region is minimal compared to the upstream region. The only exception is type B, for which there are a significant number of enriched PEs in the downstream region. The data suggest for type A TSSs a high influence of PEs that reside upstream and prefer GC-rich domains, while for type C TSSs such influence is likely through PEs that are located upstream of the TSS but prefer AT-rich domains. Contrary to these patterns, promoters with type B TSSs seem to utilize a mix of both GC-rich-preferring and AT-rich-preferring PEs. A conclusion cannot be made for type D TSSs because of the very small number of highly enriched elements overall. Moreover, applying the Chi-square test for the equality of distributions in the upstream and downstream regions we get *p* = 1.34 × 10^−07^, which strongly rejects the null hypothesis that these distributions are the same. All these findings suggest that upstream and downstream regions should be considered separately (as we do). The results emphasize enrichment of different PE groups associated with upstream and downstream regions in the promoters of the four TSS types.

### Four TSS Types Associate with Different Sets of PEs

Different compositional properties of the four TSS types suggest that the TSSs may be controlled by specialized collections of transcription factors (TFs). Thus, we attempted to find the potential TFs that could play dominant roles in the four TSS types by identifying (a) the specificity of the top-ranked PEs (relative to overrepresentation index [ORI]; see Materials and Methods) in different TSS types, (b) unique and common motifs in the GC-rich/AT-rich upstream/downstream regions for different TSS types, and (c) the most significant PEs/TFs upstream/downstream of TSSs of types A, B, C, and D.

To carry out these analyses we initially compared the incidence of predicted DNA-binding sites of known TFs in the different promoter segments in mouse in the four TSS types against those in random mouse DNA. For the top 150 predicted motifs (representing approximately 10% of all elements found in these comparisons) determined based on ORI [15], we calculated Bonferroni corrected *p*-values for enrichment in the considered promoter segments. In the selection of these top 10% of motifs we required that they be present in at least 10% of the promoters in the target groups and that they have an ORI value not less than 1.5. In these comparisons we found that the corrected *p*-value was below the threshold of 0.05 for the great majority of cases. These comparisons indicate that most of the motifs for the considered TSS types are highly specific relative to random DNA (Table S1).

Next we aimed to see if promoter segments with the same GC richness share the same set of PEs. We compared the upstream regions of groups A versus B and C versus D, and the downstream regions of groups A versus C and groups B versus D. It is interesting to note that the upstream (GC-rich) regions of type A and B TSSs do share, as expected, a subset of predicted motifs, but each type is characterized also by a specialized collection of putative binding sites that do not appear in the top 150 ranked sites of the other type (for example, ETS appears in promoters of type B TSSs, but not in promoters of type A TSSs) (Table S2).

Even those TFs that are found to be common in the upstream parts of both type A and type B TSSs appear in significantly different proportions of promoters of these types, as summarized in Figure S2. For example, Ets (Table S1) appears in AT-rich downstream segments (types B and D). However, in type B TSSs it appears in 17.08% of promoters, but only on the minus strand, while in type D it appears in 13.48% of promoters, but only on the plus strand.

Moreover, if we consider unique motifs that appear in different groups, they are commonly present in large proportions of promoters of those target groups. For example, in transcripts initiated from type D TSSs, we find only two unique PEs in the downstream region. One is DBP, a transcriptional activator in hepatic cells [18] and member of the C/EBP family, which appears in 26.77% of promoters with type D TSSs and only on the minus strand. The other element, Ncx, is enteric neuron homeobox and acts as an activator [19] that is required for proper positional specification, differentiative cell fate, and maintenance of proper function of enteric neurons [20,21]. It is present in 41.75% of promoters with type D TSSs and only on the plus strand.

Since any two of the four TSS types could differ in their GC content in the upstream, downstream, or both regions, and consequently harbor different sets of significant motifs, we conclude that, overall, TSS types contain sets of significant signature motifs (denoted by a plus sign next to the ORI value in Table S1 and a plus sign in Table S2) that potentially may contribute to orientation, and are likely to interact with distinct set of TFs. This concurs with the results of the preceding two subsections and suggests overall different transcriptional programs present in the transcripts of these TSS types. Lists of the most significant PEs that appear in the TSS groups are provided in Table S3.

### The Initiating Dinucleotide and Its Environment

We analyzed in mouse and human datasets the initiating dinucleotide, that is, the one that occupies positions [−1, +1] relative to the TSS. We found that a number of different initiating dinucleotides are statistically significant across various TSS types and that they show certain regularities related to the GC content of upstream and downstream regions surrounding the TSS. Table 2 shows for mouse and human data all statistically significant cases based on the *p*-value obtained by the right-sided Fisher's exact test and corrected for multiplicity testing by the Bonferroni method. The association of initiating dinucleotide to TSS properties is very specific. It is interesting to note that the initiating dinucleotide TA is significantly enriched in TSS types that are AT-rich upstream, downstream, or both (B, C, and D), while dinucleotides that start with guanine (GA or GG) are significantly enriched in TSS types that are AT-rich specifically downstream (B and D). Type A TSSs are significantly enriched for dinucleotides that start with cytosine (CC, CG, and CT). However, the canonical initiating dinucleotide CA appears statistically significant only for TSS types that change GC richness (B and C). Finally, the TSS type C group contains AG and TG dinucleotides at a statistically significant level, while these do not appear significant in any other TSS type.

**Table 2:**
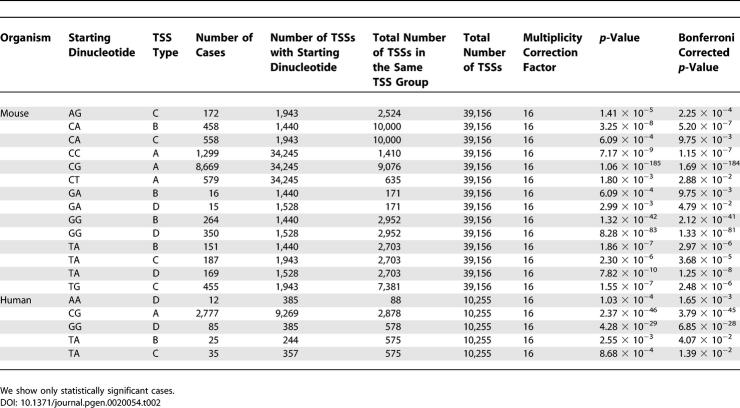
Starting Dinucleotide [−1, +1] for Various TSS Types in Mouse and Human Datasets

This compositional property of the initiating dinucleotide being linked in a statistically significant manner to the GC properties of the upstream and downstream regions would not be detectable if the TSS groups were lumped. We see that these properties characterize significant numbers of TSSs in our mouse dataset, namely, 10,547 (30.80%), 889 (61.74%), 1,372 (70.61%), and 534 (34.95%) of TSSs of type A, B, C, and D, respectively, and thus they do not appear to be artifacts of the proposed TSS classification that we have introduced. The conclusion is that the initiating dinucleotides show specific preferences at statistically significant levels to different TSS environments and that a significant portion of TSSs in our datasets are characterized by these initiating dinucleotides. Moreover, almost all of them are different from the canonical CA dinucleotide.

**Figure 5:**
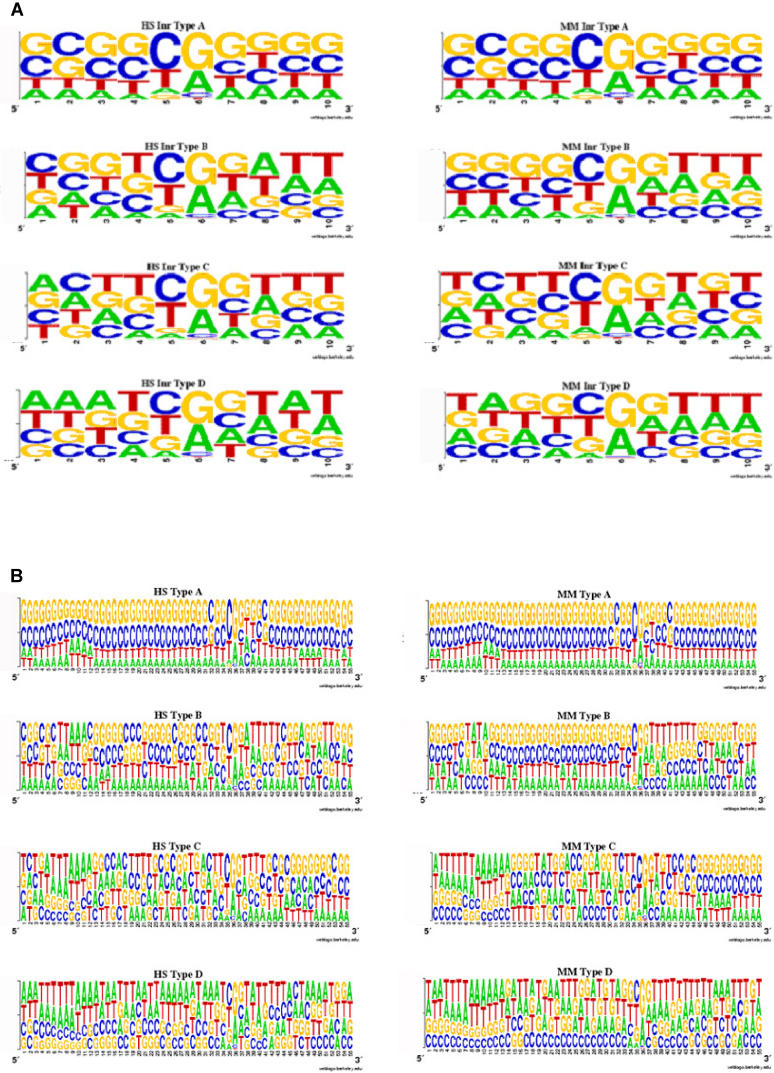
Sequence Logos (A) Sequence logos for Inr in human (left) and mouse (right) obtained using [−5, +5] segments relative to TSS locations. There is an evident bias in the nucleotide composition surrounding the TSS that effectively determines different Inr elements. (B) Sequence logos for segments [−35, +20] relative to TSS locations. Strong similarity exists between human (left) and mouse (right) in TSS type A, while that similarity is considerably reduced for the other TSS types.

This last observation leads us to hypothesize that different TSS types may be controlled by different initiator (Inr) elements. Figure 2 depicts the quite different composition of the regions immediately surrounding tentative TSSs. The Inr elements—if they appear biologically relevant for these groups—should overlap TSSs and may be qualitatively different for different TSS types. Different initiating dinucleotides of highly statistically significant enrichment support such a hypothesis, and, at the same time, the variability of the observed initiating dinucleotides could explain the nonspecific consensus of the octamer Inr element [22]. We have generated sequence logos of the TSS surroundings [−5, +5] in both mouse and human, and present them in Figure 5A. We observe that the nucleotide distributions for type A (GC-GC) TSSs are about the same in mouse and human. However, for TSS types B, C, and D, there is evident difference in these distributions in the region surrounding the TSS, which does not contradict our hypothesis of potentially different Inr elements for different TSS types. Figure 5B shows logos of regions [−35, +20] for the four TSS types in mouse and human. Again, we observe significant similarity between the species in the composition of the region for type A TSSs, while the other TSS types show significantly more variability. This may suggest species-specific organization of the core promoters for these minority TSS types (B, C, and D).

### Relation of TSS Types to TATA Box Elements and CpG Islands

We analyzed the four TSS types in mouse and in human (Tables 3 and 4) for the presence of TATA box elements and association with CGIs. Globally, there are similarities in these properties of TSS types between these two species, but there are also significant differences. This mouse–human comparison must be treated with some caution, since the mouse and human datasets are based upon analysis of distinct tissues, and the human set is probably less comprehensive. In some measure, the distinctions may also relate to depth of coverage in the two species. However, since we considered a statistically large number of well-defined TSS locations in mouse (39,156) and in human (10,255), this makes comparison between the two species feasible.

**Table 3:**
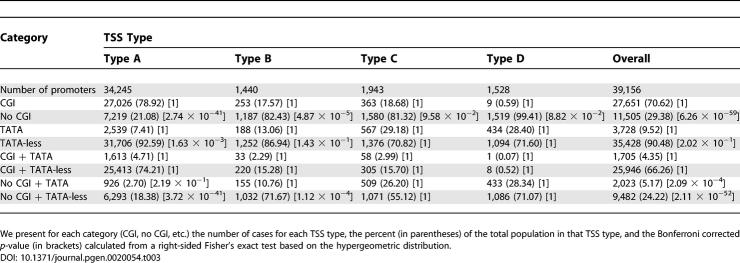
Basic Statistics on Relation of TATA Box Motifs, CGIs, and Four TSS Types for MM5 Transcripts

**Table 4:**
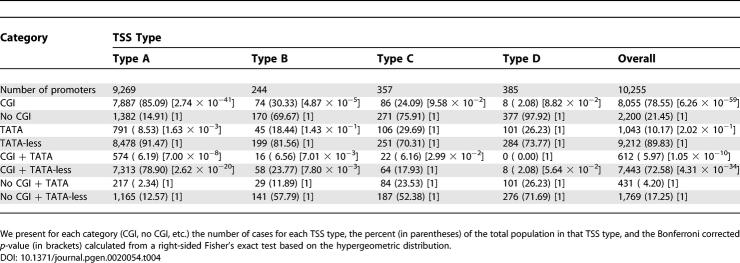
Basic Statistics on Relation of TATA Box Motifs, CGIs, and Four TSS Types for HS17 Transcripts

Based on Bonferroni corrected *p*-values we find that the mouse and human datasets differ significantly in a number of promoter features (Tables 3 and 4). Mouse promoters are significantly enriched in (a) the number of promoters not associated with CGIs in TSS types A and B, and overall; (b) the number of TATA-less promoters in group A; (c) the overall number of promoters that have TATA boxes but are not associated with CGIs; and (d) the number of TATA-less promoters not associated with CGIs in TSS groups A and B, and overall. Conversely, human promoters are significantly enriched in (a) the number of promoters associated with CGIs in TSS types A and B, and overall; (b) the number of TATA-box-containing promoters in TSS type A; (c) the number of TATA-box-containing promoters associated with CGIs in TSS types A, B, and C, and overall; and (d) the number of TATA-less promoters associated with CGIs in TSS types A and B, and overall. These data suggest that there are species-specific solutions for transcriptional initiation in mouse and human for the analyzed TSS types.

There are a number of core PEs other than TATA boxes and Inr elements, such as the downstream promoter element (DPE) [23–26], the TFIIB response element (BRE) [27], the motif ten element (MTE) [28], and the downstream core element (DCE) [29,30]. It would be of interest to investigate their presence around mammalian TSSs. Unfortunately, such an analysis represents a study on its own and requires reliable matrix models of these elements in mammals that are not yet available.

### Linking TSS Properties and Gene Expression

We were interested to find out if the TSS types show any correlation with broad expression categories. We used association of transcripts with different GO [12] and eVOC [13] categories, as well as FANTOM3 tissue expression libraries, and analyzed their TSS distribution across the four types in mouse. While it is not possible to make definite conclusions because of incomplete GO, eVOC, tissue library, and transcript data, we were able to find a number of classes that associate with specific TSS types in a statistically significant manner (Tables 5, 6, S4, and S5). Moreover, we searched for ortholog transcript groups in mouse and human whose promoters preserve enrichment in specific TSS types in both species (Table S4). Under the conditions of our study we found that 100% of GO categories whose mapped transcripts emanate from type B TSSs preserve their enrichment; this is true for 64% of GO categories associated with type C TSSs and for 80% of GO categories associated with type D TSSs. These results suggest that between mouse and human the TSS character within the GO categories is largely conserved. Distributions of all mouse TSSs across the four TSS types for GO categories and FANTOM3 tissue libraries are provided in Table S5.

**Table 5:**
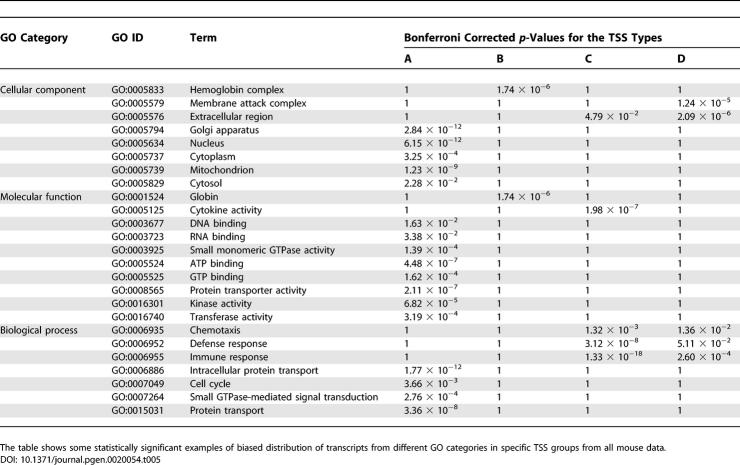
Enrichment of TSS Types in Selected GO Categories in Mouse

**Table 6:**
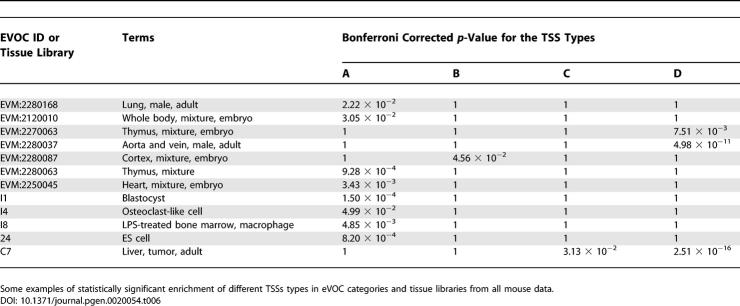
Enrichment of TSS Types in Selected eVOC Categories and Tissue Libraries in Mouse

**Figure 6:**
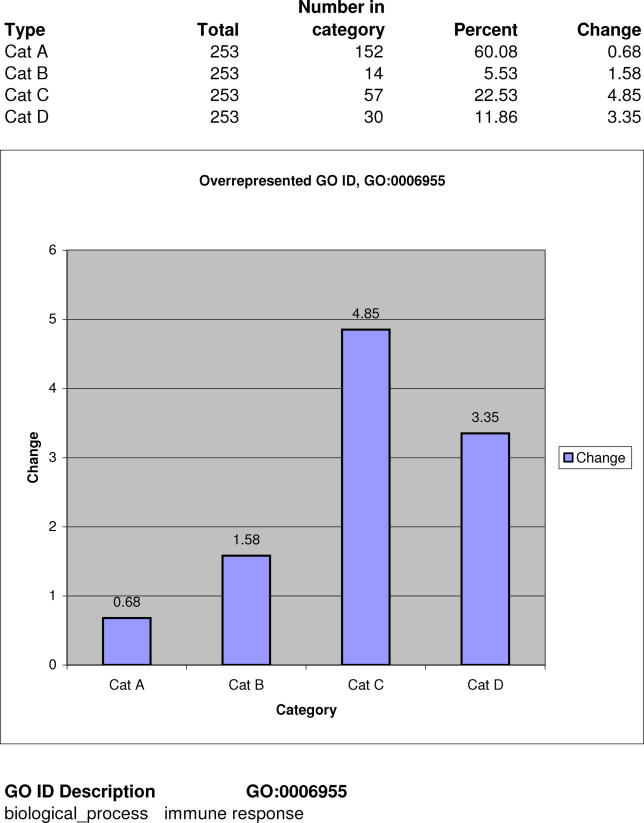
Distribution of TSSs for Transcripts Related to Immune Response through GO:0006955 There are 1.58-, 4.85-, and 3.35-fold more transcripts having TSS types B, C, and D than one would expect based on the proportion of transcripts in these groups in our reference mouse data. Enrichment is statistically significant for types C and D based on Bonferroni corrected *p*-values obtained by the right-sided Fisher's exact test (Table 5).

We further analyzed several specific cases. For many GO categories we found that transcripts associated with them prefer specific GC-rich/GC-poor transcription initiation frameworks (Table 5). For example, the immune response group (GO:0006955) (Figure 6) appears with 1.58-, 4.85-, and 3.35-fold more transcripts having TSSs of type B, C, and D, respectively, than one would expect based on the proportion of transcripts in these groups in our reference mouse data. The enrichment in type C and D TSSs is statistically significant (Bonferroni-corrected right-sided Fisher's exact test, *p* = 1.33 × 10^−18^ and *p =* 2.60 × 10^−4^, respectively). Based on this, we conclude that the transcript group GO:0006955 is characterized by increased participation of transcripts from TSS types that are AT-rich upstream or downstream. We analyzed in more detail the genomic organization of loci corresponding to genes from the most overrepresented TSS type (type C) for this GO. We found that TSSs of type C map to 36 nonredundant genes, of which two are in bidirectional promoters (2/36), which means these are underrepresented for type C TSSs relative to the genome average. There are 23 genes (64%) that are appearing in gene family clusters, that is, these genes are highly overrepresented for type C TSSs relative to the genome average. Finally, genes with type C TSSs have small genomic span: 34 out of 36 are less than 25 kb long, which is again more than one would expect based on the genome average. Most genes in the category GO:0006955 are short (the majority are actually less than 10 kb), are clustered with other members of the same families, and are not bidirectionally transcribed. This analysis illustrates a specific genomic organization of genes with TSSs of type C in this GO group. Thus, TSS properties may be associated with genomic organization.

In Table 5, one can see that GC-rich TSSs relate to genes responsible for various binding and protein transport activities. These functions usually occur in different regions of the cell and are reflected in the diverse compartments that are enriched for type A TSSs. AT-rich TSSs (types C and D), on the other hand, are enriched in processes relating to defense responses to the environment. TSSs of the membrane attack complex (GO:0005579), defense response (GO:0006952), and immune response (GO:0006955) are enriched in type D TSSs, while the last two of these (defense and immune response) and cytocine activity (GO:0005125) are enriched in type C TSSs. Globin group (GO:0001524) and hemoglobin complex (GO:0005833) are enriched in type B TSSs. These findings suggest a preference of different functional transcript groups for specific TSS types.

Similarly, for transcript groups based on eVOC terms, we find that they prefer GC-rich or GC-poor transcription initiation frameworks, depending on the eVOC category. For example, thymus-expressed transcripts (EVM:2270063 and EVM:2280063) (Table 6) seem to prefer either type A or D TSSs. The same is the case for transcripts classified according to cardiovascular function (EVM:2280037 and EVM:2250045) (Table 6).

### Conclusions

We have introduced a different way to characterize TSSs, which connects TSS properties to the GC content of the immediately upstream and downstream regions. This implicitly links the TSS type with PEs that are residing in the TSS neighborhood. We were able to delineate transcription initiation active domains in the mouse and human genomes and observed fundamental similarities in the transcription initiation active domains in the two species. Looking separately at the GC content upstream and downstream of TSSs provides a useful paradigm to view certain phenomena in a clearer and more meaningful manner. We found that two of the TSS types, types C and D, possess positionally very well defined AT-rich regions [−35, −20] relative to the TSS, suggesting the significant role of AT-rich sequences such as TATA boxes in the control of TSSs of these types. Our analysis documents that various initiating dinucleotides show very specific preferences for the TSS types we considered, are present in statistically significant proportions of the TSSs in our datasets, and are almost all different from the consensus dinucleotide. Very specific sets of initiating dinucleotides are associated with different TSS types, and surrounding GC content is well correlated with the types of these dinucleotides. This suggests the potential presence of different Inr elements that may be characteristic for each of the TSS types and associated with different nucleotide characteristics of the surrounding domain.

We have shown that different TSS types associate with different PEs, that regions upstream and downstream of different TSS types are characterized by different collections of PEs, and that the putative PE content (for the top 10% of PEs) of the TSS surroundings generally differs for the TSS types. All these findings suggest likely control of the respective transcripts by different collections of significant PEs residing upstream or downstream of the TSS. Our results on TSS properties relative to CGIs, TATA boxes, and Inr elements in mouse and human suggest species-specific adaptation. Finally, we have shown a number of examples of transcript groups obtained on the basis of different ontologies or tissue libraries that have statistically significant enrichment in at least one of the TSS types. This has provided a link between TSS characteristics and expression data.

We believe that the results of this analysis will help in better understanding the general transcription regulation properties of mammalian promoters, and prove useful for further development and enhancement of promoter and gene prediction tools.

## Materials and Methods

### TSSs.

We constructed two highly accurate sets (one for mouse and one for human) of TSSs and of the promoter sequences covering the span [−100, +100] relative to these TSSs. These datasets are available at http://www.sanbi.ac.za and were obtained as follows. If the first 5′ nucleotide of the CAGE tag or 5′ ditag (http://fantom31p.gsc.riken.jp/cage_analysis/export) coincided with the first 5′ nucleotide of the full-length cDNA (http://fantom.gsc.riken.go.jp/download.html), the TSS determined by this tag was selected. Also, in cases when this condition did not hold, we selected TSSs based on the following requirements: the TSS is a representative TSS location from a tag cluster that has at least ten tags, the representative TSS is supported by at least six tags, and there is at least one other piece of transcriptional evidence associated with this tag cluster (expressed sequence tag, full-length cDNA, or long SAGE; http://fantom.gsc.riken.go.jp/download.html). In this way, we compiled a mouse reference promoter set of 39,156 promoters and a human reference promoter set of 10,255 promoters. These two sets are used for all our analyses.

Randomly selected DNA sequences from mouse were used as the background set for analysis of TF binding sites in mouse promoters. These DNA sequences were 200 bp long and selected randomly from all mouse chromosomes, with the number of sequences from each chromosome proportional to the length of the chromosome. In total we selected 41,000 such random DNA sequences (Dataset S1).

### TSS types.

We determined the GC content of the [−100, −1] region and the [+1, +100] region relative to TSS location for each individual TSS. The TSS is considered to be between positions −1 and +1. The upstream or downstream segment was defined as GC-rich if G + C > 50% in the region. Otherwise, the region was defined as AT-rich. Four types of TSSs were defined based on the GC richness in the upstream and downstream segments as follows (Table 1): type A, GC-rich upstream and downstream (GC-GC); type B, GC-rich upstream and AT-rich downstream (GC-AT); type C, AT-rich upstream and GC-rich downstream (AT-GC); and type D, AT-rich upstream and downstream (AT-AT). Each TSS can be represented as a point in the *x–y* plane, where *x* corresponds to the GC content upstream and *y* corresponds to the GC content downstream of the considered TSS. For mouse and human these distributions are depicted in Figure 1A.

### TF binding sites in promoters.

We used all available matrix models of TFBSs contained in the TRANSFAC Professional (version 8.4) database [31] and mapped them to the extracted sequences. We used minSUM profiles for the threshold of the matrix models since these contain the optimized threshold values for the core and matrix scores [32]. The thresholds in minSUM are based on optimization that provides the minimum sum of false positive and false negative TFBS predictions. To determine the overrepresentation of TFBSs found in the target set, we used the method of Bajic et al. [15]. All TFBSs mapped to target promoters were ranked based on their ORI as defined by Bajic et al. [15]. For ORI = 1 or close to this value, there is no overrepresentation of the motif in the target promoter group. We also estimated the likelihood of observing these TFBSs in the target set using the background random promoter set as a reference. The null hypothesis was that the proportion of sequences in the target set in which a particular PE was found was the same as that in the background set. The *p*-values were calculated using right-sided Fisher's exact tests based on hypergeometric distribution. The original *p*-values were subjected to Bonferroni correction for multiplicity testing. If the corrected *p*-value of the pattern was not greater than 0.05, we placed a plus sign after the ORI value in the provided tabular reports.

### Most significant PEs.

For each of the TSS types in mouse, we analyzed the 150 top-ranked PEs (based on the values of ORI). This represents about 10% of all (1,428) PEs analyzed. We also required that the PEs have an ORI of at least 1.5 and that the PE be found in at least 10% of the target sequences. Details are explained in Tables S1–S3.

### TATA boxes.

The TATA box model used was based on that of Bucher [22]. The threshold used was 0.75, while score was normalized between zero and one (analogous to Bajic et al. [33]). A TATA box was considered detected if the maximum value of the score in the [−50, −1] region was higher than the threshold. Only one TATA box was assumed in the [−50, −1] region.

### eVOC, GO, and tissue expression libraries.

In order to assess the biological significance of our TSS classification system, we assigned TSSs according to different GO and eVOC categories, as well as tissue libraries in FANTOM3 collection (http://fantom.gsc.riken.go.jp/download.html). GO–FANTOM mapping data was downloaded from the RIKEN Web site (ftp://fantom.gsc.riken.jp/FANTOM3/annotation/fantomdb-3.0/anndata.txt.gz). The eVOC system consists of a set of orthogonal controlled vocabularies that unify gene expression data by mapping between the genome sequence and expression phenotype information. The eVOC human anatomy ontology [13] and the newly developed mouse adult and developmental ontologies (http://www.evocontology.org) have been mapped to the FANTOM3 library descriptions, providing a hierarchical representation of tissues, cell types, and developmental stage information. This allows for a standardized analysis of gene expression and promoter profiles independent of the original annotation vocabulary used in the original dataset.

For the generation of the results presented in Table S4, we used ortholog gene groups between mouse and human as defined at ftp://ftp.ncbi.nih.gov/pub/HomoloGene. Table S5 for mouse data contains statistics of all GO and tissue expression libraries from FANTOM3, complemented by the Bonferroni corrected *p*-value (right-sided Fisher's exact test based on hypergeometric distribution) for the null hypothesis that the proportion of TSSs of a specific type in the considered GO/tissue library is the same as what one can expect based on the distribution of these TSSs in mouse.

## Supporting Information

Dataset S1 Supplementary Nonpromoter Data (2.5 MB ZIP)

Figure S1 Number of TSSs of the Four Types in Human and Mouse Genomes under the Change of Parameters Blue, green, red, and light blue correspond to TSSs of type A, B, C, and D, respectively. From graphs in the first row we observe that when the length of the region considered changes, the numbers of TSSs of the different types remain almost unchanged. We changed the length of upstream and downstream regions from [−*x*, −1] and [+1, +*x*], respectively, with values of *x* from 50 to 150. From graphs in the second row we observe that the numbers of TSSs within the four types gradually change with the change of threshold for GC content. We changed this threshold from 40% to 60%.
(24 KB PDF)

Figure S2 Distributions of TFs Found to Be Common among the Top 150 PEs in Comparisons of Different TSS Types (A) Comparison of types A and B upstream regions.
(B) Comparison of types B and D downstream regions.
(C) Comparison of types A and C downstream regions.
(D) Comparison of types C and D upstream regions.
(40 KB PDF)

Table S1 List of Top 150 PEs That Appear with a Frequency of 10% or Greater in Upstream and Downstream Regions of Different TSS Categories Comparison is carried out against a background of random mouse sequences. Ranking is based on ORI value. The higher the ORI, the higher the rank. We present results for the four TSS types (A, B, C, and D). For each PE we give the strand where it is found (+1 or −1), name of TFBS, ORI value, percentage of promoters in the target set that contain the PE, percentage of sequences in the background set that contain the PE, probability of finding the PE in the target set (given as one prediction per nucleotide), probability of finding the PE in the background set (given as one prediction per nucleotide), and Bonferroni corrected *p*-value. A plus sign added after the ORI value indicates that the PE is enriched in a statistically significant manner at the level 0.05. Almost all top-ranked elements appear to be statistically significantly enriched in the target sets.
(329 KB PDF)

Table S2 Common and Specific TFBSs in the Four TSS Types PEs are compared relative to the same GC richness and same location (upstream or downstream) in different TSS types. The signs plus or minus indicate whether the PE was found to be significantly enriched in the considered region for the considered TSS type. For example, the first column (yellow), which shows comparison between the AT-rich downstream domains in TSS types B and D, contains a common element denoted as “+ − Ets.” This means that Ets was found significantly enriched for the B type, but its enrichment was not significant for D type. When an element is unique for one or another group, then it is associated only with one plus or minus sign.
(53 KB PDF)

Table S3 List of Significant PEs Unique and Common for Different TSS Types in the Upstream and Downstream Segments The yellow highlighted TFs are unique for the considered groups when compared with the same upstream or downstream segment of another TSS type with the same GC richness.
(47 KB PDF)

Table S4 GO Categories That Preserve Enrichment in Specific TSS Types between Human and Mouse We considered only those TSSs whose generated transcripts belong to the same homology group as defined on the NCBI Web site (ftp://ftp.ncbi.nih.gov/pub/HomoloGene). We only considered GO categories that were supported by at least 60 TSSs and where the target TSS type was supported by at least three TSSs.
(96 KB PDF)

Table S5 All GO and Tissue-Specific Libraries with Distribution of TSSs across the Four TSS Types in Mouse The table presents the total number of TSSs associated with the category (GO or expression library), the number of TSSs of individual TSS type, the percentage of TSSs in that TSS type, enrichment of TSSs in the TSS type relative to what can be expected based on the distribution of all TSSs in mouse across all four TSS types, and Bonferroni corrected *p*-values calculated based on right-sided Fisher's exact tests for the null hypothesis that the proportion of TSS type found in the target group is the same as that of the general mouse distribution. For example, there are 253 transcripts associated with GO:0006955. Of these, 52 transcripts have a TSS of type C. For the number of transcripts in this GO category, one would expect only 11 transcripts with TSSs of type C. Thus, in this GO category, we have 4.85-fold enrichment of transcripts of this type (compared to what we would expect based on the distribution of all transcripts across the four TSS types). If in any of the GO/eVOC categories or tissue libraries, at least one of the TSS groups of transcripts has enrichment that is 1.5-fold or greater than the expected value, we consider such TSS type overrepresented.
(2.9 MB PDF)

## Abbreviations

CGI: CpG island
GO: Gene Ontology
Inr: initiator
ORI: overrepresentation index
PE: promoter element
TF: transcription factor
TFBS: transcription factor binding site
TSS: transcription start site
