# Supplementary material for: Mice and Men: Their Promoter Properties
Source: PLoS Genet. 2006 Apr 28;2(4):e54. doi: 10.1371/journal.pgen.0020054 (PMC1449896; doi:10.1371/journal.pgen.0020054)
Supplement: Figure S1 — Blue, green, red, and light blue correspond to TSSs of type A, B, C, and D, respectively. From graphs in the first row we observe that when the length of the region considered changes, the numbers of TSSs of the different types remain almost unchanged. We changed the length of upstream and downstream regions from [−x, −1] and [+1, +x], respectively, with values of x from 50 to 150. From graphs in the second row we observe that the numbers of TSSs within the four types gradually change with the change of threshold for GC content. We changed this threshold from 40% to 60%. (24 KB PDF) [file pgen.0020054.sg001.pdf]

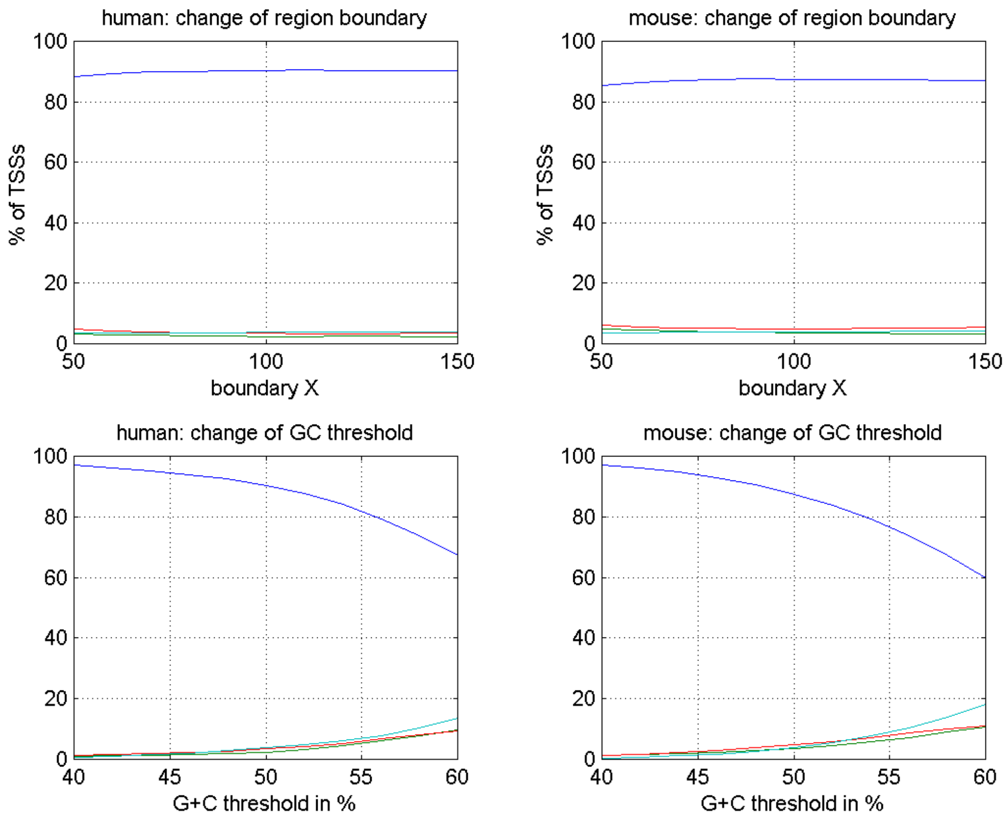

Figure S1. Number of TSSs of the four types (A,B,C,D) in human and mouse genome under the change of parameters. Blue, green, red and light blue correspond to TSSs of type A, B, C and D, respectively. From graphs in the first row we observe that when the length of the region considered changes, the number of TSSs of different types (A, B, C, D) remain almost unchanged. We changed length of upstream and downstream regions from [-X,-1] and [+1,+X] respectively, changing X from 50 to 150. From graphs in the second row we observe that the number of TSSs within the four types gradually change with the change of threshold for the GC content. We changed this threshold from 40% to 60%.
