# Supplementary material for: Mice and Men: Their Promoter Properties
Source: PLoS Genet. 2006 Apr 28;2(4):e54. doi: 10.1371/journal.pgen.0020054 (PMC1449896; doi:10.1371/journal.pgen.0020054)
Supplement: Figure S2 — (A) Comparison of types A and B upstream regions. (B) Comparison of types B and D downstream regions. (C) Comparison of types A and C downstream regions. (D) Comparison of types C and D upstream regions. (40 KB PDF) [file pgen.0020054.sg002.pdf]

Figure S2. Distributions of TFs found to be common among the top 150 PEs in comparisons of different TSS types

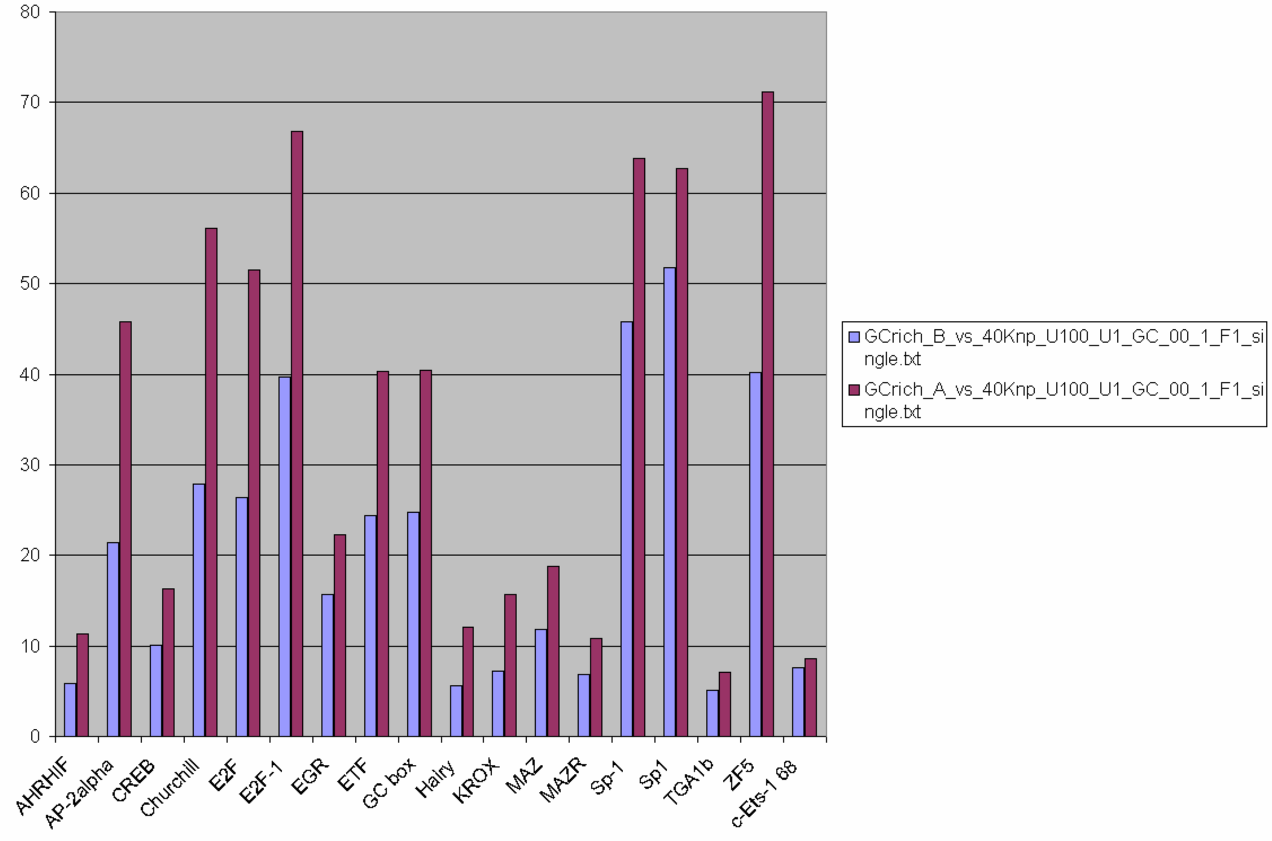

a/ Comparison of types A and B upstream regions

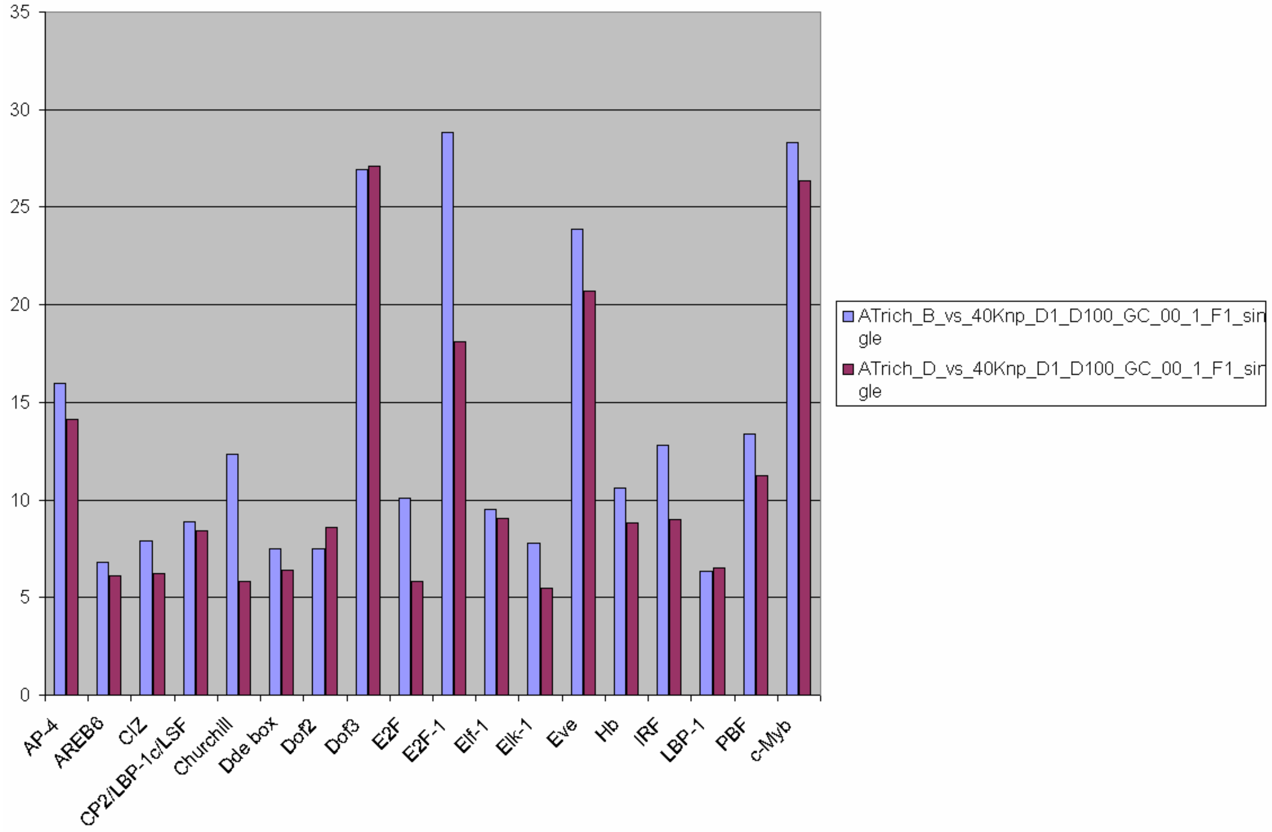

b/ Comparison of types B and D downstream regions

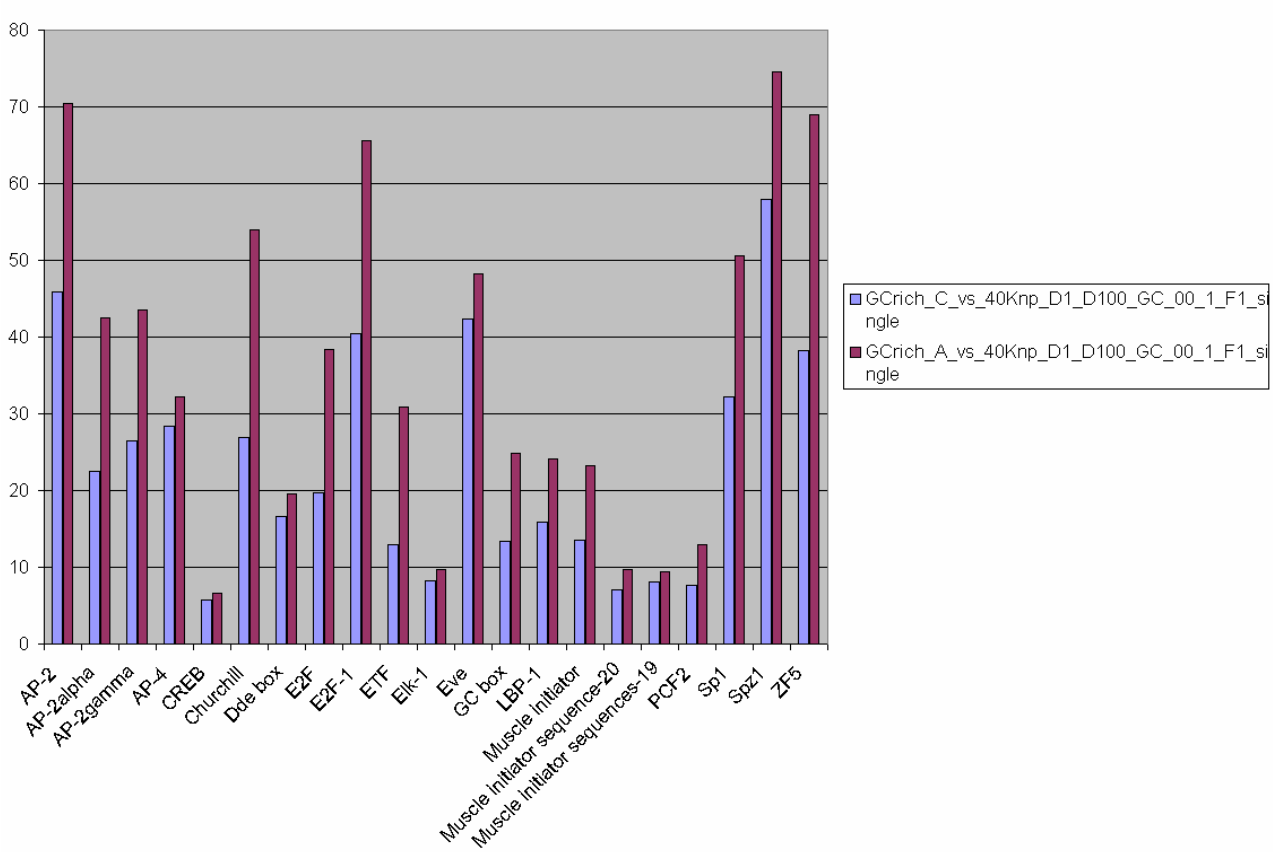

c/ Comparison of types A and C downstream regions

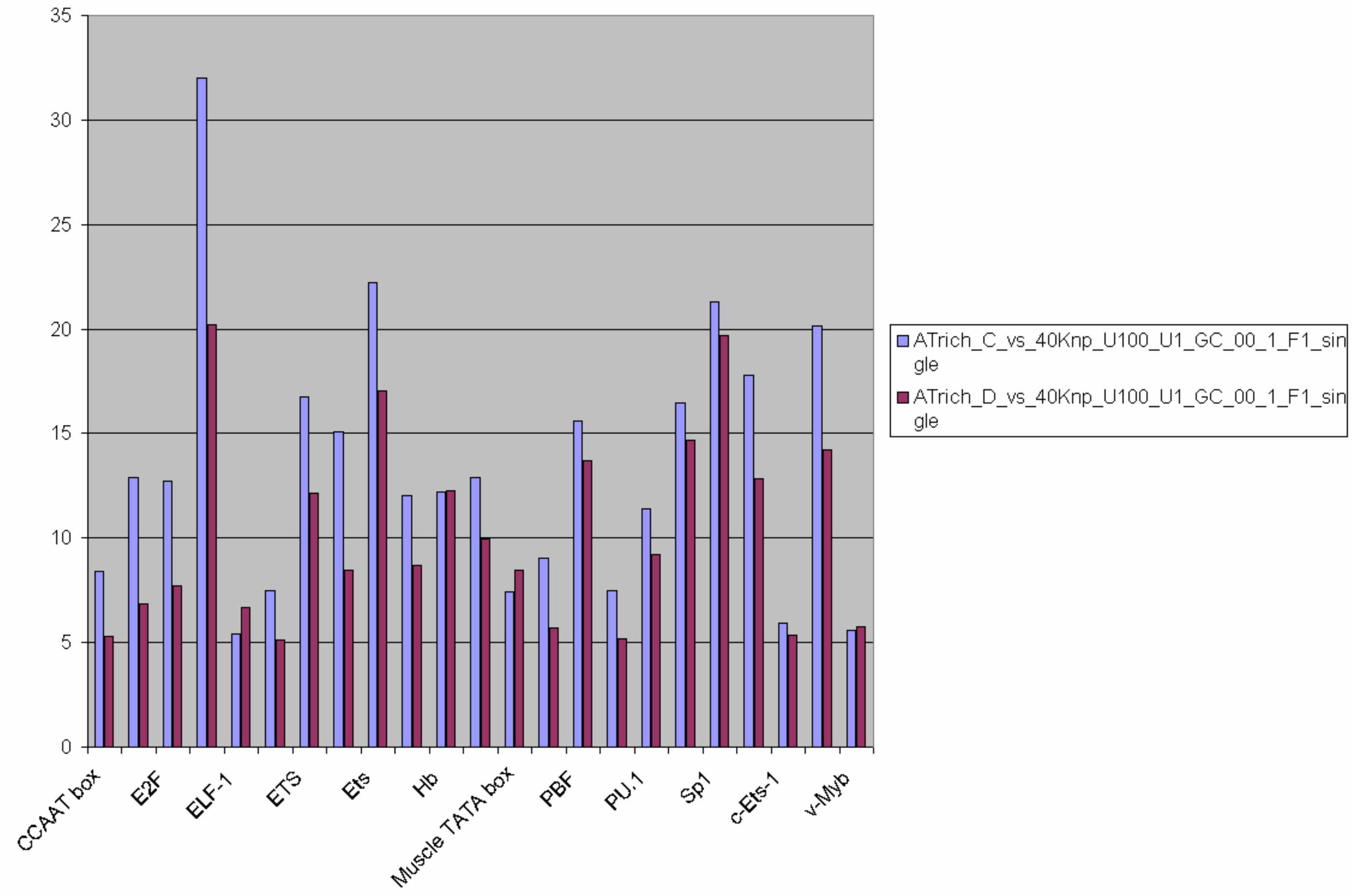

d/ Comparison of types C and D upstream regions
