# Supplementary material for: Mice and Men: Their Promoter Properties
Source: PLoS Genet. 2006 Apr 28;2(4):e54. doi: 10.1371/journal.pgen.0020054 (PMC1449896; doi:10.1371/journal.pgen.0020054)
Supplement: Table S1 — Comparison is carried out against a background of random mouse sequences. Ranking is based on ORI value. The higher the ORI, the higher the rank. We present results for the four TSS types (A, B, C, and D). For each PE we give the strand where it is found (+1 or −1), name of TFBS, ORI value, percentage of promoters in the target set that contain the PE, percentage of sequences in the background set that contain the PE, probability of finding the PE in the target set (given as one prediction per nucleotide), probability of finding the PE in the background set (given as one prediction per nucleotide), and Bonferroni corrected p-value. A plus sign added after the ORI value indicates that the PE is enriched in a statistically significant manner at the level 0.05. Almost all top-ranked elements appear to be statistically significantly enriched in the target sets. (329 KB PDF) [file pgen.0020054.st001.pdf]

TSS GROUP A (UPSTREAM)

Up to 150 top ranked promoter elements that appear with frequency >= 10% in the target set

TFBS pattern	ORI	% TAR	% BCG	Prob TARGE	Prob BACKG	# TAR	# BCG	TOT TAR	TOT BCG	p_value
============	=======	======	======	=========	=========	=====	=====	=======	=======	=========
-1 KROX	4014.7197+	15.71	0.26	1.92E-03	2.90E-05	5379	106	34245	41000	0.00E+00
+1 KROX	2307.6721+	11.84	0.25	1.42E-03	2.88E-05	4056	104	34245	41000	0.00E+00
-1 Hairy	1020.6855+	12.12	0.39	1.35E-03	4.10E-05	4151	160	34245	41000	0.00E+00
+1 E2F	365.8805+	51.48	3.24	1.01E-02	4.38E-04	17631	1330	34245	41000	0.00E+00
+1 Churchill	315.6059+	56.11	4.07	1.03E-02	4.49E-04	19216	1670	34245	41000	0.00E+00
+1 ETF	260.6690+	40.27	2.91	7.39E-03	3.92E-04	13790	1194	34245	41000	0.00E+00
-1 Churchill	228.2272+	50.43	4.17	8.74E-03	4.63E-04	17271	1708	34245	41000	0.00E+00
-1 E2F	222.2317+	42.97	3.33	7.76E-03	4.50E-04	14714	1367	34245	41000	0.00E+00
+1 ZF5	212.8799+	74.38	8.04	2.45E-02	1.06E-03	25471	3298	34245	41000	0.00E+00
+1 MAZR	194.6093+	10.84	0.64	1.51E-03	1.32E-04	3711	262	34245	41000	0.00E+00
-1 ETF	173.3759+	32.51	2.82	5.63E-03	3.74E-04	11133	1157	34245	41000	0.00E+00
-1 ZF5	171.0610+	71.11	8.3	2.19E-02	1.10E-03	24351	3403	34245	41000	0.00E+00
+1 GC box	149.7130+	49.75	4.58	9.93E-03	7.20E-04	17037	1879	34245	41000	0.00E+00
+1 EGR	133.9548+	22.21	1.9	2.94E-03	2.56E-04	7607	779	34245	41000	0.00E+00
-1 CREB	133.2002+	16.73	1.81	4.33E-03	3.00E-04	5728	743	34245	41000	0.00E+00
+1 Sp-1	128.6165+	65.01	6.5	2.03E-02	1.58E-03	22264	2663	34245	41000	0.00E+00
+1 AHRHIF	126.9181+	11.32	1.03	1.29E-03	1.12E-04	3877	422	34245	41000	0.00E+00
+1 CREB	118.7973+	16.3	1.9	4.31E-03	3.10E-04	5583	781	34245	41000	0.00E+00
-1 Sp-1	113.8594+	63.84	6.49	1.85E-02	1.60E-03	21861	2659	34245	41000	0.00E+00
-1 GC box	86.4010+	40.44	4.85	7.81E-03	7.53E-04	13847	1989	34245	41000	0.00E+00
+1 MAZ	83.6542+	22	2.46	3.07E-03	3.29E-04	7533	1008	34245	41000	0.00E+00
-1 AP-2alpha	83.6344+	45.73	5.8	6.67E-03	6.28E-04	15661	2380	34245	41000	0.00E+00
+1 AP-2alpha	76.7122+	44.32	5.81	6.24E-03	6.21E-04	15179	2381	34245	41000	0.00E+00
+1 E2F-1	75.7355+	70.85	10.93	1.59E-02	1.36E-03	24261	4483	34245	41000	0.00E+00
-1 MAZ	71.8710+	18.83	2.3	2.75E-03	3.14E-04	6450	941	34245	41000	0.00E+00
-1 E2F-1	63.3131+	66.78	10.91	1.40E-02	1.36E-03	22870	4474	34245	41000	0.00E+00
+1 Sp1	61.8822+	62.74	11.46	2.08E-02	1.84E-03	21486	4697	34245	41000	0.00E+00
-1 EGR	61.0325+	16.47	2.17	2.26E-03	2.82E-04	5639	889	34245	41000	0.00E+00
+1 Elk-1	55.5817+	19.89	3	2.91E-03	3.47E-04	6810	1230	34245	41000	0.00E+00
-1 PCF2	54.0154+	16.1	2.31	1.83E-03	2.36E-04	5514	949	34245	41000	0.00E+00

-1 AP-2	53.7797+	71.69	13.09	2.10E-02	2.14E-03	24549	5367	34245	41000	0.00E+00
+1 AP-2	49.5005+	70.06	13.05	1.98E-02	2.15E-03	23991	5350	34245	41000	0.00E+00
-1 AP-2gamma	47.3290+	46.9	7.77	6.63E-03	8.45E-04	16062	3185	34245	41000	0.00E+00
+1 AP-2gamma	44.6164+	46.11	7.81	6.39E-03	8.46E-04	15791	3202	34245	41000	0.00E+00
-1 Elk-1	44.1571+	18.25	3.03	2.54E-03	3.47E-04	6250	1242	34245	41000	0.00E+00
+1 PCF2	38.1039+	13.54	2.31	1.54E-03	2.37E-04	4637	947	34245	41000	0.00E+00
-1 Sp1	37.7827+	53.67	11.93	1.62E-02	1.93E-03	18379	4893	34245	41000	0.00E+00
-1 Muscle initiator	36.6129+	20.5	3.55	2.43E-03	3.84E-04	7019	1455	34245	41000	0.00E+00
+1 Sp3	28.6658+	10.89	2.05	1.20E-03	2.22E-04	3729	839	34245	41000	0.00E+00
+1 LBP-1	24.3291+	14.65	2.99	1.65E-03	3.33E-04	5018	1224	34245	41000	0.00E+00
-1 c-Myc:Max	22.3925+	10.07	2.22	1.24E-03	2.51E-04	3449	910	34245	41000	0.00E+00
-1 LBP-1	20.7995+	13.83	3.08	1.55E-03	3.34E-04	4735	1261	34245	41000	0.00E+00
+1 Spz1	18.5734+	74.71	25.13	2.66E-02	4.26E-03	25583	10304	34245	41000	0.00E+00
+1 Muscle initiator	17.9697+	15.47	3.79	1.79E-03	4.07E-04	5299	1553	34245	41000	0.00E+00
+1 VDR	14.8226+	30	8.8	5.58E-03	1.29E-03	10273	3606	34245	41000	0.00E+00
+1 ADR1	13.6571+	31.43	9.15	5.95E-03	1.50E-03	10763	3750	34245	41000	0.00E+00
+1 Alfin1	13.1533+	11.9	3.3	1.39E-03	3.81E-04	4075	1355	34245	41000	0.00E+00
-1 Dde box	13.1278+	18.52	5.34	2.13E-03	5.62E-04	6343	2189	34245	41000	0.00E+00
-1 Spz1	11.9676+	65.42	25.81	2.09E-02	4.42E-03	22404	10583	34245	41000	0.00E+00
+1 Zic3	11.8691+	33.48	10.45	4.52E-03	1.22E-03	11466	4283	34245	41000	0.00E+00
-1 VDR	10.5111+	25.42	8.9	4.81E-03	1.31E-03	8705	3647	34245	41000	0.00E+00
-1 USF2	9.8459+	12.31	4.02	1.36E-03	4.22E-04	4214	1649	34245	41000	0.00E+00
+1 Dde box	9.5729+	15.73	5.19	1.75E-03	5.53E-04	5388	2127	34245	41000	0.00E+00
+1 USF2	9.1175+	11.91	4.07	1.32E-03	4.25E-04	4078	1667	34245	41000	0.00E+00
-1 Eve	8.9965+	44.18	16.07	6.02E-03	1.84E-03	15130	6587	34245	41000	0.00E+00
+1 Zic1	8.8254+	42.79	15.94	6.27E-03	1.91E-03	14653	6534	34245	41000	0.00E+00
-1 ADR1	8.4784+	26.21	9.74	5.02E-03	1.59E-03	8977	3992	34245	41000	0.00E+00
+1 TFII-I	7.9267+	15.9	5.83	2.03E-03	6.97E-04	5444	2390	34245	41000	0.00E+00
+1 Eve	7.7492+	41.5	15.99	5.49E-03	1.84E-03	14213	6554	34245	41000	0.00E+00
-1 Zic3	7.0795+	27.55	10.95	3.63E-03	1.29E-03	9435	4489	34245	41000	0.00E+00
+1 c-Ets-1(p54)	6.7418+	19.23	8.94	3.39E-03	1.08E-03	6586	3665	34245	41000	0.00E+00
+1 CP2/LBP-1c/LSF	6.2566+	14.3	5.84	1.57E-03	6.15E-04	4897	2393	34245	41000	0.00E+00
-1 TFII-I	5.9889+	13.13	5.62	1.70E-03	6.63E-04	4495	2304	34245	41000	3.65E-278
-1 c-Ets-1(p54)	5.8507+	18.23	9	3.12E-03	1.08E-03	6242	3688	34245	41000	7.81E-301
-1 Zic1	5.0687+	34.22	16.05	4.65E-03	1.96E-03	11717	6580	34245	41000	0.00E+00
-1 CP2/LBP-1c/LSF	4.5748+	11.91	5.65	1.31E-03	6.01E-04	4077	2317	34245	41000	3.12E-203

+1 MZF1	4.4604+	30.38	14.63	5.01E-03	2.33E-03	10405	5998	34245	41000	0.00E+00
+1 LF-A1	4.3382+	14.23	6.94	1.54E-03	7.29E-04	4873	2846	34245	41000	4.20E-233
-1 Ets	4.0902+	21.47	10.94	2.62E-03	1.26E-03	7351	4484	34245	41000	0.00E+00
-1 MZF1	4.0184+	28.74	14.63	4.94E-03	2.42E-03	9842	6000	34245	41000	0.00E+00
+1 Zic2	3.9227+	13.83	7.02	1.55E-03	7.80E-04	4735	2878	34245	41000	1.48E-205
+1 PU.1	3.8378+	10.57	5.51	1.18E-03	5.88E-04	3619	2259	34245	41000	6.33E-143
+1 AP-4	3.8249+	20.81	10.24	3.71E-03	1.97E-03	7127	4199	34245	41000	0.00E+00
-1 AP-4	3.7018+	20.13	10.1	3.64E-03	1.96E-03	6894	4139	34245	41000	0.00E+00
+1 CF1 / USP	3.4177+	10.27	5.63	1.12E-03	6.00E-04	3518	2308	34245	41000	3.48E-121
-1 LF-A1	3.2628+	12.4	6.95	1.34E-03	7.31E-04	4246	2850	34245	41000	1.35E-139
+1 GAGA factor	3.2231+	35.26	18.27	5.56E-03	3.33E-03	12074	7489	34245	41000	0.00E+00
-1 FACB	3.1366+	14.17	7.97	1.76E-03	9.95E-04	4851	3268	34245	41000	2.52E-160
-1 NF-1	3.1297+	18.46	10.7	2.07E-03	1.14E-03	6323	4388	34245	41000	1.22E-198
+1 Ets	3.1014+	18.77	10.86	2.23E-03	1.24E-03	6427	4454	34245	41000	2.33E-203
+1 FACB	2.9777+	13.77	8.03	1.74E-03	1.00E-03	4716	3293	34245	41000	4.27E-139
+1 Knox3	2.8721+	16.94	10.17	1.88E-03	1.09E-03	5801	4168	34245	41000	1.86E-160
-1 GAGA factor	2.8412+	32.86	18.67	5.19E-03	3.22E-03	11253	7655	34245	41000	0.00E+00
+1 p300	2.8239+	18.55	11.23	2.10E-03	1.23E-03	6352	4606	34245	41000	6.60E-173
+1 NF-1	2.7171+	17.3	10.7	1.92E-03	1.14E-03	5924	4385	34245	41000	3.89E-148
-1 Knox3	2.5263+	16.27	10.31	1.77E-03	1.11E-03	5571	4229	34245	41000	1.73E-125
+1 p53 decamer	2.2345+	35.76	24.56	4.61E-03	3.00E-03	12246	10068	34245	41000	1.11E-242
-1 p53 decamer	2.2124+	35.77	24.51	4.57E-03	3.01E-03	12251	10050	34245	41000	2.57E-245
-1 RAV1	2.1250+	22.92	15.96	2.62E-03	1.77E-03	7848	6545	34245	41000	2.04E-125
-1 LIM1	1.7239+	37.7	29.29	5.38E-03	4.02E-03	12912	12010	34245	41000	1.81E-128
-1 p300	1.6172+	14.86	11.71	1.62E-03	1.27E-03	5088	4801	34245	41000	4.91E-34
-1 COUP	1.5727+	14.91	11.92	1.64E-03	1.31E-03	5107	4887	34245	41000	2.19E-30
-1 RAR	1.5727+	14.91	11.92	1.64E-03	1.31E-03	5107	4887	34245	41000	2.19E-30
+1 RAV1	1.5476+	19.83	16.02	2.23E-03	1.78E-03	6792	6569	34245	41000	3.31E-39

TSS GROUP A (DOWNSTREAM)

Up to 150 top ranked promoter elements that appear with frequency >= 10% in the target set

TFBS pattern	ORI	% TAR	% BCG	Prob TARGE	Prob BACKG	# TAR	# BCG	TOT TAR	TOT BCG	p_value
============	======	======	======	=========	=========	=====	=====	=======	=======	=========
+1 Adf-1	10910.6022+	13.75	0.14	2.99E-03	2.66E-05	4708	58	34245	41000	0.00E+00

-1 Adf-1	9671.4262+	12.66	0.15	3.01E-03	2.56E-05	4337	63	34245	41000	0.00E+00
+1 Churchill	260.7750+	53.97	4.06	8.72E-03	4.45E-04	18482	1663	34245	41000	0.00E+00
+1 E2F	159.7316+	38.41	3.44	6.80E-03	4.76E-04	13155	1409	34245	41000	0.00E+00
-1 Churchill	152.3703+	44.49	4.17	6.66E-03	4.67E-04	15234	1708	34245	41000	0.00E+00
+1 ETF	148.9839+	30.82	2.9	5.43E-03	3.87E-04	10554	1190	34245	41000	0.00E+00
+1 ZF5	132.8648+	68.95	8.61	1.80E-02	1.09E-03	23611	3530	34245	41000	0.00E+00
-1 ZF5	100.8378+	61.96	8.37	1.49E-02	1.09E-03	21219	3430	34245	41000	0.00E+00
-1 E2F	81.0920+	28.33	3.38	4.49E-03	4.64E-04	9703	1385	34245	41000	0.00E+00
+1 E2F-1	77.7542+	72.81	11.17	1.68E-02	1.41E-03	24935	4579	34245	41000	0.00E+00
+1 LBP-1	73.0613+	24.15	3.05	3.01E-03	3.26E-04	8270	1250	34245	41000	0.00E+00
-1 AP-2alpha	68.2823+	43.07	6.03	6.18E-03	6.47E-04	14749	2471	34245	41000	0.00E+00
+1 AP-2alpha	64.8571+	42.48	6	5.90E-03	6.43E-04	14548	2462	34245	41000	0.00E+00
-1 ETF	60.2040+	19.07	2.7	3.07E-03	3.60E-04	6529	1107	34245	41000	0.00E+00
-1 E2F-1	57.4390+	65.66	10.94	1.31E-02	1.37E-03	22484	4484	34245	41000	0.00E+00
-1 AP-2	50.6707+	70.38	13.05	2.01E-02	2.14E-03	24103	5352	34245	41000	0.00E+00
-1 LBP-1	47.1801+	20.21	3.14	2.53E-03	3.44E-04	6920	1289	34245	41000	0.00E+00
-1 Muscle initiator	45.7502+	23.26	3.61	2.76E-03	3.89E-04	7965	1479	34245	41000	0.00E+00
+1 AP-2gamma	41.5720+	46.39	8.07	6.33E-03	8.75E-04	15885	3308	34245	41000	0.00E+00
+1 AP-2	41.3918+	65.06	13.1	1.76E-02	2.12E-03	22279	5369	34245	41000	0.00E+00
-1 AP-2gamma	39.4382+	43.54	7.83	6.00E-03	8.45E-04	14910	3211	34245	41000	0.00E+00
-1 PCF2	33.9787+	12.93	2.28	1.39E-03	2.33E-04	4427	933	34245	41000	0.00E+00
+1 EGR	27.0683+	11.25	2.02	1.32E-03	2.71E-04	3852	829	34245	41000	0.00E+00
+1 GC box	25.4350+	24.84	4.58	3.43E-03	7.32E-04	8505	1876	34245	41000	0.00E+00
+1 Sp1	23.2821+	50.53	11.74	1.03E-02	1.91E-03	17304	4814	34245	41000	0.00E+00
-1 Dde box	16.2390+	19.63	5.06	2.25E-03	5.37E-04	6721	2074	34245	41000	0.00E+00
+1 Sp-1	15.6513+	28.15	6.45	5.73E-03	1.60E-03	9641	2645	34245	41000	0.00E+00
+1 Spz1	14.5387+	74.5	25.19	2.12E-02	4.32E-03	25511	10327	34245	41000	0.00E+00
+1 Muscle initiator	11.9368+	12.51	3.69	1.39E-03	3.96E-04	4285	1511	34245	41000	0.00E+00
-1 Sp-1	11.6156+	25.88	6.44	4.66E-03	1.61E-03	8861	2641	34245	41000	0.00E+00
-1 Eve	11.1058+	48.26	16.11	6.83E-03	1.84E-03	16527	6607	34245	41000	0.00E+00
-1 Sp1	10.0638+	35.11	11.7	6.40E-03	1.91E-03	12022	4797	34245	41000	0.00E+00
-1 AP-4	9.6254+	32.24	10.2	5.97E-03	1.96E-03	11042	4181	34245	41000	0.00E+00
+1 Zic3	9.3166+	30.35	10.32	3.84E-03	1.21E-03	10394	4230	34245	41000	0.00E+00
-1 GC box	9.2345+	15.63	4.73	2.09E-03	7.47E-04	5353	1938	34245	41000	0.00E+00
+1 VDR	9.2097+	27.13	9	4.02E-03	1.32E-03	9292	3688	34245	41000	0.00E+00
+1 Eve	8.9909+	43.1	15.62	5.84E-03	1.79E-03	14760	6404	34245	41000	0.00E+00

+1 AP-4	8.6336+	30.77	10.17	5.61E-03	1.97E-03	10537	4171	34245	41000	0.00E+00
+1 Alfin1	8.0970+	10.5	3.71	1.21E-03	4.23E-04	3597	1523	34245	41000	3.18E-297
+1 CP2/LBP-1c/LSF	7.9882+	16.17	5.85	1.80E-03	6.22E-04	5536	2397	34245	41000	0.00E+00
+1 Zic1	7.5085+	40.1	15.72	5.52E-03	1.88E-03	13731	6446	34245	41000	0.00E+00
+1 Dde box	6.8462+	13.36	5.25	1.47E-03	5.48E-04	4575	2153	34245	41000	0.00E+00
+1 LF-A1	6.7865+	18	7.09	1.99E-03	7.44E-04	6163	2907	34245	41000	0.00E+00
-1 Spz1	6.1335+	54.21	25.15	1.23E-02	4.31E-03	18564	10312	34245	41000	0.00E+00
+1 ADR1	5.4788+	23.06	9.43	3.56E-03	1.59E-03	7897	3868	34245	41000	0.00E+00
+1 TFII-I	5.2088+	13.26	5.65	1.49E-03	6.73E-04	4542	2316	34245	41000	4.48E-284
-1 CP2/LBP-1c/LSF	5.0003+	12.55	5.74	1.38E-03	6.05E-04	4298	2352	34245	41000	3.22E-233
+1 Zic2	4.5488+	14.93	7.16	1.72E-03	7.86E-04	5112	2934	34245	41000	1.03E-255
-1 LF-A1	4.4801+	14.78	7.14	1.62E-03	7.50E-04	5062	2927	34245	41000	8.84E-249
+1 GAGA factor	4.3748+	40.4	18.48	6.43E-03	3.21E-03	13836	7575	34245	41000	0.00E+00
+1 CF1 / USP	3.9783+	11.37	5.78	1.24E-03	6.12E-04	3892	2370	34245	41000	1.09E-164
-1 Zic3	3.8978+	20.49	10.49	2.47E-03	1.24E-03	7017	4300	34245	41000	2.017e-316
-1 VDR	3.4373+	17.01	9.08	2.41E-03	1.32E-03	5826	3722	34245	41000	3.71E-229
+1 MZF1	3.2912+	27.66	14.52	4.01E-03	2.32E-03	9472	5954	34245	41000	0.00E+00
+1 FACB	3.2126+	14.33	8.04	1.80E-03	9.98E-04	4908	3295	34245	41000	4.08E-164
-1 FACB	3.0156+	14.05	8.1	1.75E-03	1.01E-03	4813	3322	34245	41000	1.25E-147
-1 LIM1	2.9966+	47.53	28.98	7.26E-03	3.98E-03	16276	11883	34245	41000	0.00E+00
-1 Zic1	2.8129+	26.15	15.97	3.33E-03	1.94E-03	8955	6546	34245	41000	1.46E-255
-1 ADR1	2.6332+	16.02	9.41	2.41E-03	1.56E-03	5486	3857	34245	41000	1.24E-161
-1 P	2.3573+	10.46	6.71	1.13E-03	7.44E-04	3582	2753	34245	41000	1.71E-72
+1 p300	2.1817+	16.25	11.16	1.81E-03	1.21E-03	5564	4577	34245	41000	1.35E-88
-1 GAGA factor	2.1716+	29.74	18.55	4.52E-03	3.34E-03	10185	7605	34245	41000	7.26E-280
-1 RAV1	2.0579+	22.21	15.7	2.53E-03	1.74E-03	7606	6439	34245	41000	9.63E-112
+1 p53 decamer	2.0459+	34.7	24.57	4.36E-03	3.01E-03	11883	10074	34245	41000	4.86E-200
+1 AP-2rep	2.0103+	42.58	30.97	5.52E-03	3.78E-03	14582	12698	34245	41000	1.66E-235
-1 p53 decamer	1.9398+	33.57	24.44	4.22E-03	2.99E-03	11495	10021	34245	41000	3.84E-164
-1 COUP	1.8113+	16.01	11.89	1.77E-03	1.32E-03	5483	4876	34245	41000	9.79E-57
-1 RAR	1.8113+	16.01	11.89	1.77E-03	1.32E-03	5483	4876	34245	41000	9.79E-57
-1 MZF1	1.7264+	19.77	14.24	2.85E-03	2.29E-03	6771	5840	34245	41000	1.41E-87
+1 RFX	1.7227+	15.43	11.84	1.69E-03	1.28E-03	5285	4856	34245	41000	1.28E-43

TSS GROUP B (UPSTREAM)

Up to 150 top ranked promoter elements that appear with frequency >= 10% in the target set

TFBS pattern	ORI	% TAR	% BCG	Prob TARGE	Prob BACKG	# TAR	# BCG	TOT TAR	TOT BCG	p_value
============	======	======	======	==========	==========	=====	=====	=======	=======	=========
+1 E2F	88.1874+	26.32	3.24	4.76E-03	4.38E-04	379	1330	1440	41000	2.21E-201
+1 ETF	82.7492+	24.38	2.91	3.88E-03	3.92E-04	351	1194	1440	41000	9.47E-189
+1 GC box	78.4738+	36.32	4.58	7.13E-03	7.20E-04	523	1879	1440	41000	1.87E-285
+1 EGR	76.5309+	15.69	1.9	2.38E-03	2.56E-04	226	779	1440	41000	2.77E-116
+1 MAZ	68.7659+	18.82	2.46	2.95E-03	3.29E-04	271	1008	1440	41000	2.68E-134
+1 Sp-1	67.4470+	47.01	6.5	1.47E-02	1.58E-03	677	2663	1440	41000	0.00E+00
+1 Churchill	61.1214+	27.92	4.07	4.00E-03	4.49E-04	402	1670	1440	41000	9.09E-192
+1 CAC-binding protein	54.7367+	11.88	1.8	1.93E-03	2.33E-04	171	738	1440	41000	1.21E-73
-1 CREB	53.8446+	11.25	1.81	2.60E-03	3.00E-04	162	743	1440	41000	2.91E-66
-1 Sp-1	53.8425+	45.83	6.49	1.22E-02	1.60E-03	660	2659	1440	41000	0.00E+00
+1 ZF5	49.5788+	44.86	8.04	9.45E-03	1.06E-03	646	3298	1440	41000	2.29E-286
+1 CREB	45.3181+	10.07	1.9	2.66E-03	3.10E-04	145	781	1440	41000	2.94E-51
-1 ETF	41.4587+	17.57	2.82	2.49E-03	3.74E-04	253	1157	1440	41000	3.98E-107
-1 E2F	39.6705+	18.89	3.33	3.15E-03	4.50E-04	272	1367	1440	41000	2.59E-107
-1 Churchill	37.1160+	23.61	4.17	3.04E-03	4.63E-04	340	1708	1440	41000	2.66E-137
+1 Elk-1	35.5745+	16.39	3	2.26E-03	3.47E-04	236	1230	1440	41000	4.05E-89
+1 Sp1	34.9204+	51.81	11.46	1.42E-02	1.84E-03	746	4697	1440	41000	3.12E-288
-1 ZF5	34.4765+	40.14	8.3	7.83E-03	1.10E-03	578	3403	1440	41000	1.71E-221
+1 Sp3	30.3551+	11.04	2.05	1.25E-03	2.22E-04	159	839	1440	41000	1.24E-57
-1 EGR	28.8156+	10.69	2.17	1.65E-03	2.82E-04	154	889	1440	41000	3.24E-51
-1 GC box	28.6393+	24.79	4.85	4.22E-03	7.53E-04	357	1989	1440	41000	1.33E-132
+1 E2F-1	26.9589+	45.49	10.93	8.81E-03	1.36E-03	655	4483	1440	41000	1.17E-224
-1 MAZ	26.0793+	11.81	2.3	1.59E-03	3.14E-04	170	941	1440	41000	2.91E-59
-1 Muscle initiator	23.6173+	16.67	3.55	1.93E-03	3.84E-04	240	1455	1440	41000	6.84E-79
-1 Elk-1	21.8149+	12.36	3.03	1.85E-03	3.47E-04	178	1242	1440	41000	4.02E-49
+1 Alfin1	21.7230+	13.82	3.3	1.98E-03	3.81E-04	199	1355	1440	41000	5.43E-57
-1 E2F-1	18.6089+	39.72	10.91	6.94E-03	1.36E-03	572	4474	1440	41000	5.64E-164
+1 AP-2alpha	15.6778+	21.94	5.81	2.58E-03	6.21E-04	316	2381	1440	41000	8.09E-85
-1 AP-2alpha	14.9949+	21.46	5.8	2.55E-03	6.28E-04	309	2380	1440	41000	1.43E-80
-1 AP-2	14.2907+	43.75	13.09	9.14E-03	2.14E-03	630	5367	1440	41000	1.25E-167
+1 VDR	13.8281+	27.64	8.8	5.65E-03	1.29E-03	398	3606	1440	41000	1.27E-87
+1 TFII-I	13.5629+	18.89	5.83	2.92E-03	6.97E-04	272	2390	1440	41000	6.66E-59

+1 ADR1	13.4548+	27.92	9.15	6.60E-03	1.50E-03	402	3750	1440	41000	4.08E-85
-1 Sp1	13.3930+	35.97	11.93	8.56E-03	1.93E-03	518	4893	1440	41000	6.32E-114
+1 AP-2	12.5242+	41.88	13.05	8.39E-03	2.15E-03	603	5350	1440	41000	1.93E-150
-1 AP-2gamma	12.2772+	26.88	7.77	3.00E-03	8.45E-04	387	3185	1440	41000	3.24E-96
+1 AP-2gamma	11.8077+	25.35	7.81	3.08E-03	8.46E-04	365	3202	1440	41000	1.05E-82
+1 Spz1	11.3353+	62.85	25.13	1.93E-02	4.26E-03	905	10304	1440	41000	1.28E-188
+1 Zic3	10.5244+	30.69	10.45	4.37E-03	1.22E-03	442	4283	1440	41000	2.74E-90
+1 Muscle initiator	9.0432+	10.97	3.79	1.27E-03	4.07E-04	158	1553	1440	41000	3.84E-27
-1 Dde box	7.2563+	13.75	5.34	1.58E-03	5.62E-04	198	2189	1440	41000	1.47E-28
+1 Zic1	7.0889+	37.64	15.94	5.73E-03	1.91E-03	542	6534	1440	41000	3.45E-81
-1 VDR	6.0083+	18.61	8.9	3.76E-03	1.31E-03	268	3647	1440	41000	3.57E-26
-1 TFII-I	5.9799+	12.64	5.62	1.76E-03	6.63E-04	182	2304	1440	41000	1.11E-19
+1 PU.1	5.9676+	13.26	5.51	1.46E-03	5.88E-04	191	2259	1440	41000	4.63E-24
-1 Spz1	5.9131+	51.18	25.81	1.32E-02	4.42E-03	737	10583	1440	41000	7.73E-87
-1 Eve	5.4034+	35.49	16.07	4.50E-03	1.84E-03	511	6587	1440	41000	9.03E-66
+1 CP2/LBP-1c/LSF	5.2557+	12.57	5.84	1.50E-03	6.15E-04	181	2393	1440	41000	1.14E-17
+1 MZF1	5.1802+	30.28	14.63	5.84E-03	2.33E-03	436	5998	1440	41000	2.38E-46
+1 c-Ets-1(p54)	5.0900+	16.53	8.94	2.98E-03	1.08E-03	238	3665	1440	41000	3.71E-16
-1 ADR1	4.9670+	18.82	9.74	4.10E-03	1.59E-03	271	3992	1440	41000	1.42E-21
-1 Ets	4.9005+	23.47	10.94	2.87E-03	1.26E-03	338	4484	1440	41000	9.72E-37
-1 c-Ets-1(p54)	4.4081+	16.25	9	2.64E-03	1.08E-03	234	3688	1440	41000	1.07E-14
-1 Zic3	4.3366+	21.67	10.95	2.83E-03	1.29E-03	312	4489	1440	41000	2.63E-27
+1 Zic2	4.3335+	13.4	7.02	1.77E-03	7.80E-04	193	2878	1440	41000	8.35E-14
-1 CP2/LBP-1c/LSF	4.0751+	11.46	5.65	1.21E-03	6.01E-04	165	2317	1440	41000	1.44E-13
-1 AP-4	3.9309+	19.38	10.1	4.01E-03	1.96E-03	279	4139	1440	41000	6.53E-22
+1 Eve	3.9023+	30.97	15.99	3.70E-03	1.84E-03	446	6554	1440	41000	1.75E-40
+1 GAGA factor	3.6679+	33.82	18.27	6.60E-03	3.33E-03	487	7489	1440	41000	3.66E-40
-1 MZF1	3.5704+	26.46	14.63	4.77E-03	2.42E-03	381	6000	1440	41000	3.96E-27
-1 Zic1	3.5624+	28.61	16.05	3.91E-03	1.96E-03	412	6580	1440	41000	9.55E-29
-1 NF-1	3.4708+	19.24	10.7	2.20E-03	1.14E-03	277	4388	1440	41000	8.14E-18
+1 AP-4	3.4638+	18.26	10.24	3.83E-03	1.97E-03	263	4199	1440	41000	2.80E-16
-1 PU.1	3.2984+	10	5.61	1.11E-03	6.01E-04	144	2300	1440	41000	1.23E-07
+1 NF-1	3.2388+	18.4	10.7	2.15E-03	1.14E-03	265	4385	1440	41000	1.57E-14
-1 ETS	3.2039+	14.58	8.15	1.67E-03	9.31E-04	210	3341	1440	41000	1.86E-12
-1 P	3.1002+	11.74	6.81	1.37E-03	7.60E-04	169	2793	1440	41000	3.02E-08
+1 Ets	3.0047+	18.26	10.86	2.22E-03	1.24E-03	263	4454	1440	41000	3.28E-13

-1 FACB	2.8859+	13.4	7.97	1.71E-03	9.95E-04	193	3268	1440	41000	6.82E-09
+1 p300	2.8693+	18.89	11.23	2.09E-03	1.23E-03	272	4606	1440	41000	8.53E-14
-1 Zic2	2.6427+	12.43	7.5	1.35E-03	8.44E-04	179	3077	1440	41000	1.43E-07
-1 LIM1	2.5984+	44.93	29.29	6.81E-03	4.02E-03	647	12010	1440	41000	8.74E-32
+1 LF-A1	2.5303+	11.11	6.94	1.15E-03	7.29E-04	160	2846	1440	41000	1.48E-05
-1 Knox3	2.4816+	16.25	10.31	1.74E-03	1.11E-03	234	4229	1440	41000	1.24E-08
+1 Lyf-1	2.4473+	14.38	9.27	1.56E-03	9.86E-04	207	3799	1440	41000	8.70E-07
-1 GAGA factor	2.4423+	29.17	18.67	5.03E-03	3.22E-03	420	7655	1440	41000	3.21E-18
-1 LF-A1	2.4117+	10.69	6.95	1.15E-03	7.31E-04	154	2850	1440	41000	2.97E-04
-1 STAT6	2.3046+	28.68	19.18	3.60E-03	2.33E-03	413	7865	1440	41000	1.47E-14
+1 ETS	2.2908+	12.08	8.06	1.40E-03	9.14E-04	174	3304	1440	41000	2.23E-04
-1 STAT3	2.2794+	48.47	33.68	6.89E-03	4.35E-03	698	13809	1440	41000	6.81E-27
+1 Knox3	2.2075+	15.07	10.17	1.63E-03	1.09E-03	217	4168	1440	41000	1.19E-05
+1 RFX	2.1644+	16.88	11.79	1.90E-03	1.26E-03	243	4833	1440	41000	2.31E-05
+1 PPAR	2.0879+	13.12	9.31	1.69E-03	1.14E-03	189	3816	1440	41000	3.07E-03
-1 COUP	2.0427+	17.01	11.92	1.87E-03	1.31E-03	245	4887	1440	41000	2.53E-05
-1 RAR	2.0427+	17.01	11.92	1.87E-03	1.31E-03	245	4887	1440	41000	2.53E-05
+1 p53 decamer	1.9694+	34.58	24.56	4.20E-03	3.00E-03	498	10068	1440	41000	6.26E-14
+1 COUP	1.9275+	15.9	11.73	1.83E-03	1.28E-03	229	4811	1440	41000	3.46E-03
+1 RAR	1.9275+	15.9	11.73	1.83E-03	1.28E-03	229	4811	1440	41000	3.46E-03
-1 p53 decamer	1.9143+	32.85	24.51	4.31E-03	3.01E-03	473	10050	1440	41000	2.53E-09
-1 PPAR	1.8234+	13.06	9.58	1.56E-03	1.17E-03	188	3927	1440	41000	2.34E-02
+1 FACB	1.7691	10.14	8.03	1.40E-03	1.00E-03	146	3293	1440	41000	1.00E+00
+1 Pax	1.7425	11.25	8.65	1.23E-03	9.18E-04	162	3545	1440	41000	7.74E-01
-1 MYBAS1	1.7112+	15.83	12.16	1.72E-03	1.31E-03	228	4985	1440	41000	4.73E-02
+1 AP-2rep	1.6929+	40.07	31.22	4.99E-03	3.78E-03	577	12799	1440	41000	2.88E-09
-1 Pax	1.6782	11.04	8.43	1.15E-03	8.95E-04	159	3455	1440	41000	6.51E-01
+1 HNF-4	1.6692+	18.61	14.53	2.64E-03	2.02E-03	268	5959	1440	41000	2.66E-02
-1 RAV1	1.6614+	20.69	15.96	2.27E-03	1.77E-03	298	6545	1440	41000	2.92E-03
+1 Ik-2	1.6118	13.33	10.58	1.44E-03	1.12E-03	192	4336	1440	41000	1.00E+00
+1 STAT3	1.5983+	41.11	33.77	5.72E-03	4.35E-03	592	13844	1440	41000	1.02E-05
+1 STAT6	1.5924+	23.68	19.11	2.98E-03	2.32E-03	341	7836	1440	41000	2.12E-02
-1 p300	1.5906	14.65	11.71	1.62E-03	1.27E-03	211	4801	1440	41000	8.02E-01
-1 USF	1.5636	24.93	21.83	5.86E-03	4.28E-03	359	8950	1440	41000	1.00E+00
+1 USF	1.5578	24.65	21.79	5.88E-03	4.27E-03	355	8934	1440	41000	1.00E+00

TSS GROUP B (DOWNSTREAM)

Up to 150 top ranked promoter elements that appear with frequency >= 10% in the target set

TFBS pattern	ORI	% TAR	% BCG	Prob TARGE	Prob BACKG	# TAR	# BCG	TOT TAR	TOT BCG	p_value
============	======	======	======	=========	=========	=====	=====	=======	=======	=========
+1 E2F	9.7494+	10.07	3.44	1.58E-03	4.76E-04	145	1409	1440	41000	4.00E-25
+1 Churchill	8.8958+	12.36	4.06	1.30E-03	4.45E-04	178	1663	1440	41000	1.48E-33
+1 E2F-1	7.9020+	28.82	11.17	4.31E-03	1.41E-03	415	4579	1440	41000	5.66E-68
+1 ZF5	5.4836+	18.89	8.61	2.72E-03	1.09E-03	272	3530	1440	41000	9.13E-30
-1 E2F-1	5.3958+	24.03	10.94	3.36E-03	1.37E-03	346	4484	1440	41000	7.85E-40
-1 IRF	4.1656+	12.78	6.82	1.76E-03	7.90E-04	184	2797	1440	41000	2.80E-12
-1 ZF5	4.0033+	16.88	8.37	2.17E-03	1.09E-03	243	3430	1440	41000	3.13E-21
+1 IRF	3.7328+	12.15	6.77	1.65E-03	7.92E-04	175	2774	1440	41000	4.75E-10
-1 Hb	3.0340+	13.19	9.46	4.92E-03	2.26E-03	190	3879	1440	41000	5.59E-03
-1 PBF	2.7324+	13.4	9.01	1.82E-03	9.90E-04	193	3696	1440	41000	7.54E-05
+1 GAGA factor	2.6019+	29.31	18.48	5.27E-03	3.21E-03	422	7575	1440	41000	1.50E-19
-1 AP-4	2.4519+	15.97	10.2	3.07E-03	1.96E-03	230	4181	1440	41000	3.30E-08
-1 Ets	2.4336+	17.08	11.04	2.02E-03	1.29E-03	246	4525	1440	41000	1.73E-08
+1 Spz1	2.2982+	39.79	25.19	6.28E-03	4.32E-03	573	10327	1440	41000	9.75E-30
-1 Eve	2.2521+	23.89	16.11	2.80E-03	1.84E-03	344	6607	1440	41000	8.47E-11
+1 c-Ets-1(p54)	2.1649+	12.85	9.07	1.68E-03	1.10E-03	185	3718	1440	41000	3.04E-03
+1 Hb	2.1553	10.62	9.22	4.15E-03	2.22E-03	153	3782	1440	41000	1.00E+00
-1 Dof3	2.0130+	26.94	20.28	3.82E-03	2.52E-03	388	8316	1440	41000	2.28E-06
-1 BR-C Z4	2.0089	11.39	9.73	2.74E-03	1.59E-03	164	3990	1440	41000	1.00E+00
-1 STAT6	1.8957+	26.04	19.18	3.29E-03	2.36E-03	375	7863	1440	41000	3.82E-07
+1 p300	1.8938+	15.35	11.16	1.66E-03	1.21E-03	221	4577	1440	41000	2.09E-03
-1 c-Ets-1(p54)	1.8866+	12.57	9.17	1.54E-03	1.12E-03	181	3760	1440	41000	2.55E-02
-1 ETS	1.8747	11.25	8.2	1.31E-03	9.55E-04	162	3364	1440	41000	7.23E-02
+1 AP-4	1.8676+	13.82	10.17	2.70E-03	1.97E-03	199	4171	1440	41000	1.56E-02
-1 AP-2	1.8400+	17.99	13.05	2.85E-03	2.14E-03	259	5352	1440	41000	1.71E-04
-1 c-Myb	1.8094+	28.33	21.43	3.31E-03	2.42E-03	408	8787	1440	41000	1.22E-06
+1 Helios A	1.7913+	24.38	18.54	2.98E-03	2.19E-03	351	7602	1440	41000	5.91E-05
-1 C1	1.7750+	15.14	11.52	1.79E-03	1.32E-03	218	4722	1440	41000	4.20E-02
-1 STAT3	1.7633+	43.68	33.65	5.90E-03	4.35E-03	629	13796	1440	41000	7.87E-12
+1 Eve	1.7539+	21.04	15.62	2.33E-03	1.79E-03	303	6404	1440	41000	8.21E-05

+1 AP-2gamma	1.7369	10.97	8.07	1.12E-03	8.75E-04	158	3308	1440	41000	1.32E-01
+1 AP-2	1.6601+	17.08	13.1	2.69E-03	2.12E-03	246	5369	1440	41000	1.92E-02
+1 STAT4	1.6444+	43.89	35.12	5.86E-03	4.46E-03	632	14398	1440	41000	1.37E-08
-1 AP-2gamma	1.6391	10.28	7.83	1.06E-03	8.45E-04	148	3211	1440	41000	9.71E-01
+1 FOXD3	1.6389	13.26	12.14	3.29E-03	2.19E-03	191	4978	1440	41000	1.00E+00
+1 Zic1	1.6349+	20.07	15.72	2.40E-03	1.88E-03	289	6446	1440	41000	1.43E-02
+1 FACB	1.6182	10.21	8.04	1.27E-03	9.98E-04	147	3295	1440	41000	1.00E+00
-1 MYB	1.6131+	46.11	37.78	6.47E-03	4.89E-03	664	15488	1440	41000	2.07E-07
+1 HSF1	1.5977	15.9	12.83	1.76E-03	1.37E-03	229	5260	1440	41000	7.44E-01
+1 Ik-2	1.5721	12.99	10.46	1.40E-03	1.10E-03	187	4290	1440	41000	1.00E+00
+1 Lyf-1	1.5689	11.67	9.38	1.26E-03	1.00E-03	168	3846	1440	41000	1.00E+00
-1 HSF1	1.5642	15.42	12.78	1.76E-03	1.36E-03	222	5240	1440	41000	1.00E+00
-1 Pax	1.5595	10.83	8.5	1.11E-03	9.08E-04	156	3487	1440	41000	1.00E+00
+1 RFX	1.555	14.51	11.84	1.62E-03	1.28E-03	209	4856	1440	41000	1.00E+00
-1 FAC1	1.5464	38.89	36.86	1.13E-02	7.70E-03	560	15111	1440	41000	1.00E+00
-1 LIM1	1.5401+	35	28.98	5.07E-03	3.98E-03	504	11883	1440	41000	1.02E-03
-1 HNF-3	1.5394	22.57	20.47	4.79E-03	3.43E-03	325	8393	1440	41000	1.00E+00
-1 HSF	1.5215+	61.39	52.61	1.31E-02	1.01E-02	884	21569	1440	41000	3.72E-08
+1 ETS	1.5168	10.56	8.26	1.14E-03	9.60E-04	152	3385	1440	41000	1.00E+00
+1 VDR	1.5127+	12.78	9	1.40E-03	1.32E-03	184	3688	1440	41000	2.77E-03

TSS GROUP C (UPSTREAM)

Up to 150 top ranked promoter elements that appear with frequency >= 10% in the target set

TFBS pattern	ORI	% TAR	% BCG	Prob TARGE	Prob BACKG	# TAR	# BCG	TOT TAR	TOT BCG	p_value
============	=======	======	======	==========	==========	=====	=====	=======	=======	=========
-1 Elk-1	34.1280+	15.08	3.03	2.38E-03	3.47E-04	293	1242	1943	41000	3.06E-99
+1 Elk-1	24.8751+	13.33	3	1.94E-03	3.47E-04	259	1230	1943	41000	1.01E-77
+1 E2F	18.8670+	12.71	3.24	2.11E-03	4.38E-04	247	1330	1943	41000	2.15E-64
+1 E2F-1	11.1893+	32.01	10.93	5.19E-03	1.36E-03	622	4483	1943	41000	7.19E-126
+1 Churchill	9.8952+	12.87	4.07	1.41E-03	4.49E-04	250	1670	1943	41000	1.79E-49
-1 Churchill	9.5052+	12.92	4.17	1.42E-03	4.63E-04	251	1708	1943	41000	2.22E-48
-1 c-Ets-1(p54)	8.1625+	21.82	9	3.64E-03	1.08E-03	424	3688	1943	41000	3.30E-58
+1 c-Ets-1(p54)	6.7556+	20.12	8.94	3.25E-03	1.08E-03	391	3665	1943	41000	1.51E-45
+1 GC box	6.2593+	12.87	4.58	1.61E-03	7.20E-04	250	1879	1943	41000	2.59E-41

+1 ZF5	5.8517+	18.99	8.04	2.64E-03	1.06E-03	369	3298	1943	41000	5.91E-47
-1 E2F-1	5.7182+	23.57	10.91	3.59E-03	1.36E-03	458	4474	1943	41000	3.12E-50
+1 Ets	4.9496+	23.42	10.86	2.85E-03	1.24E-03	455	4454	1943	41000	1.33E-49
+1 Sp-1	4.9017+	16.47	6.5	3.06E-03	1.58E-03	320	2663	1943	41000	1.07E-45
-1 Sp-1	4.7691+	17.04	6.49	2.90E-03	1.60E-03	331	2659	1943	41000	1.43E-50
-1 ZF5	4.6168+	17.81	8.3	2.36E-03	1.10E-03	346	3403	1943	41000	1.74E-35
-1 Hb	4.5443+	12.2	9.09	7.63E-03	2.25E-03	237	3725	1943	41000	7.42E-03
-1 GC box	4.4770+	12.04	4.85	1.36E-03	7.53E-04	234	1989	1943	41000	7.31E-31
+1 PU.1	4.3886+	11.37	5.51	1.25E-03	5.88E-04	221	2259	1943	41000	3.14E-19
-1 Ets	4.3481+	22.23	10.94	2.69E-03	1.26E-03	432	4484	1943	41000	1.37E-40
-1 ETS	4.2229+	16.78	8.15	1.91E-03	9.31E-04	326	3341	1943	41000	5.38E-30
+1 ETS	4.2065+	16.62	8.06	1.86E-03	9.14E-04	323	3304	1943	41000	8.20E-30
-1 IRF	4.1326+	12.87	6.68	1.66E-03	7.73E-04	250	2737	1943	41000	1.95E-18
-1 PU.1	3.9192+	10.96	5.61	1.20E-03	6.01E-04	213	2300	1943	41000	6.92E-16
+1 Hb	3.6042	11.68	9.13	6.33E-03	2.25E-03	227	3742	1943	41000	1.99E-01
+1 IRF	3.4338+	12.2	6.9	1.54E-03	7.95E-04	237	2829	1943	41000	3.53E-13
+1 Sp1	3.3726+	21.31	11.46	3.33E-03	1.84E-03	414	4697	1943	41000	2.35E-30
+1 PBF	3.2646+	15.59	8.67	1.72E-03	9.48E-04	303	3553	1943	41000	8.25E-19
-1 BR-C Z4	3.0737	10.14	9.39	4.61E-03	1.62E-03	197	3851	1943	41000	1.00E+00
-1 NF-1	3.0358+	18.22	10.7	2.03E-03	1.14E-03	354	4388	1943	41000	7.90E-19
+1 NF-1	2.8805+	17.91	10.7	1.96E-03	1.14E-03	348	4385	1943	41000	2.32E-17
-1 Sp1	2.6593+	20.17	11.93	3.03E-03	1.93E-03	392	4893	1943	41000	7.71E-21
+1 STAT6	2.5898+	30.62	19.11	3.75E-03	2.32E-03	595	7836	1943	41000	3.04E-29
-1 STAT6	2.5641+	30.47	19.18	3.77E-03	2.33E-03	592	7865	1943	41000	4.66E-28
+1 TFIIA	2.4522+	16.83	10.76	1.78E-03	1.13E-03	327	4410	1943	41000	3.76E-12
+1 FOXD3	2.3239	11.99	12.07	5.26E-03	2.25E-03	233	4949	1943	41000	1.00E+00
-1 STAT3	2.2908+	48.84	33.68	6.87E-03	4.35E-03	949	13809	1943	41000	3.94E-38
-1 FACB	2.2715+	11.99	7.97	1.50E-03	9.95E-04	233	3268	1943	41000	2.11E-06
+1 Spz1	2.2373+	37.93	25.13	6.32E-03	4.26E-03	737	10304	1943	41000	7.83E-31
+1 AP-2	2.2222+	19.66	13.05	3.17E-03	2.15E-03	382	5350	1943	41000	1.95E-12
+1 p300	2.1779+	16.83	11.23	1.78E-03	1.23E-03	327	4606	1943	41000	7.59E-10
+1 STAT3	2.1547+	47.71	33.77	6.64E-03	4.35E-03	927	13844	1943	41000	3.83E-32
-1 p300	2.1180+	17.09	11.71	1.85E-03	1.27E-03	332	4801	1943	41000	1.03E-08
-1 AP-4	2.0707+	14.82	10.1	2.76E-03	1.96E-03	288	4139	1943	41000	1.91E-07
-1 HNF-3	2.0138	20.84	20.37	6.89E-03	3.50E-03	405	8350	1943	41000	1.00E+00
-1 FOXD3	1.9566	12.4	11.97	4.09E-03	2.17E-03	241	4909	1943	41000	1.00E+00

+1 TATA	1.9287+	16.57	11	2.10E-03	1.64E-03	322	4512	1943	41000	6.55E-10
-1 AP-2	1.9087+	18.53	13.09	2.88E-03	2.14E-03	360	5367	1943	41000	4.08E-08
+1 Dof3	1.8981+	27.74	20.34	3.45E-03	2.48E-03	539	8340	1943	41000	2.64E-11
-1 Knox3	1.8885+	14.1	10.31	1.53E-03	1.11E-03	274	4229	1943	41000	2.90E-04
+1 AP-4	1.8752+	13.9	10.24	2.72E-03	1.97E-03	270	4199	1943	41000	6.55E-04
+1 FACB	1.8551	10.65	8.03	1.40E-03	1.00E-03	207	3293	1943	41000	6.15E-02
+1 HNF-3	1.8527	22.49	20.39	5.83E-03	3.47E-03	437	8358	1943	41000	1.00E+00
-1 AP-2gamma	1.8102+	10.5	7.77	1.13E-03	8.45E-04	204	3185	1943	41000	2.47E-02
-1 Spz1	1.7800+	35.25	25.81	5.75E-03	4.42E-03	685	10583	1943	41000	2.63E-16
+1 HNF-4	1.7582+	18.58	14.53	2.78E-03	2.02E-03	361	5959	1943	41000	1.57E-03
+1 Knox3	1.7244+	13.23	10.17	1.45E-03	1.09E-03	257	4168	1943	41000	2.40E-02
-1 NF-AT	1.7078+	15.29	11.53	2.46E-03	1.91E-03	297	4727	1943	41000	1.06E-03
-1 C/EBPbeta	1.6498+	16.47	13.13	1.87E-03	1.42E-03	320	5383	1943	41000	3.19E-02
+1 Ik-2	1.6470+	13.64	10.58	1.44E-03	1.12E-03	265	4336	1943	41000	3.15E-02
+1 MYB	1.6366+	46.89	37.66	6.42E-03	4.89E-03	911	15442	1943	41000	5.35E-13
+1 Eve	1.6229+	20.23	15.99	2.36E-03	1.84E-03	393	6554	1943	41000	1.21E-03
+1 Zic1	1.6121+	20.54	15.94	2.39E-03	1.91E-03	399	6534	1943	41000	1.54E-04
-1 Zic3	1.5977+	14.05	10.95	1.61E-03	1.29E-03	273	4489	1943	41000	3.25E-02
+1 RFX	1.5775	14.67	11.79	1.60E-03	1.26E-03	285	4833	1943	41000	1.65E-01
-1 Eve	1.5731+	20.48	16.07	2.27E-03	1.84E-03	398	6587	1943	41000	4.73E-04
-1 PBF	1.5478	10.65	8.52	1.16E-03	9.40E-04	207	3493	1943	41000	1.00E+00
+1 PPAR	1.5351	11.22	9.31	1.45E-03	1.14E-03	218	3816	1943	41000	1.00E+00
+1 Lyf-1	1.5322	11.48	9.27	1.22E-03	9.86E-04	223	3799	1943	41000	1.00E+00
+1 Zic3	1.5195	12.97	10.45	1.49E-03	1.22E-03	252	4283	1943	41000	4.88E-01
+1 MYBAS1	1.5093	14.67	12.18	1.64E-03	1.31E-03	285	4993	1943	41000	1.00E+00
-1 MYB	1.5019+	44.57	37.66	6.19E-03	4.88E-03	866	15440	1943	41000	1.03E-06

TSS GROUP C (DOWNSTREAM)

Up to 150 top ranked promoter elements that appear with frequency >= 10% in the target set

TFBS pattern	ORI	% TAR	% BCG	Prob TARGE	Prob BACKG	# TAR	# BCG	TOT TAR	TOT BCG	p_value
============	=======	======	======	==========	==========	=====	=====	=======	=======	=========
+1 Churchill	52.6898+	26.97	4.06	3.53E-03	4.45E-04	524	1663	1943	41000	3.29E-238
-1 Churchill	41.3513+	25.27	4.17	3.18E-03	4.67E-04	491	1708	1943	41000	8.88E-207
+1 E2F	39.1052+	19.66	3.44	3.25E-03	4.76E-04	382	1409	1943	41000	1.56E-149

+1 LBP-1	28.2256+	15.95	3.05	1.76E-03	3.26E-04	310	1250	1943	41000	2.15E-110
+1 E2F-1	26.7377+	48.12	11.17	8.74E-03	1.41E-03	935	4579	1943	41000	0.00E+00
+1 ZF5	26.6602+	38.24	8.61	6.52E-03	1.09E-03	743	3530	1943	41000	1.10E-255
-1 LBP-1	24.0387+	14.87	3.14	1.75E-03	3.44E-04	289	1289	1943	41000	3.70E-93
-1 ZF5	22.7921+	35.67	8.37	5.84E-03	1.09E-03	693	3430	1943	41000	9.07E-226
-1 E2F-1	18.9913+	40.4	10.94	7.04E-03	1.37E-03	785	4484	1943	41000	3.37E-226
+1 ETF	18.7912+	12.87	2.9	1.64E-03	3.87E-04	250	1190	1943	41000	1.23E-74
-1 AP-2alpha	18.2404+	24.86	6.03	2.86E-03	6.47E-04	483	2471	1943	41000	4.49E-143
-1 E2F	17.7826+	13.12	3.38	2.13E-03	4.64E-04	255	1385	1943	41000	5.40E-66
-1 AP-2	15.6925+	45.91	13.05	9.54E-03	2.14E-03	892	5352	1943	41000	4.03E-252
+1 AP-2alpha	14.8915+	22.44	6	2.56E-03	6.43E-04	436	2462	1943	41000	4.16E-114
-1 Muscle initiator	14.7185+	13.59	3.61	1.52E-03	3.89E-04	264	1479	1943	41000	4.48E-66
+1 AP-2gamma	14.5122+	29.9	8.07	3.43E-03	8.75E-04	581	3308	1943	41000	1.93E-157
+1 AP-2	13.4353+	44.11	13.1	8.45E-03	2.12E-03	857	5369	1943	41000	2.59E-227
-1 AP-2gamma	12.1571+	26.45	7.83	3.04E-03	8.45E-04	514	3211	1943	41000	7.44E-122
-1 Dde box	11.1583+	16.62	5.06	1.82E-03	5.37E-04	323	2074	1943	41000	2.33E-69
-1 Eve	8.1560+	42.31	16.11	5.72E-03	1.84E-03	822	6607	1943	41000	5.60E-152
-1 AP-4	8.1464+	28.41	10.2	5.73E-03	1.96E-03	552	4181	1943	41000	4.99E-101
+1 CP2/LBP-1c/LSF	7.6533+	15.9	5.85	1.75E-03	6.22E-04	309	2397	1943	41000	1.14E-49
+1 Sp1	7.1730+	32.22	11.74	4.99E-03	1.91E-03	626	4814	1943	41000	5.49E-115
+1 AP-4	6.7771+	26.97	10.17	5.02E-03	1.97E-03	524	4171	1943	41000	1.35E-87
+1 Eve	6.7605+	38.34	15.62	4.94E-03	1.79E-03	745	6404	1943	41000	4.36E-119
+1 GC box	6.4654+	13.43	4.58	1.61E-03	7.32E-04	261	1876	1943	41000	1.60E-46
+1 Spz1	6.1932+	58	25.19	1.16E-02	4.32E-03	1127	10327	1943	41000	4.94E-192
+1 VDR	5.3551+	21.82	9	2.91E-03	1.32E-03	424	3688	1943	41000	3.30E-58
-1 LF-A1	5.1885+	16.26	7.14	1.71E-03	7.50E-04	316	2927	1943	41000	1.75E-36
+1 Zic3	5.1117+	22.9	10.32	2.80E-03	1.21E-03	445	4230	1943	41000	1.62E-51
+1 LF-A1	5.0993+	15.59	7.09	1.72E-03	7.44E-04	303	2907	1943	41000	3.41E-32
+1 TFII-I	4.9632+	12.87	5.65	1.47E-03	6.73E-04	250	2316	1943	41000	6.59E-28
+1 Dde box	4.6363+	11.12	5.25	1.20E-03	5.48E-04	216	2153	1943	41000	5.98E-20
+1 Zic1	4.5507+	31.96	15.72	4.20E-03	1.88E-03	621	6446	1943	41000	9.95E-64
-1 CP2/LBP-1c/LSF	4.2733+	11.58	5.74	1.28E-03	6.05E-04	225	2352	1943	41000	1.62E-18
+1 Sp-1	4.2587+	15.9	6.45	2.76E-03	1.60E-03	309	2645	1943	41000	1.15E-41
-1 FACB	3.8026+	15.9	8.1	1.95E-03	1.01E-03	309	3322	1943	41000	7.01E-25
-1 Sp1	3.7196+	24.29	11.7	3.42E-03	1.91E-03	472	4797	1943	41000	1.48E-47
+1 FACB	3.5330+	14.87	8.04	1.90E-03	9.98E-04	289	3295	1943	41000	2.07E-19

+1 Zic2	3.3791+	12.92	7.16	1.47E-03	7.86E-04	251	2934	1943	41000	3.72E-15
+1 ADR1	3.3142+	18.68	9.43	2.66E-03	1.59E-03	363	3868	1943	41000	7.96E-31
+1 GAGA factor	3.2926+	35.1	18.48	5.57E-03	3.21E-03	682	7575	1943	41000	9.58E-61
-1 Spz1	3.1677+	44.47	25.15	7.72E-03	4.31E-03	864	10312	1943	41000	3.09E-69
+1 MZF1	3.0329+	27.23	14.52	3.76E-03	2.32E-03	529	5954	1943	41000	4.22E-42
-1 Sp-1	2.8319+	13.43	6.44	2.19E-03	1.61E-03	261	2641	1943	41000	6.59E-24
+1 XPF-1	2.7797+	12.04	7.14	1.22E-03	7.40E-04	234	2928	1943	41000	6.49E-11
+1 p300	2.7545+	18.27	11.16	2.03E-03	1.21E-03	355	4577	1943	41000	2.68E-16
-1 XPF-1	2.6884+	11.73	7.07	1.18E-03	7.31E-04	228	2900	1943	41000	6.73E-10
-1 COUP	2.6036+	19.2	11.89	2.12E-03	1.32E-03	373	4876	1943	41000	2.06E-16
-1 RAR	2.6036+	19.2	11.89	2.12E-03	1.32E-03	373	4876	1943	41000	2.06E-16
-1 Zic3	2.5948+	17.19	10.49	1.96E-03	1.24E-03	334	4300	1943	41000	3.45E-15
+1 MyoD	2.5840+	10.96	7.26	1.61E-03	9.39E-04	213	2976	1943	41000	8.80E-06
-1 RAV1	2.5377+	24.81	15.7	2.80E-03	1.74E-03	482	6439	1943	41000	7.20E-21
-1 LIM1	2.4837+	44.16	28.98	6.48E-03	3.98E-03	858	11883	1943	41000	1.82E-40
+1 RAV1	2.3197+	24.14	15.97	2.73E-03	1.78E-03	469	6546	1943	41000	1.40E-16
-1 p53 decamer	2.2685+	36.03	24.44	4.60E-03	2.99E-03	700	10021	1943	41000	1.29E-25
+1 AP-2rep	2.2213+	45.24	30.97	5.74E-03	3.78E-03	879	12698	1943	41000	8.72E-35
-1 GAGA factor	2.1385+	29.49	18.55	4.49E-03	3.34E-03	573	7605	1943	41000	5.91E-27
-1 ADR1	2.1248+	14.46	9.41	2.16E-03	1.56E-03	281	3857	1943	41000	3.40E-09
-1 MZF1	2.1026+	21.67	14.24	3.17E-03	2.29E-03	421	5840	1943	41000	8.48E-15
+1 p53 decamer	2.0316+	34.38	24.57	4.36E-03	3.01E-03	668	10074	1943	41000	3.62E-18
-1 PPAR	2.0267+	13.95	9.62	1.65E-03	1.18E-03	271	3943	1943	41000	2.31E-06
+1 Ik-2	1.9689+	14.46	10.46	1.57E-03	1.10E-03	281	4290	1943	41000	7.90E-05
-1 VDR	1.9282+	13.12	9.08	1.76E-03	1.32E-03	255	3722	1943	41000	9.99E-06
+1 COUP	1.9173+	16.52	12.06	1.85E-03	1.32E-03	321	4946	1943	41000	1.72E-05
+1 RAR	1.9173+	16.52	12.06	1.85E-03	1.32E-03	321	4946	1943	41000	1.72E-05
-1 Zic1	1.8551+	21.72	15.97	2.64E-03	1.94E-03	422	6546	1943	41000	8.58E-08
+1 RFX	1.7989+	15.85	11.84	1.71E-03	1.28E-03	308	4856	1943	41000	2.82E-04
-1 PPAR direct repeat 1	1.7464	10.09	7.78	1.11E-03	8.22E-04	196	3190	1943	41000	3.16E-01
+1 Ets	1.7294+	14.82	10.98	1.64E-03	1.28E-03	288	4503	1943	41000	3.90E-04
-1 c-Ets-1(p54)	1.6967	11.07	9.17	1.58E-03	1.12E-03	215	3760	1943	41000	1.00E+00
+1 Knox3	1.6792+	13.33	10.18	1.40E-03	1.09E-03	259	4174	1943	41000	1.45E-02
-1 STAT3	1.6232+	42.82	33.65	5.54E-03	4.35E-03	832	13796	1943	41000	2.15E-13
-1 Ets	1.6155+	14.41	11.04	1.59E-03	1.29E-03	280	4525	1943	41000	7.32E-03
-1 TTF-1	1.6016+	47.56	39.18	6.65E-03	5.04E-03	924	16065	1943	41000	2.34E-10

+1 c-Ets-1(p54)	1.5892	11.12	9.07	1.43E-03	1.10E-03	216	3718	1943	41000	1.00E+00
+1 PPAR	1.5775	11.99	9.54	1.47E-03	1.17E-03	233	3912	1943	41000	4.40E-01
+1 MAF	1.5581	22.23	18.56	2.73E-03	2.10E-03	432	7611	1943	41000	5.95E-02
-1 STAT6	1.5549+	24.4	19.18	2.88E-03	2.36E-03	474	7863	1943	41000	2.79E-05
-1 Knox3	1.5302	12.61	10.14	1.33E-03	1.08E-03	245	4156	1943	41000	5.36E-01
-1 HNF-4	1.5176+	18.53	14.96	2.58E-03	2.11E-03	360	6134	1943	41000	2.47E-02

TSS GROUP D (UPSTREAM)

Up to 150 top ranked promoter elements that appear with frequency >= 10% in the target set

TFBS pattern	ORI	% TAR	% BCG	Prob TARGE	Prob BACKG	# TAR	# BCG	TOT TAR	TOT BCG	p_value
============	======	======	======	==========	==========	=====	=====	=======	=======	=========
+1 GC box	6.9508+	12.17	4.58	1.89E-03	7.20E-04	186	1879	1528	41000	6.42E-28
+1 Sp-1	5.4266+	14.66	6.5	3.80E-03	1.58E-03	224	2663	1528	41000	5.14E-25
-1 Sp-1	4.5916+	15.45	6.49	3.08E-03	1.60E-03	236	2659	1528	41000	9.42E-30
-1 c-Ets-1(p54)	4.2451+	17.15	9	2.41E-03	1.08E-03	262	3688	1528	41000	1.11E-19
+1 ETS	3.8766+	16.03	8.06	1.78E-03	9.14E-04	245	3304	1528	41000	1.84E-20
+1 Ets	3.6640+	20.16	10.86	2.45E-03	1.24E-03	308	4454	1528	41000	3.81E-22
+1 Sp1	3.6156+	19.7	11.46	3.86E-03	1.84E-03	301	4697	1528	41000	8.33E-17
+1 E2F-1	3.5988+	20.22	10.93	2.64E-03	1.36E-03	309	4483	1528	41000	5.11E-22
+1 ADR1	3.3222+	14.66	9.15	3.10E-03	1.50E-03	224	3750	1528	41000	1.03E-08
+1 c-Ets-1(p54)	2.9495+	14.2	8.94	2.01E-03	1.08E-03	217	3665	1528	41000	5.02E-08
-1 Hb	2.6994	12.24	9.09	4.52E-03	2.25E-03	187	3725	1528	41000	5.09E-02
-1 C/EBPbeta	2.5446+	20.48	13.13	2.32E-03	1.42E-03	313	5383	1528	41000	5.51E-12
+1 PBF	2.4855+	13.68	8.67	1.49E-03	9.48E-04	209	3553	1528	41000	2.11E-07
-1 Ets	2.4400+	17.02	10.94	1.97E-03	1.26E-03	260	4484	1528	41000	2.99E-09
+1 C/EBPbeta	2.4015+	20.16	13.18	2.24E-03	1.43E-03	308	5403	1528	41000	1.09E-10
+1 IRF	2.3888+	10.54	6.9	1.24E-03	7.95E-04	161	2829	1528	41000	2.64E-04
+1 STAT6	2.3400+	28.34	19.11	3.66E-03	2.32E-03	433	7836	1528	41000	1.27E-14
+1 TATA	2.2222+	17.6	11	2.28E-03	1.64E-03	269	4512	1528	41000	5.63E-11
-1 E2F-1	2.1636+	15.64	10.91	2.05E-03	1.36E-03	239	4474	1528	41000	3.35E-05
+1 Zic3	2.1496+	14.53	10.45	1.89E-03	1.22E-03	222	4283	1528	41000	1.01E-03
-1 ETS	2.1212+	12.17	8.15	1.32E-03	9.31E-04	186	3341	1528	41000	1.14E-04
-1 ZF5	2.1098+	12.83	8.3	1.50E-03	1.10E-03	196	3403	1528	41000	4.22E-06
+1 RFX	2.0943+	17.02	11.79	1.83E-03	1.26E-03	260	4833	1528	41000	3.72E-06

-1 NF-1	2.0795+	15.45	10.7	1.64E-03	1.14E-03	236	4388	1528	41000	2.45E-05
+1 ZF5	1.9688+	11.91	8.04	1.41E-03	1.06E-03	182	3298	1528	41000	2.80E-04
+1 C_EBP	1.9335+	29.19	21.4	3.49E-03	2.46E-03	446	8773	1528	41000	2.14E-09
+1 Zic1	1.9311+	21.27	15.94	2.76E-03	1.91E-03	325	6534	1528	41000	6.97E-05
+1 TCF11	1.9300+	10.27	7.32	1.05E-03	7.66E-04	157	3002	1528	41000	3.37E-02
+1 VDR	1.9286+	12.11	8.8	1.80E-03	1.29E-03	185	3606	1528	41000	1.74E-02
+1 HNF-1	1.9263+	19.9	15.78	3.23E-03	2.11E-03	304	6470	1528	41000	2.32E-02
+1 Lyf-1	1.9006+	12.7	9.27	1.37E-03	9.86E-04	194	3799	1528	41000	1.36E-02
-1 PBF	1.8577+	11.71	8.52	1.27E-03	9.40E-04	179	3493	1528	41000	2.60E-02
-1 STAT6	1.8462+	26.31	19.18	3.14E-03	2.33E-03	402	7865	1528	41000	2.47E-08
+1 NF-1	1.8171+	14.53	10.7	1.53E-03	1.14E-03	222	4385	1528	41000	4.89E-03
+1 Spz1	1.8087+	32.72	25.13	5.92E-03	4.26E-03	500	10304	1528	41000	6.29E-08
-1 C1	1.8082+	15.97	11.7	1.78E-03	1.34E-03	244	4797	1528	41000	1.03E-03
-1 c-Myb	1.7987+	28.01	21.42	3.31E-03	2.41E-03	428	8782	1528	41000	2.19E-06
-1 MYBAS1	1.7725+	16.1	12.16	1.75E-03	1.31E-03	246	4985	1528	41000	7.88E-03
-1 MYB	1.7706+	48.17	37.66	6.75E-03	4.88E-03	736	15440	1528	41000	2.13E-13
-1 AP-4	1.7667	13.15	10.1	2.66E-03	1.96E-03	201	4139	1528	41000	1.56E-01
+1 STAT3	1.7620+	43.78	33.77	5.92E-03	4.35E-03	669	13844	1528	41000	1.50E-12
-1 C_EBP	1.6811+	27.75	21.29	3.14E-03	2.43E-03	424	8728	1528	41000	4.08E-06
+1 AP-4	1.6771	12.96	10.24	2.61E-03	1.97E-03	198	4199	1528	41000	7.60E-01
-1 AP-1	1.6672+	37.76	30.96	1.13E-02	8.23E-03	577	12695	1528	41000	2.57E-05
+1 Knox3	1.6608	13.15	10.17	1.40E-03	1.09E-03	201	4168	1528	41000	2.24E-01
+1 AP-1	1.5757+	38.02	31.14	1.05E-02	8.14E-03	581	12768	1528	41000	1.83E-05
-1 BR-C Z4	1.5336	10.01	9.39	2.33E-03	1.62E-03	153	3851	1528	41000	1.00E+00
+1 c-Myb	1.5215+	26.18	21.38	2.99E-03	2.41E-03	400	8764	1528	41000	9.79E-03
+1 MYB	1.5211+	45.35	37.66	6.17E-03	4.89E-03	693	15442	1528	41000	1.49E-06
-1 Dof3	1.5204+	25.65	20.2	3.01E-03	2.51E-03	392	8283	1528	41000	3.72E-04

TSS GROUP D (DOWNSTREAM)

Up to 150 top ranked promoter elements that appear with frequency >= 10% in the target set

TFBS pattern	ORI	% TAR	% BCG	Prob TARGE	Prob BACKG	# TAR	# BCG	TOT TAR	TOT BCG	p_value
============	=======	======	======	==========	==========	=====	=====	=======	=======	=========
+1 E2F-1	2.9267+	18.13	11.17	2.54E-03	1.41E-03	277	4579	1528	41000	3.97E-12
-1 E2F-1	2.2621+	16.69	10.94	2.03E-03	1.37E-03	255	4484	1528	41000	3.43E-08

-1 AP-4	2.0636+	14.14	10.2	2.92E-03	1.96E-03	216	4181	1528	41000	1.82E-03
+1 HSF1	1.8969+	17.67	12.83	1.89E-03	1.37E-03	270	5260	1528	41000	1.05E-04
+1 AP-4	1.8641	13.09	10.17	2.85E-03	1.97E-03	200	4171	1528	41000	3.12E-01
-1 Dof3	1.7689+	27.09	20.28	3.34E-03	2.52E-03	414	8316	1528	41000	3.37E-07
-1 Hb	1.6937	12.37	9.46	2.93E-03	2.26E-03	189	3879	1528	41000	2.16E-01
-1 DBP	1.6729+	26.77	20.97	3.15E-03	2.40E-03	409	8599	1528	41000	1.02E-04
-1 Eve	1.6322+	20.68	16.11	2.34E-03	1.84E-03	316	6607	1528	41000	3.57E-03
-1 HSF1	1.6157	15.84	12.78	1.77E-03	1.36E-03	242	5240	1528	41000	5.49E-01
+1 STAT4	1.5972+	43.78	35.12	5.71E-03	4.46E-03	669	14398	1528	41000	6.25E-09
-1 HSF	1.5867+	63.94	52.61	1.31E-02	1.01E-02	977	21569	1528	41000	1.45E-15
-1 c-Myb	1.5440+	26.37	21.43	3.03E-03	2.42E-03	403	8787	1528	41000	5.60E-03
-1 PBF	1.5436	11.26	9.01	1.22E-03	9.90E-04	172	3696	1528	41000	1.00E+00
+1 Ets	1.5236	13.48	10.98	1.59E-03	1.28E-03	206	4503	1528	41000	1.00E+00
+1 Ncx	1.5156+	41.75	34.53	5.45E-03	4.34E-03	638	14157	1528	41000	7.78E-06
