# Supplementary material for: Mice and Men: Their Promoter Properties
Source: PLoS Genet. 2006 Apr 28;2(4):e54. doi: 10.1371/journal.pgen.0020054 (PMC1449896; doi:10.1371/journal.pgen.0020054)
Supplement: Table S2 — PEs are compared relative to the same GC richness and same location (upstream or downstream) in different TSS types. The signs plus or minus indicate whether the PE was found to be significantly enriched in the considered region for the considered TSS type. For example, the first column (yellow), which shows comparison between the AT-rich downstream domains in TSS types B and D, contains a common element denoted as “+ − Ets.” This means that Ets was found significantly enriched for the B type, but its enrichment was not significant for D type. When an element is unique for one or another group, then it is associated only with one plus or minus sign. (53 KB PDF) [file pgen.0020054.st002.pdf]

Table S2. Common and specific promoter elements in the four TSS types
Minimum 10% of target promoter sequences contain the motif

ATrich_B &. D_dwn		ATrich_C & D_up		GCrich_B & A_up		GCrich_C & A_dwn	
**Common**		**Common**		**Common**		**Common**	
++	AP-4	+-	AP-4	++	ADR1	++	ADR1
++	Dof3	--	BR-C Z4	++	AP-2	++	AP-2
++	E2F-1	++	C/EBPbeta	++	AP-2alpha	++	AP-2alpha
+-	Ets	++	Dof3	++	AP-2gamma	++	AP-2gamma
++	Eve	++	E2F-1	++	AP-4	++	AP-2rep
++	HSF	++	ETS	++	Alfin1	++	AP-4
-+	HSF1	++	Ets	++	COUP	++	COUP
+-	Hb	++	GC box	++	CP2/LBP-1c/LSF	++	CP2/LBP-1c/LSF
+-	PBF	+-	Hb	++	CREB	++	Churchill
++	STAT4	++	IRF	++	Churchill	++	Dde box
++	c-Myb	+-	Knox3	++	Dde box	++	E2F
		-+	Lyf-1	++	E2F	++	E2F-1
		++	MYB	++	E2F-1	++	ETF
		-+	MYBAS1	++	EGR	++	Eve
		++	NF-1	++	ETF	++	FACB
		++	PBF	++	Elk-1	++	GAGA factor
		-+	RFX	++	Ets	++	GC box
		++	STAT3	++	Eve	++	LBP-1
		++	STAT6	++	FACB	++	LF-A1
		++	Sp-1	++	GAGA factor	++	LIM1
		++	Sp1	++	GC box	++	MZF1
		++	Spz1	++	Knox3	++	Muscle initiator
		++	TATA	++	LF-A1	++	RAR
		++	ZF5	++	LIM1	++	RAV1
		++	Zic1	++	MAZ	++	RFX
		++	Zic3	++	MZF1	++	Sp-1
		++	c-Ets-1(p54)	++	Muscle initiator	++	Sp1
				++	NF-1	++	Spz1
				++	PU.1	++	TFII-I
				++	RAR	++	VDR
				++	RAV1	++	ZF5
				++	Sp-1	++	Zic1
				++	Sp1	++	Zic2
				++	Sp3	++	Zic3
				++	Spz1	++	p300
				++	TFII-I	++	p53 decamer
				++	VDR		
				++	ZF5		
				++	Zic1		
				++	Zic2		
				++	Zic3		
				++	c-Ets-1(p54)		
				++	p300		
				++	p53 decamer		
**Unique for ATrich_B**		**Unique for ATrich_C**		**Unique for GCrich_B**		**Unique for GCrich_C**	
+	AP-2	+	AP-2	+	AP-2rep	+	Ets
-	AP-2gamma	+	AP-2gamma	+	CAC-binding protein	+	HNF-4
-	BR-C Z4	+	Churchill	+	ETS	+	Ik-2
+	C1	+	E2F	+	HNF-4	+	Knox3
+	Churchill	+	Elk-1	-	Ik-2	-	MAF
+	E2F	+	Eve	+	Lyf-1	+	MyoD
-	ETS	+	FACB	+	MYBAS1	+	PPAR
-	FAC1	-	FOXD3	+	P	-	PPAR direct repeat 1
-	FACB	-	HNF-3	+	PPAR	+	STAT3
-	FOXD3	+	HNF-4	-	Pax	+	STAT6
+	GAGA factor	+	Ik-2	+	RFX	+	TTF-1
-	HNF-3	+	NF-AT	+	STAT3	+	XPF-1
+	Helios A	-	PPAR	+	STAT6	-	c-Ets-1(p54)
+	IRF	+	PU.1	-	USF		
-	Ik-2	+	TFIIA				
+	LIM1	+	p300				
-	Lyf-1						
+	MYB						
-	Pax						
-	RFX						
+	STAT3						
+	STAT6						
+	Spz1						
+	VDR						
+	ZF5						
+	Zic1						
+	c-Ets-1(p54)						
+	p300						
**Unique for D_dwn**		**Unique for D_up**		**Unique for GCrich_A_up**		**Unique for GCrich_A_dwn**	
+	DBP	+	ADR1	+	AHRHIF	+	Adf-1
+	Ncx	+	AP-1	+	CF1 / USP	+	Alfin1
		+	C1	+	Hairy	+	CF1 / USP
		+	C_EBP	+	KROX	+	EGR
		+	HNF-1	+	LBP-1	+	P
		+	TCF11	+	MAZR	+	PCF2
		+	VDR	+	PCF2		
		+	c-Myb	+	USF2		
				+	c-Myc:Max		
