# Supplementary material for: Mice and Men: Their Promoter Properties
Source: PLoS Genet. 2006 Apr 28;2(4):e54. doi: 10.1371/journal.pgen.0020054 (PMC1449896; doi:10.1371/journal.pgen.0020054)
Supplement: Table S3 — The yellow highlighted TFs are unique for the considered groups when compared with the same upstream or downstream segment of another TSS type with the same GC richness. (47 KB PDF) [file pgen.0020054.st003.pdf]

Table S3

The lists of significant PEs in different TSS types and in upstream and downstream segments.

The yellow highlighted TFs are unique for the considred groups when compared with the same upstream or downstream segment of another TSS group with the same GC-richness

**B_dwn**	**D_dwn**	**C_up**	**D_up**	**B_up**	**A_up**	**C_dwn**	**A_dwn**
AP-4	AP-4	AP-4	C/EBPbeta	ADR1	ADR1	ADR1	ADR1
Dof3	Dof3	C/EBPbeta	Dof3	AP-2	AP-2	AP-2	AP-2
E2F-1	E2F-1	Dof3	E2F-1	AP-2alpha	AP-2alpha	AP-2alpha	AP-2alpha
Ets	Eve	E2F-1	ETS	AP-2gamma	AP-2gamma	AP-2gamma	AP-2gamma
Eve	HSF	ETS	Ets	AP-4	AP-4	AP-2rep	AP-2rep
HSF	HSF1	Ets	GC box	Alfin1	Alfin1	AP-4	AP-4
Hb	STAT4	GC box	IRF	COUP	COUP	COUP	COUP
PBF	c-Myb	Hb	Lyf-1	CP2/LBP-1c/LSF	CP2/LBP-1c/LSF	CP2/LBP-1c/LSF	CP2/LBP-1c/LSF
STAT4	DBP	IRF	MYB	CREB	CREB	Churchill	Churchill
c-Myb	Ncx	Knox3	MYBAS1	Churchill	Churchill	Dde box	Dde box
AP-2		MYB	NF-1	Dde box	Dde box	E2F	E2F
C1		NF-1	PBF	E2F	E2F	E2F-1	E2F-1
Churchill		PBF	RFX	E2F-1	E2F-1	ETF	ETF
E2F		STAT3	STAT3	EGR	EGR	Eve	Eve
GAGA factor		STAT6	STAT6	ETF	ETF	FACB	FACB
Helios A		Sp-1	Sp-1	Elk-1	Elk-1	GAGA factor	GAGA factor
IRF		Sp1	Sp1	Ets	Ets	GC box	GC box
LIM1		Spz1	Spz1	Eve	Eve	LBP-1	LBP-1
MYB		TATA	TATA	FACB	FACB	LF-A1	LF-A1
STAT3		ZF5	ZF5	GAGA factor	GAGA factor	LIM1	LIM1
STAT6		Zic1	Zic1	GC box	GC box	MZF1	MZF1
Spz1		Zic3	Zic3	Knox3	Knox3	Muscle initiator	Muscle initiator
VDR		c-Ets-1(p54)	c-Ets-1(p54)	LF-A1	LF-A1	RAR	RAR
ZF5		AP-2	ADR1	LIM1	LIM1	RAV1	RAV1
Zic1		AP-2gamma	AP-1	MAZ	MAZ	RFX	RFX
c-Ets-1(p54)		Churchill	C1	MZF1	MZF1	Sp-1	Sp-1
p300		E2F	C_EBP	Muscle initiator	Muscle initiator	Sp1	Sp1
		Elk-1	HNF-1	NF-1	NF-1	Spz1	Spz1
		Eve	TCF11	PU.1	PU.1	TFII-I	TFII-I

FACB	VDR	RAR	RAR	VDR	VDR
HNF-4	c-Myb	RAV1	RAV1	ZF5	ZF5
Ik-2		Sp-1	Sp-1	Zic1	Zic1
NF-AT		Sp1	Sp1	Zic2	Zic2
PU.1		Sp3	Sp3	Zic3	Zic3
TFIIA		Spz1	Spz1	p300	p300
p300		TFII-I	TFII-I	p53 decamer	p53 decamer
		VDR	VDR	Ets	Adf-1
		ZF5	ZF5	HNF-4	Alfin1
		Zic1	Zic1	Ik-2	CF1 / USP
		Zic2	Zic2	Knox3	EGR
		Zic3	Zic3	MyoD	P
		c-Ets-1(p54)	c-Ets-1(p54)	PPAR	PCF2
		p300	p300	STAT3	
		p53 decamer	p53 decamer	STAT6	
		AP-2rep	AHRHIF	TTF-1	
		CAC-binding prote	CF1 / USP	XPF-1	
		ETS	Hairy		
		HNF-4	KROX		
		Lyf-1	LBP-1		
		MYBAS1	MAZR		
		P	PCF2		
		PPAR	USF2		
		RFX	c-Myc:Max		
		STAT3			
		STAT6			
