# Supplementary material for: Mice and Men: Their Promoter Properties
Source: PLoS Genet. 2006 Apr 28;2(4):e54. doi: 10.1371/journal.pgen.0020054 (PMC1449896; doi:10.1371/journal.pgen.0020054)
Supplement: Table S4 — We considered only those TSSs whose generated transcripts belong to the same homology group as defined on the NCBI Web site (ftp://ftp.ncbi.nih.gov/pub/HomoloGene). We only considered GO categories that were supported by at least 60 TSSs and where the target TSS type was supported by at least three TSSs. (96 KB PDF) [file pgen.0020054.st004.pdf]

Table S4. The list of GO categories that preserve enrichment in specific TSS type between human and mouse. We considered only those TSSs whose generated transcripts belong to the same homology group as defined on NCBI web-site. We only considered GO categories that were supported by at least 60 TSSs and where the target TSS type was supported by at least 3 TSSs.

**Type A**	**Human**				**mouse**			
**GOID**	**Total #**	**#A**	**%A**	**enrichment**	**Total #**	**#A**	**%A**	**enrichment**
**Matched GO groups: 0, Total # of GO groups: 0**								

**Type B**	**Human**				**Mouse**			
**GOID**	**Total #**	**#B**	**%B**	**enrichment**	**Total #**	**#B**	**%B**	**enrichment**
GO:0003700	102	6	5.88	3.74	117	7	5.98	2.77
GO:0003676	91	4	4.4	2.79	228	9	3.95	1.83
GO:0006355	239	10	4.18	2.66	303	14	4.62	2.14
GO:0005737	256	9	3.52	2.23	285	13	4.56	2.11
GO:0006350	217	6	2.76	1.76	231	12	5.19	2.4
GO:0016491	149	4	2.68	1.71	196	9	4.59	2.13
GO:0005634	711	19	2.67	1.7	886	42	4.74	2.19
**Matched GO groups: 7, Total # of GO groups: 7, Coverage: 100%**								

**Type C**	**human**				**Mouse**			
**GOID**	**Total #**	**#C**	**%C**	**enrichment**	**Total #**	**#C**	**%C**	**enrichment**
GO:0005615	69	8	11.59	4.14	428	41	9.58	2.58
GO:0007155	68	7	10.29	3.67	88	8	9.09	2.45
GO:0006412	105	7	6.67	2.38	118	9	7.63	2.05
GO:0003735	69	4	5.8	2.07	93	8	8.6	2.32
GO:0005795	109	6	5.5	1.96				
GO:0016874	73	4	5.48	1.95	90	7	7.78	2.09
GO:0005509	116	6	5.17	1.85	137	11	8.03	2.16
GO:0005624	120	6	5	1.78				
GO:0005886	81	4	4.94	1.76	95	8	8.42	2.27
GO:0007165	146	7	4.79	1.71				
GO:0004842	85	4	4.71	1.68				
GO:0003723	155	7	4.52	1.61				
GO:0007049	89	4	4.49	1.6	114	7	6.14	1.65
GO:0008270	205	9	4.39	1.57	176	12	6.82	1.84
**Matched GO groups: 9, Total # of GO groups: 14, Coverage: 64.29%**								

**Type D**	**human**				**mouse**			
**GOID**	**Total #**	**#D**	**%D**	**enrichment**	**Total #**	**#D**	**%D**	**enrichment**
GO:0005615	69	6	8.7	5.28	428	26	6.07	3.88
GO:0006810	113	8	7.08	4.3	392	11	2.81	1.79
GO:0007165	146	8	5.48	3.33				
GO:0005887	147	7	4.76	2.89				
GO:0004872	92	4	4.35	2.64	184	10	5.43	3.47
GO:0005509	116	4	3.45	2.1	137	9	6.57	4.19
GO:0008372	134	4	2.99	1.81	260	8	3.08	1.96
GO:0016020	312	9	2.88	1.75	416	14	3.37	2.15
GO:0016021	451	13	2.88	1.75	768	27	3.52	2.24
GO:0006355	239	6	2.51	1.53	303	10	3.3	2.11
**Matched GO groups: 8, Total # of GO groups: 10, Coverage: 80%**								
