# Supplementary material for: Mice and Men: Their Promoter Properties
Source: PLoS Genet. 2006 Apr 28;2(4):e54. doi: 10.1371/journal.pgen.0020054 (PMC1449896; doi:10.1371/journal.pgen.0020054)
Supplement: Table S5 — The table presents the total number of TSSs associated with the category (GO or expression library), the number of TSSs of individual TSS type, the percentage of TSSs in that TSS type, enrichment of TSSs in the TSS type relative to what can be expected based on the distribution of all TSSs in mouse across all four TSS types, and Bonferroni corrected p-values calculated based on right-sided Fisher's exact tests for the null hypothesis that the proportion of TSS type found in the target group is the same as that of the general mouse distribution. For example, there are 253 transcripts associated with GO:0006955. Of these, 52 transcripts have a TSS of type C. For the number of transcripts in this GO category, one would expect only 11 transcripts with TSSs of type C. Thus, in this GO category, we have 4.85-fold enrichment of transcripts of this type (compared to what we would expect based on the distribution of all transcripts across the four TSS types). If in any of the GO/eVOC categories or tissue libraries, at least one of the TSS groups of transcripts has enrichment that is 1.5-fold or greater than the expected value, we consider such TSS type overrepresented. (2.9 MB PDF) [file pgen.0020054.st005.pdf]

GOID	Total	#A	%A	Change A	#B	%B	Change B	#C	%C	Change C	#D	%D	Change D	p-value A	p-value B	p-value C	p-value D
GO:0000002	1	1	100	1.13	0	0	0	0	0	0	0	0	0	1	1	1	1
GO:0000004	219	197	89.95	1.02	7	3.2	0.92	11	5.02	1.08	4	1.83	0.52	1	1	1	1
GO:0000005	4	4	100	1.13	0	0	0	0	0	0	0	0	0	1	1	1	1
GO:0000008	38	34	89.47	1.01	1	2.63	0.75	3	7.89	1.7	0	0	0	1	1	1	1
GO:0000009	4	4	100	1.13	0	0	0	0	0	0	0	0	0	1	1	1	1
GO:0000012	3	3	100	1.13	0	0	0	0	0	0	0	0	0	1	1	1	1
GO:0000015	6	6	100	1.13	0	0	0	0	0	0	0	0	0	1	1	1	1
GO:0000022	4	3	75	0.85	1	25	7.16	0	0	0	0	0	0	1	1	1	1
GO:0000026	4	4	100	1.13	0	0	0	0	0	0	0	0	0	1	1	1	1
GO:0000027	1	1	100	1.13	0	0	0	0	0	0	0	0	0	1	1	1	1
GO:0000030	11	11	100	1.13	0	0	0	0	0	0	0	0	0	1	1	1	1
GO:0000033	4	4	100	1.13	0	0	0	0	0	0	0	0	0	1	1	1	1
GO:0000036	3	3	100	1.13	0	0	0	0	0	0	0	0	0	1	1	1	1
GO:0000038	1	1	100	1.13	0	0	0	0	0	0	0	0	0	1	1	1	1
GO:0000042	3	3	100	1.13	0	0	0	0	0	0	0	0	0	1	1	1	1
GO:0000045	3	3	100	1.13	0	0	0	0	0	0	0	0	0	1	1	1	1
GO:0000047	6	6	100	1.13	0	0	0	0	0	0	0	0	0	1	1	1	1
GO:0000049	9	9	100	1.13	0	0	0	0	0	0	0	0	0	1	1	1	1
GO:0000050	4	1	25	0.28	0	0	0	2	50	10.76	1	25	7.06	1	1	1	1
GO:0000059	14	13	92.86	1.05	0	0	0	1	7.14	1.54	0	0	0	1	1	1	1
GO:0000060	5	5	100	1.13	0	0	0	0	0	0	0	0	0	1	1	1	1
GO:0000062	20	20	100	1.13	0	0	0	0	0	0	0	0	0	1	1	1	1
GO:0000067	5	5	100	1.13	0	0	0	0	0	0	0	0	0	1	1	1	1
GO:0000070	3	3	100	1.13	0	0	0	0	0	0	0	0	0	1	1	1	1
GO:0000072	1	1	100	1.13	0	0	0	0	0	0	0	0	0	1	1	1	1
GO:0000074	237	214	90.3	1.02	4	1.69	0.48	15	6.33	1.36	4	1.69	0.48	1	1	1	1
GO:0000075	3	3	100	1.13	0	0	0	0	0	0	0	0	0	1	1	1	1
GO:0000077	7	7	100	1.13	0	0	0	0	0	0	0	0	0	1	1	1	1
GO:0000079	11	11	100	1.13	0	0	0	0	0	0	0	0	0	1	1	1	1
GO:0000080	2	2	100	1.13	0	0	0	0	0	0	0	0	0	1	1	1	1
GO:0000082	41	40	97.56	1.1	0	0	0	0	0	0	1	2.44	0.69	1	1	1	1
GO:0000084	1	1	100	1.13	0	0	0	0	0	0	0	0	0	1	1	1	1
GO:0000085	3	3	100	1.13	0	0	0	0	0	0	0	0	0	1	1	1	1
GO:0000086	4	4	100	1.13	0	0	0	0	0	0	0	0	0	1	1	1	1
GO:0000087	7	7	100	1.13	0	0	0	0	0	0	0	0	0	1	1	1	1
GO:0000094	1	1	100	1.13	0	0	0	0	0	0	0	0	0	1	1	1	1
GO:0000096	2	2	100	1.13	0	0	0	0	0	0	0	0	0	1	1	1	1
GO:0000103	5	4	80	0.91	0	0	0	0	0	0	1	20	5.64	1	1	1	1
GO:0000104	2	2	100	1.13	0	0	0	0	0	0	0	0	0	1	1	1	1
GO:0000106	1	1	100	1.13	0	0	0	0	0	0	0	0	0	1	1	1	1

GO:0000109	3	3	100	1.13	0	0	0	0	0	0	0	0	0	1	1	1	1
GO:0000118	11	11	100	1.13	0	0	0	0	0	0	0	0	0	1	1	1	1
GO:0000119	11	5	45.45	0.51	2	18.18	5.21	1	9.09	1.96	3	27.27	7.7	1	1	1	1
GO:0000120	2	2	100	1.13	0	0	0	0	0	0	0	0	0	1	1	1	1
GO:0000122	53	49	92.45	1.05	2	3.77	1.08	2	3.77	0.81	0	0	0	1	1	1	1
GO:0000123	6	5	83.33	0.94	0	0	0	1	16.67	3.59	0	0	0	1	1	1	1
GO:0000127	2	2	100	1.13	0	0	0	0	0	0	0	0	0	1	1	1	1
GO:0000137	2	2	100	1.13	0	0	0	0	0	0	0	0	0	1	1	1	1
GO:0000139	49	47	95.92	1.09	0	0	0	2	4.08	0.88	0	0	0	1	1	1	1
GO:0000145	7	7	100	1.13	0	0	0	0	0	0	0	0	0	1	1	1	1
GO:0000147	6	6	100	1.13	0	0	0	0	0	0	0	0	0	1	1	1	1
GO:0000149	2	2	100	1.13	0	0	0	0	0	0	0	0	0	1	1	1	1
GO:0000151	33	25	75.76	0.86	2	6.06	1.74	6	18.18	3.91	0	0	0	1	1	1	1
GO:0000152	1	1	100	1.13	0	0	0	0	0	0	0	0	0	1	1	1	1
GO:0000154	9	9	100	1.13	0	0	0	0	0	0	0	0	0	1	1	1	1
GO:0000155	6	6	100	1.13	0	0	0	0	0	0	0	0	0	1	1	1	1
GO:0000157	35	33	94.29	1.07	1	2.86	0.82	0	0	0	1	2.86	0.81	1	1	1	1
GO:0000158	15	14	93.33	1.06	1	6.67	1.91	0	0	0	0	0	0	1	1	1	1
GO:0000159	22	21	95.45	1.08	0	0	0	0	0	0	1	4.55	1.28	1	1	1	1
GO:0000160	58	58	100	1.13	0	0	0	0	0	0	0	0	0	1	1	1	1
GO:0000163	17	17	100	1.13	0	0	0	0	0	0	0	0	0	1	1	1	1
GO:0000164	2	2	100	1.13	0	0	0	0	0	0	0	0	0	1	1	1	1
GO:0000165	23	20	86.96	0.98	2	8.7	2.49	1	4.35	0.94	0	0	0	1	1	1	1
GO:0000166	167	156	93.41	1.06	4	2.4	0.69	5	2.99	0.64	2	1.2	0.34	1	1	1	1
GO:0000171	4	4	100	1.13	0	0	0	0	0	0	0	0	0	1	1	1	1
GO:0000172	6	5	83.33	0.94	1	16.67	4.77	0	0	0	0	0	0	1	1	1	1
GO:0000175	12	12	100	1.13	0	0	0	0	0	0	0	0	0	1	1	1	1
GO:0000178	2	2	100	1.13	0	0	0	0	0	0	0	0	0	1	1	1	1
GO:0000179	7	7	100	1.13	0	0	0	0	0	0	0	0	0	1	1	1	1
GO:0000184	1	1	100	1.13	0	0	0	0	0	0	0	0	0	1	1	1	1
GO:0000186	1	0	0	0	1	100	28.63	0	0	0	0	0	0	1	1	1	1
GO:0000187	5	1	20	0.23	1	20	5.73	0	0	0	3	60	16.93	1	1	1	1
GO:0000188	7	5	71.43	0.81	1	14.29	4.09	0	0	0	1	14.29	4.03	1	1	1	1
GO:0000209	5	5	100	1.13	0	0	0	0	0	0	0	0	0	1	1	1	1
GO:0000211	3	2	66.67	0.75	0	0	0	1	33.33	7.18	0	0	0	1	1	1	1
GO:0000213	1	1	100	1.13	0	0	0	0	0	0	0	0	0	1	1	1	1
GO:0000214	1	1	100	1.13	0	0	0	0	0	0	0	0	0	1	1	1	1
GO:0000219	10	10	100	1.13	0	0	0	0	0	0	0	0	0	1	1	1	1
GO:0000225	1	1	100	1.13	0	0	0	0	0	0	0	0	0	1	1	1	1
GO:0000226	6	6	100	1.13	0	0	0	0	0	0	0	0	0	1	1	1	1
GO:0000228	9	8	88.89	1.01	0	0	0	1	11.11	2.39	0	0	0	1	1	1	1

GO:0000230	4	4	100	1.13	0	0	0	0	0	0	0	0	0	1	1	1	1
GO:0000232	2	2	100	1.13	0	0	0	0	0	0	0	0	0	1	1	1	1
GO:0000242	4	4	100	1.13	0	0	0	0	0	0	0	0	0	1	1	1	1
GO:0000244	2	2	100	1.13	0	0	0	0	0	0	0	0	0	1	1	1	1
GO:0000245	9	7	77.78	0.88	2	22.22	6.36	0	0	0	0	0	0	1	1	1	1
GO:0000247	3	3	100	1.13	0	0	0	0	0	0	0	0	0	1	1	1	1
GO:0000250	1	1	100	1.13	0	0	0	0	0	0	0	0	0	1	1	1	1
GO:0000253	1	1	100	1.13	0	0	0	0	0	0	0	0	0	1	1	1	1
GO:0000254	1	1	100	1.13	0	0	0	0	0	0	0	0	0	1	1	1	1
GO:0000257	1	1	100	1.13	0	0	0	0	0	0	0	0	0	1	1	1	1
GO:0000260	2	2	100	1.13	0	0	0	0	0	0	0	0	0	1	1	1	1
GO:0000263	7	7	100	1.13	0	0	0	0	0	0	0	0	0	1	1	1	1
GO:0000264	2	2	100	1.13	0	0	0	0	0	0	0	0	0	1	1	1	1
GO:0000265	6	3	50	0.57	3	50	14.32	0	0	0	0	0	0	1	1	1	1
GO:0000271	3	3	100	1.13	0	0	0	0	0	0	0	0	0	1	1	1	1
GO:0000278	5	5	100	1.13	0	0	0	0	0	0	0	0	0	1	1	1	1
GO:0000287	95	93	97.89	1.11	1	1.05	0.3	0	0	0	1	1.05	0.3	1	1	1	1
GO:0000299	1	1	100	1.13	0	0	0	0	0	0	0	0	0	1	1	1	1
GO:0000300	3	2	66.67	0.75	0	0	0	1	33.33	7.18	0	0	0	1	1	1	1
GO:0000302	4	4	100	1.13	0	0	0	0	0	0	0	0	0	1	1	1	1
GO:0000307	9	9	100	1.13	0	0	0	0	0	0	0	0	0	1	1	1	1
GO:0000320	4	4	100	1.13	0	0	0	0	0	0	0	0	0	1	1	1	1
GO:0000398	49	47	95.92	1.09	1	2.04	0.58	0	0	0	1	2.04	0.58	1	1	1	1
GO:0000502	6	6	100	1.13	0	0	0	0	0	0	0	0	0	1	1	1	1
GO:0000702	3	1	33.33	0.38	1	33.33	9.54	1	33.33	7.18	0	0	0	1	1	1	1
GO:0000723	5	5	100	1.13	0	0	0	0	0	0	0	0	0	1	1	1	1
GO:0000775	3	3	100	1.13	0	0	0	0	0	0	0	0	0	1	1	1	1
GO:0000781	6	6	100	1.13	0	0	0	0	0	0	0	0	0	1	1	1	1
GO:0000785	30	27	90	1.02	0	0	0	3	10	2.15	0	0	0	1	1	1	1
GO:0000786	19	14	73.68	0.83	0	0	0	3	15.79	3.4	2	10.53	2.97	1	1	1	1
GO:0000790	2	2	100	1.13	0	0	0	0	0	0	0	0	0	1	1	1	1
GO:0000793	15	15	100	1.13	0	0	0	0	0	0	0	0	0	1	1	1	1
GO:0000794	2	2	100	1.13	0	0	0	0	0	0	0	0	0	1	1	1	1
GO:0000795	2	2	100	1.13	0	0	0	0	0	0	0	0	0	1	1	1	1
GO:0000800	1	1	100	1.13	0	0	0	0	0	0	0	0	0	1	1	1	1
GO:0000808	1	1	100	1.13	0	0	0	0	0	0	0	0	0	1	1	1	1
GO:0000902	16	15	93.75	1.06	0	0	0	1	6.25	1.35	0	0	0	1	1	1	1
GO:0000904	4	1	25	0.28	0	0	0	3	75	16.15	0	0	0	1	1	1	1
GO:0000910	102	100	98.04	1.11	0	0	0	2	1.96	0.42	0	0	0	0.491473811	1	1	1
GO:0001501	44	38	86.36	0.98	1	2.27	0.65	4	9.09	1.96	1	2.27	0.64	1	1	1	1
GO:0001502	8	7	87.5	0.99	0	0	0	1	12.5	2.69	0	0	0	1	1	1	1

GO:0001503	33	28	84.85	0.96	0	0	0	3	9.09	1.96	2	6.06	1.71	1	1	1	1
GO:0001504	4	3	75	0.85	0	0	0	1	25	5.38	0	0	0	1	1	1	1
GO:0001506	2	2	100	1.13	0	0	0	0	0	0	0	0	0	1	1	1	1
GO:0001508	1	1	100	1.13	0	0	0	0	0	0	0	0	0	1	1	1	1
GO:0001509	6	6	100	1.13	0	0	0	0	0	0	0	0	0	1	1	1	1
GO:0001510	1	1	100	1.13	0	0	0	0	0	0	0	0	0	1	1	1	1
GO:0001516	10	9	90	1.02	0	0	0	0	0	0	1	10	2.82	1	1	1	1
GO:0001517	1	1	100	1.13	0	0	0	0	0	0	0	0	0	1	1	1	1
GO:0001523	6	4	66.67	0.75	1	16.67	4.77	1	16.67	3.59	0	0	0	1	1	1	1
GO:0001524	11	2	18.18	0.21	8	72.73	20.82	0	0	0	1	9.09	2.57	1	1.74E-06	1	1
GO:0001525	30	25	83.33	0.94	3	10	2.86	2	6.67	1.44	0	0	0	1	1	1	1
GO:0001527	5	4	80	0.91	0	0	0	1	20	4.31	0	0	0	1	1	1	1
GO:0001533	7	1	14.29	0.16	2	28.57	8.18	1	14.29	3.08	3	42.86	12.1	1	1	1	1
GO:0001537	2	2	100	1.13	0	0	0	0	0	0	0	0	0	1	1	1	1
GO:0001540	2	2	100	1.13	0	0	0	0	0	0	0	0	0	1	1	1	1
GO:0001542	4	4	100	1.13	0	0	0	0	0	0	0	0	0	1	1	1	1
GO:0001544	1	1	100	1.13	0	0	0	0	0	0	0	0	0	1	1	1	1
GO:0001556	1	1	100	1.13	0	0	0	0	0	0	0	0	0	1	1	1	1
GO:0001558	41	38	92.68	1.05	2	4.88	1.4	0	0	0	1	2.44	0.69	1	1	1	1
GO:0001560	1	1	100	1.13	0	0	0	0	0	0	0	0	0	1	1	1	1
GO:0001561	5	5	100	1.13	0	0	0	0	0	0	0	0	0	1	1	1	1
GO:0001568	9	7	77.78	0.88	0	0	0	1	11.11	2.39	1	11.11	3.14	1	1	1	1
GO:0001569	2	2	100	1.13	0	0	0	0	0	0	0	0	0	1	1	1	1
GO:0001570	2	2	100	1.13	0	0	0	0	0	0	0	0	0	1	1	1	1
GO:0001578	1	1	100	1.13	0	0	0	0	0	0	0	0	0	1	1	1	1
GO:0001584	71	53	74.65	0.85	7	9.86	2.82	5	7.04	1.52	6	8.45	2.39	1	1	1	1
GO:0001601	1	0	0	0	1	100	28.63	0	0	0	0	0	0	1	1	1	1
GO:0001602	1	0	0	0	1	100	28.63	0	0	0	0	0	0	1	1	1	1
GO:0001614	2	0	0	0	0	0	0	2	100	21.53	0	0	0	1	1	1	1
GO:0001619	7	7	100	1.13	0	0	0	0	0	0	0	0	0	1	1	1	1
GO:0001650	3	3	100	1.13	0	0	0	0	0	0	0	0	0	1	1	1	1
GO:0001651	1	1	100	1.13	0	0	0	0	0	0	0	0	0	1	1	1	1
GO:0001654	2	1	50	0.57	0	0	0	0	0	0	1	50	14.11	1	1	1	1
GO:0001655	2	2	100	1.13	0	0	0	0	0	0	0	0	0	1	1	1	1
GO:0001656	3	3	100	1.13	0	0	0	0	0	0	0	0	0	1	1	1	1
GO:0001657	3	3	100	1.13	0	0	0	0	0	0	0	0	0	1	1	1	1
GO:0001658	1	1	100	1.13	0	0	0	0	0	0	0	0	0	1	1	1	1
GO:0001662	15	14	93.33	1.06	0	0	0	1	6.67	1.44	0	0	0	1	1	1	1
GO:0001664	2	2	100	1.13	0	0	0	0	0	0	0	0	0	1	1	1	1
GO:0001666	4	4	100	1.13	0	0	0	0	0	0	0	0	0	1	1	1	1
GO:0001667	2	2	100	1.13	0	0	0	0	0	0	0	0	0	1	1	1	1

GO:0001668	1	1	100	1.13	0	0	0	0	0	0	0	0	0	1	1	1	1
GO:0001669	1	1	100	1.13	0	0	0	0	0	0	0	0	0	1	1	1	1
GO:0001671	3	3	100	1.13	0	0	0	0	0	0	0	0	0	1	1	1	1
GO:0001674	1	1	100	1.13	0	0	0	0	0	0	0	0	0	1	1	1	1
GO:0001675	10	10	100	1.13	0	0	0	0	0	0	0	0	0	1	1	1	1
GO:0001701	8	5	62.5	0.71	0	0	0	3	37.5	8.07	0	0	0	1	1	1	1
GO:0001707	8	8	100	1.13	0	0	0	0	0	0	0	0	0	1	1	1	1
GO:0001709	4	0	0	0	2	50	14.32	2	50	10.76	0	0	0	1	1	1	1
GO:0001719	1	1	100	1.13	0	0	0	0	0	0	0	0	0	1	1	1	1
GO:0001725	4	2	50	0.57	1	25	7.16	0	0	0	1	25	7.06	1	1	1	1
GO:0001726	17	16	94.12	1.07	0	0	0	1	5.88	1.27	0	0	0	1	1	1	1
GO:0001729	1	1	100	1.13	0	0	0	0	0	0	0	0	0	1	1	1	1
GO:0001730	1	0	0	0	0	0	0	1	100	21.53	0	0	0	1	1	1	1
GO:0001734	1	1	100	1.13	0	0	0	0	0	0	0	0	0	1	1	1	1
GO:0001735	3	3	100	1.13	0	0	0	0	0	0	0	0	0	1	1	1	1
GO:0001756	3	3	100	1.13	0	0	0	0	0	0	0	0	0	1	1	1	1
GO:0001757	1	0	0	0	1	100	28.63	0	0	0	0	0	0	1	1	1	1
GO:0001763	2	2	100	1.13	0	0	0	0	0	0	0	0	0	1	1	1	1
GO:0001764	2	2	100	1.13	0	0	0	0	0	0	0	0	0	1	1	1	1
GO:0001779	1	1	100	1.13	0	0	0	0	0	0	0	0	0	1	1	1	1
GO:0001781	1	0	0	0	0	0	0	1	100	21.53	0	0	0	1	1	1	1
GO:0001782	7	6	85.71	0.97	0	0	0	1	14.29	3.08	0	0	0	1	1	1	1
GO:0001784	2	2	100	1.13	0	0	0	0	0	0	0	0	0	1	1	1	1
GO:0001786	3	3	100	1.13	0	0	0	0	0	0	0	0	0	1	1	1	1
GO:0001788	3	0	0	0	0	0	0	0	0	0	3	100	28.22	1	1	1	0.21002992
GO:0001798	4	1	25	0.28	0	0	0	0	0	0	3	75	21.17	1	1	1	0.815577835
GO:0001805	4	1	25	0.28	0	0	0	0	0	0	3	75	21.17	1	1	1	0.815577835
GO:0001811	3	0	0	0	0	0	0	3	100	21.53	0	0	0	1	1	0.43202791	1
GO:0001812	3	0	0	0	0	0	0	0	0	0	3	100	28.22	1	1	1	0.21002992
GO:0001820	3	0	0	0	0	0	0	0	0	0	3	100	28.22	1	1	1	0.21002992
GO:0001836	1	1	100	1.13	0	0	0	0	0	0	0	0	0	1	1	1	1
GO:0001837	3	2	66.67	0.75	0	0	0	0	0	0	1	33.33	9.41	1	1	1	1
GO:0001843	1	1	100	1.13	0	0	0	0	0	0	0	0	0	1	1	1	1
GO:0001844	1	1	100	1.13	0	0	0	0	0	0	0	0	0	1	1	1	1
GO:0002009	1	0	0	0	0	0	0	0	0	0	1	100	28.22	1	1	1	1
GO:0002011	5	5	100	1.13	0	0	0	0	0	0	0	0	0	1	1	1	1
GO:0003674	1	1	100	1.13	0	0	0	0	0	0	0	0	0	1	1	1	1
GO:0003676	1030	913	88.64	1	56	5.44	1.56	43	4.17	0.9	18	1.75	0.49	1	1	1	1
GO:0003677	1730	1571	90.81	1.03	59	3.41	0.98	66	3.82	0.82	34	1.97	0.55	0.016366804	1	1	1
GO:0003678	73	68	93.15	1.05	4	5.48	1.57	0	0	0	1	1.37	0.39	1	1	1	1
GO:0003682	58	51	87.93	1	3	5.17	1.48	4	6.9	1.48	0	0	0	1	1	1	1

GO:0003683	1	1	100	1.13	0	0	0	0	0	0	0	0	0	1	1	1	1
GO:0003684	16	16	100	1.13	0	0	0	0	0	0	0	0	0	1	1	1	1
GO:0003685	24	24	100	1.13	0	0	0	0	0	0	0	0	0	1	1	1	1
GO:0003686	3	3	100	1.13	0	0	0	0	0	0	0	0	0	1	1	1	1
GO:0003687	7	7	100	1.13	0	0	0	0	0	0	0	0	0	1	1	1	1
GO:0003688	3	3	100	1.13	0	0	0	0	0	0	0	0	0	1	1	1	1
GO:0003690	24	20	83.33	0.94	4	16.67	4.77	0	0	0	0	0	0	1	1	1	1
GO:0003697	26	20	76.92	0.87	4	15.38	4.41	2	7.69	1.66	0	0	0	1	1	1	1
GO:0003700	744	649	87.23	0.99	33	4.44	1.27	40	5.38	1.16	22	2.96	0.83	1	1	1	1
GO:0003701	1	1	100	1.13	0	0	0	0	0	0	0	0	0	1	1	1	1
GO:0003702	87	82	94.25	1.07	4	4.6	1.32	0	0	0	1	1.15	0.32	1	1	1	1
GO:0003704	12	9	75	0.85	1	8.33	2.39	2	16.67	3.59	0	0	0	1	1	1	1
GO:0003705	7	7	100	1.13	0	0	0	0	0	0	0	0	0	1	1	1	1
GO:0003707	38	31	81.58	0.92	2	5.26	1.51	2	5.26	1.13	3	7.89	2.23	1	1	1	1
GO:0003708	3	3	100	1.13	0	0	0	0	0	0	0	0	0	1	1	1	1
GO:0003709	5	3	60	0.68	1	20	5.73	1	20	4.31	0	0	0	1	1	1	1
GO:0003711	6	6	100	1.13	0	0	0	0	0	0	0	0	0	1	1	1	1
GO:0003712	21	20	95.24	1.08	1	4.76	1.36	0	0	0	0	0	0	1	1	1	1
GO:0003713	93	84	90.32	1.02	5	5.38	1.54	3	3.23	0.69	1	1.08	0.3	1	1	1	1
GO:0003714	66	56	84.85	0.96	5	7.58	2.17	3	4.55	0.98	2	3.03	0.86	1	1	1	1
GO:0003716	1	1	100	1.13	0	0	0	0	0	0	0	0	0	1	1	1	1
GO:0003717	1	1	100	1.13	0	0	0	0	0	0	0	0	0	1	1	1	1
GO:0003719	5	5	100	1.13	0	0	0	0	0	0	0	0	0	1	1	1	1
GO:0003721	1	1	100	1.13	0	0	0	0	0	0	0	0	0	1	1	1	1
GO:0003723	645	598	92.71	1.05	30	4.65	1.33	9	1.4	0.3	8	1.24	0.35	0.033847102	1	1	1
GO:0003724	30	28	93.33	1.06	0	0	0	0	0	0	2	6.67	1.88	1	1	1	1
GO:0003725	29	27	93.1	1.05	0	0	0	1	3.45	0.74	1	3.45	0.97	1	1	1	1
GO:0003726	3	3	100	1.13	0	0	0	0	0	0	0	0	0	1	1	1	1
GO:0003727	11	10	90.91	1.03	0	0	0	0	0	0	1	9.09	2.57	1	1	1	1
GO:0003729	11	10	90.91	1.03	1	9.09	2.6	0	0	0	0	0	0	1	1	1	1
GO:0003730	6	5	83.33	0.94	0	0	0	0	0	0	1	16.67	4.7	1	1	1	1
GO:0003731	5	5	100	1.13	0	0	0	0	0	0	0	0	0	1	1	1	1
GO:0003732	2	2	100	1.13	0	0	0	0	0	0	0	0	0	1	1	1	1
GO:0003733	57	57	100	1.13	0	0	0	0	0	0	0	0	0	1	1	1	1
GO:0003734	42	41	97.62	1.11	0	0	0	1	2.38	0.51	0	0	0	1	1	1	1
GO:0003735	343	306	89.21	1.01	10	2.92	0.83	24	7	1.51	3	0.87	0.25	1	1	1	1
GO:0003743	146	137	93.84	1.06	2	1.37	0.39	5	3.42	0.74	2	1.37	0.39	1	1	1	1
GO:0003746	57	56	98.25	1.11	0	0	0	1	1.75	0.38	0	0	0	1	1	1	1
GO:0003747	10	8	80	0.91	0	0	0	1	10	2.15	1	10	2.82	1	1	1	1
GO:0003750	70	66	94.29	1.07	1	1.43	0.41	1	1.43	0.31	2	2.86	0.81	1	1	1	1
GO:0003751	23	23	100	1.13	0	0	0	0	0	0	0	0	0	1	1	1	1

GO:0003752	3	3	100	1.13	0	0	0	0	0	0	0	0	0	1	1	1	1
GO:0003754	257	238	92.61	1.05	3	1.17	0.33	13	5.06	1.09	3	1.17	0.33	1	1	1	1
GO:0003755	64	61	95.31	1.08	0	0	0	1	1.56	0.34	2	3.13	0.88	1	1	1	1
GO:0003756	10	10	100	1.13	0	0	0	0	0	0	0	0	0	1	1	1	1
GO:0003762	1	1	100	1.13	0	0	0	0	0	0	0	0	0	1	1	1	1
GO:0003763	1	1	100	1.13	0	0	0	0	0	0	0	0	0	1	1	1	1
GO:0003767	15	15	100	1.13	0	0	0	0	0	0	0	0	0	1	1	1	1
GO:0003772	7	7	100	1.13	0	0	0	0	0	0	0	0	0	1	1	1	1
GO:0003773	70	65	92.86	1.05	3	4.29	1.23	1	1.43	0.31	1	1.43	0.4	1	1	1	1
GO:0003774	135	130	96.3	1.09	3	2.22	0.64	2	1.48	0.32	0	0	0	1	1	1	1
GO:0003777	39	38	97.44	1.1	1	2.56	0.73	0	0	0	0	0	0	1	1	1	1
GO:0003778	3	3	100	1.13	0	0	0	0	0	0	0	0	0	1	1	1	1
GO:0003779	255	232	90.98	1.03	6	2.35	0.67	14	5.49	1.18	3	1.18	0.33	1	1	1	1
GO:0003780	1	1	100	1.13	0	0	0	0	0	0	0	0	0	1	1	1	1
GO:0003782	9	8	88.89	1.01	0	0	0	0	0	0	1	11.11	3.14	1	1	1	1
GO:0003784	1	1	100	1.13	0	0	0	0	0	0	0	0	0	1	1	1	1
GO:0003789	2	2	100	1.13	0	0	0	0	0	0	0	0	0	1	1	1	1
GO:0003790	5	5	100	1.13	0	0	0	0	0	0	0	0	0	1	1	1	1
GO:0003791	1	1	100	1.13	0	0	0	0	0	0	0	0	0	1	1	1	1
GO:0003792	1	0	0	0	1	100	28.63	0	0	0	0	0	0	1	1	1	1
GO:0003793	22	13	59.09	0.67	2	9.09	2.6	5	22.73	4.89	2	9.09	2.57	1	1	1	1
GO:0003794	1	0	0	0	1	100	28.63	0	0	0	0	0	0	1	1	1	1
GO:0003795	2	0	0	0	0	0	0	1	50	10.76	1	50	14.11	1	1	1	1
GO:0003796	1	1	100	1.13	0	0	0	0	0	0	0	0	0	1	1	1	1
GO:0003797	4	1	25	0.28	0	0	0	0	0	0	3	75	21.17	1	1	1	0.815577835
GO:0003800	5	4	80	0.91	0	0	0	1	20	4.31	0	0	0	1	1	1	1
GO:0003802	4	4	100	1.13	0	0	0	0	0	0	0	0	0	1	1	1	1
GO:0003803	1	0	0	0	0	0	0	0	0	0	1	100	28.22	1	1	1	1
GO:0003805	1	0	0	0	0	0	0	0	0	0	1	100	28.22	1	1	1	1
GO:0003807	1	1	100	1.13	0	0	0	0	0	0	0	0	0	1	1	1	1
GO:0003809	5	3	60	0.68	0	0	0	1	20	4.31	1	20	5.64	1	1	1	1
GO:0003810	9	8	88.89	1.01	0	0	0	0	0	0	1	11.11	3.14	1	1	1	1
GO:0003811	10	3	30	0.34	1	10	2.86	0	0	0	6	60	16.93	1	1	1	0.002271866
GO:0003812	3	3	100	1.13	0	0	0	0	0	0	0	0	0	1	1	1	1
GO:0003813	1	1	100	1.13	0	0	0	0	0	0	0	0	0	1	1	1	1
GO:0003817	1	1	100	1.13	0	0	0	0	0	0	0	0	0	1	1	1	1
GO:0003819	11	8	72.73	0.82	1	9.09	2.6	2	18.18	3.91	0	0	0	1	1	1	1
GO:0003820	4	4	100	1.13	0	0	0	0	0	0	0	0	0	1	1	1	1
GO:0003821	3	1	33.33	0.38	0	0	0	2	66.67	14.35	0	0	0	1	1	1	1
GO:0003822	4	3	75	0.85	1	25	7.16	0	0	0	0	0	0	1	1	1	1
GO:0003823	23	11	47.83	0.54	2	8.7	2.49	6	26.09	5.62	4	17.39	4.91	1	1	1	1

GO:0003824	637	583	91.52	1.04	8	1.26	0.36	28	4.4	0.95	18	2.83	0.8	1	1	1	1
GO:0003826	1	1	100	1.13	0	0	0	0	0	0	0	0	0	1	1	1	1
GO:0003827	3	2	66.67	0.75	0	0	0	1	33.33	7.18	0	0	0	1	1	1	1
GO:0003828	5	5	100	1.13	0	0	0	0	0	0	0	0	0	1	1	1	1
GO:0003829	1	1	100	1.13	0	0	0	0	0	0	0	0	0	1	1	1	1
GO:0003830	1	1	100	1.13	0	0	0	0	0	0	0	0	0	1	1	1	1
GO:0003834	1	0	0	0	0	0	0	1	100	21.53	0	0	0	1	1	1	1
GO:0003835	1	1	100	1.13	0	0	0	0	0	0	0	0	0	1	1	1	1
GO:0003836	3	3	100	1.13	0	0	0	0	0	0	0	0	0	1	1	1	1
GO:0003840	2	2	100	1.13	0	0	0	0	0	0	0	0	0	1	1	1	1
GO:0003841	7	6	85.71	0.97	1	14.29	4.09	0	0	0	0	0	0	1	1	1	1
GO:0003842	1	1	100	1.13	0	0	0	0	0	0	0	0	0	1	1	1	1
GO:0003843	1	1	100	1.13	0	0	0	0	0	0	0	0	0	1	1	1	1
GO:0003844	4	4	100	1.13	0	0	0	0	0	0	0	0	0	1	1	1	1
GO:0003845	2	2	100	1.13	0	0	0	0	0	0	0	0	0	1	1	1	1
GO:0003847	17	15	88.24	1	0	0	0	0	0	0	2	11.76	3.32	1	1	1	1
GO:0003851	2	2	100	1.13	0	0	0	0	0	0	0	0	0	1	1	1	1
GO:0003854	3	0	0	0	0	0	0	2	66.67	14.35	1	33.33	9.41	1	1	1	1
GO:0003855	5	5	100	1.13	0	0	0	0	0	0	0	0	0	1	1	1	1
GO:0003857	9	9	100	1.13	0	0	0	0	0	0	0	0	0	1	1	1	1
GO:0003863	2	0	0	0	0	0	0	2	100	21.53	0	0	0	1	1	1	1
GO:0003868	1	1	100	1.13	0	0	0	0	0	0	0	0	0	1	1	1	1
GO:0003870	3	2	66.67	0.75	1	33.33	9.54	0	0	0	0	0	0	1	1	1	1
GO:0003872	9	9	100	1.13	0	0	0	0	0	0	0	0	0	1	1	1	1
GO:0003873	1	1	100	1.13	0	0	0	0	0	0	0	0	0	1	1	1	1
GO:0003874	1	1	100	1.13	0	0	0	0	0	0	0	0	0	1	1	1	1
GO:0003875	3	3	100	1.13	0	0	0	0	0	0	0	0	0	1	1	1	1
GO:0003876	2	1	50	0.57	0	0	0	0	0	0	1	50	14.11	1	1	1	1
GO:0003878	3	3	100	1.13	0	0	0	0	0	0	0	0	0	1	1	1	1
GO:0003880	5	5	100	1.13	0	0	0	0	0	0	0	0	0	1	1	1	1
GO:0003881	1	0	0	0	1	100	28.63	0	0	0	0	0	0	1	1	1	1
GO:0003882	1	1	100	1.13	0	0	0	0	0	0	0	0	0	1	1	1	1
GO:0003883	9	9	100	1.13	0	0	0	0	0	0	0	0	0	1	1	1	1
GO:0003886	5	5	100	1.13	0	0	0	0	0	0	0	0	0	1	1	1	1
GO:0003887	24	24	100	1.13	0	0	0	0	0	0	0	0	0	1	1	1	1
GO:0003889	2	2	100	1.13	0	0	0	0	0	0	0	0	0	1	1	1	1
GO:0003890	4	4	100	1.13	0	0	0	0	0	0	0	0	0	1	1	1	1
GO:0003891	7	7	100	1.13	0	0	0	0	0	0	0	0	0	1	1	1	1
GO:0003893	2	2	100	1.13	0	0	0	0	0	0	0	0	0	1	1	1	1
GO:0003894	2	2	100	1.13	0	0	0	0	0	0	0	0	0	1	1	1	1
GO:0003895	5	5	100	1.13	0	0	0	0	0	0	0	0	0	1	1	1	1

GO:0003896	4	4	100	1.13	0	0	0	0	0	0	0	0	0	1	1	1	1
GO:0003899	53	51	96.23	1.09	2	3.77	1.08	0	0	0	0	0	0	1	1	1	1
GO:0003900	18	17	94.44	1.07	1	5.56	1.59	0	0	0	0	0	0	1	1	1	1
GO:0003901	20	19	95	1.08	1	5	1.43	0	0	0	0	0	0	1	1	1	1
GO:0003902	26	25	96.15	1.09	1	3.85	1.1	0	0	0	0	0	0	1	1	1	1
GO:0003905	2	2	100	1.13	0	0	0	0	0	0	0	0	0	1	1	1	1
GO:0003906	1	1	100	1.13	0	0	0	0	0	0	0	0	0	1	1	1	1
GO:0003907	2	1	50	0.57	1	50	14.32	0	0	0	0	0	0	1	1	1	1
GO:0003908	4	3	75	0.85	0	0	0	1	25	5.38	0	0	0	1	1	1	1
GO:0003910	3	3	100	1.13	0	0	0	0	0	0	0	0	0	1	1	1	1
GO:0003916	10	10	100	1.13	0	0	0	0	0	0	0	0	0	1	1	1	1
GO:0003917	7	7	100	1.13	0	0	0	0	0	0	0	0	0	1	1	1	1
GO:0003918	6	6	100	1.13	0	0	0	0	0	0	0	0	0	1	1	1	1
GO:0003920	3	3	100	1.13	0	0	0	0	0	0	0	0	0	1	1	1	1
GO:0003921	7	7	100	1.13	0	0	0	0	0	0	0	0	0	1	1	1	1
GO:0003922	7	7	100	1.13	0	0	0	0	0	0	0	0	0	1	1	1	1
GO:0003923	2	1	50	0.57	0	0	0	0	0	0	1	50	14.11	1	1	1	1
GO:0003924	215	205	95.35	1.08	5	2.33	0.67	4	1.86	0.4	1	0.47	0.13	0.268122438	1	1	1
GO:0003925	280	271	96.79	1.1	5	1.79	0.51	3	1.07	0.23	1	0.36	0.1	0.000139686	1	1	1
GO:0003926	2	2	100	1.13	0	0	0	0	0	0	0	0	0	1	1	1	1
GO:0003927	30	27	90	1.02	3	10	2.86	0	0	0	0	0	0	1	1	1	1
GO:0003928	227	217	95.59	1.08	5	2.2	0.63	4	1.76	0.38	1	0.44	0.12	0.09066663	1	1	1
GO:0003929	2	2	100	1.13	0	0	0	0	0	0	0	0	0	1	1	1	1
GO:0003930	169	160	94.67	1.07	4	2.37	0.68	4	2.37	0.51	1	0.59	0.17	1	1	1	1
GO:0003931	160	152	95	1.08	4	2.5	0.72	3	1.88	0.4	1	0.63	0.18	1	1	1	1
GO:0003934	1	1	100	1.13	0	0	0	0	0	0	0	0	0	1	1	1	1
GO:0003936	45	43	95.56	1.08	0	0	0	2	4.44	0.96	0	0	0	1	1	1	1
GO:0003937	4	4	100	1.13	0	0	0	0	0	0	0	0	0	1	1	1	1
GO:0003938	6	6	100	1.13	0	0	0	0	0	0	0	0	0	1	1	1	1
GO:0003939	4	4	100	1.13	0	0	0	0	0	0	0	0	0	1	1	1	1
GO:0003940	3	3	100	1.13	0	0	0	0	0	0	0	0	0	1	1	1	1
GO:0003943	3	3	100	1.13	0	0	0	0	0	0	0	0	0	1	1	1	1
GO:0003944	1	1	100	1.13	0	0	0	0	0	0	0	0	0	1	1	1	1
GO:0003945	4	4	100	1.13	0	0	0	0	0	0	0	0	0	1	1	1	1
GO:0003947	1	1	100	1.13	0	0	0	0	0	0	0	0	0	1	1	1	1
GO:0003948	1	1	100	1.13	0	0	0	0	0	0	0	0	0	1	1	1	1
GO:0003950	11	9	81.82	0.93	1	9.09	2.6	1	9.09	1.96	0	0	0	1	1	1	1
GO:0003951	4	4	100	1.13	0	0	0	0	0	0	0	0	0	1	1	1	1
GO:0003952	1	1	100	1.13	0	0	0	0	0	0	0	0	0	1	1	1	1
GO:0003953	6	4	66.67	0.75	0	0	0	2	33.33	7.18	0	0	0	1	1	1	1
GO:0003954	31	28	90.32	1.02	1	3.23	0.92	1	3.23	0.69	1	3.23	0.91	1	1	1	1

GO:0003955	4	4	100	1.13	0	0	0	0	0	0	0	0	0	1	1	1	1
GO:0003956	1	1	100	1.13	0	0	0	0	0	0	0	0	0	1	1	1	1
GO:0003957	6	6	100	1.13	0	0	0	0	0	0	0	0	0	1	1	1	1
GO:0003958	1	1	100	1.13	0	0	0	0	0	0	0	0	0	1	1	1	1
GO:0003960	10	10	100	1.13	0	0	0	0	0	0	0	0	0	1	1	1	1
GO:0003963	6	6	100	1.13	0	0	0	0	0	0	0	0	0	1	1	1	1
GO:0003964	1	1	100	1.13	0	0	0	0	0	0	0	0	0	1	1	1	1
GO:0003969	1	1	100	1.13	0	0	0	0	0	0	0	0	0	1	1	1	1
GO:0003973	2	0	0	0	0	0	0	0	0	0	2	100	28.22	1	1	1	1
GO:0003975	1	1	100	1.13	0	0	0	0	0	0	0	0	0	1	1	1	1
GO:0003977	3	3	100	1.13	0	0	0	0	0	0	0	0	0	1	1	1	1
GO:0003978	2	2	100	1.13	0	0	0	0	0	0	0	0	0	1	1	1	1
GO:0003979	6	6	100	1.13	0	0	0	0	0	0	0	0	0	1	1	1	1
GO:0003980	4	4	100	1.13	0	0	0	0	0	0	0	0	0	1	1	1	1
GO:0003981	3	0	0	0	0	0	0	1	33.33	7.18	2	66.67	18.82	1	1	1	1
GO:0003982	2	0	0	0	1	50	14.32	1	50	10.76	0	0	0	1	1	1	1
GO:0003983	1	1	100	1.13	0	0	0	0	0	0	0	0	0	1	1	1	1
GO:0003985	7	7	100	1.13	0	0	0	0	0	0	0	0	0	1	1	1	1
GO:0003987	6	5	83.33	0.94	1	16.67	4.77	0	0	0	0	0	0	1	1	1	1
GO:0003988	5	5	100	1.13	0	0	0	0	0	0	0	0	0	1	1	1	1
GO:0003993	25	25	100	1.13	0	0	0	0	0	0	0	0	0	1	1	1	1
GO:0003994	4	4	100	1.13	0	0	0	0	0	0	0	0	0	1	1	1	1
GO:0003995	27	27	100	1.13	0	0	0	0	0	0	0	0	0	1	1	1	1
GO:0003996	1	0	0	0	0	0	0	1	100	21.53	0	0	0	1	1	1	1
GO:0003997	4	4	100	1.13	0	0	0	0	0	0	0	0	0	1	1	1	1
GO:0003998	7	7	100	1.13	0	0	0	0	0	0	0	0	0	1	1	1	1
GO:0003999	1	1	100	1.13	0	0	0	0	0	0	0	0	0	1	1	1	1
GO:0004000	2	2	100	1.13	0	0	0	0	0	0	0	0	0	1	1	1	1
GO:0004001	2	2	100	1.13	0	0	0	0	0	0	0	0	0	1	1	1	1
GO:0004002	46	46	100	1.13	0	0	0	0	0	0	0	0	0	1	1	1	1
GO:0004003	18	17	94.44	1.07	0	0	0	1	5.56	1.2	0	0	0	1	1	1	1
GO:0004004	12	12	100	1.13	0	0	0	0	0	0	0	0	0	1	1	1	1
GO:0004005	12	10	83.33	0.94	0	0	0	0	0	0	2	16.67	4.7	1	1	1	1
GO:0004008	2	2	100	1.13	0	0	0	0	0	0	0	0	0	1	1	1	1
GO:0004009	62	55	88.71	1	2	3.23	0.92	3	4.84	1.04	2	3.23	0.91	1	1	1	1
GO:0004012	5	4	80	0.91	0	0	0	0	0	0	1	20	5.64	1	1	1	1
GO:0004013	1	1	100	1.13	0	0	0	0	0	0	0	0	0	1	1	1	1
GO:0004016	5	5	100	1.13	0	0	0	0	0	0	0	0	0	1	1	1	1
GO:0004017	11	11	100	1.13	0	0	0	0	0	0	0	0	0	1	1	1	1
GO:0004018	3	3	100	1.13	0	0	0	0	0	0	0	0	0	1	1	1	1
GO:0004019	8	8	100	1.13	0	0	0	0	0	0	0	0	0	1	1	1	1

GO:0004021	1	1	100	1.13	0	0	0	0	0	0	0	0	0	1	1	1	1
GO:0004022	3	1	33.33	0.38	1	33.33	9.54	1	33.33	7.18	0	0	0	1	1	1	1
GO:0004023	2	2	100	1.13	0	0	0	0	0	0	0	0	0	1	1	1	1
GO:0004024	37	34	91.89	1.04	2	5.41	1.55	1	2.7	0.58	0	0	0	1	1	1	1
GO:0004025	2	2	100	1.13	0	0	0	0	0	0	0	0	0	1	1	1	1
GO:0004027	2	0	0	0	0	0	0	0	0	0	2	100	28.22	1	1	1	1
GO:0004028	2	1	50	0.57	1	50	14.32	0	0	0	0	0	0	1	1	1	1
GO:0004029	15	14	93.33	1.06	1	6.67	1.91	0	0	0	0	0	0	1	1	1	1
GO:0004030	1	0	0	0	1	100	28.63	0	0	0	0	0	0	1	1	1	1
GO:0004031	3	1	33.33	0.38	1	33.33	9.54	1	33.33	7.18	0	0	0	1	1	1	1
GO:0004032	20	19	95	1.08	1	5	1.43	0	0	0	0	0	0	1	1	1	1
GO:0004033	8	5	62.5	0.71	0	0	0	0	0	0	3	37.5	10.58	1	1	1	1
GO:0004034	1	0	0	0	0	0	0	0	0	0	1	100	28.22	1	1	1	1
GO:0004035	3	3	100	1.13	0	0	0	0	0	0	0	0	0	1	1	1	1
GO:0004040	3	3	100	1.13	0	0	0	0	0	0	0	0	0	1	1	1	1
GO:0004041	5	3	60	0.68	2	40	11.45	0	0	0	0	0	0	1	1	1	1
GO:0004042	1	1	100	1.13	0	0	0	0	0	0	0	0	0	1	1	1	1
GO:0004044	4	4	100	1.13	0	0	0	0	0	0	0	0	0	1	1	1	1
GO:0004045	1	1	100	1.13	0	0	0	0	0	0	0	0	0	1	1	1	1
GO:0004046	2	1	50	0.57	0	0	0	0	0	0	1	50	14.11	1	1	1	1
GO:0004047	1	1	100	1.13	0	0	0	0	0	0	0	0	0	1	1	1	1
GO:0004049	7	7	100	1.13	0	0	0	0	0	0	0	0	0	1	1	1	1
GO:0004050	10	9	90	1.02	1	10	2.86	0	0	0	0	0	0	1	1	1	1
GO:0004051	2	2	100	1.13	0	0	0	0	0	0	0	0	0	1	1	1	1
GO:0004052	4	4	100	1.13	0	0	0	0	0	0	0	0	0	1	1	1	1
GO:0004053	2	0	0	0	0	0	0	1	50	10.76	1	50	14.11	1	1	1	1
GO:0004055	1	1	100	1.13	0	0	0	0	0	0	0	0	0	1	1	1	1
GO:0004056	3	3	100	1.13	0	0	0	0	0	0	0	0	0	1	1	1	1
GO:0004057	3	3	100	1.13	0	0	0	0	0	0	0	0	0	1	1	1	1
GO:0004060	1	0	0	0	0	0	0	1	100	21.53	0	0	0	1	1	1	1
GO:0004062	2	0	0	0	0	0	0	1	50	10.76	1	50	14.11	1	1	1	1
GO:0004064	4	4	100	1.13	0	0	0	0	0	0	0	0	0	1	1	1	1
GO:0004065	3	3	100	1.13	0	0	0	0	0	0	0	0	0	1	1	1	1
GO:0004066	4	4	100	1.13	0	0	0	0	0	0	0	0	0	1	1	1	1
GO:0004067	4	4	100	1.13	0	0	0	0	0	0	0	0	0	1	1	1	1
GO:0004069	8	8	100	1.13	0	0	0	0	0	0	0	0	0	1	1	1	1
GO:0004074	3	3	100	1.13	0	0	0	0	0	0	0	0	0	1	1	1	1
GO:0004075	1	1	100	1.13	0	0	0	0	0	0	0	0	0	1	1	1	1
GO:0004077	1	1	100	1.13	0	0	0	0	0	0	0	0	0	1	1	1	1
GO:0004081	2	2	100	1.13	0	0	0	0	0	0	0	0	0	1	1	1	1
GO:0004082	4	2	50	0.57	0	0	0	2	50	10.76	0	0	0	1	1	1	1

GO:0004083	1	1	100	1.13	0	0	0	0	0	0	0	0	0	1	1	1	1
GO:0004084	5	5	100	1.13	0	0	0	0	0	0	0	0	0	1	1	1	1
GO:0004085	3	3	100	1.13	0	0	0	0	0	0	0	0	0	1	1	1	1
GO:0004089	18	16	88.89	1.01	1	5.56	1.59	1	5.56	1.2	0	0	0	1	1	1	1
GO:0004090	5	5	100	1.13	0	0	0	0	0	0	0	0	0	1	1	1	1
GO:0004091	7	4	57.14	0.65	0	0	0	1	14.29	3.08	2	28.57	8.06	1	1	1	1
GO:0004092	2	2	100	1.13	0	0	0	0	0	0	0	0	0	1	1	1	1
GO:0004093	1	1	100	1.13	0	0	0	0	0	0	0	0	0	1	1	1	1
GO:0004094	1	1	100	1.13	0	0	0	0	0	0	0	0	0	1	1	1	1
GO:0004095	6	6	100	1.13	0	0	0	0	0	0	0	0	0	1	1	1	1
GO:0004096	6	6	100	1.13	0	0	0	0	0	0	0	0	0	1	1	1	1
GO:0004098	1	1	100	1.13	0	0	0	0	0	0	0	0	0	1	1	1	1
GO:0004103	4	4	100	1.13	0	0	0	0	0	0	0	0	0	1	1	1	1
GO:0004105	3	2	66.67	0.75	1	33.33	9.54	0	0	0	0	0	0	1	1	1	1
GO:0004108	9	9	100	1.13	0	0	0	0	0	0	0	0	0	1	1	1	1
GO:0004109	2	2	100	1.13	0	0	0	0	0	0	0	0	0	1	1	1	1
GO:0004111	4	4	100	1.13	0	0	0	0	0	0	0	0	0	1	1	1	1
GO:0004113	4	4	100	1.13	0	0	0	0	0	0	0	0	0	1	1	1	1
GO:0004114	8	7	87.5	0.99	0	0	0	0	0	0	1	12.5	3.53	1	1	1	1
GO:0004115	3	3	100	1.13	0	0	0	0	0	0	0	0	0	1	1	1	1
GO:0004116	1	1	100	1.13	0	0	0	0	0	0	0	0	0	1	1	1	1
GO:0004119	1	1	100	1.13	0	0	0	0	0	0	0	0	0	1	1	1	1
GO:0004122	1	1	100	1.13	0	0	0	0	0	0	0	0	0	1	1	1	1
GO:0004127	2	2	100	1.13	0	0	0	0	0	0	0	0	0	1	1	1	1
GO:0004128	3	3	100	1.13	0	0	0	0	0	0	0	0	0	1	1	1	1
GO:0004129	34	31	91.18	1.03	0	0	0	3	8.82	1.9	0	0	0	1	1	1	1
GO:0004132	1	1	100	1.13	0	0	0	0	0	0	0	0	0	1	1	1	1
GO:0004137	2	2	100	1.13	0	0	0	0	0	0	0	0	0	1	1	1	1
GO:0004138	2	2	100	1.13	0	0	0	0	0	0	0	0	0	1	1	1	1
GO:0004139	2	2	100	1.13	0	0	0	0	0	0	0	0	0	1	1	1	1
GO:0004140	4	4	100	1.13	0	0	0	0	0	0	0	0	0	1	1	1	1
GO:0004143	7	6	85.71	0.97	1	14.29	4.09	0	0	0	0	0	0	1	1	1	1
GO:0004144	1	1	100	1.13	0	0	0	0	0	0	0	0	0	1	1	1	1
GO:0004145	3	3	100	1.13	0	0	0	0	0	0	0	0	0	1	1	1	1
GO:0004146	1	1	100	1.13	0	0	0	0	0	0	0	0	0	1	1	1	1
GO:0004148	4	4	100	1.13	0	0	0	0	0	0	0	0	0	1	1	1	1
GO:0004149	1	1	100	1.13	0	0	0	0	0	0	0	0	0	1	1	1	1
GO:0004152	5	5	100	1.13	0	0	0	0	0	0	0	0	0	1	1	1	1
GO:0004155	2	2	100	1.13	0	0	0	0	0	0	0	0	0	1	1	1	1
GO:0004156	1	1	100	1.13	0	0	0	0	0	0	0	0	0	1	1	1	1
GO:0004157	3	3	100	1.13	0	0	0	0	0	0	0	0	0	1	1	1	1

GO:0004158	1	1	100	1.13	0	0	0	0	0	0	0	0	0	1	1	1	1
GO:0004161	1	1	100	1.13	0	0	0	0	0	0	0	0	0	1	1	1	1
GO:0004164	2	1	50	0.57	1	50	14.32	0	0	0	0	0	0	1	1	1	1
GO:0004165	3	3	100	1.13	0	0	0	0	0	0	0	0	0	1	1	1	1
GO:0004167	1	0	0	0	1	100	28.63	0	0	0	0	0	0	1	1	1	1
GO:0004169	1	1	100	1.13	0	0	0	0	0	0	0	0	0	1	1	1	1
GO:0004170	3	3	100	1.13	0	0	0	0	0	0	0	0	0	1	1	1	1
GO:0004171	2	0	0	0	0	0	0	1	50	10.76	1	50	14.11	1	1	1	1
GO:0004174	3	3	100	1.13	0	0	0	0	0	0	0	0	0	1	1	1	1
GO:0004175	43	40	93.02	1.05	0	0	0	3	6.98	1.5	0	0	0	1	1	1	1
GO:0004176	5	4	80	0.91	0	0	0	1	20	4.31	0	0	0	1	1	1	1
GO:0004177	47	47	100	1.13	0	0	0	0	0	0	0	0	0	1	1	1	1
GO:0004178	5	5	100	1.13	0	0	0	0	0	0	0	0	0	1	1	1	1
GO:0004179	11	11	100	1.13	0	0	0	0	0	0	0	0	0	1	1	1	1
GO:0004180	39	30	76.92	0.87	0	0	0	7	17.95	3.86	2	5.13	1.45	1	1	1	1
GO:0004182	24	20	83.33	0.94	0	0	0	3	12.5	2.69	1	4.17	1.18	1	1	1	1
GO:0004183	1	1	100	1.13	0	0	0	0	0	0	0	0	0	1	1	1	1
GO:0004184	1	0	0	0	0	0	0	1	100	21.53	0	0	0	1	1	1	1
GO:0004185	6	3	50	0.57	0	0	0	3	50	10.76	0	0	0	1	1	1	1
GO:0004186	5	2	40	0.45	0	0	0	3	60	12.92	0	0	0	1	1	1	1
GO:0004188	2	2	100	1.13	0	0	0	0	0	0	0	0	0	1	1	1	1
GO:0004190	39	31	79.49	0.9	2	5.13	1.47	4	10.26	2.21	2	5.13	1.45	1	1	1	1
GO:0004192	15	14	93.33	1.06	0	0	0	1	6.67	1.44	0	0	0	1	1	1	1
GO:0004193	1	1	100	1.13	0	0	0	0	0	0	0	0	0	1	1	1	1
GO:0004194	25	24	96	1.09	0	0	0	1	4	0.86	0	0	0	1	1	1	1
GO:0004195	1	1	100	1.13	0	0	0	0	0	0	0	0	0	1	1	1	1
GO:0004197	172	164	95.35	1.08	1	0.58	0.17	3	1.74	0.38	4	2.33	0.66	1	1	1	1
GO:0004198	13	13	100	1.13	0	0	0	0	0	0	0	0	0	1	1	1	1
GO:0004199	6	5	83.33	0.94	0	0	0	0	0	0	1	16.67	4.7	1	1	1	1
GO:0004200	3	3	100	1.13	0	0	0	0	0	0	0	0	0	1	1	1	1
GO:0004202	3	3	100	1.13	0	0	0	0	0	0	0	0	0	1	1	1	1
GO:0004203	3	3	100	1.13	0	0	0	0	0	0	0	0	0	1	1	1	1
GO:0004204	3	3	100	1.13	0	0	0	0	0	0	0	0	0	1	1	1	1
GO:0004205	3	3	100	1.13	0	0	0	0	0	0	0	0	0	1	1	1	1
GO:0004206	3	3	100	1.13	0	0	0	0	0	0	0	0	0	1	1	1	1
GO:0004207	3	3	100	1.13	0	0	0	0	0	0	0	0	0	1	1	1	1
GO:0004208	3	3	100	1.13	0	0	0	0	0	0	0	0	0	1	1	1	1
GO:0004209	3	3	100	1.13	0	0	0	0	0	0	0	0	0	1	1	1	1
GO:0004210	3	3	100	1.13	0	0	0	0	0	0	0	0	0	1	1	1	1
GO:0004211	3	3	100	1.13	0	0	0	0	0	0	0	0	0	1	1	1	1
GO:0004212	3	3	100	1.13	0	0	0	0	0	0	0	0	0	1	1	1	1

GO:0004213	6	6	100	1.13	0	0	0	0	0	0	0	0	0	1	1	1	1
GO:0004214	4	4	100	1.13	0	0	0	0	0	0	0	0	0	1	1	1	1
GO:0004215	4	4	100	1.13	0	0	0	0	0	0	0	0	0	1	1	1	1
GO:0004216	2	0	0	0	0	0	0	0	0	0	2	100	28.22	1	1	1	1
GO:0004217	3	3	100	1.13	0	0	0	0	0	0	0	0	0	1	1	1	1
GO:0004218	3	0	0	0	0	0	0	2	66.67	14.35	1	33.33	9.41	1	1	1	1
GO:0004219	1	1	100	1.13	0	0	0	0	0	0	0	0	0	1	1	1	1
GO:0004221	79	78	98.73	1.12	1	1.27	0.36	0	0	0	0	0	0	1	1	1	1
GO:0004222	115	82	71.3	0.81	15	13.04	3.73	15	13.04	2.81	3	2.61	0.74	1	0.07648839	1	1
GO:0004228	1	1	100	1.13	0	0	0	0	0	0	0	0	0	1	1	1	1
GO:0004229	1	1	100	1.13	0	0	0	0	0	0	0	0	0	1	1	1	1
GO:0004232	3	0	0	0	0	0	0	3	100	21.53	0	0	0	1	1	0.43202791	1
GO:0004235	1	0	0	0	0	0	0	1	100	21.53	0	0	0	1	1	1	1
GO:0004237	2	2	100	1.13	0	0	0	0	0	0	0	0	0	1	1	1	1
GO:0004239	12	12	100	1.13	0	0	0	0	0	0	0	0	0	1	1	1	1
GO:0004240	3	3	100	1.13	0	0	0	0	0	0	0	0	0	1	1	1	1
GO:0004243	3	3	100	1.13	0	0	0	0	0	0	0	0	0	1	1	1	1
GO:0004245	6	5	83.33	0.94	1	16.67	4.77	0	0	0	0	0	0	1	1	1	1
GO:0004246	2	2	100	1.13	0	0	0	0	0	0	0	0	0	1	1	1	1
GO:0004249	8	5	62.5	0.71	3	37.5	10.74	0	0	0	0	0	0	1	1	1	1
GO:0004250	4	4	100	1.13	0	0	0	0	0	0	0	0	0	1	1	1	1
GO:0004251	1	1	100	1.13	0	0	0	0	0	0	0	0	0	1	1	1	1
GO:0004252	119	94	78.99	0.89	10	8.4	2.41	7	5.88	1.27	8	6.72	1.9	1	1	1	1
GO:0004254	1	1	100	1.13	0	0	0	0	0	0	0	0	0	1	1	1	1
GO:0004258	1	0	0	0	0	0	0	1	100	21.53	0	0	0	1	1	1	1
GO:0004263	50	40	80	0.91	2	4	1.15	2	4	0.86	6	12	3.39	1	1	1	1
GO:0004274	6	6	100	1.13	0	0	0	0	0	0	0	0	0	1	1	1	1
GO:0004277	2	0	0	0	0	0	0	2	100	21.53	0	0	0	1	1	1	1
GO:0004278	1	0	0	0	0	0	0	1	100	21.53	0	0	0	1	1	1	1
GO:0004283	1	0	0	0	0	0	0	0	0	0	1	100	28.22	1	1	1	1
GO:0004286	2	1	50	0.57	1	50	14.32	0	0	0	0	0	0	1	1	1	1
GO:0004287	6	6	100	1.13	0	0	0	0	0	0	0	0	0	1	1	1	1
GO:0004289	21	19	90.48	1.02	2	9.52	2.73	0	0	0	0	0	0	1	1	1	1
GO:0004293	1	1	100	1.13	0	0	0	0	0	0	0	0	0	1	1	1	1
GO:0004294	4	4	100	1.13	0	0	0	0	0	0	0	0	0	1	1	1	1
GO:0004295	61	46	75.41	0.85	2	3.28	0.94	5	8.2	1.76	8	13.11	3.7	1	1	1	1
GO:0004296	2	2	100	1.13	0	0	0	0	0	0	0	0	0	1	1	1	1
GO:0004299	27	25	92.59	1.05	0	0	0	2	7.41	1.59	0	0	0	1	1	1	1
GO:0004300	8	8	100	1.13	0	0	0	0	0	0	0	0	0	1	1	1	1
GO:0004301	5	5	100	1.13	0	0	0	0	0	0	0	0	0	1	1	1	1
GO:0004303	11	8	72.73	0.82	0	0	0	1	9.09	1.96	2	18.18	5.13	1	1	1	1

GO:0004305	2	2	100	1.13	0	0	0	0	0	0	0	0	0	1	1	1	1
GO:0004308	2	2	100	1.13	0	0	0	0	0	0	0	0	0	1	1	1	1
GO:0004310	5	5	100	1.13	0	0	0	0	0	0	0	0	0	1	1	1	1
GO:0004311	4	4	100	1.13	0	0	0	0	0	0	0	0	0	1	1	1	1
GO:0004312	3	3	100	1.13	0	0	0	0	0	0	0	0	0	1	1	1	1
GO:0004316	2	2	100	1.13	0	0	0	0	0	0	0	0	0	1	1	1	1
GO:0004321	1	1	100	1.13	0	0	0	0	0	0	0	0	0	1	1	1	1
GO:0004324	1	1	100	1.13	0	0	0	0	0	0	0	0	0	1	1	1	1
GO:0004325	4	4	100	1.13	0	0	0	0	0	0	0	0	0	1	1	1	1
GO:0004327	2	2	100	1.13	0	0	0	0	0	0	0	0	0	1	1	1	1
GO:0004329	9	9	100	1.13	0	0	0	0	0	0	0	0	0	1	1	1	1
GO:0004331	4	4	100	1.13	0	0	0	0	0	0	0	0	0	1	1	1	1
GO:0004332	4	2	50	0.57	0	0	0	0	0	0	2	50	14.11	1	1	1	1
GO:0004333	2	2	100	1.13	0	0	0	0	0	0	0	0	0	1	1	1	1
GO:0004334	2	2	100	1.13	0	0	0	0	0	0	0	0	0	1	1	1	1
GO:0004335	6	6	100	1.13	0	0	0	0	0	0	0	0	0	1	1	1	1
GO:0004337	2	2	100	1.13	0	0	0	0	0	0	0	0	0	1	1	1	1
GO:0004342	4	4	100	1.13	0	0	0	0	0	0	0	0	0	1	1	1	1
GO:0004343	6	6	100	1.13	0	0	0	0	0	0	0	0	0	1	1	1	1
GO:0004345	4	2	50	0.57	0	0	0	2	50	10.76	0	0	0	1	1	1	1
GO:0004346	1	0	0	0	0	0	0	1	100	21.53	0	0	0	1	1	1	1
GO:0004347	4	4	100	1.13	0	0	0	0	0	0	0	0	0	1	1	1	1
GO:0004348	3	0	0	0	1	33.33	9.54	0	0	0	2	66.67	18.82	1	1	1	1
GO:0004349	3	3	100	1.13	0	0	0	0	0	0	0	0	0	1	1	1	1
GO:0004350	3	3	100	1.13	0	0	0	0	0	0	0	0	0	1	1	1	1
GO:0004351	4	4	100	1.13	0	0	0	0	0	0	0	0	0	1	1	1	1
GO:0004353	2	2	100	1.13	0	0	0	0	0	0	0	0	0	1	1	1	1
GO:0004356	5	5	100	1.13	0	0	0	0	0	0	0	0	0	1	1	1	1
GO:0004357	2	2	100	1.13	0	0	0	0	0	0	0	0	0	1	1	1	1
GO:0004359	1	1	100	1.13	0	0	0	0	0	0	0	0	0	1	1	1	1
GO:0004360	1	1	100	1.13	0	0	0	0	0	0	0	0	0	1	1	1	1
GO:0004361	1	1	100	1.13	0	0	0	0	0	0	0	0	0	1	1	1	1
GO:0004362	5	5	100	1.13	0	0	0	0	0	0	0	0	0	1	1	1	1
GO:0004363	3	3	100	1.13	0	0	0	0	0	0	0	0	0	1	1	1	1
GO:0004364	22	20	90.91	1.03	0	0	0	1	4.55	0.98	1	4.55	1.28	1	1	1	1
GO:0004366	1	0	0	0	0	0	0	1	100	21.53	0	0	0	1	1	1	1
GO:0004367	4	4	100	1.13	0	0	0	0	0	0	0	0	0	1	1	1	1
GO:0004368	1	1	100	1.13	0	0	0	0	0	0	0	0	0	1	1	1	1
GO:0004370	3	3	100	1.13	0	0	0	0	0	0	0	0	0	1	1	1	1
GO:0004371	1	1	100	1.13	0	0	0	0	0	0	0	0	0	1	1	1	1
GO:0004372	5	2	40	0.45	0	0	0	3	60	12.92	0	0	0	1	1	1	1

GO:0004373	3	3	100	1.13	0	0	0	0	0	0	0	0	0	1	1	1	1
GO:0004374	1	1	100	1.13	0	0	0	0	0	0	0	0	0	1	1	1	1
GO:0004375	1	1	100	1.13	0	0	0	0	0	0	0	0	0	1	1	1	1
GO:0004376	4	4	100	1.13	0	0	0	0	0	0	0	0	0	1	1	1	1
GO:0004379	4	4	100	1.13	0	0	0	0	0	0	0	0	0	1	1	1	1
GO:0004382	1	1	100	1.13	0	0	0	0	0	0	0	0	0	1	1	1	1
GO:0004383	5	5	100	1.13	0	0	0	0	0	0	0	0	0	1	1	1	1
GO:0004384	1	1	100	1.13	0	0	0	0	0	0	0	0	0	1	1	1	1
GO:0004385	7	6	85.71	0.97	0	0	0	1	14.29	3.08	0	0	0	1	1	1	1
GO:0004386	158	146	92.41	1.05	5	3.16	0.91	2	1.27	0.27	5	3.16	0.89	1	1	1	1
GO:0004392	7	7	100	1.13	0	0	0	0	0	0	0	0	0	1	1	1	1
GO:0004393	4	4	100	1.13	0	0	0	0	0	0	0	0	0	1	1	1	1
GO:0004394	3	3	100	1.13	0	0	0	0	0	0	0	0	0	1	1	1	1
GO:0004396	12	7	58.33	0.66	2	16.67	4.77	1	8.33	1.79	2	16.67	4.7	1	1	1	1
GO:0004397	2	1	50	0.57	0	0	0	1	50	10.76	0	0	0	1	1	1	1
GO:0004402	8	7	87.5	0.99	0	0	0	1	12.5	2.69	0	0	0	1	1	1	1
GO:0004407	14	14	100	1.13	0	0	0	0	0	0	0	0	0	1	1	1	1
GO:0004408	3	3	100	1.13	0	0	0	0	0	0	0	0	0	1	1	1	1
GO:0004415	4	4	100	1.13	0	0	0	0	0	0	0	0	0	1	1	1	1
GO:0004416	7	7	100	1.13	0	0	0	0	0	0	0	0	0	1	1	1	1
GO:0004418	5	4	80	0.91	1	20	5.73	0	0	0	0	0	0	1	1	1	1
GO:0004419	3	3	100	1.13	0	0	0	0	0	0	0	0	0	1	1	1	1
GO:0004420	3	3	100	1.13	0	0	0	0	0	0	0	0	0	1	1	1	1
GO:0004422	2	2	100	1.13	0	0	0	0	0	0	0	0	0	1	1	1	1
GO:0004423	1	1	100	1.13	0	0	0	0	0	0	0	0	0	1	1	1	1
GO:0004427	6	6	100	1.13	0	0	0	0	0	0	0	0	0	1	1	1	1
GO:0004428	12	11	91.67	1.04	1	8.33	2.39	0	0	0	0	0	0	1	1	1	1
GO:0004429	3	2	66.67	0.75	1	33.33	9.54	0	0	0	0	0	0	1	1	1	1
GO:0004430	3	3	100	1.13	0	0	0	0	0	0	0	0	0	1	1	1	1
GO:0004431	1	1	100	1.13	0	0	0	0	0	0	0	0	0	1	1	1	1
GO:0004435	11	11	100	1.13	0	0	0	0	0	0	0	0	0	1	1	1	1
GO:0004437	33	33	100	1.13	0	0	0	0	0	0	0	0	0	1	1	1	1
GO:0004439	8	8	100	1.13	0	0	0	0	0	0	0	0	0	1	1	1	1
GO:0004441	1	1	100	1.13	0	0	0	0	0	0	0	0	0	1	1	1	1
GO:0004445	8	7	87.5	0.99	0	0	0	1	12.5	2.69	0	0	0	1	1	1	1
GO:0004448	5	1	20	0.23	1	20	5.73	2	40	8.61	1	20	5.64	1	1	1	1
GO:0004449	3	3	100	1.13	0	0	0	0	0	0	0	0	0	1	1	1	1
GO:0004450	16	16	100	1.13	0	0	0	0	0	0	0	0	0	1	1	1	1
GO:0004452	2	1	50	0.57	0	0	0	0	0	0	1	50	14.11	1	1	1	1
GO:0004459	9	9	100	1.13	0	0	0	0	0	0	0	0	0	1	1	1	1
GO:0004462	8	7	87.5	0.99	1	12.5	3.58	0	0	0	0	0	0	1	1	1	1

GO:0004463	3	3	100	1.13	0	0	0	0	0	0	0	0	0	1	1	1	1
GO:0004465	13	10	76.92	0.87	0	0	0	2	15.38	3.31	1	7.69	2.17	1	1	1	1
GO:0004466	2	2	100	1.13	0	0	0	0	0	0	0	0	0	1	1	1	1
GO:0004467	4	3	75	0.85	1	25	7.16	0	0	0	0	0	0	1	1	1	1
GO:0004469	2	2	100	1.13	0	0	0	0	0	0	0	0	0	1	1	1	1
GO:0004470	13	13	100	1.13	0	0	0	0	0	0	0	0	0	1	1	1	1
GO:0004471	4	4	100	1.13	0	0	0	0	0	0	0	0	0	1	1	1	1
GO:0004473	3	3	100	1.13	0	0	0	0	0	0	0	0	0	1	1	1	1
GO:0004476	2	2	100	1.13	0	0	0	0	0	0	0	0	0	1	1	1	1
GO:0004477	11	11	100	1.13	0	0	0	0	0	0	0	0	0	1	1	1	1
GO:0004478	4	2	50	0.57	0	0	0	2	50	10.76	0	0	0	1	1	1	1
GO:0004482	3	0	0	0	3	100	28.63	0	0	0	0	0	0	1	0.175770672	1	1
GO:0004484	7	7	100	1.13	0	0	0	0	0	0	0	0	0	1	1	1	1
GO:0004485	1	1	100	1.13	0	0	0	0	0	0	0	0	0	1	1	1	1
GO:0004488	11	11	100	1.13	0	0	0	0	0	0	0	0	0	1	1	1	1
GO:0004489	1	1	100	1.13	0	0	0	0	0	0	0	0	0	1	1	1	1
GO:0004491	1	1	100	1.13	0	0	0	0	0	0	0	0	0	1	1	1	1
GO:0004494	1	1	100	1.13	0	0	0	0	0	0	0	0	0	1	1	1	1
GO:0004495	5	1	20	0.23	1	20	5.73	2	40	8.61	1	20	5.64	1	1	1	1
GO:0004496	1	1	100	1.13	0	0	0	0	0	0	0	0	0	1	1	1	1
GO:0004497	81	59	72.84	0.82	2	2.47	0.71	6	7.41	1.59	14	17.28	4.88	1	1	1	0.009987669
GO:0004499	5	3	60	0.68	0	0	0	0	0	0	2	40	11.29	1	1	1	1
GO:0004500	1	1	100	1.13	0	0	0	0	0	0	0	0	0	1	1	1	1
GO:0004503	1	0	0	0	0	0	0	0	0	0	1	100	28.22	1	1	1	1
GO:0004504	1	1	100	1.13	0	0	0	0	0	0	0	0	0	1	1	1	1
GO:0004505	2	2	100	1.13	0	0	0	0	0	0	0	0	0	1	1	1	1
GO:0004506	2	2	100	1.13	0	0	0	0	0	0	0	0	0	1	1	1	1
GO:0004508	1	0	0	0	0	0	0	1	100	21.53	0	0	0	1	1	1	1
GO:0004510	1	1	100	1.13	0	0	0	0	0	0	0	0	0	1	1	1	1
GO:0004512	2	2	100	1.13	0	0	0	0	0	0	0	0	0	1	1	1	1
GO:0004513	1	1	100	1.13	0	0	0	0	0	0	0	0	0	1	1	1	1
GO:0004514	1	1	100	1.13	0	0	0	0	0	0	0	0	0	1	1	1	1
GO:0004516	2	2	100	1.13	0	0	0	0	0	0	0	0	0	1	1	1	1
GO:0004517	2	2	100	1.13	0	0	0	0	0	0	0	0	0	1	1	1	1
GO:0004518	71	59	83.1	0.94	4	5.63	1.61	7	9.86	2.12	1	1.41	0.4	1	1	1	1
GO:0004519	66	54	81.82	0.93	5	7.58	2.17	4	6.06	1.3	3	4.55	1.28	1	1	1	1
GO:0004520	1	1	100	1.13	0	0	0	0	0	0	0	0	0	1	1	1	1
GO:0004521	1	1	100	1.13	0	0	0	0	0	0	0	0	0	1	1	1	1
GO:0004522	6	2	33.33	0.38	2	33.33	9.54	1	16.67	3.59	1	16.67	4.7	1	1	1	1
GO:0004523	2	2	100	1.13	0	0	0	0	0	0	0	0	0	1	1	1	1
GO:0004524	1	1	100	1.13	0	0	0	0	0	0	0	0	0	1	1	1	1

GO:0004525	5	5	100	1.13	0	0	0	0	0	0	0	0	0	1	1	1	1
GO:0004526	10	9	90	1.02	1	10	2.86	0	0	0	0	0	0	1	1	1	1
GO:0004527	49	46	93.88	1.06	1	2.04	0.58	2	4.08	0.88	0	0	0	1	1	1	1
GO:0004528	2	2	100	1.13	0	0	0	0	0	0	0	0	0	1	1	1	1
GO:0004531	3	2	66.67	0.75	1	33.33	9.54	0	0	0	0	0	0	1	1	1	1
GO:0004536	2	1	50	0.57	0	0	0	1	50	10.76	0	0	0	1	1	1	1
GO:0004540	6	6	100	1.13	0	0	0	0	0	0	0	0	0	1	1	1	1
GO:0004550	16	14	87.5	0.99	1	6.25	1.79	1	6.25	1.35	0	0	0	1	1	1	1
GO:0004551	2	2	100	1.13	0	0	0	0	0	0	0	0	0	1	1	1	1
GO:0004553	39	34	87.18	0.99	0	0	0	1	2.56	0.55	4	10.26	2.89	1	1	1	1
GO:0004555	1	1	100	1.13	0	0	0	0	0	0	0	0	0	1	1	1	1
GO:0004556	9	8	88.89	1.01	0	0	0	0	0	0	1	11.11	3.14	1	1	1	1
GO:0004557	5	0	0	0	0	0	0	1	20	4.31	4	80	22.58	1	1	1	0.039629347
GO:0004558	7	7	100	1.13	0	0	0	0	0	0	0	0	0	1	1	1	1
GO:0004559	15	14	93.33	1.06	1	6.67	1.91	0	0	0	0	0	0	1	1	1	1
GO:0004560	12	12	100	1.13	0	0	0	0	0	0	0	0	0	1	1	1	1
GO:0004563	15	15	100	1.13	0	0	0	0	0	0	0	0	0	1	1	1	1
GO:0004565	8	8	100	1.13	0	0	0	0	0	0	0	0	0	1	1	1	1
GO:0004566	7	5	71.43	0.81	0	0	0	2	28.57	6.15	0	0	0	1	1	1	1
GO:0004567	3	3	100	1.13	0	0	0	0	0	0	0	0	0	1	1	1	1
GO:0004568	1	0	0	0	0	0	0	0	0	0	1	100	28.22	1	1	1	1
GO:0004571	12	12	100	1.13	0	0	0	0	0	0	0	0	0	1	1	1	1
GO:0004572	1	1	100	1.13	0	0	0	0	0	0	0	0	0	1	1	1	1
GO:0004573	2	2	100	1.13	0	0	0	0	0	0	0	0	0	1	1	1	1
GO:0004576	10	10	100	1.13	0	0	0	0	0	0	0	0	0	1	1	1	1
GO:0004579	10	10	100	1.13	0	0	0	0	0	0	0	0	0	1	1	1	1
GO:0004580	4	4	100	1.13	0	0	0	0	0	0	0	0	0	1	1	1	1
GO:0004582	2	2	100	1.13	0	0	0	0	0	0	0	0	0	1	1	1	1
GO:0004585	1	0	0	0	0	0	0	1	100	21.53	0	0	0	1	1	1	1
GO:0004586	2	2	100	1.13	0	0	0	0	0	0	0	0	0	1	1	1	1
GO:0004587	2	2	100	1.13	0	0	0	0	0	0	0	0	0	1	1	1	1
GO:0004588	1	1	100	1.13	0	0	0	0	0	0	0	0	0	1	1	1	1
GO:0004590	1	1	100	1.13	0	0	0	0	0	0	0	0	0	1	1	1	1
GO:0004591	4	4	100	1.13	0	0	0	0	0	0	0	0	0	1	1	1	1
GO:0004594	2	2	100	1.13	0	0	0	0	0	0	0	0	0	1	1	1	1
GO:0004595	4	4	100	1.13	0	0	0	0	0	0	0	0	0	1	1	1	1
GO:0004597	8	5	62.5	0.71	3	37.5	10.74	0	0	0	0	0	0	1	1	1	1
GO:0004600	16	15	93.75	1.06	0	0	0	0	0	0	1	6.25	1.76	1	1	1	1
GO:0004601	57	53	92.98	1.05	1	1.75	0.5	3	5.26	1.13	0	0	0	1	1	1	1
GO:0004602	6	4	66.67	0.75	1	16.67	4.77	1	16.67	3.59	0	0	0	1	1	1	1
GO:0004603	1	1	100	1.13	0	0	0	0	0	0	0	0	0	1	1	1	1

GO:0004605	3	3	100	1.13	0	0	0	0	0	0	0	0	0	1	1	1	1
GO:0004607	3	3	100	1.13	0	0	0	0	0	0	0	0	0	1	1	1	1
GO:0004609	4	4	100	1.13	0	0	0	0	0	0	0	0	0	1	1	1	1
GO:0004610	4	4	100	1.13	0	0	0	0	0	0	0	0	0	1	1	1	1
GO:0004613	4	4	100	1.13	0	0	0	0	0	0	0	0	0	1	1	1	1
GO:0004614	6	6	100	1.13	0	0	0	0	0	0	0	0	0	1	1	1	1
GO:0004615	8	8	100	1.13	0	0	0	0	0	0	0	0	0	1	1	1	1
GO:0004616	3	3	100	1.13	0	0	0	0	0	0	0	0	0	1	1	1	1
GO:0004618	3	3	100	1.13	0	0	0	0	0	0	0	0	0	1	1	1	1
GO:0004619	5	3	60	0.68	0	0	0	2	40	8.61	0	0	0	1	1	1	1
GO:0004620	3	2	66.67	0.75	1	33.33	9.54	0	0	0	0	0	0	1	1	1	1
GO:0004621	1	1	100	1.13	0	0	0	0	0	0	0	0	0	1	1	1	1
GO:0004622	5	4	80	0.91	1	20	5.73	0	0	0	0	0	0	1	1	1	1
GO:0004623	20	15	75	0.85	3	15	4.29	1	5	1.08	1	5	1.41	1	1	1	1
GO:0004624	2	2	100	1.13	0	0	0	0	0	0	0	0	0	1	1	1	1
GO:0004625	2	2	100	1.13	0	0	0	0	0	0	0	0	0	1	1	1	1
GO:0004626	2	2	100	1.13	0	0	0	0	0	0	0	0	0	1	1	1	1
GO:0004627	2	2	100	1.13	0	0	0	0	0	0	0	0	0	1	1	1	1
GO:0004628	2	2	100	1.13	0	0	0	0	0	0	0	0	0	1	1	1	1
GO:0004629	18	17	94.44	1.07	0	0	0	1	5.56	1.2	0	0	0	1	1	1	1
GO:0004630	2	2	100	1.13	0	0	0	0	0	0	0	0	0	1	1	1	1
GO:0004631	2	2	100	1.13	0	0	0	0	0	0	0	0	0	1	1	1	1
GO:0004634	6	6	100	1.13	0	0	0	0	0	0	0	0	0	1	1	1	1
GO:0004637	9	9	100	1.13	0	0	0	0	0	0	0	0	0	1	1	1	1
GO:0004638	7	7	100	1.13	0	0	0	0	0	0	0	0	0	1	1	1	1
GO:0004639	7	7	100	1.13	0	0	0	0	0	0	0	0	0	1	1	1	1
GO:0004641	6	6	100	1.13	0	0	0	0	0	0	0	0	0	1	1	1	1
GO:0004643	4	4	100	1.13	0	0	0	0	0	0	0	0	0	1	1	1	1
GO:0004644	6	6	100	1.13	0	0	0	0	0	0	0	0	0	1	1	1	1
GO:0004645	13	13	100	1.13	0	0	0	0	0	0	0	0	0	1	1	1	1
GO:0004646	2	2	100	1.13	0	0	0	0	0	0	0	0	0	1	1	1	1
GO:0004647	1	1	100	1.13	0	0	0	0	0	0	0	0	0	1	1	1	1
GO:0004648	2	2	100	1.13	0	0	0	0	0	0	0	0	0	1	1	1	1
GO:0004649	2	2	100	1.13	0	0	0	0	0	0	0	0	0	1	1	1	1
GO:0004652	1	1	100	1.13	0	0	0	0	0	0	0	0	0	1	1	1	1
GO:0004653	12	12	100	1.13	0	0	0	0	0	0	0	0	0	1	1	1	1
GO:0004654	1	1	100	1.13	0	0	0	0	0	0	0	0	0	1	1	1	1
GO:0004655	5	5	100	1.13	0	0	0	0	0	0	0	0	0	1	1	1	1
GO:0004656	13	13	100	1.13	0	0	0	0	0	0	0	0	0	1	1	1	1
GO:0004657	3	3	100	1.13	0	0	0	0	0	0	0	0	0	1	1	1	1
GO:0004658	1	1	100	1.13	0	0	0	0	0	0	0	0	0	1	1	1	1

GO:0004659	2	2	100	1.13	0	0	0	0	0	0	0	0	0	1	1	1	1
GO:0004660	1	1	100	1.13	0	0	0	0	0	0	0	0	0	1	1	1	1
GO:0004661	1	1	100	1.13	0	0	0	0	0	0	0	0	0	1	1	1	1
GO:0004662	1	1	100	1.13	0	0	0	0	0	0	0	0	0	1	1	1	1
GO:0004663	1	1	100	1.13	0	0	0	0	0	0	0	0	0	1	1	1	1
GO:0004664	1	1	100	1.13	0	0	0	0	0	0	0	0	0	1	1	1	1
GO:0004666	7	7	100	1.13	0	0	0	0	0	0	0	0	0	1	1	1	1
GO:0004667	4	3	75	0.85	1	25	7.16	0	0	0	0	0	0	1	1	1	1
GO:0004668	4	4	100	1.13	0	0	0	0	0	0	0	0	0	1	1	1	1
GO:0004671	1	1	100	1.13	0	0	0	0	0	0	0	0	0	1	1	1	1
GO:0004672	474	437	92.19	1.04	7	1.48	0.42	23	4.85	1.04	7	1.48	0.42	1	1	1	1
GO:0004673	8	8	100	1.13	0	0	0	0	0	0	0	0	0	1	1	1	1
GO:0004674	541	503	92.98	1.05	9	1.66	0.48	20	3.7	0.8	9	1.66	0.47	0.069349507	1	1	1
GO:0004675	29	29	100	1.13	0	0	0	0	0	0	0	0	0	1	1	1	1
GO:0004676	21	21	100	1.13	0	0	0	0	0	0	0	0	0	1	1	1	1
GO:0004677	17	17	100	1.13	0	0	0	0	0	0	0	0	0	1	1	1	1
GO:0004678	17	17	100	1.13	0	0	0	0	0	0	0	0	0	1	1	1	1
GO:0004679	21	21	100	1.13	0	0	0	0	0	0	0	0	0	1	1	1	1
GO:0004680	17	17	100	1.13	0	0	0	0	0	0	0	0	0	1	1	1	1
GO:0004681	17	17	100	1.13	0	0	0	0	0	0	0	0	0	1	1	1	1
GO:0004682	19	19	100	1.13	0	0	0	0	0	0	0	0	0	1	1	1	1
GO:0004683	19	19	100	1.13	0	0	0	0	0	0	0	0	0	1	1	1	1
GO:0004684	4	4	100	1.13	0	0	0	0	0	0	0	0	0	1	1	1	1
GO:0004685	7	7	100	1.13	0	0	0	0	0	0	0	0	0	1	1	1	1
GO:0004686	17	17	100	1.13	0	0	0	0	0	0	0	0	0	1	1	1	1
GO:0004688	17	17	100	1.13	0	0	0	0	0	0	0	0	0	1	1	1	1
GO:0004689	6	6	100	1.13	0	0	0	0	0	0	0	0	0	1	1	1	1
GO:0004690	17	17	100	1.13	0	0	0	0	0	0	0	0	0	1	1	1	1
GO:0004691	17	17	100	1.13	0	0	0	0	0	0	0	0	0	1	1	1	1
GO:0004692	18	18	100	1.13	0	0	0	0	0	0	0	0	0	1	1	1	1
GO:0004693	41	41	100	1.13	0	0	0	0	0	0	0	0	0	1	1	1	1
GO:0004694	17	17	100	1.13	0	0	0	0	0	0	0	0	0	1	1	1	1
GO:0004695	17	17	100	1.13	0	0	0	0	0	0	0	0	0	1	1	1	1
GO:0004696	19	19	100	1.13	0	0	0	0	0	0	0	0	0	1	1	1	1
GO:0004697	18	18	100	1.13	0	0	0	0	0	0	0	0	0	1	1	1	1
GO:0004698	18	18	100	1.13	0	0	0	0	0	0	0	0	0	1	1	1	1
GO:0004700	17	17	100	1.13	0	0	0	0	0	0	0	0	0	1	1	1	1
GO:0004701	17	17	100	1.13	0	0	0	0	0	0	0	0	0	1	1	1	1
GO:0004702	31	31	100	1.13	0	0	0	0	0	0	0	0	0	1	1	1	1
GO:0004703	19	19	100	1.13	0	0	0	0	0	0	0	0	0	1	1	1	1
GO:0004704	18	18	100	1.13	0	0	0	0	0	0	0	0	0	1	1	1	1

GO:0004705	19	19	100	1.13	0	0	0	0	0	0	0	0	0	1	1	1	1
GO:0004706	17	17	100	1.13	0	0	0	0	0	0	0	0	0	1	1	1	1
GO:0004707	33	33	100	1.13	0	0	0	0	0	0	0	0	0	1	1	1	1
GO:0004708	22	22	100	1.13	0	0	0	0	0	0	0	0	0	1	1	1	1
GO:0004709	18	18	100	1.13	0	0	0	0	0	0	0	0	0	1	1	1	1
GO:0004710	17	17	100	1.13	0	0	0	0	0	0	0	0	0	1	1	1	1
GO:0004711	17	17	100	1.13	0	0	0	0	0	0	0	0	0	1	1	1	1
GO:0004712	19	19	100	1.13	0	0	0	0	0	0	0	0	0	1	1	1	1
GO:0004713	473	433	91.54	1.04	9	1.9	0.54	24	5.07	1.09	7	1.48	0.42	1	1	1	1
GO:0004714	57	44	77.19	0.87	0	0	0	10	17.54	3.78	3	5.26	1.49	1	1	1	1
GO:0004715	17	17	100	1.13	0	0	0	0	0	0	0	0	0	1	1	1	1
GO:0004716	19	19	100	1.13	0	0	0	0	0	0	0	0	0	1	1	1	1
GO:0004717	17	17	100	1.13	0	0	0	0	0	0	0	0	0	1	1	1	1
GO:0004718	22	22	100	1.13	0	0	0	0	0	0	0	0	0	1	1	1	1
GO:0004719	4	4	100	1.13	0	0	0	0	0	0	0	0	0	1	1	1	1
GO:0004720	18	8	44.44	0.5	4	22.22	6.36	6	33.33	7.18	0	0	0	1	1	0.580757432	1
GO:0004721	116	112	96.55	1.09	1	0.86	0.25	0	0	0	3	2.59	0.73	1	1	1	1
GO:0004722	53	50	94.34	1.07	0	0	0	2	3.77	0.81	1	1.89	0.53	1	1	1	1
GO:0004724	2	2	100	1.13	0	0	0	0	0	0	0	0	0	1	1	1	1
GO:0004725	90	86	95.56	1.08	1	1.11	0.32	0	0	0	3	3.33	0.94	1	1	1	1
GO:0004726	5	5	100	1.13	0	0	0	0	0	0	0	0	0	1	1	1	1
GO:0004727	12	11	91.67	1.04	1	8.33	2.39	0	0	0	0	0	0	1	1	1	1
GO:0004729	1	1	100	1.13	0	0	0	0	0	0	0	0	0	1	1	1	1
GO:0004730	18	17	94.44	1.07	0	0	0	1	5.56	1.2	0	0	0	1	1	1	1
GO:0004731	2	2	100	1.13	0	0	0	0	0	0	0	0	0	1	1	1	1
GO:0004733	1	1	100	1.13	0	0	0	0	0	0	0	0	0	1	1	1	1
GO:0004735	3	3	100	1.13	0	0	0	0	0	0	0	0	0	1	1	1	1
GO:0004736	3	3	100	1.13	0	0	0	0	0	0	0	0	0	1	1	1	1
GO:0004738	5	5	100	1.13	0	0	0	0	0	0	0	0	0	1	1	1	1
GO:0004739	8	8	100	1.13	0	0	0	0	0	0	0	0	0	1	1	1	1
GO:0004740	5	5	100	1.13	0	0	0	0	0	0	0	0	0	1	1	1	1
GO:0004741	1	1	100	1.13	0	0	0	0	0	0	0	0	0	1	1	1	1
GO:0004742	1	1	100	1.13	0	0	0	0	0	0	0	0	0	1	1	1	1
GO:0004743	7	7	100	1.13	0	0	0	0	0	0	0	0	0	1	1	1	1
GO:0004745	1	1	100	1.13	0	0	0	0	0	0	0	0	0	1	1	1	1
GO:0004748	8	8	100	1.13	0	0	0	0	0	0	0	0	0	1	1	1	1
GO:0004749	7	7	100	1.13	0	0	0	0	0	0	0	0	0	1	1	1	1
GO:0004750	2	2	100	1.13	0	0	0	0	0	0	0	0	0	1	1	1	1
GO:0004752	4	4	100	1.13	0	0	0	0	0	0	0	0	0	1	1	1	1
GO:0004756	4	4	100	1.13	0	0	0	0	0	0	0	0	0	1	1	1	1
GO:0004757	1	1	100	1.13	0	0	0	0	0	0	0	0	0	1	1	1	1

GO:0004758	8	8	100	1.13	0	0	0	0	0	0	0	0	0	1	1	1	1
GO:0004759	21	14	66.67	0.75	1	4.76	1.36	2	9.52	2.05	4	19.05	5.38	1	1	1	1
GO:0004765	1	1	100	1.13	0	0	0	0	0	0	0	0	0	1	1	1	1
GO:0004766	5	5	100	1.13	0	0	0	0	0	0	0	0	0	1	1	1	1
GO:0004767	6	6	100	1.13	0	0	0	0	0	0	0	0	0	1	1	1	1
GO:0004768	5	5	100	1.13	0	0	0	0	0	0	0	0	0	1	1	1	1
GO:0004769	4	3	75	0.85	0	0	0	0	0	0	1	25	7.06	1	1	1	1
GO:0004771	1	1	100	1.13	0	0	0	0	0	0	0	0	0	1	1	1	1
GO:0004772	3	3	100	1.13	0	0	0	0	0	0	0	0	0	1	1	1	1
GO:0004774	5	5	100	1.13	0	0	0	0	0	0	0	0	0	1	1	1	1
GO:0004776	2	2	100	1.13	0	0	0	0	0	0	0	0	0	1	1	1	1
GO:0004777	2	2	100	1.13	0	0	0	0	0	0	0	0	0	1	1	1	1
GO:0004779	1	1	100	1.13	0	0	0	0	0	0	0	0	0	1	1	1	1
GO:0004781	4	4	100	1.13	0	0	0	0	0	0	0	0	0	1	1	1	1
GO:0004782	1	0	0	0	1	100	28.63	0	0	0	0	0	0	1	1	1	1
GO:0004784	1	1	100	1.13	0	0	0	0	0	0	0	0	0	1	1	1	1
GO:0004785	8	8	100	1.13	0	0	0	0	0	0	0	0	0	1	1	1	1
GO:0004787	1	1	100	1.13	0	0	0	0	0	0	0	0	0	1	1	1	1
GO:0004788	4	4	100	1.13	0	0	0	0	0	0	0	0	0	1	1	1	1
GO:0004790	2	0	0	0	0	0	0	1	50	10.76	1	50	14.11	1	1	1	1
GO:0004791	8	8	100	1.13	0	0	0	0	0	0	0	0	0	1	1	1	1
GO:0004792	3	3	100	1.13	0	0	0	0	0	0	0	0	0	1	1	1	1
GO:0004795	1	1	100	1.13	0	0	0	0	0	0	0	0	0	1	1	1	1
GO:0004796	1	0	0	0	0	0	0	0	0	0	1	100	28.22	1	1	1	1
GO:0004797	3	2	66.67	0.75	1	33.33	9.54	0	0	0	0	0	0	1	1	1	1
GO:0004798	10	10	100	1.13	0	0	0	0	0	0	0	0	0	1	1	1	1
GO:0004799	2	2	100	1.13	0	0	0	0	0	0	0	0	0	1	1	1	1
GO:0004800	2	2	100	1.13	0	0	0	0	0	0	0	0	0	1	1	1	1
GO:0004801	2	2	100	1.13	0	0	0	0	0	0	0	0	0	1	1	1	1
GO:0004802	13	13	100	1.13	0	0	0	0	0	0	0	0	0	1	1	1	1
GO:0004806	4	3	75	0.85	0	0	0	1	25	5.38	0	0	0	1	1	1	1
GO:0004807	3	3	100	1.13	0	0	0	0	0	0	0	0	0	1	1	1	1
GO:0004808	1	1	100	1.13	0	0	0	0	0	0	0	0	0	1	1	1	1
GO:0004809	1	1	100	1.13	0	0	0	0	0	0	0	0	0	1	1	1	1
GO:0004810	1	1	100	1.13	0	0	0	0	0	0	0	0	0	1	1	1	1
GO:0004811	1	1	100	1.13	0	0	0	0	0	0	0	0	0	1	1	1	1
GO:0004812	135	121	89.63	1.01	6	4.44	1.27	6	4.44	0.96	2	1.48	0.42	1	1	1	1
GO:0004813	5	5	100	1.13	0	0	0	0	0	0	0	0	0	1	1	1	1
GO:0004814	5	5	100	1.13	0	0	0	0	0	0	0	0	0	1	1	1	1
GO:0004815	9	6	66.67	0.75	3	33.33	9.54	0	0	0	0	0	0	1	1	1	1
GO:0004816	4	4	100	1.13	0	0	0	0	0	0	0	0	0	1	1	1	1

GO:0004817	3	3	100	1.13	0	0	0	0	0	0	0	0	0	1	1	1	1
GO:0004818	9	9	100	1.13	0	0	0	0	0	0	0	0	0	1	1	1	1
GO:0004819	3	3	100	1.13	0	0	0	0	0	0	0	0	0	1	1	1	1
GO:0004820	2	2	100	1.13	0	0	0	0	0	0	0	0	0	1	1	1	1
GO:0004821	8	8	100	1.13	0	0	0	0	0	0	0	0	0	1	1	1	1
GO:0004822	7	7	100	1.13	0	0	0	0	0	0	0	0	0	1	1	1	1
GO:0004823	2	2	100	1.13	0	0	0	0	0	0	0	0	0	1	1	1	1
GO:0004824	4	1	25	0.28	3	75	21.47	0	0	0	0	0	0	1	0.683729195	1	1
GO:0004825	2	2	100	1.13	0	0	0	0	0	0	0	0	0	1	1	1	1
GO:0004826	9	9	100	1.13	0	0	0	0	0	0	0	0	0	1	1	1	1
GO:0004827	2	2	100	1.13	0	0	0	0	0	0	0	0	0	1	1	1	1
GO:0004828	6	5	83.33	0.94	0	0	0	1	16.67	3.59	0	0	0	1	1	1	1
GO:0004829	13	12	92.31	1.05	0	0	0	1	7.69	1.66	0	0	0	1	1	1	1
GO:0004830	4	4	100	1.13	0	0	0	0	0	0	0	0	0	1	1	1	1
GO:0004831	5	5	100	1.13	0	0	0	0	0	0	0	0	0	1	1	1	1
GO:0004832	2	2	100	1.13	0	0	0	0	0	0	0	0	0	1	1	1	1
GO:0004833	1	0	0	0	0	0	0	0	0	0	1	100	28.22	1	1	1	1
GO:0004835	10	9	90	1.02	0	0	0	1	10	2.15	0	0	0	1	1	1	1
GO:0004838	1	1	100	1.13	0	0	0	0	0	0	0	0	0	1	1	1	1
GO:0004839	14	11	78.57	0.89	0	0	0	3	21.43	4.61	0	0	0	1	1	1	1
GO:0004840	104	101	97.12	1.1	1	0.96	0.28	2	1.92	0.41	0	0	0	1	1	1	1
GO:0004842	108	100	92.59	1.05	1	0.93	0.27	6	5.56	1.2	1	0.93	0.26	1	1	1	1
GO:0004843	25	24	96	1.09	1	4	1.15	0	0	0	0	0	0	1	1	1	1
GO:0004844	3	1	33.33	0.38	0	0	0	2	66.67	14.35	0	0	0	1	1	1	1
GO:0004846	1	0	0	0	0	0	0	0	0	0	1	100	28.22	1	1	1	1
GO:0004849	9	9	100	1.13	0	0	0	0	0	0	0	0	0	1	1	1	1
GO:0004850	1	1	100	1.13	0	0	0	0	0	0	0	0	0	1	1	1	1
GO:0004852	1	1	100	1.13	0	0	0	0	0	0	0	0	0	1	1	1	1
GO:0004853	1	1	100	1.13	0	0	0	0	0	0	0	0	0	1	1	1	1
GO:0004854	3	0	0	0	0	0	0	1	33.33	7.18	2	66.67	18.82	1	1	1	1
GO:0004855	3	0	0	0	0	0	0	1	33.33	7.18	2	66.67	18.82	1	1	1	1
GO:0004857	11	9	81.82	0.93	0	0	0	0	0	0	2	18.18	5.13	1	1	1	1
GO:0004859	5	4	80	0.91	0	0	0	0	0	0	1	20	5.64	1	1	1	1
GO:0004860	13	11	84.62	0.96	0	0	0	0	0	0	2	15.38	4.34	1	1	1	1
GO:0004861	9	7	77.78	0.88	1	11.11	3.18	1	11.11	2.39	0	0	0	1	1	1	1
GO:0004862	4	4	100	1.13	0	0	0	0	0	0	0	0	0	1	1	1	1
GO:0004864	7	6	85.71	0.97	0	0	0	1	14.29	3.08	0	0	0	1	1	1	1
GO:0004865	1	1	100	1.13	0	0	0	0	0	0	0	0	0	1	1	1	1
GO:0004866	29	21	72.41	0.82	2	6.9	1.97	2	6.9	1.48	4	13.79	3.89	1	1	1	1
GO:0004867	78	54	69.23	0.78	5	6.41	1.84	7	8.97	1.93	12	15.38	4.34	1	1	1	0.169399239
GO:0004868	27	14	51.85	0.59	1	3.7	1.06	4	14.81	3.19	8	29.63	8.36	1	1	1	0.021368858

GO:0004869	22	16	72.73	0.82	0	0	0	4	18.18	3.91	2	9.09	2.57	1	1	1	1
GO:0004870	2	2	100	1.13	0	0	0	0	0	0	0	0	0	1	1	1	1
GO:0004871	220	204	92.73	1.05	8	3.64	1.04	5	2.27	0.49	3	1.36	0.38	1	1	1	1
GO:0004872	604	488	80.79	0.91	25	4.14	1.19	48	7.95	1.71	43	7.12	2.01	1	1	1	0.463264602
GO:0004873	1	1	100	1.13	0	0	0	0	0	0	0	0	0	1	1	1	1
GO:0004875	1	1	100	1.13	0	0	0	0	0	0	0	0	0	1	1	1	1
GO:0004879	30	25	83.33	0.94	1	3.33	0.95	2	6.67	1.44	2	6.67	1.88	1	1	1	1
GO:0004882	1	1	100	1.13	0	0	0	0	0	0	0	0	0	1	1	1	1
GO:0004883	1	1	100	1.13	0	0	0	0	0	0	0	0	0	1	1	1	1
GO:0004884	1	1	100	1.13	0	0	0	0	0	0	0	0	0	1	1	1	1
GO:0004887	3	3	100	1.13	0	0	0	0	0	0	0	0	0	1	1	1	1
GO:0004888	72	54	75	0.85	6	8.33	2.39	6	8.33	1.79	6	8.33	2.35	1	1	1	1
GO:0004889	4	4	100	1.13	0	0	0	0	0	0	0	0	0	1	1	1	1
GO:0004890	9	6	66.67	0.75	1	11.11	3.18	0	0	0	2	22.22	6.27	1	1	1	1
GO:0004894	2	2	100	1.13	0	0	0	0	0	0	0	0	0	1	1	1	1
GO:0004895	15	12	80	0.91	1	6.67	1.91	0	0	0	2	13.33	3.76	1	1	1	1
GO:0004896	53	45	84.91	0.96	3	5.66	1.62	3	5.66	1.22	2	3.77	1.07	1	1	1	1
GO:0004905	6	6	100	1.13	0	0	0	0	0	0	0	0	0	1	1	1	1
GO:0004906	1	1	100	1.13	0	0	0	0	0	0	0	0	0	1	1	1	1
GO:0004907	25	19	76	0.86	3	12	3.44	0	0	0	3	12	3.39	1	1	1	1
GO:0004908	5	5	100	1.13	0	0	0	0	0	0	0	0	0	1	1	1	1
GO:0004909	4	3	75	0.85	0	0	0	0	0	0	1	25	7.06	1	1	1	1
GO:0004910	1	1	100	1.13	0	0	0	0	0	0	0	0	0	1	1	1	1
GO:0004915	2	2	100	1.13	0	0	0	0	0	0	0	0	0	1	1	1	1
GO:0004920	1	1	100	1.13	0	0	0	0	0	0	0	0	0	1	1	1	1
GO:0004921	2	2	100	1.13	0	0	0	0	0	0	0	0	0	1	1	1	1
GO:0004926	2	2	100	1.13	0	0	0	0	0	0	0	0	0	1	1	1	1
GO:0004928	5	5	100	1.13	0	0	0	0	0	0	0	0	0	1	1	1	1
GO:0004930	162	116	71.6	0.81	13	8.02	2.3	18	11.11	2.39	15	9.26	2.61	1	1	1	1
GO:0004931	8	8	100	1.13	0	0	0	0	0	0	0	0	0	1	1	1	1
GO:0004935	5	4	80	0.91	0	0	0	0	0	0	1	20	5.64	1	1	1	1
GO:0004937	2	0	0	0	1	50	14.32	0	0	0	1	50	14.11	1	1	1	1
GO:0004938	2	2	100	1.13	0	0	0	0	0	0	0	0	0	1	1	1	1
GO:0004943	1	0	0	0	1	100	28.63	0	0	0	0	0	0	1	1	1	1
GO:0004945	5	4	80	0.91	0	0	0	0	0	0	1	20	5.64	1	1	1	1
GO:0004948	2	2	100	1.13	0	0	0	0	0	0	0	0	0	1	1	1	1
GO:0004949	4	3	75	0.85	0	0	0	1	25	5.38	0	0	0	1	1	1	1
GO:0004950	4	1	25	0.28	0	0	0	2	50	10.76	1	25	7.06	1	1	1	1
GO:0004951	1	1	100	1.13	0	0	0	0	0	0	0	0	0	1	1	1	1
GO:0004957	2	2	100	1.13	0	0	0	0	0	0	0	0	0	1	1	1	1
GO:0004960	2	2	100	1.13	0	0	0	0	0	0	0	0	0	1	1	1	1

GO:0004962	3	3	100	1.13	0	0	0	0	0	0	0	0	0	1	1	1	1
GO:0004964	1	1	100	1.13	0	0	0	0	0	0	0	0	0	1	1	1	1
GO:0004967	1	1	100	1.13	0	0	0	0	0	0	0	0	0	1	1	1	1
GO:0004970	10	9	90	1.02	1	10	2.86	0	0	0	0	0	0	1	1	1	1
GO:0004971	3	3	100	1.13	0	0	0	0	0	0	0	0	0	1	1	1	1
GO:0004972	1	1	100	1.13	0	0	0	0	0	0	0	0	0	1	1	1	1
GO:0004974	2	0	0	0	2	100	28.63	0	0	0	0	0	0	1	1	1	1
GO:0004977	2	2	100	1.13	0	0	0	0	0	0	0	0	0	1	1	1	1
GO:0004982	1	0	0	0	1	100	28.63	0	0	0	0	0	0	1	1	1	1
GO:0004983	2	1	50	0.57	1	50	14.32	0	0	0	0	0	0	1	1	1	1
GO:0004984	1	0	0	0	0	0	0	0	0	0	1	100	28.22	1	1	1	1
GO:0004985	3	3	100	1.13	0	0	0	0	0	0	0	0	0	1	1	1	1
GO:0004990	1	1	100	1.13	0	0	0	0	0	0	0	0	0	1	1	1	1
GO:0004993	1	1	100	1.13	0	0	0	0	0	0	0	0	0	1	1	1	1
GO:0004994	1	1	100	1.13	0	0	0	0	0	0	0	0	0	1	1	1	1
GO:0004995	1	1	100	1.13	0	0	0	0	0	0	0	0	0	1	1	1	1
GO:0004996	1	1	100	1.13	0	0	0	0	0	0	0	0	0	1	1	1	1
GO:0004998	3	3	100	1.13	0	0	0	0	0	0	0	0	0	1	1	1	1
GO:0004999	2	2	100	1.13	0	0	0	0	0	0	0	0	0	1	1	1	1
GO:0005000	2	2	100	1.13	0	0	0	0	0	0	0	0	0	1	1	1	1
GO:0005001	1	0	0	0	1	100	28.63	0	0	0	0	0	0	1	1	1	1
GO:0005003	13	13	100	1.13	0	0	0	0	0	0	0	0	0	1	1	1	1
GO:0005005	4	4	100	1.13	0	0	0	0	0	0	0	0	0	1	1	1	1
GO:0005006	4	4	100	1.13	0	0	0	0	0	0	0	0	0	1	1	1	1
GO:0005007	4	4	100	1.13	0	0	0	0	0	0	0	0	0	1	1	1	1
GO:0005021	4	4	100	1.13	0	0	0	0	0	0	0	0	0	1	1	1	1
GO:0005024	10	10	100	1.13	0	0	0	0	0	0	0	0	0	1	1	1	1
GO:0005027	1	1	100	1.13	0	0	0	0	0	0	0	0	0	1	1	1	1
GO:0005031	4	3	75	0.85	0	0	0	0	0	0	1	25	7.06	1	1	1	1
GO:0005034	2	2	100	1.13	0	0	0	0	0	0	0	0	0	1	1	1	1
GO:0005035	1	1	100	1.13	0	0	0	0	0	0	0	0	0	1	1	1	1
GO:0005037	2	2	100	1.13	0	0	0	0	0	0	0	0	0	1	1	1	1
GO:0005041	12	11	91.67	1.04	0	0	0	0	0	0	1	8.33	2.35	1	1	1	1
GO:0005042	2	2	100	1.13	0	0	0	0	0	0	0	0	0	1	1	1	1
GO:0005044	34	22	64.71	0.73	2	5.88	1.68	6	17.65	3.8	4	11.76	3.32	1	1	1	1
GO:0005045	4	4	100	1.13	0	0	0	0	0	0	0	0	0	1	1	1	1
GO:0005046	3	3	100	1.13	0	0	0	0	0	0	0	0	0	1	1	1	1
GO:0005047	3	3	100	1.13	0	0	0	0	0	0	0	0	0	1	1	1	1
GO:0005048	4	3	75	0.85	1	25	7.16	0	0	0	0	0	0	1	1	1	1
GO:0005049	1	1	100	1.13	0	0	0	0	0	0	0	0	0	1	1	1	1
GO:0005051	2	2	100	1.13	0	0	0	0	0	0	0	0	0	1	1	1	1

GO:0005055	4	4	100	1.13	0	0	0	0	0	0	0	0	0	1	1	1	1
GO:0005057	30	26	86.67	0.98	1	3.33	0.95	3	10	2.15	0	0	0	1	1	1	1
GO:0005061	1	1	100	1.13	0	0	0	0	0	0	0	0	0	1	1	1	1
GO:0005062	2	2	100	1.13	0	0	0	0	0	0	0	0	0	1	1	1	1
GO:0005065	7	6	85.71	0.97	1	14.29	4.09	0	0	0	0	0	0	1	1	1	1
GO:0005066	10	8	80	0.91	0	0	0	2	20	4.31	0	0	0	1	1	1	1
GO:0005068	10	10	100	1.13	0	0	0	0	0	0	0	0	0	1	1	1	1
GO:0005069	3	2	66.67	0.75	0	0	0	1	33.33	7.18	0	0	0	1	1	1	1
GO:0005070	32	28	87.5	0.99	1	3.13	0.89	2	6.25	1.35	1	3.13	0.88	1	1	1	1
GO:0005074	1	1	100	1.13	0	0	0	0	0	0	0	0	0	1	1	1	1
GO:0005075	5	5	100	1.13	0	0	0	0	0	0	0	0	0	1	1	1	1
GO:0005076	1	1	100	1.13	0	0	0	0	0	0	0	0	0	1	1	1	1
GO:0005078	2	2	100	1.13	0	0	0	0	0	0	0	0	0	1	1	1	1
GO:0005079	3	3	100	1.13	0	0	0	0	0	0	0	0	0	1	1	1	1
GO:0005080	16	15	93.75	1.06	0	0	0	1	6.25	1.35	0	0	0	1	1	1	1
GO:0005083	45	40	88.89	1.01	3	6.67	1.91	2	4.44	0.96	0	0	0	1	1	1	1
GO:0005085	69	62	89.86	1.02	3	4.35	1.24	2	2.9	0.62	2	2.9	0.82	1	1	1	1
GO:0005086	8	3	37.5	0.42	0	0	0	5	62.5	13.45	0	0	0	1	1	0.052329147	1
GO:0005087	6	5	83.33	0.94	1	16.67	4.77	0	0	0	0	0	0	1	1	1	1
GO:0005089	5	4	80	0.91	0	0	0	0	0	0	1	20	5.64	1	1	1	1
GO:0005093	3	3	100	1.13	0	0	0	0	0	0	0	0	0	1	1	1	1
GO:0005094	9	6	66.67	0.75	0	0	0	3	33.33	7.18	0	0	0	1	1	1	1
GO:0005095	7	6	85.71	0.97	0	0	0	0	0	0	1	14.29	4.03	1	1	1	1
GO:0005096	135	119	88.15	1	6	4.44	1.27	6	4.44	0.96	4	2.96	0.84	1	1	1	1
GO:0005097	11	10	90.91	1.03	0	0	0	0	0	0	1	9.09	2.57	1	1	1	1
GO:0005098	1	1	100	1.13	0	0	0	0	0	0	0	0	0	1	1	1	1
GO:0005099	11	10	90.91	1.03	0	0	0	0	0	0	1	9.09	2.57	1	1	1	1
GO:0005100	16	16	100	1.13	0	0	0	0	0	0	0	0	0	1	1	1	1
GO:0005102	44	38	86.36	0.98	1	2.27	0.65	2	4.55	0.98	3	6.82	1.92	1	1	1	1
GO:0005104	1	1	100	1.13	0	0	0	0	0	0	0	0	0	1	1	1	1
GO:0005106	2	2	100	1.13	0	0	0	0	0	0	0	0	0	1	1	1	1
GO:0005112	1	1	100	1.13	0	0	0	0	0	0	0	0	0	1	1	1	1
GO:0005113	1	1	100	1.13	0	0	0	0	0	0	0	0	0	1	1	1	1
GO:0005123	3	3	100	1.13	0	0	0	0	0	0	0	0	0	1	1	1	1
GO:0005125	123	86	69.92	0.79	2	1.63	0.47	27	21.95	4.73	8	6.5	1.84	1	1	1.98E-07	1
GO:0005126	1	1	100	1.13	0	0	0	0	0	0	0	0	0	1	1	1	1
GO:0005138	1	0	0	0	0	0	0	1	100	21.53	0	0	0	1	1	1	1
GO:0005139	1	1	100	1.13	0	0	0	0	0	0	0	0	0	1	1	1	1
GO:0005149	11	2	18.18	0.21	1	9.09	2.6	5	45.45	9.78	3	27.27	7.7	1	1	0.380704074	1
GO:0005152	7	2	28.57	0.32	0	0	0	4	57.14	12.3	1	14.29	4.03	1	1	0.663800322	1
GO:0005153	2	2	100	1.13	0	0	0	0	0	0	0	0	0	1	1	1	1

GO:0005154	1	1	100	1.13	0	0	0	0	0	0	0	0	0	1	1	1	1
GO:0005158	5	4	80	0.91	0	0	0	1	20	4.31	0	0	0	1	1	1	1
GO:0005160	3	2	66.67	0.75	1	33.33	9.54	0	0	0	0	0	0	1	1	1	1
GO:0005161	2	1	50	0.57	1	50	14.32	0	0	0	0	0	0	1	1	1	1
GO:0005162	6	4	66.67	0.75	0	0	0	0	0	0	2	33.33	9.41	1	1	1	1
GO:0005164	15	11	73.33	0.83	1	6.67	1.91	3	20	4.31	0	0	0	1	1	1	1
GO:0005172	7	3	42.86	0.49	1	14.29	4.09	3	42.86	9.23	0	0	0	1	1	1	1
GO:0005173	5	5	100	1.13	0	0	0	0	0	0	0	0	0	1	1	1	1
GO:0005177	1	1	100	1.13	0	0	0	0	0	0	0	0	0	1	1	1	1
GO:0005178	18	11	61.11	0.69	0	0	0	5	27.78	5.98	2	11.11	3.14	1	1	1	1
GO:0005179	35	26	74.29	0.84	0	0	0	4	11.43	2.46	5	14.29	4.03	1	1	1	1
GO:0005180	12	11	91.67	1.04	0	0	0	1	8.33	1.79	0	0	0	1	1	1	1
GO:0005181	2	1	50	0.57	0	0	0	0	0	0	1	50	14.11	1	1	1	1
GO:0005184	5	4	80	0.91	0	0	0	0	0	0	1	20	5.64	1	1	1	1
GO:0005187	4	3	75	0.85	1	25	7.16	0	0	0	0	0	0	1	1	1	1
GO:0005189	5	3	60	0.68	0	0	0	0	0	0	2	40	11.29	1	1	1	1
GO:0005192	1	1	100	1.13	0	0	0	0	0	0	0	0	0	1	1	1	1
GO:0005194	50	46	92	1.04	0	0	0	3	6	1.29	1	2	0.56	1	1	1	1
GO:0005198	206	179	86.89	0.98	8	3.88	1.11	12	5.83	1.25	7	3.4	0.96	1	1	1	1
GO:0005200	146	131	89.73	1.02	5	3.42	0.98	7	4.79	1.03	3	2.05	0.58	1	1	1	1
GO:0005201	53	45	84.91	0.96	4	7.55	2.16	3	5.66	1.22	1	1.89	0.53	1	1	1	1
GO:0005202	17	13	76.47	0.87	3	17.65	5.05	1	5.88	1.27	0	0	0	1	1	1	1
GO:0005203	12	8	66.67	0.75	2	16.67	4.77	0	0	0	2	16.67	4.7	1	1	1	1
GO:0005204	2	2	100	1.13	0	0	0	0	0	0	0	0	0	1	1	1	1
GO:0005206	2	2	100	1.13	0	0	0	0	0	0	0	0	0	1	1	1	1
GO:0005207	17	12	70.59	0.8	3	17.65	5.05	1	5.88	1.27	1	5.88	1.66	1	1	1	1
GO:0005208	11	10	90.91	1.03	1	9.09	2.6	0	0	0	0	0	0	1	1	1	1
GO:0005209	13	4	30.77	0.35	3	23.08	6.61	4	30.77	6.62	2	15.38	4.34	1	1	1	1
GO:0005211	8	4	50	0.57	0	0	0	0	0	0	4	50	14.11	1	1	1	0.50470628
GO:0005212	13	12	92.31	1.05	0	0	0	0	0	0	1	7.69	2.17	1	1	1	1
GO:0005215	357	318	89.08	1.01	13	3.64	1.04	15	4.2	0.9	11	3.08	0.87	1	1	1	1
GO:0005216	127	107	84.25	0.95	9	7.09	2.03	2	1.57	0.34	9	7.09	2	1	1	1	1
GO:0005220	2	2	100	1.13	0	0	0	0	0	0	0	0	0	1	1	1	1
GO:0005224	5	5	100	1.13	0	0	0	0	0	0	0	0	0	1	1	1	1
GO:0005229	1	1	100	1.13	0	0	0	0	0	0	0	0	0	1	1	1	1
GO:0005230	14	11	78.57	0.89	1	7.14	2.05	0	0	0	2	14.29	4.03	1	1	1	1
GO:0005234	9	8	88.89	1.01	1	11.11	3.18	0	0	0	0	0	0	1	1	1	1
GO:0005242	8	6	75	0.85	2	25	7.16	0	0	0	0	0	0	1	1	1	1
GO:0005243	9	6	66.67	0.75	1	11.11	3.18	0	0	0	2	22.22	6.27	1	1	1	1
GO:0005244	55	43	78.18	0.89	6	10.91	3.12	2	3.64	0.78	4	7.27	2.05	1	1	1	1
GO:0005245	5	2	40	0.45	3	60	17.18	0	0	0	0	0	0	1	1	1	1

GO:0005247	21	17	80.95	0.92	3	14.29	4.09	1	4.76	1.03	0	0	0	1	1	1	1
GO:0005248	1	1	100	1.13	0	0	0	0	0	0	0	0	0	1	1	1	1
GO:0005249	61	57	93.44	1.06	1	1.64	0.47	2	3.28	0.71	1	1.64	0.46	1	1	1	1
GO:0005251	1	1	100	1.13	0	0	0	0	0	0	0	0	0	1	1	1	1
GO:0005254	4	4	100	1.13	0	0	0	0	0	0	0	0	0	1	1	1	1
GO:0005261	32	31	96.88	1.1	0	0	0	0	0	0	1	3.13	0.88	1	1	1	1
GO:0005262	22	19	86.36	0.98	2	9.09	2.6	0	0	0	1	4.55	1.28	1	1	1	1
GO:0005267	40	37	92.5	1.05	2	5	1.43	0	0	0	1	2.5	0.71	1	1	1	1
GO:0005272	2	2	100	1.13	0	0	0	0	0	0	0	0	0	1	1	1	1
GO:0005279	43	40	93.02	1.05	0	0	0	2	4.65	1	1	2.33	0.66	1	1	1	1
GO:0005280	1	1	100	1.13	0	0	0	0	0	0	0	0	0	1	1	1	1
GO:0005286	3	3	100	1.13	0	0	0	0	0	0	0	0	0	1	1	1	1
GO:0005311	7	6	85.71	0.97	0	0	0	1	14.29	3.08	0	0	0	1	1	1	1
GO:0005313	1	0	0	0	0	0	0	1	100	21.53	0	0	0	1	1	1	1
GO:0005315	1	1	100	1.13	0	0	0	0	0	0	0	0	0	1	1	1	1
GO:0005319	31	25	80.65	0.91	0	0	0	3	9.68	2.08	3	9.68	2.73	1	1	1	1
GO:0005320	4	2	50	0.57	0	0	0	2	50	10.76	0	0	0	1	1	1	1
GO:0005328	11	9	81.82	0.93	2	18.18	5.21	0	0	0	0	0	0	1	1	1	1
GO:0005329	1	1	100	1.13	0	0	0	0	0	0	0	0	0	1	1	1	1
GO:0005330	1	1	100	1.13	0	0	0	0	0	0	0	0	0	1	1	1	1
GO:0005331	5	5	100	1.13	0	0	0	0	0	0	0	0	0	1	1	1	1
GO:0005332	6	5	83.33	0.94	1	16.67	4.77	0	0	0	0	0	0	1	1	1	1
GO:0005337	9	9	100	1.13	0	0	0	0	0	0	0	0	0	1	1	1	1
GO:0005338	5	5	100	1.13	0	0	0	0	0	0	0	0	0	1	1	1	1
GO:0005344	14	5	35.71	0.4	8	57.14	16.36	0	0	0	1	7.14	2.02	1	2.86E-05	1	1
GO:0005351	38	33	86.84	0.98	3	7.89	2.26	0	0	0	2	5.26	1.49	1	1	1	1
GO:0005355	3	1	33.33	0.38	1	33.33	9.54	0	0	0	1	33.33	9.41	1	1	1	1
GO:0005360	1	1	100	1.13	0	0	0	0	0	0	0	0	0	1	1	1	1
GO:0005372	1	1	100	1.13	0	0	0	0	0	0	0	0	0	1	1	1	1
GO:0005375	6	6	100	1.13	0	0	0	0	0	0	0	0	0	1	1	1	1
GO:0005377	1	1	100	1.13	0	0	0	0	0	0	0	0	0	1	1	1	1
GO:0005381	6	6	100	1.13	0	0	0	0	0	0	0	0	0	1	1	1	1
GO:0005385	7	7	100	1.13	0	0	0	0	0	0	0	0	0	1	1	1	1
GO:0005386	95	79	83.16	0.94	3	3.16	0.9	7	7.37	1.59	6	6.32	1.78	1	1	1	1
GO:0005388	9	8	88.89	1.01	0	0	0	0	0	0	1	11.11	3.14	1	1	1	1
GO:0005391	11	11	100	1.13	0	0	0	0	0	0	0	0	0	1	1	1	1
GO:0005427	3	1	33.33	0.38	1	33.33	9.54	1	33.33	7.18	0	0	0	1	1	1	1
GO:0005432	6	6	100	1.13	0	0	0	0	0	0	0	0	0	1	1	1	1
GO:0005436	1	0	0	0	0	0	0	0	0	0	1	100	28.22	1	1	1	1
GO:0005452	11	11	100	1.13	0	0	0	0	0	0	0	0	0	1	1	1	1
GO:0005459	4	4	100	1.13	0	0	0	0	0	0	0	0	0	1	1	1	1

GO:0005468	3	3	100	1.13	0	0	0	0	0	0	0	0	0	1	1	1	1
GO:0005471	7	7	100	1.13	0	0	0	0	0	0	0	0	0	1	1	1	1
GO:0005478	14	14	100	1.13	0	0	0	0	0	0	0	0	0	1	1	1	1
GO:0005480	18	18	100	1.13	0	0	0	0	0	0	0	0	0	1	1	1	1
GO:0005482	9	9	100	1.13	0	0	0	0	0	0	0	0	0	1	1	1	1
GO:0005484	1	1	100	1.13	0	0	0	0	0	0	0	0	0	1	1	1	1
GO:0005486	4	4	100	1.13	0	0	0	0	0	0	0	0	0	1	1	1	1
GO:0005487	16	16	100	1.13	0	0	0	0	0	0	0	0	0	1	1	1	1
GO:0005488	135	124	91.85	1.04	2	1.48	0.42	5	3.7	0.8	4	2.96	0.84	1	1	1	1
GO:0005489	428	399	93.22	1.06	9	2.1	0.6	11	2.57	0.55	9	2.1	0.59	0.24149745	1	1	1
GO:0005496	26	23	88.46	1	1	3.85	1.1	1	3.85	0.83	1	3.85	1.09	1	1	1	1
GO:0005498	10	9	90	1.02	0	0	0	1	10	2.15	0	0	0	1	1	1	1
GO:0005499	1	0	0	0	1	100	28.63	0	0	0	0	0	0	1	1	1	1
GO:0005501	3	2	66.67	0.75	0	0	0	1	33.33	7.18	0	0	0	1	1	1	1
GO:0005504	5	4	80	0.91	0	0	0	1	20	4.31	0	0	0	1	1	1	1
GO:0005505	14	14	100	1.13	0	0	0	0	0	0	0	0	0	1	1	1	1
GO:0005506	35	34	97.14	1.1	0	0	0	1	2.86	0.62	0	0	0	1	1	1	1
GO:0005507	52	41	78.85	0.89	4	7.69	2.2	7	13.46	2.9	0	0	0	1	1	1	1
GO:0005509	621	535	86.15	0.98	18	2.9	0.83	32	5.15	1.11	36	5.8	1.64	1	1	1	1
GO:0005514	10	9	90	1.02	0	0	0	1	10	2.15	0	0	0	1	1	1	1
GO:0005515	1590	1433	90.13	1.02	33	2.08	0.59	74	4.65	1	50	3.14	0.89	1	1	1	1
GO:0005516	83	73	87.95	1	3	3.61	1.03	3	3.61	0.78	4	4.82	1.36	1	1	1	1
GO:0005518	2	2	100	1.13	0	0	0	0	0	0	0	0	0	1	1	1	1
GO:0005519	11	11	100	1.13	0	0	0	0	0	0	0	0	0	1	1	1	1
GO:0005520	17	14	82.35	0.93	2	11.76	3.37	0	0	0	1	5.88	1.66	1	1	1	1
GO:0005521	1	1	100	1.13	0	0	0	0	0	0	0	0	0	1	1	1	1
GO:0005522	4	3	75	0.85	0	0	0	1	25	5.38	0	0	0	1	1	1	1
GO:0005523	7	6	85.71	0.97	0	0	0	0	0	0	1	14.29	4.03	1	1	1	1
GO:0005524	1798	1655	92.05	1.04	46	2.56	0.73	67	3.73	0.8	30	1.67	0.47	4.48E-07	1	1	1
GO:0005525	527	498	94.5	1.07	11	2.09	0.6	12	2.28	0.49	6	1.14	0.32	0.000162377	1	1	1
GO:0005528	13	12	92.31	1.05	0	0	0	0	0	0	1	7.69	2.17	1	1	1	1
GO:0005529	88	66	75	0.85	3	3.41	0.98	7	7.95	1.71	12	13.64	3.85	1	1	1	0.560080948
GO:0005530	12	9	75	0.85	0	0	0	1	8.33	1.79	2	16.67	4.7	1	1	1	1
GO:0005531	6	5	83.33	0.94	0	0	0	1	16.67	3.59	0	0	0	1	1	1	1
GO:0005532	2	1	50	0.57	0	0	0	1	50	10.76	0	0	0	1	1	1	1
GO:0005537	4	2	50	0.57	0	0	0	1	25	5.38	1	25	7.06	1	1	1	1
GO:0005540	13	9	69.23	0.78	1	7.69	2.2	3	23.08	4.97	0	0	0	1	1	1	1
GO:0005542	6	6	100	1.13	0	0	0	0	0	0	0	0	0	1	1	1	1
GO:0005543	4	2	50	0.57	1	25	7.16	1	25	5.38	0	0	0	1	1	1	1
GO:0005544	48	46	95.83	1.09	0	0	0	1	2.08	0.45	1	2.08	0.59	1	1	1	1
GO:0005545	4	4	100	1.13	0	0	0	0	0	0	0	0	0	1	1	1	1

GO:0005548	1	1	100	1.13	0	0	0	0	0	0	0	0	0	1	1	1	1
GO:0005549	1	0	0	0	0	0	0	1	100	21.53	0	0	0	1	1	1	1
GO:0005551	1	0	0	0	0	0	0	1	100	21.53	0	0	0	1	1	1	1
GO:0005554	234	211	90.17	1.02	5	2.14	0.61	14	5.98	1.29	4	1.71	0.48	1	1	1	1
GO:0005555	1	1	100	1.13	0	0	0	0	0	0	0	0	0	1	1	1	1
GO:0005557	25	13	52	0.59	3	12	3.44	4	16	3.44	5	20	5.64	1	1	1	1
GO:0005558	7	6	85.71	0.97	0	0	0	0	0	0	1	14.29	4.03	1	1	1	1
GO:0005576	257	183	71.21	0.81	10	3.89	1.11	30	11.67	2.51	34	13.23	3.73	1	1	0.047933431	2.09E-06
GO:0005577	1	0	0	0	0	0	0	0	0	0	1	100	28.22	1	1	1	1
GO:0005578	174	138	79.31	0.9	11	6.32	1.81	16	9.2	1.98	9	5.17	1.46	1	1	1	1
GO:0005579	6	0	0	0	0	0	0	0	0	0	6	100	28.22	1	1	1	1.24E-05
GO:0005581	31	24	77.42	0.88	2	6.45	1.85	5	16.13	3.47	0	0	0	1	1	1	1
GO:0005588	1	0	0	0	1	100	28.63	0	0	0	0	0	0	1	1	1	1
GO:0005594	1	1	100	1.13	0	0	0	0	0	0	0	0	0	1	1	1	1
GO:0005597	1	1	100	1.13	0	0	0	0	0	0	0	0	0	1	1	1	1
GO:0005602	1	1	100	1.13	0	0	0	0	0	0	0	0	0	1	1	1	1
GO:0005604	29	29	100	1.13	0	0	0	0	0	0	0	0	0	1	1	1	1
GO:0005605	9	9	100	1.13	0	0	0	0	0	0	0	0	0	1	1	1	1
GO:0005615	115	94	81.74	0.93	3	2.61	0.75	11	9.57	2.06	7	6.09	1.72	1	1	1	1
GO:0005622	931	819	87.97	1	31	3.33	0.95	64	6.87	1.48	17	1.83	0.52	1	1	1	1
GO:0005623	2	2	100	1.13	0	0	0	0	0	0	0	0	0	1	1	1	1
GO:0005624	931	797	85.61	0.97	41	4.4	1.26	38	4.08	0.88	55	5.91	1.67	1	1	1	1
GO:0005625	75	70	93.33	1.06	2	2.67	0.76	3	4	0.86	0	0	0	1	1	1	1
GO:0005626	4	4	100	1.13	0	0	0	0	0	0	0	0	0	1	1	1	1
GO:0005634	3168	2905	91.7	1.04	107	3.38	0.97	107	3.38	0.73	49	1.55	0.44	6.15E-12	1	1	1
GO:0005634,	1	1	100	1.13	0	0	0	0	0	0	0	0	0	1	1	1	1
GO:0005635	31	29	93.55	1.06	0	0	0	1	3.23	0.69	1	3.23	0.91	1	1	1	1
GO:0005637	9	9	100	1.13	0	0	0	0	0	0	0	0	0	1	1	1	1
GO:0005638	15	15	100	1.13	0	0	0	0	0	0	0	0	0	1	1	1	1
GO:0005639	1	1	100	1.13	0	0	0	0	0	0	0	0	0	1	1	1	1
GO:0005640	5	5	100	1.13	0	0	0	0	0	0	0	0	0	1	1	1	1
GO:0005643	55	54	98.18	1.11	0	0	0	1	1.82	0.39	0	0	0	1	1	1	1
GO:0005645	1	1	100	1.13	0	0	0	0	0	0	0	0	0	1	1	1	1
GO:0005648	10	10	100	1.13	0	0	0	0	0	0	0	0	0	1	1	1	1
GO:0005652	10	10	100	1.13	0	0	0	0	0	0	0	0	0	1	1	1	1
GO:0005653	16	16	100	1.13	0	0	0	0	0	0	0	0	0	1	1	1	1
GO:0005654	46	41	89.13	1.01	2	4.35	1.24	2	4.35	0.94	1	2.17	0.61	1	1	1	1
GO:0005655	7	7	100	1.13	0	0	0	0	0	0	0	0	0	1	1	1	1
GO:0005657	5	5	100	1.13	0	0	0	0	0	0	0	0	0	1	1	1	1
GO:0005658	3	3	100	1.13	0	0	0	0	0	0	0	0	0	1	1	1	1
GO:0005659	4	4	100	1.13	0	0	0	0	0	0	0	0	0	1	1	1	1

GO:0005660	3	3	100	1.13	0	0	0	0	0	0	0	0	0	1	1	1	1
GO:0005662	2	2	100	1.13	0	0	0	0	0	0	0	0	0	1	1	1	1
GO:0005663	9	9	100	1.13	0	0	0	0	0	0	0	0	0	1	1	1	1
GO:0005664	3	3	100	1.13	0	0	0	0	0	0	0	0	0	1	1	1	1
GO:0005665	19	17	89.47	1.01	0	0	0	2	10.53	2.27	0	0	0	1	1	1	1
GO:0005666	9	9	100	1.13	0	0	0	0	0	0	0	0	0	1	1	1	1
GO:0005667	261	229	87.74	0.99	11	4.21	1.21	9	3.45	0.74	12	4.6	1.3	1	1	1	1
GO:0005669	23	22	95.65	1.08	0	0	0	1	4.35	0.94	0	0	0	1	1	1	1
GO:0005672	2	2	100	1.13	0	0	0	0	0	0	0	0	0	1	1	1	1
GO:0005673	5	5	100	1.13	0	0	0	0	0	0	0	0	0	1	1	1	1
GO:0005674	2	1	50	0.57	1	50	14.32	0	0	0	0	0	0	1	1	1	1
GO:0005675	6	4	66.67	0.75	0	0	0	0	0	0	2	33.33	9.41	1	1	1	1
GO:0005677	3	2	66.67	0.75	0	0	0	1	33.33	7.18	0	0	0	1	1	1	1
GO:0005678	6	6	100	1.13	0	0	0	0	0	0	0	0	0	1	1	1	1
GO:0005679	7	7	100	1.13	0	0	0	0	0	0	0	0	0	1	1	1	1
GO:0005680	7	5	71.43	0.81	0	0	0	2	28.57	6.15	0	0	0	1	1	1	1
GO:0005681	90	84	93.33	1.06	2	2.22	0.64	3	3.33	0.72	1	1.11	0.31	1	1	1	1
GO:0005682	5	5	100	1.13	0	0	0	0	0	0	0	0	0	1	1	1	1
GO:0005685	1	1	100	1.13	0	0	0	0	0	0	0	0	0	1	1	1	1
GO:0005686	4	4	100	1.13	0	0	0	0	0	0	0	0	0	1	1	1	1
GO:0005694	26	21	80.77	0.91	0	0	0	3	11.54	2.48	2	7.69	2.17	1	1	1	1
GO:0005696	4	4	100	1.13	0	0	0	0	0	0	0	0	0	1	1	1	1
GO:0005698	6	6	100	1.13	0	0	0	0	0	0	0	0	0	1	1	1	1
GO:0005699	7	7	100	1.13	0	0	0	0	0	0	0	0	0	1	1	1	1
GO:0005711	1	1	100	1.13	0	0	0	0	0	0	0	0	0	1	1	1	1
GO:0005716	6	6	100	1.13	0	0	0	0	0	0	0	0	0	1	1	1	1
GO:0005717	33	29	87.88	1	3	9.09	2.6	1	3.03	0.65	0	0	0	1	1	1	1
GO:0005718	47	40	85.11	0.96	1	2.13	0.61	5	10.64	2.29	1	2.13	0.6	1	1	1	1
GO:0005720	6	6	100	1.13	0	0	0	0	0	0	0	0	0	1	1	1	1
GO:0005721	1	1	100	1.13	0	0	0	0	0	0	0	0	0	1	1	1	1
GO:0005727	6	6	100	1.13	0	0	0	0	0	0	0	0	0	1	1	1	1
GO:0005730	85	82	96.47	1.09	0	0	0	2	2.35	0.51	1	1.18	0.33	1	1	1	1
GO:0005732	35	35	100	1.13	0	0	0	0	0	0	0	0	0	1	1	1	1
GO:0005735	2	2	100	1.13	0	0	0	0	0	0	0	0	0	1	1	1	1
GO:0005736	4	4	100	1.13	0	0	0	0	0	0	0	0	0	1	1	1	1
GO:0005737	884	821	92.87	1.05	26	2.94	0.84	22	2.49	0.54	15	1.7	0.48	0.000325324	1	1	1
GO:0005739	1206	1130	93.7	1.06	21	1.74	0.5	42	3.48	0.75	13	1.08	0.3	1.23E-09	1	1	1
GO:0005740	24	24	100	1.13	0	0	0	0	0	0	0	0	0	1	1	1	1
GO:0005741	61	57	93.44	1.06	2	3.28	0.94	1	1.64	0.35	1	1.64	0.46	1	1	1	1
GO:0005743	143	132	92.31	1.05	2	1.4	0.4	7	4.9	1.05	2	1.4	0.39	1	1	1	1
GO:0005744	19	17	89.47	1.01	0	0	0	2	10.53	2.27	0	0	0	1	1	1	1

GO:0005746	30	26	86.67	0.98	1	3.33	0.95	3	10	2.15	0	0	0	1	1	1	1
GO:0005747	3	3	100	1.13	0	0	0	0	0	0	0	0	0	1	1	1	1
GO:0005748	35	32	91.43	1.04	1	2.86	0.82	1	2.86	0.62	1	2.86	0.81	1	1	1	1
GO:0005751	7	5	71.43	0.81	0	0	0	2	28.57	6.15	0	0	0	1	1	1	1
GO:0005752	19	19	100	1.13	0	0	0	0	0	0	0	0	0	1	1	1	1
GO:0005753	36	31	86.11	0.98	1	2.78	0.8	4	11.11	2.39	0	0	0	1	1	1	1
GO:0005758	7	7	100	1.13	0	0	0	0	0	0	0	0	0	1	1	1	1
GO:0005759	19	16	84.21	0.95	0	0	0	2	10.53	2.27	1	5.26	1.49	1	1	1	1
GO:0005760	2	2	100	1.13	0	0	0	0	0	0	0	0	0	1	1	1	1
GO:0005761	19	19	100	1.13	0	0	0	0	0	0	0	0	0	1	1	1	1
GO:0005762	36	33	91.67	1.04	0	0	0	3	8.33	1.79	0	0	0	1	1	1	1
GO:0005763	34	34	100	1.13	0	0	0	0	0	0	0	0	0	1	1	1	1
GO:0005764	259	225	86.87	0.98	11	4.25	1.22	11	4.25	0.91	12	4.63	1.31	1	1	1	1
GO:0005765	5	4	80	0.91	0	0	0	1	20	4.31	0	0	0	1	1	1	1
GO:0005768	43	43	100	1.13	0	0	0	0	0	0	0	0	0	1	1	1	1
GO:0005769	28	27	96.43	1.09	0	0	0	1	3.57	0.77	0	0	0	1	1	1	1
GO:0005770	25	24	96	1.09	0	0	0	1	4	0.86	0	0	0	1	1	1	1
GO:0005771	9	7	77.78	0.88	0	0	0	2	22.22	4.78	0	0	0	1	1	1	1
GO:0005773	4	4	100	1.13	0	0	0	0	0	0	0	0	0	1	1	1	1
GO:0005776	2	2	100	1.13	0	0	0	0	0	0	0	0	0	1	1	1	1
GO:0005777	99	88	88.89	1.01	4	4.04	1.16	0	0	0	7	7.07	2	1	1	1	1
GO:0005778	7	7	100	1.13	0	0	0	0	0	0	0	0	0	1	1	1	1
GO:0005779	5	5	100	1.13	0	0	0	0	0	0	0	0	0	1	1	1	1
GO:0005782	6	6	100	1.13	0	0	0	0	0	0	0	0	0	1	1	1	1
GO:0005783	495	455	91.92	1.04	10	2.02	0.58	13	2.63	0.57	17	3.43	0.97	1	1	1	1
GO:0005784	5	5	100	1.13	0	0	0	0	0	0	0	0	0	1	1	1	1
GO:0005785	2	2	100	1.13	0	0	0	0	0	0	0	0	0	1	1	1	1
GO:0005786	26	26	100	1.13	0	0	0	0	0	0	0	0	0	1	1	1	1
GO:0005788	7	7	100	1.13	0	0	0	0	0	0	0	0	0	1	1	1	1
GO:0005789	38	35	92.11	1.04	3	7.89	2.26	0	0	0	0	0	0	1	1	1	1
GO:0005790	3	3	100	1.13	0	0	0	0	0	0	0	0	0	1	1	1	1
GO:0005792	114	90	78.95	0.89	2	1.75	0.5	7	6.14	1.32	15	13.16	3.71	1	1	1	0.136029022
GO:0005793	7	7	100	1.13	0	0	0	0	0	0	0	0	0	1	1	1	1
GO:0005794	441	432	97.96	1.11	2	0.45	0.13	5	1.13	0.24	2	0.45	0.13	2.84E-12	1	1	1
GO:0005795	25	25	100	1.13	0	0	0	0	0	0	0	0	0	1	1	1	1
GO:0005796	2	2	100	1.13	0	0	0	0	0	0	0	0	0	1	1	1	1
GO:0005798	1	1	100	1.13	0	0	0	0	0	0	0	0	0	1	1	1	1
GO:0005799	1	1	100	1.13	0	0	0	0	0	0	0	0	0	1	1	1	1
GO:0005800	1	1	100	1.13	0	0	0	0	0	0	0	0	0	1	1	1	1
GO:0005801	3	3	100	1.13	0	0	0	0	0	0	0	0	0	1	1	1	1
GO:0005802	29	29	100	1.13	0	0	0	0	0	0	0	0	0	1	1	1	1

GO:0005803	12	12	100	1.13	0	0	0	0	0	0	0	0	0	1	1	1	1
GO:0005804	1	0	0	0	1	100	28.63	0	0	0	0	0	0	1	1	1	1
GO:0005811	8	8	100	1.13	0	0	0	0	0	0	0	0	0	1	1	1	1
GO:0005813	32	32	100	1.13	0	0	0	0	0	0	0	0	0	1	1	1	1
GO:0005814	6	5	83.33	0.94	1	16.67	4.77	0	0	0	0	0	0	1	1	1	1
GO:0005815	1	1	100	1.13	0	0	0	0	0	0	0	0	0	1	1	1	1
GO:0005816	2	2	100	1.13	0	0	0	0	0	0	0	0	0	1	1	1	1
GO:0005819	6	6	100	1.13	0	0	0	0	0	0	0	0	0	1	1	1	1
GO:0005827	1	1	100	1.13	0	0	0	0	0	0	0	0	0	1	1	1	1
GO:0005829	267	255	95.51	1.08	0	0	0	8	3	0.65	4	1.5	0.42	0.022770101	1	1	1
GO:0005830	50	41	82	0.93	3	6	1.72	6	12	2.58	0	0	0	1	1	1	1
GO:0005831	3	3	100	1.13	0	0	0	0	0	0	0	0	0	1	1	1	1
GO:0005833	11	2	18.18	0.21	8	72.73	20.82	0	0	0	1	9.09	2.57	1	1.74E-06	1	1
GO:0005834	43	39	90.7	1.03	3	6.98	2	1	2.33	0.5	0	0	0	1	1	1	1
GO:0005837	13	11	84.62	0.96	0	0	0	2	15.38	3.31	0	0	0	1	1	1	1
GO:0005838	17	16	94.12	1.07	0	0	0	1	5.88	1.27	0	0	0	1	1	1	1
GO:0005839	47	43	91.49	1.04	0	0	0	4	8.51	1.83	0	0	0	1	1	1	1
GO:0005840	262	231	88.17	1	10	3.82	1.09	18	6.87	1.48	3	1.15	0.32	1	1	1	1
GO:0005842	24	24	100	1.13	0	0	0	0	0	0	0	0	0	1	1	1	1
GO:0005843	28	26	92.86	1.05	0	0	0	2	7.14	1.54	0	0	0	1	1	1	1
GO:0005844	3	2	66.67	0.75	0	0	0	0	0	0	1	33.33	9.41	1	1	1	1
GO:0005850	1	1	100	1.13	0	0	0	0	0	0	0	0	0	1	1	1	1
GO:0005851	7	7	100	1.13	0	0	0	0	0	0	0	0	0	1	1	1	1
GO:0005852	16	13	81.25	0.92	0	0	0	3	18.75	4.04	0	0	0	1	1	1	1
GO:0005853	9	9	100	1.13	0	0	0	0	0	0	0	0	0	1	1	1	1
GO:0005856	357	327	91.6	1.04	10	2.8	0.8	13	3.64	0.78	7	1.96	0.55	1	1	1	1
GO:0005858	5	5	100	1.13	0	0	0	0	0	0	0	0	0	1	1	1	1
GO:0005859	5	5	100	1.13	0	0	0	0	0	0	0	0	0	1	1	1	1
GO:0005860	1	1	100	1.13	0	0	0	0	0	0	0	0	0	1	1	1	1
GO:0005861	3	3	100	1.13	0	0	0	0	0	0	0	0	0	1	1	1	1
GO:0005862	6	6	100	1.13	0	0	0	0	0	0	0	0	0	1	1	1	1
GO:0005863	3	2	66.67	0.75	0	0	0	1	33.33	7.18	0	0	0	1	1	1	1
GO:0005865	1	1	100	1.13	0	0	0	0	0	0	0	0	0	1	1	1	1
GO:0005868	15	15	100	1.13	0	0	0	0	0	0	0	0	0	1	1	1	1
GO:0005869	13	13	100	1.13	0	0	0	0	0	0	0	0	0	1	1	1	1
GO:0005871	31	30	96.77	1.1	1	3.23	0.92	0	0	0	0	0	0	1	1	1	1
GO:0005874	40	34	85	0.96	2	5	1.43	1	2.5	0.54	3	7.5	2.12	1	1	1	1
GO:0005875	79	75	94.94	1.07	2	2.53	0.72	2	2.53	0.54	0	0	0	1	1	1	1
GO:0005876	2	1	50	0.57	1	50	14.32	0	0	0	0	0	0	1	1	1	1
GO:0005881	2	1	50	0.57	0	0	0	0	0	0	1	50	14.11	1	1	1	1
GO:0005882	63	54	85.71	0.97	0	0	0	9	14.29	3.08	0	0	0	1	1	1	1

GO:0005883	2	2	100	1.13	0	0	0	0	0	0	0	0	0	1	1	1	1
GO:0005884	28	27	96.43	1.09	0	0	0	0	0	0	1	3.57	1.01	1	1	1	1
GO:0005885	39	39	100	1.13	0	0	0	0	0	0	0	0	0	1	1	1	1
GO:0005886	382	316	82.72	0.94	13	3.4	0.97	23	6.02	1.3	30	7.85	2.22	1	1	1	0.911489533
GO:0005887	486	408	83.95	0.95	24	4.94	1.41	23	4.73	1.02	31	6.38	1.8	1	1	1	1
GO:0005888	3	3	100	1.13	0	0	0	0	0	0	0	0	0	1	1	1	1
GO:0005889	2	2	100	1.13	0	0	0	0	0	0	0	0	0	1	1	1	1
GO:0005890	11	11	100	1.13	0	0	0	0	0	0	0	0	0	1	1	1	1
GO:0005891	6	3	50	0.57	3	50	14.32	0	0	0	0	0	0	1	1	1	1
GO:0005896	2	2	100	1.13	0	0	0	0	0	0	0	0	0	1	1	1	1
GO:0005901	12	10	83.33	0.94	1	8.33	2.39	1	8.33	1.79	0	0	0	1	1	1	1
GO:0005902	4	3	75	0.85	0	0	0	1	25	5.38	0	0	0	1	1	1	1
GO:0005903	4	3	75	0.85	0	0	0	1	25	5.38	0	0	0	1	1	1	1
GO:0005905	43	42	97.67	1.11	0	0	0	1	2.33	0.5	0	0	0	1	1	1	1
GO:0005906	14	13	92.86	1.05	0	0	0	1	7.14	1.54	0	0	0	1	1	1	1
GO:0005909	4	4	100	1.13	0	0	0	0	0	0	0	0	0	1	1	1	1
GO:0005911	8	8	100	1.13	0	0	0	0	0	0	0	0	0	1	1	1	1
GO:0005912	12	12	100	1.13	0	0	0	0	0	0	0	0	0	1	1	1	1
GO:0005913	4	4	100	1.13	0	0	0	0	0	0	0	0	0	1	1	1	1
GO:0005915	6	6	100	1.13	0	0	0	0	0	0	0	0	0	1	1	1	1
GO:0005916	1	0	0	0	0	0	0	1	100	21.53	0	0	0	1	1	1	1
GO:0005918	1	1	100	1.13	0	0	0	0	0	0	0	0	0	1	1	1	1
GO:0005921	11	8	72.73	0.82	1	9.09	2.6	0	0	0	2	18.18	5.13	1	1	1	1
GO:0005922	9	6	66.67	0.75	1	11.11	3.18	0	0	0	2	22.22	6.27	1	1	1	1
GO:0005923	25	23	92	1.04	1	4	1.15	0	0	0	1	4	1.13	1	1	1	1
GO:0005925	11	9	81.82	0.93	1	9.09	2.6	1	9.09	1.96	0	0	0	1	1	1	1
GO:0005929	7	7	100	1.13	0	0	0	0	0	0	0	0	0	1	1	1	1
GO:0005930	2	2	100	1.13	0	0	0	0	0	0	0	0	0	1	1	1	1
GO:0005932	2	2	100	1.13	0	0	0	0	0	0	0	0	0	1	1	1	1
GO:0005938	12	12	100	1.13	0	0	0	0	0	0	0	0	0	1	1	1	1
GO:0005942	16	15	93.75	1.06	1	6.25	1.79	0	0	0	0	0	0	1	1	1	1
GO:0005945	9	9	100	1.13	0	0	0	0	0	0	0	0	0	1	1	1	1
GO:0005952	3	1	33.33	0.38	0	0	0	1	33.33	7.18	1	33.33	9.41	1	1	1	1
GO:0005954	4	4	100	1.13	0	0	0	0	0	0	0	0	0	1	1	1	1
GO:0005956	2	2	100	1.13	0	0	0	0	0	0	0	0	0	1	1	1	1
GO:0005960	1	1	100	1.13	0	0	0	0	0	0	0	0	0	1	1	1	1
GO:0005962	2	2	100	1.13	0	0	0	0	0	0	0	0	0	1	1	1	1
GO:0005964	7	7	100	1.13	0	0	0	0	0	0	0	0	0	1	1	1	1
GO:0005967	2	2	100	1.13	0	0	0	0	0	0	0	0	0	1	1	1	1
GO:0005971	8	8	100	1.13	0	0	0	0	0	0	0	0	0	1	1	1	1
GO:0005975	222	205	92.34	1.05	4	1.8	0.52	4	1.8	0.39	9	4.05	1.14	1	1	1	1

GO:0005977	37	37	100	1.13	0	0	0	0	0	0	0	0	0	1	1	1	1
GO:0005978	12	10	83.33	0.94	0	0	0	2	16.67	3.59	0	0	0	1	1	1	1
GO:0005980	6	6	100	1.13	0	0	0	0	0	0	0	0	0	1	1	1	1
GO:0005991	1	1	100	1.13	0	0	0	0	0	0	0	0	0	1	1	1	1
GO:0005993	1	1	100	1.13	0	0	0	0	0	0	0	0	0	1	1	1	1
GO:0005997	2	1	50	0.57	0	0	0	1	50	10.76	0	0	0	1	1	1	1
GO:0006002	6	6	100	1.13	0	0	0	0	0	0	0	0	0	1	1	1	1
GO:0006003	4	4	100	1.13	0	0	0	0	0	0	0	0	0	1	1	1	1
GO:0006004	5	5	100	1.13	0	0	0	0	0	0	0	0	0	1	1	1	1
GO:0006006	27	26	96.3	1.09	1	3.7	1.06	0	0	0	0	0	0	1	1	1	1
GO:0006007	2	2	100	1.13	0	0	0	0	0	0	0	0	0	1	1	1	1
GO:0006011	1	1	100	1.13	0	0	0	0	0	0	0	0	0	1	1	1	1
GO:0006012	11	8	72.73	0.82	1	9.09	2.6	1	9.09	1.96	1	9.09	2.57	1	1	1	1
GO:0006013	3	3	100	1.13	0	0	0	0	0	0	0	0	0	1	1	1	1
GO:0006020	15	15	100	1.13	0	0	0	0	0	0	0	0	0	1	1	1	1
GO:0006021	2	2	100	1.13	0	0	0	0	0	0	0	0	0	1	1	1	1
GO:0006024	2	2	100	1.13	0	0	0	0	0	0	0	0	0	1	1	1	1
GO:0006027	5	4	80	0.91	0	0	0	1	20	4.31	0	0	0	1	1	1	1
GO:0006029	2	2	100	1.13	0	0	0	0	0	0	0	0	0	1	1	1	1
GO:0006032	1	0	0	0	0	0	0	0	0	0	1	100	28.22	1	1	1	1
GO:0006041	4	4	100	1.13	0	0	0	0	0	0	0	0	0	1	1	1	1
GO:0006044	9	9	100	1.13	0	0	0	0	0	0	0	0	0	1	1	1	1
GO:0006048	9	9	100	1.13	0	0	0	0	0	0	0	0	0	1	1	1	1
GO:0006054	2	2	100	1.13	0	0	0	0	0	0	0	0	0	1	1	1	1
GO:0006055	1	1	100	1.13	0	0	0	0	0	0	0	0	0	1	1	1	1
GO:0006060	1	1	100	1.13	0	0	0	0	0	0	0	0	0	1	1	1	1
GO:0006066	2	2	100	1.13	0	0	0	0	0	0	0	0	0	1	1	1	1
GO:0006069	2	2	100	1.13	0	0	0	0	0	0	0	0	0	1	1	1	1
GO:0006071	12	12	100	1.13	0	0	0	0	0	0	0	0	0	1	1	1	1
GO:0006072	8	7	87.5	0.99	1	12.5	3.58	0	0	0	0	0	0	1	1	1	1
GO:0006081	9	9	100	1.13	0	0	0	0	0	0	0	0	0	1	1	1	1
GO:0006083	1	0	0	0	0	0	0	0	0	0	1	100	28.22	1	1	1	1
GO:0006085	7	7	100	1.13	0	0	0	0	0	0	0	0	0	1	1	1	1
GO:0006086	4	4	100	1.13	0	0	0	0	0	0	0	0	0	1	1	1	1
GO:0006090	3	3	100	1.13	0	0	0	0	0	0	0	0	0	1	1	1	1
GO:0006091	33	33	100	1.13	0	0	0	0	0	0	0	0	0	1	1	1	1
GO:0006092	12	12	100	1.13	0	0	0	0	0	0	0	0	0	1	1	1	1
GO:0006094	24	24	100	1.13	0	0	0	0	0	0	0	0	0	1	1	1	1
GO:0006096	87	80	91.95	1.04	2	2.3	0.66	3	3.45	0.74	2	2.3	0.65	1	1	1	1
GO:0006097	11	11	100	1.13	0	0	0	0	0	0	0	0	0	1	1	1	1
GO:0006098	14	12	85.71	0.97	0	0	0	2	14.29	3.08	0	0	0	1	1	1	1

GO:0006099	61	61	100	1.13	0	0	0	0	0	0	0	0	0	0.990699217	1	1	1
GO:0006101	3	3	100	1.13	0	0	0	0	0	0	0	0	0	1	1	1	1
GO:0006104	5	5	100	1.13	0	0	0	0	0	0	0	0	0	1	1	1	1
GO:0006106	2	2	100	1.13	0	0	0	0	0	0	0	0	0	1	1	1	1
GO:0006108	5	5	100	1.13	0	0	0	0	0	0	0	0	0	1	1	1	1
GO:0006110	2	2	100	1.13	0	0	0	0	0	0	0	0	0	1	1	1	1
GO:0006112	3	3	100	1.13	0	0	0	0	0	0	0	0	0	1	1	1	1
GO:0006116	1	1	100	1.13	0	0	0	0	0	0	0	0	0	1	1	1	1
GO:0006118	663	602	90.8	1.03	10	1.51	0.43	26	3.92	0.84	25	3.77	1.06	1	1	1	1
GO:0006119	8	8	100	1.13	0	0	0	0	0	0	0	0	0	1	1	1	1
GO:0006120	27	24	88.89	1.01	1	3.7	1.06	1	3.7	0.8	1	3.7	1.05	1	1	1	1
GO:0006122	5	4	80	0.91	1	20	5.73	0	0	0	0	0	0	1	1	1	1
GO:0006125	3	3	100	1.13	0	0	0	0	0	0	0	0	0	1	1	1	1
GO:0006127	2	1	50	0.57	1	50	14.32	0	0	0	0	0	0	1	1	1	1
GO:0006128	1	1	100	1.13	0	0	0	0	0	0	0	0	0	1	1	1	1
GO:0006129	5	3	60	0.68	0	0	0	2	40	8.61	0	0	0	1	1	1	1
GO:0006134	2	2	100	1.13	0	0	0	0	0	0	0	0	0	1	1	1	1
GO:0006139	58	57	98.28	1.11	0	0	0	1	1.72	0.37	0	0	0	1	1	1	1
GO:0006143	2	1	50	0.57	0	0	0	0	0	0	1	50	14.11	1	1	1	1
GO:0006144	8	7	87.5	0.99	0	0	0	0	0	0	1	12.5	3.53	1	1	1	1
GO:0006155	1	1	100	1.13	0	0	0	0	0	0	0	0	0	1	1	1	1
GO:0006163	2	2	100	1.13	0	0	0	0	0	0	0	0	0	1	1	1	1
GO:0006164	44	44	100	1.13	0	0	0	0	0	0	0	0	0	1	1	1	1
GO:0006166	6	6	100	1.13	0	0	0	0	0	0	0	0	0	1	1	1	1
GO:0006168	1	1	100	1.13	0	0	0	0	0	0	0	0	0	1	1	1	1
GO:0006171	5	5	100	1.13	0	0	0	0	0	0	0	0	0	1	1	1	1
GO:0006172	1	1	100	1.13	0	0	0	0	0	0	0	0	0	1	1	1	1
GO:0006173	1	1	100	1.13	0	0	0	0	0	0	0	0	0	1	1	1	1
GO:0006177	13	13	100	1.13	0	0	0	0	0	0	0	0	0	1	1	1	1
GO:0006182	2	2	100	1.13	0	0	0	0	0	0	0	0	0	1	1	1	1
GO:0006183	18	16	88.89	1.01	1	5.56	1.59	1	5.56	1.2	0	0	0	1	1	1	1
GO:0006189	13	13	100	1.13	0	0	0	0	0	0	0	0	0	1	1	1	1
GO:0006203	1	1	100	1.13	0	0	0	0	0	0	0	0	0	1	1	1	1
GO:0006205	2	2	100	1.13	0	0	0	0	0	0	0	0	0	1	1	1	1
GO:0006207	11	11	100	1.13	0	0	0	0	0	0	0	0	0	1	1	1	1
GO:0006208	1	1	100	1.13	0	0	0	0	0	0	0	0	0	1	1	1	1
GO:0006213	2	2	100	1.13	0	0	0	0	0	0	0	0	0	1	1	1	1
GO:0006220	1	1	100	1.13	0	0	0	0	0	0	0	0	0	1	1	1	1
GO:0006221	8	8	100	1.13	0	0	0	0	0	0	0	0	0	1	1	1	1
GO:0006224	3	3	100	1.13	0	0	0	0	0	0	0	0	0	1	1	1	1
GO:0006228	16	14	87.5	0.99	1	6.25	1.79	1	6.25	1.35	0	0	0	1	1	1	1

GO:0006231	2	2	100	1.13	0	0	0	0	0	0	0	0	0	1	1	1	1
GO:0006233	10	10	100	1.13	0	0	0	0	0	0	0	0	0	1	1	1	1
GO:0006235	10	10	100	1.13	0	0	0	0	0	0	0	0	0	1	1	1	1
GO:0006241	16	14	87.5	0.99	1	6.25	1.79	1	6.25	1.35	0	0	0	1	1	1	1
GO:0006259	37	34	91.89	1.04	1	2.7	0.77	2	5.41	1.16	0	0	0	1	1	1	1
GO:0006260	142	137	96.48	1.09	4	2.82	0.81	1	0.7	0.15	0	0	0	0.673267658	1	1	1
GO:0006261	2	2	100	1.13	0	0	0	0	0	0	0	0	0	1	1	1	1
GO:0006265	16	16	100	1.13	0	0	0	0	0	0	0	0	0	1	1	1	1
GO:0006268	10	10	100	1.13	0	0	0	0	0	0	0	0	0	1	1	1	1
GO:0006269	7	7	100	1.13	0	0	0	0	0	0	0	0	0	1	1	1	1
GO:0006270	29	29	100	1.13	0	0	0	0	0	0	0	0	0	1	1	1	1
GO:0006275	3	3	100	1.13	0	0	0	0	0	0	0	0	0	1	1	1	1
GO:0006278	1	1	100	1.13	0	0	0	0	0	0	0	0	0	1	1	1	1
GO:0006280	1	1	100	1.13	0	0	0	0	0	0	0	0	0	1	1	1	1
GO:0006281	159	146	91.82	1.04	4	2.52	0.72	6	3.77	0.81	3	1.89	0.53	1	1	1	1
GO:0006282	1	1	100	1.13	0	0	0	0	0	0	0	0	0	1	1	1	1
GO:0006283	4	4	100	1.13	0	0	0	0	0	0	0	0	0	1	1	1	1
GO:0006284	14	13	92.86	1.05	1	7.14	2.05	0	0	0	0	0	0	1	1	1	1
GO:0006287	2	2	100	1.13	0	0	0	0	0	0	0	0	0	1	1	1	1
GO:0006289	30	28	93.33	1.06	1	3.33	0.95	1	3.33	0.72	0	0	0	1	1	1	1
GO:0006298	10	10	100	1.13	0	0	0	0	0	0	0	0	0	1	1	1	1
GO:0006301	4	4	100	1.13	0	0	0	0	0	0	0	0	0	1	1	1	1
GO:0006302	10	9	90	1.02	0	0	0	1	10	2.15	0	0	0	1	1	1	1
GO:0006303	4	4	100	1.13	0	0	0	0	0	0	0	0	0	1	1	1	1
GO:0006304	6	6	100	1.13	0	0	0	0	0	0	0	0	0	1	1	1	1
GO:0006305	1	1	100	1.13	0	0	0	0	0	0	0	0	0	1	1	1	1
GO:0006306	24	23	95.83	1.09	0	0	0	1	4.17	0.9	0	0	0	1	1	1	1
GO:0006308	2	1	50	0.57	0	0	0	1	50	10.76	0	0	0	1	1	1	1
GO:0006309	2	1	50	0.57	0	0	0	1	50	10.76	0	0	0	1	1	1	1
GO:0006310	32	29	90.63	1.03	2	6.25	1.79	1	3.13	0.67	0	0	0	1	1	1	1
GO:0006312	2	2	100	1.13	0	0	0	0	0	0	0	0	0	1	1	1	1
GO:0006313	2	2	100	1.13	0	0	0	0	0	0	0	0	0	1	1	1	1
GO:0006323	38	38	100	1.13	0	0	0	0	0	0	0	0	0	1	1	1	1
GO:0006325	4	3	75	0.85	0	0	0	1	25	5.38	0	0	0	1	1	1	1
GO:0006333	31	27	87.1	0.99	3	9.68	2.77	1	3.23	0.69	0	0	0	1	1	1	1
GO:0006334	77	66	85.71	0.97	1	1.3	0.37	7	9.09	1.96	3	3.9	1.1	1	1	1	1
GO:0006335	1	1	100	1.13	0	0	0	0	0	0	0	0	0	1	1	1	1
GO:0006337	2	2	100	1.13	0	0	0	0	0	0	0	0	0	1	1	1	1
GO:0006338	30	29	96.67	1.09	0	0	0	1	3.33	0.72	0	0	0	1	1	1	1
GO:0006342	5	4	80	0.91	0	0	0	1	20	4.31	0	0	0	1	1	1	1
GO:0006349	3	3	100	1.13	0	0	0	0	0	0	0	0	0	1	1	1	1

GO:0006350	116	108	93.1	1.05	5	4.31	1.23	2	1.72	0.37	1	0.86	0.24	1	1	1	1
GO:0006351	8	7	87.5	0.99	0	0	0	0	0	0	1	12.5	3.53	1	1	1	1
GO:0006352	7	6	85.71	0.97	0	0	0	1	14.29	3.08	0	0	0	1	1	1	1
GO:0006353	1	1	100	1.13	0	0	0	0	0	0	0	0	0	1	1	1	1
GO:0006354	10	10	100	1.13	0	0	0	0	0	0	0	0	0	1	1	1	1
GO:0006355	1487	1315	88.43	1	60	4.03	1.16	74	4.98	1.07	38	2.56	0.72	1	1	1	1
GO:0006356	4	2	50	0.57	0	0	0	2	50	10.76	0	0	0	1	1	1	1
GO:0006357	114	101	88.6	1	7	6.14	1.76	2	1.75	0.38	4	3.51	0.99	1	1	1	1
GO:0006359	11	9	81.82	0.93	1	9.09	2.6	1	9.09	1.96	0	0	0	1	1	1	1
GO:0006360	2	2	100	1.13	0	0	0	0	0	0	0	0	0	1	1	1	1
GO:0006361	1	1	100	1.13	0	0	0	0	0	0	0	0	0	1	1	1	1
GO:0006364	55	52	94.55	1.07	1	1.82	0.52	2	3.64	0.78	0	0	0	1	1	1	1
GO:0006365	8	8	100	1.13	0	0	0	0	0	0	0	0	0	1	1	1	1
GO:0006366	80	71	88.75	1	1	1.25	0.36	3	3.75	0.81	5	6.25	1.76	1	1	1	1
GO:0006367	20	19	95	1.08	1	5	1.43	0	0	0	0	0	0	1	1	1	1
GO:0006368	5	5	100	1.13	0	0	0	0	0	0	0	0	0	1	1	1	1
GO:0006370	10	7	70	0.79	3	30	8.59	0	0	0	0	0	0	1	1	1	1
GO:0006371	114	110	96.49	1.09	0	0	0	3	2.63	0.57	1	0.88	0.25	1	1	1	1
GO:0006376	24	21	87.5	0.99	0	0	0	2	8.33	1.79	1	4.17	1.18	1	1	1	1
GO:0006378	13	12	92.31	1.05	1	7.69	2.2	0	0	0	0	0	0	1	1	1	1
GO:0006379	10	9	90	1.02	1	10	2.86	0	0	0	0	0	0	1	1	1	1
GO:0006381	7	6	85.71	0.97	1	14.29	4.09	0	0	0	0	0	0	1	1	1	1
GO:0006383	12	12	100	1.13	0	0	0	0	0	0	0	0	0	1	1	1	1
GO:0006388	2	2	100	1.13	0	0	0	0	0	0	0	0	0	1	1	1	1
GO:0006396	123	112	91.06	1.03	9	7.32	2.1	1	0.81	0.18	1	0.81	0.23	1	1	1	1
GO:0006397	125	116	92.8	1.05	2	1.6	0.46	5	4	0.86	2	1.6	0.45	1	1	1	1
GO:0006399	12	11	91.67	1.04	1	8.33	2.39	0	0	0	0	0	0	1	1	1	1
GO:0006400	1	1	100	1.13	0	0	0	0	0	0	0	0	0	1	1	1	1
GO:0006401	5	5	100	1.13	0	0	0	0	0	0	0	0	0	1	1	1	1
GO:0006402	5	5	100	1.13	0	0	0	0	0	0	0	0	0	1	1	1	1
GO:0006405	6	6	100	1.13	0	0	0	0	0	0	0	0	0	1	1	1	1
GO:0006406	13	13	100	1.13	0	0	0	0	0	0	0	0	0	1	1	1	1
GO:0006408	5	5	100	1.13	0	0	0	0	0	0	0	0	0	1	1	1	1
GO:0006411	3	3	100	1.13	0	0	0	0	0	0	0	0	0	1	1	1	1
GO:0006412	491	434	88.39	1	17	3.46	0.99	33	6.72	1.45	7	1.43	0.4	1	1	1	1
GO:0006413	73	72	98.63	1.12	0	0	0	1	1.37	0.29	0	0	0	1	1	1	1
GO:0006414	49	49	100	1.13	0	0	0	0	0	0	0	0	0	1	1	1	1
GO:0006415	10	8	80	0.91	0	0	0	1	10	2.15	1	10	2.82	1	1	1	1
GO:0006417	36	36	100	1.13	0	0	0	0	0	0	0	0	0	1	1	1	1
GO:0006418	139	126	90.65	1.03	6	4.32	1.24	5	3.6	0.77	2	1.44	0.41	1	1	1	1
GO:0006419	5	5	100	1.13	0	0	0	0	0	0	0	0	0	1	1	1	1

GO:0006420	5	5	100	1.13	0	0	0	0	0	0	0	0	0	1	1	1	1
GO:0006421	3	3	100	1.13	0	0	0	0	0	0	0	0	0	1	1	1	1
GO:0006422	9	6	66.67	0.75	3	33.33	9.54	0	0	0	0	0	0	1	1	1	1
GO:0006423	3	3	100	1.13	0	0	0	0	0	0	0	0	0	1	1	1	1
GO:0006424	9	9	100	1.13	0	0	0	0	0	0	0	0	0	1	1	1	1
GO:0006425	1	1	100	1.13	0	0	0	0	0	0	0	0	0	1	1	1	1
GO:0006426	2	2	100	1.13	0	0	0	0	0	0	0	0	0	1	1	1	1
GO:0006427	3	3	100	1.13	0	0	0	0	0	0	0	0	0	1	1	1	1
GO:0006428	7	7	100	1.13	0	0	0	0	0	0	0	0	0	1	1	1	1
GO:0006429	1	1	100	1.13	0	0	0	0	0	0	0	0	0	1	1	1	1
GO:0006430	4	1	25	0.28	3	75	21.47	0	0	0	0	0	0	1	0.683729195	1	1
GO:0006431	2	2	100	1.13	0	0	0	0	0	0	0	0	0	1	1	1	1
GO:0006432	8	8	100	1.13	0	0	0	0	0	0	0	0	0	1	1	1	1
GO:0006433	2	2	100	1.13	0	0	0	0	0	0	0	0	0	1	1	1	1
GO:0006434	6	5	83.33	0.94	0	0	0	1	16.67	3.59	0	0	0	1	1	1	1
GO:0006435	13	12	92.31	1.05	0	0	0	1	7.69	1.66	0	0	0	1	1	1	1
GO:0006436	4	4	100	1.13	0	0	0	0	0	0	0	0	0	1	1	1	1
GO:0006437	5	5	100	1.13	0	0	0	0	0	0	0	0	0	1	1	1	1
GO:0006438	2	2	100	1.13	0	0	0	0	0	0	0	0	0	1	1	1	1
GO:0006441	1	0	0	0	1	100	28.63	0	0	0	0	0	0	1	1	1	1
GO:0006445	37	33	89.19	1.01	4	10.81	3.1	0	0	0	0	0	0	1	1	1	1
GO:0006446	44	41	93.18	1.06	0	0	0	3	6.82	1.47	0	0	0	1	1	1	1
GO:0006449	3	1	33.33	0.38	0	0	0	1	33.33	7.18	1	33.33	9.41	1	1	1	1
GO:0006450	1	1	100	1.13	0	0	0	0	0	0	0	0	0	1	1	1	1
GO:0006457	206	194	94.17	1.07	1	0.49	0.14	9	4.37	0.94	2	0.97	0.27	1	1	1	1
GO:0006461	37	35	94.59	1.07	0	0	0	1	2.7	0.58	1	2.7	0.76	1	1	1	1
GO:0006464	230	210	91.3	1.03	3	1.3	0.37	16	6.96	1.5	1	0.43	0.12	1	1	1	1
GO:0006468	508	468	92.13	1.04	8	1.57	0.45	24	4.72	1.02	8	1.57	0.44	1	1	1	1
GO:0006469	8	6	75	0.85	1	12.5	3.58	0	0	0	1	12.5	3.53	1	1	1	1
GO:0006470	147	139	94.56	1.07	2	1.36	0.39	2	1.36	0.29	4	2.72	0.77	1	1	1	1
GO:0006471	20	18	90	1.02	1	5	1.43	1	5	1.08	0	0	0	1	1	1	1
GO:0006473	2	1	50	0.57	0	0	0	1	50	10.76	0	0	0	1	1	1	1
GO:0006475	7	7	100	1.13	0	0	0	0	0	0	0	0	0	1	1	1	1
GO:0006476	7	7	100	1.13	0	0	0	0	0	0	0	0	0	1	1	1	1
GO:0006477	6	6	100	1.13	0	0	0	0	0	0	0	0	0	1	1	1	1
GO:0006478	1	1	100	1.13	0	0	0	0	0	0	0	0	0	1	1	1	1
GO:0006479	1	1	100	1.13	0	0	0	0	0	0	0	0	0	1	1	1	1
GO:0006481	3	3	100	1.13	0	0	0	0	0	0	0	0	0	1	1	1	1
GO:0006486	62	60	96.77	1.1	2	3.23	0.92	0	0	0	0	0	0	1	1	1	1
GO:0006487	22	21	95.45	1.08	0	0	0	1	4.55	0.98	0	0	0	1	1	1	1
GO:0006488	2	2	100	1.13	0	0	0	0	0	0	0	0	0	1	1	1	1

GO:0006491	4	4	100	1.13	0	0	0	0	0	0	0	0	0	1	1	1	1
GO:0006493	9	8	88.89	1.01	1	11.11	3.18	0	0	0	0	0	0	1	1	1	1
GO:0006497	2	1	50	0.57	0	0	0	1	50	10.76	0	0	0	1	1	1	1
GO:0006499	24	23	95.83	1.09	1	4.17	1.19	0	0	0	0	0	0	1	1	1	1
GO:0006502	14	12	85.71	0.97	0	0	0	2	14.29	3.08	0	0	0	1	1	1	1
GO:0006506	4	4	100	1.13	0	0	0	0	0	0	0	0	0	1	1	1	1
GO:0006507	1	1	100	1.13	0	0	0	0	0	0	0	0	0	1	1	1	1
GO:0006508	562	485	86.3	0.98	23	4.09	1.17	33	5.87	1.26	21	3.74	1.05	1	1	1	1
GO:0006510	5	4	80	0.91	0	0	0	1	20	4.31	0	0	0	1	1	1	1
GO:0006511	201	183	91.04	1.03	6	2.99	0.85	11	5.47	1.18	1	0.5	0.14	1	1	1	1
GO:0006512	130	118	90.77	1.03	1	0.77	0.22	10	7.69	1.66	1	0.77	0.22	1	1	1	1
GO:0006513	3	3	100	1.13	0	0	0	0	0	0	0	0	0	1	1	1	1
GO:0006514	13	13	100	1.13	0	0	0	0	0	0	0	0	0	1	1	1	1
GO:0006515	4	4	100	1.13	0	0	0	0	0	0	0	0	0	1	1	1	1
GO:0006516	9	9	100	1.13	0	0	0	0	0	0	0	0	0	1	1	1	1
GO:0006518	2	2	100	1.13	0	0	0	0	0	0	0	0	0	1	1	1	1
GO:0006520	68	64	94.12	1.07	1	1.47	0.42	2	2.94	0.63	1	1.47	0.42	1	1	1	1
GO:0006525	2	0	0	0	0	0	0	1	50	10.76	1	50	14.11	1	1	1	1
GO:0006526	2	1	50	0.57	0	0	0	1	50	10.76	0	0	0	1	1	1	1
GO:0006527	4	2	50	0.57	0	0	0	1	25	5.38	1	25	7.06	1	1	1	1
GO:0006529	4	4	100	1.13	0	0	0	0	0	0	0	0	0	1	1	1	1
GO:0006533	3	0	0	0	0	0	0	0	0	0	3	100	28.22	1	1	1	0.21002992
GO:0006534	2	2	100	1.13	0	0	0	0	0	0	0	0	0	1	1	1	1
GO:0006535	1	1	100	1.13	0	0	0	0	0	0	0	0	0	1	1	1	1
GO:0006536	1	1	100	1.13	0	0	0	0	0	0	0	0	0	1	1	1	1
GO:0006537	3	3	100	1.13	0	0	0	0	0	0	0	0	0	1	1	1	1
GO:0006541	18	18	100	1.13	0	0	0	0	0	0	0	0	0	1	1	1	1
GO:0006544	1	1	100	1.13	0	0	0	0	0	0	0	0	0	1	1	1	1
GO:0006545	1	1	100	1.13	0	0	0	0	0	0	0	0	0	1	1	1	1
GO:0006546	1	1	100	1.13	0	0	0	0	0	0	0	0	0	1	1	1	1
GO:0006547	8	7	87.5	0.99	0	0	0	1	12.5	2.69	0	0	0	1	1	1	1
GO:0006548	5	5	100	1.13	0	0	0	0	0	0	0	0	0	1	1	1	1
GO:0006552	1	1	100	1.13	0	0	0	0	0	0	0	0	0	1	1	1	1
GO:0006555	10	9	90	1.02	0	0	0	1	10	2.15	0	0	0	1	1	1	1
GO:0006556	2	2	100	1.13	0	0	0	0	0	0	0	0	0	1	1	1	1
GO:0006558	2	2	100	1.13	0	0	0	0	0	0	0	0	0	1	1	1	1
GO:0006559	7	7	100	1.13	0	0	0	0	0	0	0	0	0	1	1	1	1
GO:0006560	3	3	100	1.13	0	0	0	0	0	0	0	0	0	1	1	1	1
GO:0006561	7	7	100	1.13	0	0	0	0	0	0	0	0	0	1	1	1	1
GO:0006562	4	4	100	1.13	0	0	0	0	0	0	0	0	0	1	1	1	1
GO:0006563	4	4	100	1.13	0	0	0	0	0	0	0	0	0	1	1	1	1

GO:0006564	18	17	94.44	1.07	0	0	0	1	5.56	1.2	0	0	0	1	1	1	1
GO:0006565	1	1	100	1.13	0	0	0	0	0	0	0	0	0	1	1	1	1
GO:0006568	1	0	0	0	0	0	0	0	0	0	1	100	28.22	1	1	1	1
GO:0006569	1	0	0	0	0	0	0	0	0	0	1	100	28.22	1	1	1	1
GO:0006572	3	3	100	1.13	0	0	0	0	0	0	0	0	0	1	1	1	1
GO:0006573	1	1	100	1.13	0	0	0	0	0	0	0	0	0	1	1	1	1
GO:0006583	7	3	42.86	0.49	2	28.57	8.18	1	14.29	3.08	1	14.29	4.03	1	1	1	1
GO:0006584	5	3	60	0.68	2	40	11.45	0	0	0	0	0	0	1	1	1	1
GO:0006587	1	1	100	1.13	0	0	0	0	0	0	0	0	0	1	1	1	1
GO:0006590	3	3	100	1.13	0	0	0	0	0	0	0	0	0	1	1	1	1
GO:0006595	8	8	100	1.13	0	0	0	0	0	0	0	0	0	1	1	1	1
GO:0006596	5	5	100	1.13	0	0	0	0	0	0	0	0	0	1	1	1	1
GO:0006598	1	1	100	1.13	0	0	0	0	0	0	0	0	0	1	1	1	1
GO:0006601	5	5	100	1.13	0	0	0	0	0	0	0	0	0	1	1	1	1
GO:0006605	205	180	87.8	0.99	7	3.41	0.98	13	6.34	1.37	5	2.44	0.69	1	1	1	1
GO:0006606	31	29	93.55	1.06	0	0	0	2	6.45	1.39	0	0	0	1	1	1	1
GO:0006607	2	2	100	1.13	0	0	0	0	0	0	0	0	0	1	1	1	1
GO:0006608	2	2	100	1.13	0	0	0	0	0	0	0	0	0	1	1	1	1
GO:0006610	9	8	88.89	1.01	0	0	0	0	0	0	1	11.11	3.14	1	1	1	1
GO:0006611	8	8	100	1.13	0	0	0	0	0	0	0	0	0	1	1	1	1
GO:0006613	18	17	94.44	1.07	1	5.56	1.59	0	0	0	0	0	0	1	1	1	1
GO:0006614	2	2	100	1.13	0	0	0	0	0	0	0	0	0	1	1	1	1
GO:0006620	33	23	69.7	0.79	5	15.15	4.34	3	9.09	1.96	2	6.06	1.71	1	1	1	1
GO:0006622	1	1	100	1.13	0	0	0	0	0	0	0	0	0	1	1	1	1
GO:0006624	6	6	100	1.13	0	0	0	0	0	0	0	0	0	1	1	1	1
GO:0006625	2	2	100	1.13	0	0	0	0	0	0	0	0	0	1	1	1	1
GO:0006626	2	1	50	0.57	0	0	0	1	50	10.76	0	0	0	1	1	1	1
GO:0006627	3	3	100	1.13	0	0	0	0	0	0	0	0	0	1	1	1	1
GO:0006628	14	12	85.71	0.97	0	0	0	2	14.29	3.08	0	0	0	1	1	1	1
GO:0006629	115	98	85.22	0.96	4	3.48	1	10	8.7	1.87	3	2.61	0.74	1	1	1	1
GO:0006630	11	7	63.64	0.72	0	0	0	2	18.18	3.91	2	18.18	5.13	1	1	1	1
GO:0006631	87	80	91.95	1.04	5	5.75	1.65	1	1.15	0.25	1	1.15	0.32	1	1	1	1
GO:0006633	38	34	89.47	1.01	1	2.63	0.75	2	5.26	1.13	1	2.63	0.74	1	1	1	1
GO:0006635	15	15	100	1.13	0	0	0	0	0	0	0	0	0	1	1	1	1
GO:0006636	4	4	100	1.13	0	0	0	0	0	0	0	0	0	1	1	1	1
GO:0006637	3	3	100	1.13	0	0	0	0	0	0	0	0	0	1	1	1	1
GO:0006641	3	1	33.33	0.38	0	0	0	1	33.33	7.18	1	33.33	9.41	1	1	1	1
GO:0006643	18	17	94.44	1.07	0	0	0	1	5.56	1.2	0	0	0	1	1	1	1
GO:0006644	30	25	83.33	0.94	3	10	2.86	1	3.33	0.72	1	3.33	0.94	1	1	1	1
GO:0006646	2	2	100	1.13	0	0	0	0	0	0	0	0	0	1	1	1	1
GO:0006650	3	3	100	1.13	0	0	0	0	0	0	0	0	0	1	1	1	1

GO:0006653	1	1	100	1.13	0	0	0	0	0	0	0	0	0	1	1	1	1
GO:0006654	5	5	100	1.13	0	0	0	0	0	0	0	0	0	1	1	1	1
GO:0006656	1	0	0	0	1	100	28.63	0	0	0	0	0	0	1	1	1	1
GO:0006658	1	1	100	1.13	0	0	0	0	0	0	0	0	0	1	1	1	1
GO:0006659	9	9	100	1.13	0	0	0	0	0	0	0	0	0	1	1	1	1
GO:0006661	6	6	100	1.13	0	0	0	0	0	0	0	0	0	1	1	1	1
GO:0006665	25	21	84	0.95	1	4	1.15	1	4	0.86	2	8	2.26	1	1	1	1
GO:0006668	2	2	100	1.13	0	0	0	0	0	0	0	0	0	1	1	1	1
GO:0006670	2	2	100	1.13	0	0	0	0	0	0	0	0	0	1	1	1	1
GO:0006672	4	4	100	1.13	0	0	0	0	0	0	0	0	0	1	1	1	1
GO:0006684	3	3	100	1.13	0	0	0	0	0	0	0	0	0	1	1	1	1
GO:0006688	3	3	100	1.13	0	0	0	0	0	0	0	0	0	1	1	1	1
GO:0006691	15	13	86.67	0.98	1	6.67	1.91	1	6.67	1.44	0	0	0	1	1	1	1
GO:0006693	9	7	77.78	0.88	1	11.11	3.18	0	0	0	1	11.11	3.14	1	1	1	1
GO:0006694	34	20	58.82	0.67	0	0	0	8	23.53	5.07	6	17.65	4.98	1	1	0.731242595	1
GO:0006695	21	18	85.71	0.97	0	0	0	2	9.52	2.05	1	4.76	1.34	1	1	1	1
GO:0006700	6	3	50	0.57	0	0	0	2	33.33	7.18	1	16.67	4.7	1	1	1	1
GO:0006725	34	33	97.06	1.1	0	0	0	1	2.94	0.63	0	0	0	1	1	1	1
GO:0006729	4	4	100	1.13	0	0	0	0	0	0	0	0	0	1	1	1	1
GO:0006730	32	26	81.25	0.92	1	3.13	0.89	5	15.63	3.36	0	0	0	1	1	1	1
GO:0006731	1	1	100	1.13	0	0	0	0	0	0	0	0	0	1	1	1	1
GO:0006736	1	1	100	1.13	0	0	0	0	0	0	0	0	0	1	1	1	1
GO:0006739	2	1	50	0.57	0	0	0	1	50	10.76	0	0	0	1	1	1	1
GO:0006742	6	5	83.33	0.94	0	0	0	1	16.67	3.59	0	0	0	1	1	1	1
GO:0006744	11	10	90.91	1.03	0	0	0	1	9.09	1.96	0	0	0	1	1	1	1
GO:0006749	10	10	100	1.13	0	0	0	0	0	0	0	0	0	1	1	1	1
GO:0006750	6	6	100	1.13	0	0	0	0	0	0	0	0	0	1	1	1	1
GO:0006754	89	86	96.63	1.09	1	1.12	0.32	2	2.25	0.48	0	0	0	1	1	1	1
GO:0006758	10	10	100	1.13	0	0	0	0	0	0	0	0	0	1	1	1	1
GO:0006766	1	1	100	1.13	0	0	0	0	0	0	0	0	0	1	1	1	1
GO:0006768	1	1	100	1.13	0	0	0	0	0	0	0	0	0	1	1	1	1
GO:0006772	5	5	100	1.13	0	0	0	0	0	0	0	0	0	1	1	1	1
GO:0006776	2	2	100	1.13	0	0	0	0	0	0	0	0	0	1	1	1	1
GO:0006777	6	4	66.67	0.75	0	0	0	0	0	0	2	33.33	9.41	1	1	1	1
GO:0006779	19	18	94.74	1.07	1	5.26	1.51	0	0	0	0	0	0	1	1	1	1
GO:0006783	25	23	92	1.04	2	8	2.29	0	0	0	0	0	0	1	1	1	1
GO:0006788	7	7	100	1.13	0	0	0	0	0	0	0	0	0	1	1	1	1
GO:0006790	3	3	100	1.13	0	0	0	0	0	0	0	0	0	1	1	1	1
GO:0006796	12	10	83.33	0.94	1	8.33	2.39	0	0	0	1	8.33	2.35	1	1	1	1
GO:0006800	4	4	100	1.13	0	0	0	0	0	0	0	0	0	1	1	1	1
GO:0006801	12	12	100	1.13	0	0	0	0	0	0	0	0	0	1	1	1	1

GO:0006802	2	2	100	1.13	0	0	0	0	0	0	0	0	0	1	1	1	1
GO:0006803	11	9	81.82	0.93	0	0	0	1	9.09	1.96	1	9.09	2.57	1	1	1	1
GO:0006804	23	20	86.96	0.98	2	8.7	2.49	1	4.35	0.94	0	0	0	1	1	1	1
GO:0006805	25	14	56	0.63	1	4	1.15	3	12	2.58	7	28	7.9	1	1	1	0.124582103
GO:0006807	13	10	76.92	0.87	0	0	0	2	15.38	3.31	1	7.69	2.17	1	1	1	1
GO:0006809	5	5	100	1.13	0	0	0	0	0	0	0	0	0	1	1	1	1
GO:0006810	794	722	90.93	1.03	20	2.52	0.72	29	3.65	0.79	23	2.9	0.82	1	1	1	1
GO:0006811	128	116	90.63	1.03	7	5.47	1.57	2	1.56	0.34	3	2.34	0.66	1	1	1	1
GO:0006812	78	75	96.15	1.09	0	0	0	0	0	0	3	3.85	1.09	1	1	1	1
GO:0006813	105	100	95.24	1.08	2	1.9	0.55	2	1.9	0.41	1	0.95	0.27	1	1	1	1
GO:0006814	53	44	83.02	0.94	2	3.77	1.08	2	3.77	0.81	5	9.43	2.66	1	1	1	1
GO:0006816	35	31	88.57	1	3	8.57	2.45	0	0	0	1	2.86	0.81	1	1	1	1
GO:0006817	4	3	75	0.85	0	0	0	1	25	5.38	0	0	0	1	1	1	1
GO:0006818	35	33	94.29	1.07	0	0	0	2	5.71	1.23	0	0	0	1	1	1	1
GO:0006820	33	32	96.97	1.1	0	0	0	1	3.03	0.65	0	0	0	1	1	1	1
GO:0006821	49	41	83.67	0.95	4	8.16	2.34	2	4.08	0.88	2	4.08	1.15	1	1	1	1
GO:0006823	9	8	88.89	1.01	1	11.11	3.18	0	0	0	0	0	0	1	1	1	1
GO:0006824	5	5	100	1.13	0	0	0	0	0	0	0	0	0	1	1	1	1
GO:0006825	9	9	100	1.13	0	0	0	0	0	0	0	0	0	1	1	1	1
GO:0006826	22	19	86.36	0.98	0	0	0	2	9.09	1.96	1	4.55	1.28	1	1	1	1
GO:0006829	10	10	100	1.13	0	0	0	0	0	0	0	0	0	1	1	1	1
GO:0006832	68	55	80.88	0.92	6	8.82	2.53	5	7.35	1.58	2	2.94	0.83	1	1	1	1
GO:0006833	6	4	66.67	0.75	1	16.67	4.77	0	0	0	1	16.67	4.7	1	1	1	1
GO:0006835	13	12	92.31	1.05	0	0	0	1	7.69	1.66	0	0	0	1	1	1	1
GO:0006836	25	23	92	1.04	2	8	2.29	0	0	0	0	0	0	1	1	1	1
GO:0006839	20	19	95	1.08	0	0	0	1	5	1.08	0	0	0	1	1	1	1
GO:0006843	1	1	100	1.13	0	0	0	0	0	0	0	0	0	1	1	1	1
GO:0006844	1	1	100	1.13	0	0	0	0	0	0	0	0	0	1	1	1	1
GO:0006854	7	7	100	1.13	0	0	0	0	0	0	0	0	0	1	1	1	1
GO:0006855	1	1	100	1.13	0	0	0	0	0	0	0	0	0	1	1	1	1
GO:0006857	12	10	83.33	0.94	1	8.33	2.39	1	8.33	1.79	0	0	0	1	1	1	1
GO:0006865	44	41	93.18	1.06	0	0	0	2	4.55	0.98	1	2.27	0.64	1	1	1	1
GO:0006868	1	1	100	1.13	0	0	0	0	0	0	0	0	0	1	1	1	1
GO:0006869	40	34	85	0.96	0	0	0	6	15	3.23	0	0	0	1	1	1	1
GO:0006874	13	12	92.31	1.05	0	0	0	1	7.69	1.66	0	0	0	1	1	1	1
GO:0006878	8	8	100	1.13	0	0	0	0	0	0	0	0	0	1	1	1	1
GO:0006879	21	18	85.71	0.97	0	0	0	2	9.52	2.05	1	4.76	1.34	1	1	1	1
GO:0006882	2	2	100	1.13	0	0	0	0	0	0	0	0	0	1	1	1	1
GO:0006884	8	8	100	1.13	0	0	0	0	0	0	0	0	0	1	1	1	1
GO:0006885	11	9	81.82	0.93	0	0	0	0	0	0	2	18.18	5.13	1	1	1	1
GO:0006886	583	565	96.91	1.1	6	1.03	0.29	10	1.72	0.37	2	0.34	0.1	1.77E-12	1	1	1

GO:0006887	13	13	100	1.13	0	0	0	0	0	0	0	0	0	1	1	1	1
GO:0006888	35	34	97.14	1.1	0	0	0	1	2.86	0.62	0	0	0	1	1	1	1
GO:0006890	6	6	100	1.13	0	0	0	0	0	0	0	0	0	1	1	1	1
GO:0006891	22	22	100	1.13	0	0	0	0	0	0	0	0	0	1	1	1	1
GO:0006892	7	7	100	1.13	0	0	0	0	0	0	0	0	0	1	1	1	1
GO:0006893	4	4	100	1.13	0	0	0	0	0	0	0	0	0	1	1	1	1
GO:0006895	4	4	100	1.13	0	0	0	0	0	0	0	0	0	1	1	1	1
GO:0006896	3	3	100	1.13	0	0	0	0	0	0	0	0	0	1	1	1	1
GO:0006897	89	83	93.26	1.06	2	2.25	0.64	0	0	0	4	4.49	1.27	1	1	1	1
GO:0006898	15	8	53.33	0.6	0	0	0	3	20	4.31	4	26.67	7.53	1	1	1	1
GO:0006899	41	41	100	1.13	0	0	0	0	0	0	0	0	0	1	1	1	1
GO:0006901	4	4	100	1.13	0	0	0	0	0	0	0	0	0	1	1	1	1
GO:0006903	4	4	100	1.13	0	0	0	0	0	0	0	0	0	1	1	1	1
GO:0006904	15	15	100	1.13	0	0	0	0	0	0	0	0	0	1	1	1	1
GO:0006906	6	6	100	1.13	0	0	0	0	0	0	0	0	0	1	1	1	1
GO:0006909	13	10	76.92	0.87	0	0	0	3	23.08	4.97	0	0	0	1	1	1	1
GO:0006910	9	6	66.67	0.75	0	0	0	0	0	0	3	33.33	9.41	1	1	1	1
GO:0006911	11	5	45.45	0.51	0	0	0	3	27.27	5.87	3	27.27	7.7	1	1	1	1
GO:0006912	1	1	100	1.13	0	0	0	0	0	0	0	0	0	1	1	1	1
GO:0006913	19	18	94.74	1.07	1	5.26	1.51	0	0	0	0	0	0	1	1	1	1
GO:0006914	8	8	100	1.13	0	0	0	0	0	0	0	0	0	1	1	1	1
GO:0006915	295	263	89.15	1.01	6	2.03	0.58	13	4.41	0.95	13	4.41	1.24	1	1	1	1
GO:0006916	76	72	94.74	1.07	1	1.32	0.38	2	2.63	0.57	1	1.32	0.37	1	1	1	1
GO:0006917	67	62	92.54	1.05	0	0	0	1	1.49	0.32	4	5.97	1.68	1	1	1	1
GO:0006919	12	12	100	1.13	0	0	0	0	0	0	0	0	0	1	1	1	1
GO:0006927	4	4	100	1.13	0	0	0	0	0	0	0	0	0	1	1	1	1
GO:0006928	68	67	98.53	1.12	0	0	0	1	1.47	0.32	0	0	0	1	1	1	1
GO:0006929	1	1	100	1.13	0	0	0	0	0	0	0	0	0	1	1	1	1
GO:0006930	4	4	100	1.13	0	0	0	0	0	0	0	0	0	1	1	1	1
GO:0006931	4	4	100	1.13	0	0	0	0	0	0	0	0	0	1	1	1	1
GO:0006935	72	39	54.17	0.61	4	5.56	1.59	16	22.22	4.78	13	18.06	5.1	1	1	0.001318854	0.013581426
GO:0006936	26	18	69.23	0.78	5	19.23	5.51	2	7.69	1.66	1	3.85	1.09	1	1	1	1
GO:0006937	10	9	90	1.02	0	0	0	0	0	0	1	10	2.82	1	1	1	1
GO:0006938	1	1	100	1.13	0	0	0	0	0	0	0	0	0	1	1	1	1
GO:0006939	10	10	100	1.13	0	0	0	0	0	0	0	0	0	1	1	1	1
GO:0006940	4	3	75	0.85	1	25	7.16	0	0	0	0	0	0	1	1	1	1
GO:0006941	8	5	62.5	0.71	0	0	0	3	37.5	8.07	0	0	0	1	1	1	1
GO:0006944	15	15	100	1.13	0	0	0	0	0	0	0	0	0	1	1	1	1
GO:0006950	38	34	89.47	1.01	0	0	0	0	0	0	4	10.53	2.97	1	1	1	1
GO:0006951	38	34	89.47	1.01	2	5.26	1.51	1	2.63	0.57	1	2.63	0.74	1	1	1	1
GO:0006952	131	75	57.25	0.65	10	7.63	2.19	29	22.14	4.77	17	12.98	3.66	1	1	3.12E-08	0.051134543

GO:0006953	13	8	61.54	0.7	1	7.69	2.2	3	23.08	4.97	1	7.69	2.17	1	1	1	1
GO:0006954	105	77	73.33	0.83	4	3.81	1.09	13	12.38	2.67	11	10.48	2.96	1	1	1	1
GO:0006955	253	152	60.08	0.68	14	5.53	1.58	57	22.53	4.85	30	11.86	3.35	1	1	1.33E-18	0.000260026
GO:0006956	17	11	64.71	0.73	3	17.65	5.05	0	0	0	3	17.65	4.98	1	1	1	1
GO:0006957	15	9	60	0.68	0	0	0	0	0	0	6	40	11.29	1	1	1	0.04573977
GO:0006958	21	14	66.67	0.75	0	0	0	0	0	0	7	33.33	9.41	1	1	1	0.034565048
GO:0006959	14	8	57.14	0.65	1	7.14	2.05	2	14.29	3.08	3	21.43	6.05	1	1	1	1
GO:0006960	14	10	71.43	0.81	0	0	0	3	21.43	4.61	1	7.14	2.02	1	1	1	1
GO:0006961	2	1	50	0.57	0	0	0	1	50	10.76	0	0	0	1	1	1	1
GO:0006968	18	14	77.78	0.88	0	0	0	2	11.11	2.39	2	11.11	3.14	1	1	1	1
GO:0006970	2	2	100	1.13	0	0	0	0	0	0	0	0	0	1	1	1	1
GO:0006972	6	5	83.33	0.94	0	0	0	1	16.67	3.59	0	0	0	1	1	1	1
GO:0006974	20	20	100	1.13	0	0	0	0	0	0	0	0	0	1	1	1	1
GO:0006976	2	2	100	1.13	0	0	0	0	0	0	0	0	0	1	1	1	1
GO:0006979	63	59	93.65	1.06	1	1.59	0.45	3	4.76	1.03	0	0	0	1	1	1	1
GO:0006983	6	6	100	1.13	0	0	0	0	0	0	0	0	0	1	1	1	1
GO:0006986	7	7	100	1.13	0	0	0	0	0	0	0	0	0	1	1	1	1
GO:0006990	2	2	100	1.13	0	0	0	0	0	0	0	0	0	1	1	1	1
GO:0006991	3	3	100	1.13	0	0	0	0	0	0	0	0	0	1	1	1	1
GO:0006996	6	6	100	1.13	0	0	0	0	0	0	0	0	0	1	1	1	1
GO:0006998	1	1	100	1.13	0	0	0	0	0	0	0	0	0	1	1	1	1
GO:0007000	2	2	100	1.13	0	0	0	0	0	0	0	0	0	1	1	1	1
GO:0007001	85	73	85.88	0.97	1	1.18	0.34	8	9.41	2.03	3	3.53	1	1	1	1	1
GO:0007004	8	8	100	1.13	0	0	0	0	0	0	0	0	0	1	1	1	1
GO:0007005	2	2	100	1.13	0	0	0	0	0	0	0	0	0	1	1	1	1
GO:0007006	1	1	100	1.13	0	0	0	0	0	0	0	0	0	1	1	1	1
GO:0007010	97	89	91.75	1.04	1	1.03	0.3	6	6.19	1.33	1	1.03	0.29	1	1	1	1
GO:0007011	2	2	100	1.13	0	0	0	0	0	0	0	0	0	1	1	1	1
GO:0007012	17	17	100	1.13	0	0	0	0	0	0	0	0	0	1	1	1	1
GO:0007015	12	11	91.67	1.04	0	0	0	0	0	0	1	8.33	2.35	1	1	1	1
GO:0007016	1	1	100	1.13	0	0	0	0	0	0	0	0	0	1	1	1	1
GO:0007017	104	97	93.27	1.06	4	3.85	1.1	3	2.88	0.62	0	0	0	1	1	1	1
GO:0007018	37	32	86.49	0.98	1	2.7	0.77	1	2.7	0.58	3	8.11	2.29	1	1	1	1
GO:0007019	1	1	100	1.13	0	0	0	0	0	0	0	0	0	1	1	1	1
GO:0007020	6	6	100	1.13	0	0	0	0	0	0	0	0	0	1	1	1	1
GO:0007021	1	1	100	1.13	0	0	0	0	0	0	0	0	0	1	1	1	1
GO:0007022	3	3	100	1.13	0	0	0	0	0	0	0	0	0	1	1	1	1
GO:0007023	1	1	100	1.13	0	0	0	0	0	0	0	0	0	1	1	1	1
GO:0007024	1	1	100	1.13	0	0	0	0	0	0	0	0	0	1	1	1	1
GO:0007025	8	8	100	1.13	0	0	0	0	0	0	0	0	0	1	1	1	1
GO:0007026	6	5	83.33	0.94	0	0	0	0	0	0	1	16.67	4.7	1	1	1	1

GO:0007028	2	2	100	1.13	0	0	0	0	0	0	0	0	0	1	1	1	1
GO:0007030	6	4	66.67	0.75	1	16.67	4.77	1	16.67	3.59	0	0	0	1	1	1	1
GO:0007031	20	20	100	1.13	0	0	0	0	0	0	0	0	0	1	1	1	1
GO:0007032	3	3	100	1.13	0	0	0	0	0	0	0	0	0	1	1	1	1
GO:0007035	4	2	50	0.57	0	0	0	2	50	10.76	0	0	0	1	1	1	1
GO:0007039	1	0	0	0	0	0	0	1	100	21.53	0	0	0	1	1	1	1
GO:0007040	3	0	0	0	1	33.33	9.54	0	0	0	2	66.67	18.82	1	1	1	1
GO:0007042	4	2	50	0.57	0	0	0	2	50	10.76	0	0	0	1	1	1	1
GO:0007044	7	7	100	1.13	0	0	0	0	0	0	0	0	0	1	1	1	1
GO:0007046	72	62	86.11	0.98	3	4.17	1.19	7	9.72	2.09	0	0	0	1	1	1	1
GO:0007048	70	63	90	1.02	6	8.57	2.45	1	1.43	0.31	0	0	0	1	1	1	1
GO:0007049	312	298	95.51	1.08	5	1.6	0.46	7	2.24	0.48	2	0.64	0.18	0.003663386	1	1	1
GO:0007050	39	39	100	1.13	0	0	0	0	0	0	0	0	0	1	1	1	1
GO:0007051	4	4	100	1.13	0	0	0	0	0	0	0	0	0	1	1	1	1
GO:0007052	2	2	100	1.13	0	0	0	0	0	0	0	0	0	1	1	1	1
GO:0007059	24	23	95.83	1.09	0	0	0	1	4.17	0.9	0	0	0	1	1	1	1
GO:0007060	1	1	100	1.13	0	0	0	0	0	0	0	0	0	1	1	1	1
GO:0007067	124	119	95.97	1.09	0	0	0	4	3.23	0.69	1	0.81	0.23	1	1	1	1
GO:0007076	10	9	90	1.02	0	0	0	1	10	2.15	0	0	0	1	1	1	1
GO:0007088	6	6	100	1.13	0	0	0	0	0	0	0	0	0	1	1	1	1
GO:0007089	5	5	100	1.13	0	0	0	0	0	0	0	0	0	1	1	1	1
GO:0007090	1	1	100	1.13	0	0	0	0	0	0	0	0	0	1	1	1	1
GO:0007091	1	1	100	1.13	0	0	0	0	0	0	0	0	0	1	1	1	1
GO:0007094	6	6	100	1.13	0	0	0	0	0	0	0	0	0	1	1	1	1
GO:0007095	1	1	100	1.13	0	0	0	0	0	0	0	0	0	1	1	1	1
GO:0007096	2	1	50	0.57	1	50	14.32	0	0	0	0	0	0	1	1	1	1
GO:0007098	1	1	100	1.13	0	0	0	0	0	0	0	0	0	1	1	1	1
GO:0007100	1	1	100	1.13	0	0	0	0	0	0	0	0	0	1	1	1	1
GO:0007113	4	4	100	1.13	0	0	0	0	0	0	0	0	0	1	1	1	1
GO:0007125	5	5	100	1.13	0	0	0	0	0	0	0	0	0	1	1	1	1
GO:0007126	31	29	93.55	1.06	0	0	0	2	6.45	1.39	0	0	0	1	1	1	1
GO:0007127	1	1	100	1.13	0	0	0	0	0	0	0	0	0	1	1	1	1
GO:0007129	1	1	100	1.13	0	0	0	0	0	0	0	0	0	1	1	1	1
GO:0007130	3	3	100	1.13	0	0	0	0	0	0	0	0	0	1	1	1	1
GO:0007131	2	2	100	1.13	0	0	0	0	0	0	0	0	0	1	1	1	1
GO:0007143	11	11	100	1.13	0	0	0	0	0	0	0	0	0	1	1	1	1
GO:0007145	4	4	100	1.13	0	0	0	0	0	0	0	0	0	1	1	1	1
GO:0007148	41	36	87.8	0.99	2	4.88	1.4	0	0	0	3	7.32	2.07	1	1	1	1
GO:0007154	17	13	76.47	0.87	2	11.76	3.37	0	0	0	2	11.76	3.32	1	1	1	1
GO:0007155	412	326	79.13	0.9	22	5.34	1.53	36	8.74	1.88	28	6.8	1.92	1	1	1	1
GO:0007156	45	27	60	0.68	4	8.89	2.55	2	4.44	0.96	12	26.67	7.53	1	1	1	0.000367938

GO:0007157	55	42	76.36	0.86	1	1.82	0.52	6	10.91	2.35	6	10.91	3.08	1	1	1	1
GO:0007160	72	58	80.56	0.91	3	4.17	1.19	5	6.94	1.49	6	8.33	2.35	1	1	1	1
GO:0007162	4	4	100	1.13	0	0	0	0	0	0	0	0	0	1	1	1	1
GO:0007163	4	4	100	1.13	0	0	0	0	0	0	0	0	0	1	1	1	1
GO:0007164	2	2	100	1.13	0	0	0	0	0	0	0	0	0	1	1	1	1
GO:0007165	544	482	88.6	1	16	2.94	0.84	27	4.96	1.07	19	3.49	0.99	1	1	1	1
GO:0007166	90	55	61.11	0.69	8	8.89	2.55	14	15.56	3.35	13	14.44	4.08	1	1	0.483553535	0.164358293
GO:0007167	3	0	0	0	0	0	0	1	33.33	7.18	2	66.67	18.82	1	1	1	1
GO:0007168	3	2	66.67	0.75	0	0	0	1	33.33	7.18	0	0	0	1	1	1	1
GO:0007169	72	51	70.83	0.8	1	1.39	0.4	15	20.83	4.48	5	6.94	1.96	1	1	0.007252094	1
GO:0007170	3	3	100	1.13	0	0	0	0	0	0	0	0	0	1	1	1	1
GO:0007171	1	0	0	0	1	100	28.63	0	0	0	0	0	0	1	1	1	1
GO:0007173	6	4	66.67	0.75	1	16.67	4.77	0	0	0	1	16.67	4.7	1	1	1	1
GO:0007176	7	7	100	1.13	0	0	0	0	0	0	0	0	0	1	1	1	1
GO:0007177	2	2	100	1.13	0	0	0	0	0	0	0	0	0	1	1	1	1
GO:0007178	16	16	100	1.13	0	0	0	0	0	0	0	0	0	1	1	1	1
GO:0007179	23	21	91.3	1.03	0	0	0	1	4.35	0.94	1	4.35	1.23	1	1	1	1
GO:0007181	1	1	100	1.13	0	0	0	0	0	0	0	0	0	1	1	1	1
GO:0007182	7	7	100	1.13	0	0	0	0	0	0	0	0	0	1	1	1	1
GO:0007183	5	5	100	1.13	0	0	0	0	0	0	0	0	0	1	1	1	1
GO:0007184	2	2	100	1.13	0	0	0	0	0	0	0	0	0	1	1	1	1
GO:0007185	22	19	86.36	0.98	1	4.55	1.3	0	0	0	2	9.09	2.57	1	1	1	1
GO:0007186	264	205	77.65	0.88	16	6.06	1.74	21	7.95	1.71	22	8.33	2.35	1	1	1	1
GO:0007187	2	2	100	1.13	0	0	0	0	0	0	0	0	0	1	1	1	1
GO:0007188	4	3	75	0.85	0	0	0	0	0	0	1	25	7.06	1	1	1	1
GO:0007189	8	8	100	1.13	0	0	0	0	0	0	0	0	0	1	1	1	1
GO:0007190	12	12	100	1.13	0	0	0	0	0	0	0	0	0	1	1	1	1
GO:0007193	10	10	100	1.13	0	0	0	0	0	0	0	0	0	1	1	1	1
GO:0007194	3	3	100	1.13	0	0	0	0	0	0	0	0	0	1	1	1	1
GO:0007199	3	3	100	1.13	0	0	0	0	0	0	0	0	0	1	1	1	1
GO:0007200	7	6	85.71	0.97	1	14.29	4.09	0	0	0	0	0	0	1	1	1	1
GO:0007202	2	2	100	1.13	0	0	0	0	0	0	0	0	0	1	1	1	1
GO:0007203	3	3	100	1.13	0	0	0	0	0	0	0	0	0	1	1	1	1
GO:0007204	10	10	100	1.13	0	0	0	0	0	0	0	0	0	1	1	1	1
GO:0007205	13	12	92.31	1.05	1	7.69	2.2	0	0	0	0	0	0	1	1	1	1
GO:0007210	1	1	100	1.13	0	0	0	0	0	0	0	0	0	1	1	1	1
GO:0007212	2	2	100	1.13	0	0	0	0	0	0	0	0	0	1	1	1	1
GO:0007213	3	3	100	1.13	0	0	0	0	0	0	0	0	0	1	1	1	1
GO:0007214	8	5	62.5	0.71	1	12.5	3.58	0	0	0	2	25	7.06	1	1	1	1
GO:0007215	1	1	100	1.13	0	0	0	0	0	0	0	0	0	1	1	1	1
GO:0007216	1	1	100	1.13	0	0	0	0	0	0	0	0	0	1	1	1	1

GO:0007217	1	1	100	1.13	0	0	0	0	0	0	0	0	0	1	1	1	1
GO:0007218	59	54	91.53	1.04	0	0	0	3	5.08	1.09	2	3.39	0.96	1	1	1	1
GO:0007219	8	7	87.5	0.99	0	0	0	0	0	0	1	12.5	3.53	1	1	1	1
GO:0007222	6	6	100	1.13	0	0	0	0	0	0	0	0	0	1	1	1	1
GO:0007223	8	8	100	1.13	0	0	0	0	0	0	0	0	0	1	1	1	1
GO:0007229	74	56	75.68	0.86	5	6.76	1.93	8	10.81	2.33	5	6.76	1.91	1	1	1	1
GO:0007231	2	2	100	1.13	0	0	0	0	0	0	0	0	0	1	1	1	1
GO:0007242	537	486	90.5	1.02	11	2.05	0.59	29	5.4	1.16	11	2.05	0.58	1	1	1	1
GO:0007243	11	9	81.82	0.93	0	0	0	1	9.09	1.96	1	9.09	2.57	1	1	1	1
GO:0007249	41	38	92.68	1.05	0	0	0	3	7.32	1.58	0	0	0	1	1	1	1
GO:0007250	1	0	0	0	0	0	0	1	100	21.53	0	0	0	1	1	1	1
GO:0007252	9	9	100	1.13	0	0	0	0	0	0	0	0	0	1	1	1	1
GO:0007253	1	1	100	1.13	0	0	0	0	0	0	0	0	0	1	1	1	1
GO:0007254	14	14	100	1.13	0	0	0	0	0	0	0	0	0	1	1	1	1
GO:0007256	1	1	100	1.13	0	0	0	0	0	0	0	0	0	1	1	1	1
GO:0007257	3	3	100	1.13	0	0	0	0	0	0	0	0	0	1	1	1	1
GO:0007259	16	14	87.5	0.99	0	0	0	2	12.5	2.69	0	0	0	1	1	1	1
GO:0007260	3	3	100	1.13	0	0	0	0	0	0	0	0	0	1	1	1	1
GO:0007261	3	3	100	1.13	0	0	0	0	0	0	0	0	0	1	1	1	1
GO:0007262	3	3	100	1.13	0	0	0	0	0	0	0	0	0	1	1	1	1
GO:0007263	1	1	100	1.13	0	0	0	0	0	0	0	0	0	1	1	1	1
GO:0007264	314	302	96.18	1.09	4	1.27	0.36	5	1.59	0.34	3	0.96	0.27	0.000275514	1	1	1
GO:0007265	33	29	87.88	1	1	3.03	0.87	3	9.09	1.96	0	0	0	1	1	1	1
GO:0007266	45	45	100	1.13	0	0	0	0	0	0	0	0	0	1	1	1	1
GO:0007267	52	38	73.08	0.83	5	9.62	2.75	5	9.62	2.07	4	7.69	2.17	1	1	1	1
GO:0007268	68	59	86.76	0.98	5	7.35	2.11	1	1.47	0.32	3	4.41	1.25	1	1	1	1
GO:0007269	21	19	90.48	1.02	0	0	0	2	9.52	2.05	0	0	0	1	1	1	1
GO:0007270	13	13	100	1.13	0	0	0	0	0	0	0	0	0	1	1	1	1
GO:0007271	2	2	100	1.13	0	0	0	0	0	0	0	0	0	1	1	1	1
GO:0007272	4	3	75	0.85	1	25	7.16	0	0	0	0	0	0	1	1	1	1
GO:0007273	1	1	100	1.13	0	0	0	0	0	0	0	0	0	1	1	1	1
GO:0007275	289	249	86.16	0.98	18	6.23	1.78	11	3.81	0.82	11	3.81	1.07	1	1	1	1
GO:0007276	5	5	100	1.13	0	0	0	0	0	0	0	0	0	1	1	1	1
GO:0007281	9	9	100	1.13	0	0	0	0	0	0	0	0	0	1	1	1	1
GO:0007283	74	64	86.49	0.98	4	5.41	1.55	5	6.76	1.45	1	1.35	0.38	1	1	1	1
GO:0007286	5	5	100	1.13	0	0	0	0	0	0	0	0	0	1	1	1	1
GO:0007288	3	3	100	1.13	0	0	0	0	0	0	0	0	0	1	1	1	1
GO:0007292	8	8	100	1.13	0	0	0	0	0	0	0	0	0	1	1	1	1
GO:0007301	1	1	100	1.13	0	0	0	0	0	0	0	0	0	1	1	1	1
GO:0007304	1	0	0	0	1	100	28.63	0	0	0	0	0	0	1	1	1	1
GO:0007328	1	1	100	1.13	0	0	0	0	0	0	0	0	0	1	1	1	1

GO:0007335	3	3	100	1.13	0	0	0	0	0	0	0	0	0	1	1	1	1
GO:0007338	13	9	69.23	0.78	0	0	0	2	15.38	3.31	2	15.38	4.34	1	1	1	1
GO:0007339	1	1	100	1.13	0	0	0	0	0	0	0	0	0	1	1	1	1
GO:0007340	3	3	100	1.13	0	0	0	0	0	0	0	0	0	1	1	1	1
GO:0007341	1	0	0	0	0	0	0	0	0	0	1	100	28.22	1	1	1	1
GO:0007345	53	49	92.45	1.05	3	5.66	1.62	1	1.89	0.41	0	0	0	1	1	1	1
GO:0007346	3	3	100	1.13	0	0	0	0	0	0	0	0	0	1	1	1	1
GO:0007368	2	2	100	1.13	0	0	0	0	0	0	0	0	0	1	1	1	1
GO:0007369	1	1	100	1.13	0	0	0	0	0	0	0	0	0	1	1	1	1
GO:0007379	6	6	100	1.13	0	0	0	0	0	0	0	0	0	1	1	1	1
GO:0007386	1	1	100	1.13	0	0	0	0	0	0	0	0	0	1	1	1	1
GO:0007389	21	13	61.9	0.7	4	19.05	5.45	3	14.29	3.08	1	4.76	1.34	1	1	1	1
GO:0007397	54	41	75.93	0.86	7	12.96	3.71	5	9.26	1.99	1	1.85	0.52	1	1	1	1
GO:0007398	1	1	100	1.13	0	0	0	0	0	0	0	0	0	1	1	1	1
GO:0007399	138	115	83.33	0.94	12	8.7	2.49	5	3.62	0.78	6	4.35	1.23	1	1	1	1
GO:0007400	1	1	100	1.13	0	0	0	0	0	0	0	0	0	1	1	1	1
GO:0007401	1	0	0	0	1	100	28.63	0	0	0	0	0	0	1	1	1	1
GO:0007405	13	13	100	1.13	0	0	0	0	0	0	0	0	0	1	1	1	1
GO:0007409	13	12	92.31	1.05	0	0	0	1	7.69	1.66	0	0	0	1	1	1	1
GO:0007411	26	23	88.46	1	1	3.85	1.1	1	3.85	0.83	1	3.85	1.09	1	1	1	1
GO:0007417	33	30	90.91	1.03	2	6.06	1.74	1	3.03	0.65	0	0	0	1	1	1	1
GO:0007418	1	0	0	0	1	100	28.63	0	0	0	0	0	0	1	1	1	1
GO:0007420	18	18	100	1.13	0	0	0	0	0	0	0	0	0	1	1	1	1
GO:0007422	4	4	100	1.13	0	0	0	0	0	0	0	0	0	1	1	1	1
GO:0007423	12	11	91.67	1.04	0	0	0	0	0	0	1	8.33	2.35	1	1	1	1
GO:0007435	1	1	100	1.13	0	0	0	0	0	0	0	0	0	1	1	1	1
GO:0007456	6	6	100	1.13	0	0	0	0	0	0	0	0	0	1	1	1	1
GO:0007485	1	0	0	0	1	100	28.63	0	0	0	0	0	0	1	1	1	1
GO:0007492	10	6	60	0.68	1	10	2.86	3	30	6.46	0	0	0	1	1	1	1
GO:0007498	6	5	83.33	0.94	0	0	0	1	16.67	3.59	0	0	0	1	1	1	1
GO:0007500	2	1	50	0.57	1	50	14.32	0	0	0	0	0	0	1	1	1	1
GO:0007507	28	26	92.86	1.05	0	0	0	1	3.57	0.77	1	3.57	1.01	1	1	1	1
GO:0007515	27	23	85.19	0.96	1	3.7	1.06	0	0	0	3	11.11	3.14	1	1	1	1
GO:0007516	1	1	100	1.13	0	0	0	0	0	0	0	0	0	1	1	1	1
GO:0007517	72	63	87.5	0.99	4	5.56	1.59	4	5.56	1.2	1	1.39	0.39	1	1	1	1
GO:0007519	17	15	88.24	1	1	5.88	1.68	0	0	0	1	5.88	1.66	1	1	1	1
GO:0007528	1	1	100	1.13	0	0	0	0	0	0	0	0	0	1	1	1	1
GO:0007529	1	1	100	1.13	0	0	0	0	0	0	0	0	0	1	1	1	1
GO:0007530	4	4	100	1.13	0	0	0	0	0	0	0	0	0	1	1	1	1
GO:0007548	6	6	100	1.13	0	0	0	0	0	0	0	0	0	1	1	1	1
GO:0007549	2	2	100	1.13	0	0	0	0	0	0	0	0	0	1	1	1	1

GO:0007565	9	9	100	1.13	0	0	0	0	0	0	0	0	0	1	1	1	1
GO:0007566	2	2	100	1.13	0	0	0	0	0	0	0	0	0	1	1	1	1
GO:0007568	3	3	100	1.13	0	0	0	0	0	0	0	0	0	1	1	1	1
GO:0007569	4	4	100	1.13	0	0	0	0	0	0	0	0	0	1	1	1	1
GO:0007582	11	10	90.91	1.03	0	0	0	0	0	0	1	9.09	2.57	1	1	1	1
GO:0007583	5	4	80	0.91	1	20	5.73	0	0	0	0	0	0	1	1	1	1
GO:0007584	3	3	100	1.13	0	0	0	0	0	0	0	0	0	1	1	1	1
GO:0007585	11	11	100	1.13	0	0	0	0	0	0	0	0	0	1	1	1	1
GO:0007586	12	9	75	0.85	1	8.33	2.39	0	0	0	2	16.67	4.7	1	1	1	1
GO:0007588	9	5	55.56	0.63	0	0	0	4	44.44	9.57	0	0	0	1	1	1	1
GO:0007595	9	4	44.44	0.5	0	0	0	3	33.33	7.18	2	22.22	6.27	1	1	1	1
GO:0007596	56	43	76.79	0.87	1	1.79	0.51	2	3.57	0.77	10	17.86	5.04	1	1	1	0.194824138
GO:0007599	2	1	50	0.57	1	50	14.32	0	0	0	0	0	0	1	1	1	1
GO:0007600	19	19	100	1.13	0	0	0	0	0	0	0	0	0	1	1	1	1
GO:0007601	34	33	97.06	1.1	1	2.94	0.84	0	0	0	0	0	0	1	1	1	1
GO:0007602	11	11	100	1.13	0	0	0	0	0	0	0	0	0	1	1	1	1
GO:0007603	2	2	100	1.13	0	0	0	0	0	0	0	0	0	1	1	1	1
GO:0007605	34	31	91.18	1.03	0	0	0	2	5.88	1.27	1	2.94	0.83	1	1	1	1
GO:0007607	1	1	100	1.13	0	0	0	0	0	0	0	0	0	1	1	1	1
GO:0007608	6	6	100	1.13	0	0	0	0	0	0	0	0	0	1	1	1	1
GO:0007610	10	7	70	0.79	1	10	2.86	0	0	0	2	20	5.64	1	1	1	1
GO:0007611	2	2	100	1.13	0	0	0	0	0	0	0	0	0	1	1	1	1
GO:0007612	18	18	100	1.13	0	0	0	0	0	0	0	0	0	1	1	1	1
GO:0007613	9	9	100	1.13	0	0	0	0	0	0	0	0	0	1	1	1	1
GO:0007620	3	1	33.33	0.38	0	0	0	0	0	0	2	66.67	18.82	1	1	1	1
GO:0007622	13	13	100	1.13	0	0	0	0	0	0	0	0	0	1	1	1	1
GO:0007623	14	12	85.71	0.97	2	14.29	4.09	0	0	0	0	0	0	1	1	1	1
GO:0007624	1	1	100	1.13	0	0	0	0	0	0	0	0	0	1	1	1	1
GO:0007625	5	5	100	1.13	0	0	0	0	0	0	0	0	0	1	1	1	1
GO:0007626	5	3	60	0.68	1	20	5.73	1	20	4.31	0	0	0	1	1	1	1
GO:0007631	7	6	85.71	0.97	1	14.29	4.09	0	0	0	0	0	0	1	1	1	1
GO:0007638	1	0	0	0	1	100	28.63	0	0	0	0	0	0	1	1	1	1
GO:0008001	1	0	0	0	0	0	0	0	0	0	1	100	28.22	1	1	1	1
GO:0008009	45	25	55.56	0.63	0	0	0	11	24.44	5.26	9	20	5.64	1	1	0.032849416	0.180543513
GO:0008013	1	1	100	1.13	0	0	0	0	0	0	0	0	0	1	1	1	1
GO:0008014	28	15	53.57	0.61	3	10.71	3.07	1	3.57	0.77	9	32.14	9.07	1	1	1	0.00255928
GO:0008015	6	3	50	0.57	0	0	0	3	50	10.76	0	0	0	1	1	1	1
GO:0008016	13	12	92.31	1.05	0	0	0	1	7.69	1.66	0	0	0	1	1	1	1
GO:0008017	22	21	95.45	1.08	0	0	0	0	0	0	1	4.55	1.28	1	1	1	1
GO:0008020	2	2	100	1.13	0	0	0	0	0	0	0	0	0	1	1	1	1
GO:0008021	44	38	86.36	0.98	2	4.55	1.3	4	9.09	1.96	0	0	0	1	1	1	1

GO:0008022	10	10	100	1.13	0	0	0	0	0	0	0	0	0	1	1	1	1
GO:0008023	6	6	100	1.13	0	0	0	0	0	0	0	0	0	1	1	1	1
GO:0008026	146	136	93.15	1.05	6	4.11	1.18	1	0.68	0.15	3	2.05	0.58	1	1	1	1
GO:0008028	3	2	66.67	0.75	0	0	0	1	33.33	7.18	0	0	0	1	1	1	1
GO:0008033	28	27	96.43	1.09	1	3.57	1.02	0	0	0	0	0	0	1	1	1	1
GO:0008034	1	1	100	1.13	0	0	0	0	0	0	0	0	0	1	1	1	1
GO:0008037	3	3	100	1.13	0	0	0	0	0	0	0	0	0	1	1	1	1
GO:0008042	15	15	100	1.13	0	0	0	0	0	0	0	0	0	1	1	1	1
GO:0008045	5	5	100	1.13	0	0	0	0	0	0	0	0	0	1	1	1	1
GO:0008046	5	5	100	1.13	0	0	0	0	0	0	0	0	0	1	1	1	1
GO:0008047	19	17	89.47	1.01	0	0	0	2	10.53	2.27	0	0	0	1	1	1	1
GO:0008048	1	1	100	1.13	0	0	0	0	0	0	0	0	0	1	1	1	1
GO:0008051	5	5	100	1.13	0	0	0	0	0	0	0	0	0	1	1	1	1
GO:0008053	5	5	100	1.13	0	0	0	0	0	0	0	0	0	1	1	1	1
GO:0008054	2	1	50	0.57	1	50	14.32	0	0	0	0	0	0	1	1	1	1
GO:0008060	1	1	100	1.13	0	0	0	0	0	0	0	0	0	1	1	1	1
GO:0008064	2	2	100	1.13	0	0	0	0	0	0	0	0	0	1	1	1	1
GO:0008067	7	7	100	1.13	0	0	0	0	0	0	0	0	0	1	1	1	1
GO:0008073	4	4	100	1.13	0	0	0	0	0	0	0	0	0	1	1	1	1
GO:0008075	1	1	100	1.13	0	0	0	0	0	0	0	0	0	1	1	1	1
GO:0008076	60	56	93.33	1.06	1	1.67	0.48	2	3.33	0.72	1	1.67	0.47	1	1	1	1
GO:0008077	6	6	100	1.13	0	0	0	0	0	0	0	0	0	1	1	1	1
GO:0008080	42	39	92.86	1.05	2	4.76	1.36	0	0	0	1	2.38	0.67	1	1	1	1
GO:0008081	3	3	100	1.13	0	0	0	0	0	0	0	0	0	1	1	1	1
GO:0008083	60	49	81.67	0.92	1	1.67	0.48	4	6.67	1.44	6	10	2.82	1	1	1	1
GO:0008090	12	12	100	1.13	0	0	0	0	0	0	0	0	0	1	1	1	1
GO:0008091	4	1	25	0.28	2	50	14.32	0	0	0	1	25	7.06	1	1	1	1
GO:0008092	23	23	100	1.13	0	0	0	0	0	0	0	0	0	1	1	1	1
GO:0008093	3	2	66.67	0.75	0	0	0	0	0	0	1	33.33	9.41	1	1	1	1
GO:0008094	40	37	92.5	1.05	2	5	1.43	0	0	0	1	2.5	0.71	1	1	1	1
GO:0008095	1	1	100	1.13	0	0	0	0	0	0	0	0	0	1	1	1	1
GO:0008097	1	1	100	1.13	0	0	0	0	0	0	0	0	0	1	1	1	1
GO:0008099	1	1	100	1.13	0	0	0	0	0	0	0	0	0	1	1	1	1
GO:0008104	15	15	100	1.13	0	0	0	0	0	0	0	0	0	1	1	1	1
GO:0008106	6	6	100	1.13	0	0	0	0	0	0	0	0	0	1	1	1	1
GO:0008107	1	1	100	1.13	0	0	0	0	0	0	0	0	0	1	1	1	1
GO:0008108	1	0	0	0	0	0	0	1	100	21.53	0	0	0	1	1	1	1
GO:0008109	1	1	100	1.13	0	0	0	0	0	0	0	0	0	1	1	1	1
GO:0008110	1	1	100	1.13	0	0	0	0	0	0	0	0	0	1	1	1	1
GO:0008111	3	3	100	1.13	0	0	0	0	0	0	0	0	0	1	1	1	1
GO:0008113	2	1	50	0.57	0	0	0	1	50	10.76	0	0	0	1	1	1	1

GO:0008114	6	6	100	1.13	0	0	0	0	0	0	0	0	0	1	1	1	1
GO:0008115	1	1	100	1.13	0	0	0	0	0	0	0	0	0	1	1	1	1
GO:0008116	1	1	100	1.13	0	0	0	0	0	0	0	0	0	1	1	1	1
GO:0008117	5	5	100	1.13	0	0	0	0	0	0	0	0	0	1	1	1	1
GO:0008118	5	5	100	1.13	0	0	0	0	0	0	0	0	0	1	1	1	1
GO:0008119	1	0	0	0	1	100	28.63	0	0	0	0	0	0	1	1	1	1
GO:0008120	2	2	100	1.13	0	0	0	0	0	0	0	0	0	1	1	1	1
GO:0008121	18	15	83.33	0.94	1	5.56	1.59	2	11.11	2.39	0	0	0	1	1	1	1
GO:0008123	2	0	0	0	0	0	0	0	0	0	2	100	28.22	1	1	1	1
GO:0008124	2	2	100	1.13	0	0	0	0	0	0	0	0	0	1	1	1	1
GO:0008129	3	3	100	1.13	0	0	0	0	0	0	0	0	0	1	1	1	1
GO:0008130	2	1	50	0.57	0	0	0	1	50	10.76	0	0	0	1	1	1	1
GO:0008131	3	3	100	1.13	0	0	0	0	0	0	0	0	0	1	1	1	1
GO:0008132	2	2	100	1.13	0	0	0	0	0	0	0	0	0	1	1	1	1
GO:0008133	4	0	0	0	1	25	7.16	2	50	10.76	1	25	7.06	1	1	1	1
GO:0008134	27	27	100	1.13	0	0	0	0	0	0	0	0	0	1	1	1	1
GO:0008135	10	10	100	1.13	0	0	0	0	0	0	0	0	0	1	1	1	1
GO:0008136	2	2	100	1.13	0	0	0	0	0	0	0	0	0	1	1	1	1
GO:0008137	47	44	93.62	1.06	1	2.13	0.61	1	2.13	0.46	1	2.13	0.6	1	1	1	1
GO:0008138	51	50	98.04	1.11	1	1.96	0.56	0	0	0	0	0	0	1	1	1	1
GO:0008139	5	5	100	1.13	0	0	0	0	0	0	0	0	0	1	1	1	1
GO:0008140	2	2	100	1.13	0	0	0	0	0	0	0	0	0	1	1	1	1
GO:0008142	1	1	100	1.13	0	0	0	0	0	0	0	0	0	1	1	1	1
GO:0008143	2	2	100	1.13	0	0	0	0	0	0	0	0	0	1	1	1	1
GO:0008146	24	18	75	0.85	0	0	0	2	8.33	1.79	4	16.67	4.7	1	1	1	1
GO:0008147	1	1	100	1.13	0	0	0	0	0	0	0	0	0	1	1	1	1
GO:0008151	301	266	88.37	1	9	2.99	0.86	21	6.98	1.5	5	1.66	0.47	1	1	1	1
GO:0008152	577	504	87.35	0.99	14	2.43	0.69	40	6.93	1.49	19	3.29	0.93	1	1	1	1
GO:0008154	3	2	66.67	0.75	0	0	0	1	33.33	7.18	0	0	0	1	1	1	1
GO:0008166	8	8	100	1.13	0	0	0	0	0	0	0	0	0	1	1	1	1
GO:0008168	63	57	90.48	1.02	2	3.17	0.91	3	4.76	1.03	1	1.59	0.45	1	1	1	1
GO:0008169	3	3	100	1.13	0	0	0	0	0	0	0	0	0	1	1	1	1
GO:0008170	17	16	94.12	1.07	0	0	0	1	5.88	1.27	0	0	0	1	1	1	1
GO:0008171	5	5	100	1.13	0	0	0	0	0	0	0	0	0	1	1	1	1
GO:0008172	3	3	100	1.13	0	0	0	0	0	0	0	0	0	1	1	1	1
GO:0008173	6	6	100	1.13	0	0	0	0	0	0	0	0	0	1	1	1	1
GO:0008174	3	3	100	1.13	0	0	0	0	0	0	0	0	0	1	1	1	1
GO:0008175	3	3	100	1.13	0	0	0	0	0	0	0	0	0	1	1	1	1
GO:0008177	4	4	100	1.13	0	0	0	0	0	0	0	0	0	1	1	1	1
GO:0008180	2	2	100	1.13	0	0	0	0	0	0	0	0	0	1	1	1	1
GO:0008181	42	38	90.48	1.02	1	2.38	0.68	1	2.38	0.51	2	4.76	1.34	1	1	1	1

GO:0008184	2	2	100	1.13	0	0	0	0	0	0	0	0	0	1	1	1	1
GO:0008186	4	3	75	0.85	0	0	0	0	0	0	1	25	7.06	1	1	1	1
GO:0008187	3	2	66.67	0.75	0	0	0	0	0	0	1	33.33	9.41	1	1	1	1
GO:0008189	14	13	92.86	1.05	0	0	0	1	7.14	1.54	0	0	0	1	1	1	1
GO:0008191	4	4	100	1.13	0	0	0	0	0	0	0	0	0	1	1	1	1
GO:0008192	1	1	100	1.13	0	0	0	0	0	0	0	0	0	1	1	1	1
GO:0008193	4	4	100	1.13	0	0	0	0	0	0	0	0	0	1	1	1	1
GO:0008195	2	2	100	1.13	0	0	0	0	0	0	0	0	0	1	1	1	1
GO:0008198	3	3	100	1.13	0	0	0	0	0	0	0	0	0	1	1	1	1
GO:0008199	16	13	81.25	0.92	0	0	0	2	12.5	2.69	1	6.25	1.76	1	1	1	1
GO:0008200	2	2	100	1.13	0	0	0	0	0	0	0	0	0	1	1	1	1
GO:0008201	74	59	79.73	0.9	1	1.35	0.39	12	16.22	3.49	2	2.7	0.76	1	1	0.957753773	1
GO:0008202	19	11	57.89	0.66	1	5.26	1.51	3	15.79	3.4	4	21.05	5.94	1	1	1	1
GO:0008203	33	30	90.91	1.03	0	0	0	3	9.09	1.96	0	0	0	1	1	1	1
GO:0008206	3	1	33.33	0.38	0	0	0	0	0	0	2	66.67	18.82	1	1	1	1
GO:0008209	2	0	0	0	0	0	0	0	0	0	2	100	28.22	1	1	1	1
GO:0008212	2	2	100	1.13	0	0	0	0	0	0	0	0	0	1	1	1	1
GO:0008215	4	4	100	1.13	0	0	0	0	0	0	0	0	0	1	1	1	1
GO:0008217	19	16	84.21	0.95	2	10.53	3.01	0	0	0	1	5.26	1.49	1	1	1	1
GO:0008219	5	5	100	1.13	0	0	0	0	0	0	0	0	0	1	1	1	1
GO:0008220	5	5	100	1.13	0	0	0	0	0	0	0	0	0	1	1	1	1
GO:0008222	22	19	86.36	0.98	2	9.09	2.6	0	0	0	1	4.55	1.28	1	1	1	1
GO:0008228	1	1	100	1.13	0	0	0	0	0	0	0	0	0	1	1	1	1
GO:0008233	259	222	85.71	0.97	13	5.02	1.44	17	6.56	1.41	7	2.7	0.76	1	1	1	1
GO:0008234	120	112	93.33	1.06	0	0	0	4	3.33	0.72	4	3.33	0.94	1	1	1	1
GO:0008235	18	17	94.44	1.07	1	5.56	1.59	0	0	0	0	0	0	1	1	1	1
GO:0008236	22	22	100	1.13	0	0	0	0	0	0	0	0	0	1	1	1	1
GO:0008237	135	105	77.78	0.88	11	8.15	2.33	16	11.85	2.55	3	2.22	0.63	1	1	1	1
GO:0008238	3	3	100	1.13	0	0	0	0	0	0	0	0	0	1	1	1	1
GO:0008239	5	5	100	1.13	0	0	0	0	0	0	0	0	0	1	1	1	1
GO:0008243	2	2	100	1.13	0	0	0	0	0	0	0	0	0	1	1	1	1
GO:0008246	50	43	86	0.97	1	2	0.57	2	4	0.86	4	8	2.26	1	1	1	1
GO:0008247	5	3	60	0.68	0	0	0	0	0	0	2	40	11.29	1	1	1	1
GO:0008248	108	103	95.37	1.08	2	1.85	0.53	2	1.85	0.4	1	0.93	0.26	1	1	1	1
GO:0008249	1	1	100	1.13	0	0	0	0	0	0	0	0	0	1	1	1	1
GO:0008250	8	8	100	1.13	0	0	0	0	0	0	0	0	0	1	1	1	1
GO:0008251	2	2	100	1.13	0	0	0	0	0	0	0	0	0	1	1	1	1
GO:0008253	2	2	100	1.13	0	0	0	0	0	0	0	0	0	1	1	1	1
GO:0008260	4	4	100	1.13	0	0	0	0	0	0	0	0	0	1	1	1	1
GO:0008264	1	1	100	1.13	0	0	0	0	0	0	0	0	0	1	1	1	1
GO:0008266	3	2	66.67	0.75	0	0	0	0	0	0	1	33.33	9.41	1	1	1	1

GO:0008270	523	464	88.72	1	23	4.4	1.26	30	5.74	1.23	6	1.15	0.32	1	1	1	1
GO:0008271	5	2	40	0.45	0	0	0	2	40	8.61	1	20	5.64	1	1	1	1
GO:0008272	8	5	62.5	0.71	0	0	0	2	25	5.38	1	12.5	3.53	1	1	1	1
GO:0008276	11	11	100	1.13	0	0	0	0	0	0	0	0	0	1	1	1	1
GO:0008277	14	14	100	1.13	0	0	0	0	0	0	0	0	0	1	1	1	1
GO:0008279	3	3	100	1.13	0	0	0	0	0	0	0	0	0	1	1	1	1
GO:0008283	139	125	89.93	1.02	5	3.6	1.03	5	3.6	0.77	4	2.88	0.81	1	1	1	1
GO:0008284	56	51	91.07	1.03	1	1.79	0.51	3	5.36	1.15	1	1.79	0.5	1	1	1	1
GO:0008285	50	48	96	1.09	1	2	0.57	1	2	0.43	0	0	0	1	1	1	1
GO:0008286	9	9	100	1.13	0	0	0	0	0	0	0	0	0	1	1	1	1
GO:0008287	38	35	92.11	1.04	0	0	0	2	5.26	1.13	1	2.63	0.74	1	1	1	1
GO:0008289	75	64	85.33	0.97	2	2.67	0.76	7	9.33	2.01	2	2.67	0.75	1	1	1	1
GO:0008290	12	11	91.67	1.04	0	0	0	0	0	0	1	8.33	2.35	1	1	1	1
GO:0008295	1	1	100	1.13	0	0	0	0	0	0	0	0	0	1	1	1	1
GO:0008298	5	5	100	1.13	0	0	0	0	0	0	0	0	0	1	1	1	1
GO:0008299	12	11	91.67	1.04	0	0	0	0	0	0	1	8.33	2.35	1	1	1	1
GO:0008300	3	3	100	1.13	0	0	0	0	0	0	0	0	0	1	1	1	1
GO:0008301	1	1	100	1.13	0	0	0	0	0	0	0	0	0	1	1	1	1
GO:0008303	3	2	66.67	0.75	0	0	0	0	0	0	1	33.33	9.41	1	1	1	1
GO:0008304	14	13	92.86	1.05	1	7.14	2.05	0	0	0	0	0	0	1	1	1	1
GO:0008305	47	34	72.34	0.82	3	6.38	1.83	5	10.64	2.29	5	10.64	3	1	1	1	1
GO:0008307	22	17	77.27	0.87	4	18.18	5.21	0	0	0	1	4.55	1.28	1	1	1	1
GO:0008308	19	19	100	1.13	0	0	0	0	0	0	0	0	0	1	1	1	1
GO:0008312	5	5	100	1.13	0	0	0	0	0	0	0	0	0	1	1	1	1
GO:0008318	22	20	90.91	1.03	0	0	0	2	9.09	1.96	0	0	0	1	1	1	1
GO:0008319	8	5	62.5	0.71	3	37.5	10.74	0	0	0	0	0	0	1	1	1	1
GO:0008320	16	16	100	1.13	0	0	0	0	0	0	0	0	0	1	1	1	1
GO:0008321	1	1	100	1.13	0	0	0	0	0	0	0	0	0	1	1	1	1
GO:0008324	17	17	100	1.13	0	0	0	0	0	0	0	0	0	1	1	1	1
GO:0008326	2	2	100	1.13	0	0	0	0	0	0	0	0	0	1	1	1	1
GO:0008327	1	1	100	1.13	0	0	0	0	0	0	0	0	0	1	1	1	1
GO:0008328	1	1	100	1.13	0	0	0	0	0	0	0	0	0	1	1	1	1
GO:0008332	2	2	100	1.13	0	0	0	0	0	0	0	0	0	1	1	1	1
GO:0008333	2	2	100	1.13	0	0	0	0	0	0	0	0	0	1	1	1	1
GO:0008336	1	0	0	0	1	100	28.63	0	0	0	0	0	0	1	1	1	1
GO:0008338	17	17	100	1.13	0	0	0	0	0	0	0	0	0	1	1	1	1
GO:0008339	17	17	100	1.13	0	0	0	0	0	0	0	0	0	1	1	1	1
GO:0008340	3	2	66.67	0.75	0	0	0	1	33.33	7.18	0	0	0	1	1	1	1
GO:0008344	7	7	100	1.13	0	0	0	0	0	0	0	0	0	1	1	1	1
GO:0008347	1	1	100	1.13	0	0	0	0	0	0	0	0	0	1	1	1	1
GO:0008349	17	17	100	1.13	0	0	0	0	0	0	0	0	0	1	1	1	1

GO:0008352	1	1	100	1.13	0	0	0	0	0	0	0	0	0	1	1	1	1
GO:0008353	1	1	100	1.13	0	0	0	0	0	0	0	0	0	1	1	1	1
GO:0008354	2	2	100	1.13	0	0	0	0	0	0	0	0	0	1	1	1	1
GO:0008356	1	1	100	1.13	0	0	0	0	0	0	0	0	0	1	1	1	1
GO:0008360	16	15	93.75	1.06	0	0	0	0	0	0	1	6.25	1.76	1	1	1	1
GO:0008361	2	2	100	1.13	0	0	0	0	0	0	0	0	0	1	1	1	1
GO:0008366	4	1	25	0.28	3	75	21.47	0	0	0	0	0	0	1	0.683729195	1	1
GO:0008367	1	1	100	1.13	0	0	0	0	0	0	0	0	0	1	1	1	1
GO:0008372	236	217	91.95	1.04	2	0.85	0.24	10	4.24	0.91	7	2.97	0.84	1	1	1	1
GO:0008373	25	25	100	1.13	0	0	0	0	0	0	0	0	0	1	1	1	1
GO:0008374	2	2	100	1.13	0	0	0	0	0	0	0	0	0	1	1	1	1
GO:0008375	7	7	100	1.13	0	0	0	0	0	0	0	0	0	1	1	1	1
GO:0008376	6	6	100	1.13	0	0	0	0	0	0	0	0	0	1	1	1	1
GO:0008378	16	16	100	1.13	0	0	0	0	0	0	0	0	0	1	1	1	1
GO:0008379	5	5	100	1.13	0	0	0	0	0	0	0	0	0	1	1	1	1
GO:0008380	5	5	100	1.13	0	0	0	0	0	0	0	0	0	1	1	1	1
GO:0008382	1	1	100	1.13	0	0	0	0	0	0	0	0	0	1	1	1	1
GO:0008383	1	1	100	1.13	0	0	0	0	0	0	0	0	0	1	1	1	1
GO:0008384	19	18	94.74	1.07	0	0	0	1	5.26	1.13	0	0	0	1	1	1	1
GO:0008395	2	2	100	1.13	0	0	0	0	0	0	0	0	0	1	1	1	1
GO:0008398	2	2	100	1.13	0	0	0	0	0	0	0	0	0	1	1	1	1
GO:0008408	19	19	100	1.13	0	0	0	0	0	0	0	0	0	1	1	1	1
GO:0008409	4	4	100	1.13	0	0	0	0	0	0	0	0	0	1	1	1	1
GO:0008410	4	4	100	1.13	0	0	0	0	0	0	0	0	0	1	1	1	1
GO:0008413	3	3	100	1.13	0	0	0	0	0	0	0	0	0	1	1	1	1
GO:0008415	85	80	94.12	1.07	3	3.53	1.01	1	1.18	0.25	1	1.18	0.33	1	1	1	1
GO:0008417	8	8	100	1.13	0	0	0	0	0	0	0	0	0	1	1	1	1
GO:0008418	3	2	66.67	0.75	0	0	0	1	33.33	7.18	0	0	0	1	1	1	1
GO:0008419	2	2	100	1.13	0	0	0	0	0	0	0	0	0	1	1	1	1
GO:0008420	13	12	92.31	1.05	1	7.69	2.2	0	0	0	0	0	0	1	1	1	1
GO:0008423	3	3	100	1.13	0	0	0	0	0	0	0	0	0	1	1	1	1
GO:0008425	3	3	100	1.13	0	0	0	0	0	0	0	0	0	1	1	1	1
GO:0008426	1	1	100	1.13	0	0	0	0	0	0	0	0	0	1	1	1	1
GO:0008429	3	3	100	1.13	0	0	0	0	0	0	0	0	0	1	1	1	1
GO:0008430	9	6	66.67	0.75	3	33.33	9.54	0	0	0	0	0	0	1	1	1	1
GO:0008431	2	2	100	1.13	0	0	0	0	0	0	0	0	0	1	1	1	1
GO:0008433	5	4	80	0.91	1	20	5.73	0	0	0	0	0	0	1	1	1	1
GO:0008435	4	4	100	1.13	0	0	0	0	0	0	0	0	0	1	1	1	1
GO:0008436	20	19	95	1.08	0	0	0	0	0	0	1	5	1.41	1	1	1	1
GO:0008440	1	1	100	1.13	0	0	0	0	0	0	0	0	0	1	1	1	1
GO:0008441	1	1	100	1.13	0	0	0	0	0	0	0	0	0	1	1	1	1

GO:0008442	1	1	100	1.13	0	0	0	0	0	0	0	0	0	1	1	1	1
GO:0008443	19	19	100	1.13	0	0	0	0	0	0	0	0	0	1	1	1	1
GO:0008445	2	0	0	0	0	0	0	0	0	0	2	100	28.22	1	1	1	1
GO:0008448	4	4	100	1.13	0	0	0	0	0	0	0	0	0	1	1	1	1
GO:0008449	5	5	100	1.13	0	0	0	0	0	0	0	0	0	1	1	1	1
GO:0008450	6	6	100	1.13	0	0	0	0	0	0	0	0	0	1	1	1	1
GO:0008451	1	1	100	1.13	0	0	0	0	0	0	0	0	0	1	1	1	1
GO:0008453	1	0	0	0	1	100	28.63	0	0	0	0	0	0	1	1	1	1
GO:0008455	3	3	100	1.13	0	0	0	0	0	0	0	0	0	1	1	1	1
GO:0008456	1	1	100	1.13	0	0	0	0	0	0	0	0	0	1	1	1	1
GO:0008458	1	1	100	1.13	0	0	0	0	0	0	0	0	0	1	1	1	1
GO:0008460	3	3	100	1.13	0	0	0	0	0	0	0	0	0	1	1	1	1
GO:0008462	10	10	100	1.13	0	0	0	0	0	0	0	0	0	1	1	1	1
GO:0008464	2	2	100	1.13	0	0	0	0	0	0	0	0	0	1	1	1	1
GO:0008467	3	3	100	1.13	0	0	0	0	0	0	0	0	0	1	1	1	1
GO:0008474	5	5	100	1.13	0	0	0	0	0	0	0	0	0	1	1	1	1
GO:0008475	5	5	100	1.13	0	0	0	0	0	0	0	0	0	1	1	1	1
GO:0008476	1	1	100	1.13	0	0	0	0	0	0	0	0	0	1	1	1	1
GO:0008478	2	2	100	1.13	0	0	0	0	0	0	0	0	0	1	1	1	1
GO:0008479	4	4	100	1.13	0	0	0	0	0	0	0	0	0	1	1	1	1
GO:0008481	2	2	100	1.13	0	0	0	0	0	0	0	0	0	1	1	1	1
GO:0008482	1	1	100	1.13	0	0	0	0	0	0	0	0	0	1	1	1	1
GO:0008483	37	35	94.59	1.07	2	5.41	1.55	0	0	0	0	0	0	1	1	1	1
GO:0008484	22	18	81.82	0.93	3	13.64	3.9	1	4.55	0.98	0	0	0	1	1	1	1
GO:0008486	1	1	100	1.13	0	0	0	0	0	0	0	0	0	1	1	1	1
GO:0008488	1	1	100	1.13	0	0	0	0	0	0	0	0	0	1	1	1	1
GO:0008494	1	1	100	1.13	0	0	0	0	0	0	0	0	0	1	1	1	1
GO:0008495	3	3	100	1.13	0	0	0	0	0	0	0	0	0	1	1	1	1
GO:0008497	1	1	100	1.13	0	0	0	0	0	0	0	0	0	1	1	1	1
GO:0008499	3	3	100	1.13	0	0	0	0	0	0	0	0	0	1	1	1	1
GO:0008501	3	3	100	1.13	0	0	0	0	0	0	0	0	0	1	1	1	1
GO:0008504	3	3	100	1.13	0	0	0	0	0	0	0	0	0	1	1	1	1
GO:0008507	1	1	100	1.13	0	0	0	0	0	0	0	0	0	1	1	1	1
GO:0008508	8	8	100	1.13	0	0	0	0	0	0	0	0	0	1	1	1	1
GO:0008510	1	1	100	1.13	0	0	0	0	0	0	0	0	0	1	1	1	1
GO:0008514	2	2	100	1.13	0	0	0	0	0	0	0	0	0	1	1	1	1
GO:0008517	1	1	100	1.13	0	0	0	0	0	0	0	0	0	1	1	1	1
GO:0008518	5	5	100	1.13	0	0	0	0	0	0	0	0	0	1	1	1	1
GO:0008519	1	1	100	1.13	0	0	0	0	0	0	0	0	0	1	1	1	1
GO:0008521	3	3	100	1.13	0	0	0	0	0	0	0	0	0	1	1	1	1
GO:0008526	1	1	100	1.13	0	0	0	0	0	0	0	0	0	1	1	1	1

GO:0008531	3	3	100	1.13	0	0	0	0	0	0	0	0	0	1	1	1	1
GO:0008532	1	1	100	1.13	0	0	0	0	0	0	0	0	0	1	1	1	1
GO:0008533	6	5	83.33	0.94	0	0	0	1	16.67	3.59	0	0	0	1	1	1	1
GO:0008534	2	1	50	0.57	1	50	14.32	0	0	0	0	0	0	1	1	1	1
GO:0008535	2	2	100	1.13	0	0	0	0	0	0	0	0	0	1	1	1	1
GO:0008536	10	9	90	1.02	1	10	2.86	0	0	0	0	0	0	1	1	1	1
GO:0008537	5	5	100	1.13	0	0	0	0	0	0	0	0	0	1	1	1	1
GO:0008538	5	5	100	1.13	0	0	0	0	0	0	0	0	0	1	1	1	1
GO:0008541	1	1	100	1.13	0	0	0	0	0	0	0	0	0	1	1	1	1
GO:0008543	3	3	100	1.13	0	0	0	0	0	0	0	0	0	1	1	1	1
GO:0008544	9	5	55.56	0.63	1	11.11	3.18	2	22.22	4.78	1	11.11	3.14	1	1	1	1
GO:0008545	17	17	100	1.13	0	0	0	0	0	0	0	0	0	1	1	1	1
GO:0008547	23	23	100	1.13	0	0	0	0	0	0	0	0	0	1	1	1	1
GO:0008549	2	2	100	1.13	0	0	0	0	0	0	0	0	0	1	1	1	1
GO:0008553	39	34	87.18	0.99	1	2.56	0.73	4	10.26	2.21	0	0	0	1	1	1	1
GO:0008559	1	1	100	1.13	0	0	0	0	0	0	0	0	0	1	1	1	1
GO:0008565	294	288	97.96	1.11	0	0	0	5	1.7	0.37	1	0.34	0.1	2.11E-07	1	1	1
GO:0008568	1	1	100	1.13	0	0	0	0	0	0	0	0	0	1	1	1	1
GO:0008569	3	3	100	1.13	0	0	0	0	0	0	0	0	0	1	1	1	1
GO:0008575	1	0	0	0	0	0	0	1	100	21.53	0	0	0	1	1	1	1
GO:0008577	1	1	100	1.13	0	0	0	0	0	0	0	0	0	1	1	1	1
GO:0008580	3	3	100	1.13	0	0	0	0	0	0	0	0	0	1	1	1	1
GO:0008583	4	4	100	1.13	0	0	0	0	0	0	0	0	0	1	1	1	1
GO:0008584	3	3	100	1.13	0	0	0	0	0	0	0	0	0	1	1	1	1
GO:0008593	1	1	100	1.13	0	0	0	0	0	0	0	0	0	1	1	1	1
GO:0008599	1	1	100	1.13	0	0	0	0	0	0	0	0	0	1	1	1	1
GO:0008600	2	2	100	1.13	0	0	0	0	0	0	0	0	0	1	1	1	1
GO:0008601	22	21	95.45	1.08	0	0	0	0	0	0	1	4.55	1.28	1	1	1	1
GO:0008602	17	17	100	1.13	0	0	0	0	0	0	0	0	0	1	1	1	1
GO:0008603	33	30	90.91	1.03	0	0	0	2	6.06	1.3	1	3.03	0.86	1	1	1	1
GO:0008604	17	17	100	1.13	0	0	0	0	0	0	0	0	0	1	1	1	1
GO:0008605	19	19	100	1.13	0	0	0	0	0	0	0	0	0	1	1	1	1
GO:0008606	18	18	100	1.13	0	0	0	0	0	0	0	0	0	1	1	1	1
GO:0008607	20	20	100	1.13	0	0	0	0	0	0	0	0	0	1	1	1	1
GO:0008609	2	2	100	1.13	0	0	0	0	0	0	0	0	0	1	1	1	1
GO:0008610	10	10	100	1.13	0	0	0	0	0	0	0	0	0	1	1	1	1
GO:0008611	4	4	100	1.13	0	0	0	0	0	0	0	0	0	1	1	1	1
GO:0008612	2	0	0	0	0	0	0	1	50	10.76	1	50	14.11	1	1	1	1
GO:0008615	1	1	100	1.13	0	0	0	0	0	0	0	0	0	1	1	1	1
GO:0008616	1	1	100	1.13	0	0	0	0	0	0	0	0	0	1	1	1	1
GO:0008617	1	1	100	1.13	0	0	0	0	0	0	0	0	0	1	1	1	1

GO:0008624	7	7	100	1.13	0	0	0	0	0	0	0	0	0	1	1	1	1
GO:0008625	14	14	100	1.13	0	0	0	0	0	0	0	0	0	1	1	1	1
GO:0008628	1	1	100	1.13	0	0	0	0	0	0	0	0	0	1	1	1	1
GO:0008629	8	8	100	1.13	0	0	0	0	0	0	0	0	0	1	1	1	1
GO:0008630	2	2	100	1.13	0	0	0	0	0	0	0	0	0	1	1	1	1
GO:0008631	2	2	100	1.13	0	0	0	0	0	0	0	0	0	1	1	1	1
GO:0008632	8	8	100	1.13	0	0	0	0	0	0	0	0	0	1	1	1	1
GO:0008635	4	4	100	1.13	0	0	0	0	0	0	0	0	0	1	1	1	1
GO:0008637	6	6	100	1.13	0	0	0	0	0	0	0	0	0	1	1	1	1
GO:0008640	3	3	100	1.13	0	0	0	0	0	0	0	0	0	1	1	1	1
GO:0008642	10	10	100	1.13	0	0	0	0	0	0	0	0	0	1	1	1	1
GO:0008643	34	30	88.24	1	2	5.88	1.68	0	0	0	2	5.88	1.66	1	1	1	1
GO:0008649	7	7	100	1.13	0	0	0	0	0	0	0	0	0	1	1	1	1
GO:0008650	3	3	100	1.13	0	0	0	0	0	0	0	0	0	1	1	1	1
GO:0008652	4	4	100	1.13	0	0	0	0	0	0	0	0	0	1	1	1	1
GO:0008654	26	23	88.46	1	1	3.85	1.1	2	7.69	1.66	0	0	0	1	1	1	1
GO:0008656	9	9	100	1.13	0	0	0	0	0	0	0	0	0	1	1	1	1
GO:0008665	1	1	100	1.13	0	0	0	0	0	0	0	0	0	1	1	1	1
GO:0008667	5	3	60	0.68	1	20	5.73	1	20	4.31	0	0	0	1	1	1	1
GO:0008668	1	1	100	1.13	0	0	0	0	0	0	0	0	0	1	1	1	1
GO:0008670	2	2	100	1.13	0	0	0	0	0	0	0	0	0	1	1	1	1
GO:0008705	2	1	50	0.57	1	50	14.32	0	0	0	0	0	0	1	1	1	1
GO:0008717	7	7	100	1.13	0	0	0	0	0	0	0	0	0	1	1	1	1
GO:0008722	1	1	100	1.13	0	0	0	0	0	0	0	0	0	1	1	1	1
GO:0008723	1	1	100	1.13	0	0	0	0	0	0	0	0	0	1	1	1	1
GO:0008736	1	1	100	1.13	0	0	0	0	0	0	0	0	0	1	1	1	1
GO:0008746	6	6	100	1.13	0	0	0	0	0	0	0	0	0	1	1	1	1
GO:0008750	1	1	100	1.13	0	0	0	0	0	0	0	0	0	1	1	1	1
GO:0008753	3	3	100	1.13	0	0	0	0	0	0	0	0	0	1	1	1	1
GO:0008757	75	72	96	1.09	0	0	0	3	4	0.86	0	0	0	1	1	1	1
GO:0008759	2	1	50	0.57	0	0	0	1	50	10.76	0	0	0	1	1	1	1
GO:0008768	1	1	100	1.13	0	0	0	0	0	0	0	0	0	1	1	1	1
GO:0008778	2	2	100	1.13	0	0	0	0	0	0	0	0	0	1	1	1	1
GO:0008781	1	1	100	1.13	0	0	0	0	0	0	0	0	0	1	1	1	1
GO:0008784	3	3	100	1.13	0	0	0	0	0	0	0	0	0	1	1	1	1
GO:0008796	4	4	100	1.13	0	0	0	0	0	0	0	0	0	1	1	1	1
GO:0008798	2	2	100	1.13	0	0	0	0	0	0	0	0	0	1	1	1	1
GO:0008803	2	2	100	1.13	0	0	0	0	0	0	0	0	0	1	1	1	1
GO:0008808	2	2	100	1.13	0	0	0	0	0	0	0	0	0	1	1	1	1
GO:0008812	1	1	100	1.13	0	0	0	0	0	0	0	0	0	1	1	1	1
GO:0008819	17	17	100	1.13	0	0	0	0	0	0	0	0	0	1	1	1	1

GO:0008822	1	1	100	1.13	0	0	0	0	0	0	0	0	0	1	1	1	1
GO:0008828	1	1	100	1.13	0	0	0	0	0	0	0	0	0	1	1	1	1
GO:0008831	1	1	100	1.13	0	0	0	0	0	0	0	0	0	1	1	1	1
GO:0008875	5	1	20	0.23	1	20	5.73	2	40	8.61	1	20	5.64	1	1	1	1
GO:0008878	2	1	50	0.57	0	0	0	1	50	10.76	0	0	0	1	1	1	1
GO:0008889	9	9	100	1.13	0	0	0	0	0	0	0	0	0	1	1	1	1
GO:0008890	5	5	100	1.13	0	0	0	0	0	0	0	0	0	1	1	1	1
GO:0008892	2	2	100	1.13	0	0	0	0	0	0	0	0	0	1	1	1	1
GO:0008898	4	3	75	0.85	1	25	7.16	0	0	0	0	0	0	1	1	1	1
GO:0008900	2	2	100	1.13	0	0	0	0	0	0	0	0	0	1	1	1	1
GO:0008905	1	1	100	1.13	0	0	0	0	0	0	0	0	0	1	1	1	1
GO:0008907	4	4	100	1.13	0	0	0	0	0	0	0	0	0	1	1	1	1
GO:0008920	4	4	100	1.13	0	0	0	0	0	0	0	0	0	1	1	1	1
GO:0008921	4	4	100	1.13	0	0	0	0	0	0	0	0	0	1	1	1	1
GO:0008932	2	2	100	1.13	0	0	0	0	0	0	0	0	0	1	1	1	1
GO:0008934	13	13	100	1.13	0	0	0	0	0	0	0	0	0	1	1	1	1
GO:0008955	1	1	100	1.13	0	0	0	0	0	0	0	0	0	1	1	1	1
GO:0008967	1	1	100	1.13	0	0	0	0	0	0	0	0	0	1	1	1	1
GO:0008970	1	1	100	1.13	0	0	0	0	0	0	0	0	0	1	1	1	1
GO:0008974	2	2	100	1.13	0	0	0	0	0	0	0	0	0	1	1	1	1
GO:0008978	3	3	100	1.13	0	0	0	0	0	0	0	0	0	1	1	1	1
GO:0009008	3	3	100	1.13	0	0	0	0	0	0	0	0	0	1	1	1	1
GO:0009017	2	1	50	0.57	0	0	0	1	50	10.76	0	0	0	1	1	1	1
GO:0009022	1	1	100	1.13	0	0	0	0	0	0	0	0	0	1	1	1	1
GO:0009045	1	1	100	1.13	0	0	0	0	0	0	0	0	0	1	1	1	1
GO:0009048	1	1	100	1.13	0	0	0	0	0	0	0	0	0	1	1	1	1
GO:0009049	3	3	100	1.13	0	0	0	0	0	0	0	0	0	1	1	1	1
GO:0009051	1	1	100	1.13	0	0	0	0	0	0	0	0	0	1	1	1	1
GO:0009052	1	1	100	1.13	0	0	0	0	0	0	0	0	0	1	1	1	1
GO:0009054	2	2	100	1.13	0	0	0	0	0	0	0	0	0	1	1	1	1
GO:0009055	5	3	60	0.68	1	20	5.73	1	20	4.31	0	0	0	1	1	1	1
GO:0009056	2	1	50	0.57	0	0	0	0	0	0	1	50	14.11	1	1	1	1
GO:0009058	71	67	94.37	1.07	3	4.23	1.21	1	1.41	0.3	0	0	0	1	1	1	1
GO:0009060	19	18	94.74	1.07	1	5.26	1.51	0	0	0	0	0	0	1	1	1	1
GO:0009063	1	1	100	1.13	0	0	0	0	0	0	0	0	0	1	1	1	1
GO:0009072	6	6	100	1.13	0	0	0	0	0	0	0	0	0	1	1	1	1
GO:0009073	5	5	100	1.13	0	0	0	0	0	0	0	0	0	1	1	1	1
GO:0009081	3	3	100	1.13	0	0	0	0	0	0	0	0	0	1	1	1	1
GO:0009082	5	5	100	1.13	0	0	0	0	0	0	0	0	0	1	1	1	1
GO:0009083	1	1	100	1.13	0	0	0	0	0	0	0	0	0	1	1	1	1
GO:0009086	5	4	80	0.91	1	20	5.73	0	0	0	0	0	0	1	1	1	1

GO:0009088	1	1	100	1.13	0	0	0	0	0	0	0	0	0	1	1	1	1
GO:0009094	1	1	100	1.13	0	0	0	0	0	0	0	0	0	1	1	1	1
GO:0009101	3	3	100	1.13	0	0	0	0	0	0	0	0	0	1	1	1	1
GO:0009103	4	4	100	1.13	0	0	0	0	0	0	0	0	0	1	1	1	1
GO:0009105	3	3	100	1.13	0	0	0	0	0	0	0	0	0	1	1	1	1
GO:0009107	4	4	100	1.13	0	0	0	0	0	0	0	0	0	1	1	1	1
GO:0009108	3	3	100	1.13	0	0	0	0	0	0	0	0	0	1	1	1	1
GO:0009113	22	22	100	1.13	0	0	0	0	0	0	0	0	0	1	1	1	1
GO:0009116	15	15	100	1.13	0	0	0	0	0	0	0	0	0	1	1	1	1
GO:0009117	15	14	93.33	1.06	0	0	0	0	0	0	1	6.67	1.88	1	1	1	1
GO:0009152	2	2	100	1.13	0	0	0	0	0	0	0	0	0	1	1	1	1
GO:0009156	7	7	100	1.13	0	0	0	0	0	0	0	0	0	1	1	1	1
GO:0009165	13	13	100	1.13	0	0	0	0	0	0	0	0	0	1	1	1	1
GO:0009166	3	3	100	1.13	0	0	0	0	0	0	0	0	0	1	1	1	1
GO:0009168	1	0	0	0	0	0	0	0	0	0	1	100	28.22	1	1	1	1
GO:0009181	1	1	100	1.13	0	0	0	0	0	0	0	0	0	1	1	1	1
GO:0009186	4	4	100	1.13	0	0	0	0	0	0	0	0	0	1	1	1	1
GO:0009191	1	1	100	1.13	0	0	0	0	0	0	0	0	0	1	1	1	1
GO:0009220	3	3	100	1.13	0	0	0	0	0	0	0	0	0	1	1	1	1
GO:0009225	9	9	100	1.13	0	0	0	0	0	0	0	0	0	1	1	1	1
GO:0009229	1	1	100	1.13	0	0	0	0	0	0	0	0	0	1	1	1	1
GO:0009231	3	3	100	1.13	0	0	0	0	0	0	0	0	0	1	1	1	1
GO:0009239	5	3	60	0.68	1	20	5.73	1	20	4.31	0	0	0	1	1	1	1
GO:0009247	2	2	100	1.13	0	0	0	0	0	0	0	0	0	1	1	1	1
GO:0009249	2	2	100	1.13	0	0	0	0	0	0	0	0	0	1	1	1	1
GO:0009257	2	2	100	1.13	0	0	0	0	0	0	0	0	0	1	1	1	1
GO:0009262	3	3	100	1.13	0	0	0	0	0	0	0	0	0	1	1	1	1
GO:0009264	1	1	100	1.13	0	0	0	0	0	0	0	0	0	1	1	1	1
GO:0009266	1	0	0	0	0	0	0	0	0	0	1	100	28.22	1	1	1	1
GO:0009298	4	4	100	1.13	0	0	0	0	0	0	0	0	0	1	1	1	1
GO:0009301	5	5	100	1.13	0	0	0	0	0	0	0	0	0	1	1	1	1
GO:0009303	1	1	100	1.13	0	0	0	0	0	0	0	0	0	1	1	1	1
GO:0009306	27	27	100	1.13	0	0	0	0	0	0	0	0	0	1	1	1	1
GO:0009311	2	2	100	1.13	0	0	0	0	0	0	0	0	0	1	1	1	1
GO:0009312	7	7	100	1.13	0	0	0	0	0	0	0	0	0	1	1	1	1
GO:0009314	5	5	100	1.13	0	0	0	0	0	0	0	0	0	1	1	1	1
GO:0009315	7	6	85.71	0.97	1	14.29	4.09	0	0	0	0	0	0	1	1	1	1
GO:0009320	7	7	100	1.13	0	0	0	0	0	0	0	0	0	1	1	1	1
GO:0009331	5	5	100	1.13	0	0	0	0	0	0	0	0	0	1	1	1	1
GO:0009341	8	8	100	1.13	0	0	0	0	0	0	0	0	0	1	1	1	1
GO:0009348	1	0	0	0	0	0	0	1	100	21.53	0	0	0	1	1	1	1

GO:0009353	1	1	100	1.13	0	0	0	0	0	0	0	0	0	1	1	1	1
GO:0009374	4	4	100	1.13	0	0	0	0	0	0	0	0	0	1	1	1	1
GO:0009383	3	3	100	1.13	0	0	0	0	0	0	0	0	0	1	1	1	1
GO:0009384	1	1	100	1.13	0	0	0	0	0	0	0	0	0	1	1	1	1
GO:0009395	3	2	66.67	0.75	1	33.33	9.54	0	0	0	0	0	0	1	1	1	1
GO:0009396	12	12	100	1.13	0	0	0	0	0	0	0	0	0	1	1	1	1
GO:0009399	5	5	100	1.13	0	0	0	0	0	0	0	0	0	1	1	1	1
GO:0009401	12	9	75	0.85	2	16.67	4.77	1	8.33	1.79	0	0	0	1	1	1	1
GO:0009405	21	18	85.71	0.97	2	9.52	2.73	1	4.76	1.03	0	0	0	1	1	1	1
GO:0009406	7	5	71.43	0.81	1	14.29	4.09	1	14.29	3.08	0	0	0	1	1	1	1
GO:0009408	30	26	86.67	0.98	2	6.67	1.91	1	3.33	0.72	1	3.33	0.94	1	1	1	1
GO:0009409	4	4	100	1.13	0	0	0	0	0	0	0	0	0	1	1	1	1
GO:0009411	2	2	100	1.13	0	0	0	0	0	0	0	0	0	1	1	1	1
GO:0009416	3	3	100	1.13	0	0	0	0	0	0	0	0	0	1	1	1	1
GO:0009434	3	3	100	1.13	0	0	0	0	0	0	0	0	0	1	1	1	1
GO:0009435	2	2	100	1.13	0	0	0	0	0	0	0	0	0	1	1	1	1
GO:0009437	7	7	100	1.13	0	0	0	0	0	0	0	0	0	1	1	1	1
GO:0009448	1	1	100	1.13	0	0	0	0	0	0	0	0	0	1	1	1	1
GO:0009451	2	2	100	1.13	0	0	0	0	0	0	0	0	0	1	1	1	1
GO:0009457	3	2	66.67	0.75	0	0	0	1	33.33	7.18	0	0	0	1	1	1	1
GO:0009460	4	3	75	0.85	0	0	0	0	0	0	1	25	7.06	1	1	1	1
GO:0009461	3	3	100	1.13	0	0	0	0	0	0	0	0	0	1	1	1	1
GO:0009481	11	10	90.91	1.03	0	0	0	1	9.09	1.96	0	0	0	1	1	1	1
GO:0009482	11	10	90.91	1.03	0	0	0	1	9.09	1.96	0	0	0	1	1	1	1
GO:0009483	11	10	90.91	1.03	0	0	0	1	9.09	1.96	0	0	0	1	1	1	1
GO:0009485	11	10	90.91	1.03	0	0	0	1	9.09	1.96	0	0	0	1	1	1	1
GO:0009487	2	1	50	0.57	0	0	0	1	50	10.76	0	0	0	1	1	1	1
GO:0009489	1	1	100	1.13	0	0	0	0	0	0	0	0	0	1	1	1	1
GO:0009492	3	3	100	1.13	0	0	0	0	0	0	0	0	0	1	1	1	1
GO:0009497	5	4	80	0.91	0	0	0	0	0	0	1	20	5.64	1	1	1	1
GO:0009589	1	1	100	1.13	0	0	0	0	0	0	0	0	0	1	1	1	1
GO:0009592	1	1	100	1.13	0	0	0	0	0	0	0	0	0	1	1	1	1
GO:0009597	1	0	0	0	0	0	0	1	100	21.53	0	0	0	1	1	1	1
GO:0009605	1	1	100	1.13	0	0	0	0	0	0	0	0	0	1	1	1	1
GO:0009607	2	0	0	0	0	0	0	2	100	21.53	0	0	0	1	1	1	1
GO:0009611	2	2	100	1.13	0	0	0	0	0	0	0	0	0	1	1	1	1
GO:0009613	10	10	100	1.13	0	0	0	0	0	0	0	0	0	1	1	1	1
GO:0009615	4	2	50	0.57	2	50	14.32	0	0	0	0	0	0	1	1	1	1
GO:0009617	7	5	71.43	0.81	0	0	0	2	28.57	6.15	0	0	0	1	1	1	1
GO:0009618	13	7	53.85	0.61	0	0	0	0	0	0	6	46.15	13.03	1	1	1	0.016775835
GO:0009619	1	1	100	1.13	0	0	0	0	0	0	0	0	0	1	1	1	1

GO:0009631	1	1	100	1.13	0	0	0	0	0	0	0	0	0	1	1	1	1
GO:0009634	4	4	100	1.13	0	0	0	0	0	0	0	0	0	1	1	1	1
GO:0009636	14	14	100	1.13	0	0	0	0	0	0	0	0	0	1	1	1	1
GO:0009653	4	4	100	1.13	0	0	0	0	0	0	0	0	0	1	1	1	1
GO:0009725	1	1	100	1.13	0	0	0	0	0	0	0	0	0	1	1	1	1
GO:0009790	4	4	100	1.13	0	0	0	0	0	0	0	0	0	1	1	1	1
GO:0009791	2	2	100	1.13	0	0	0	0	0	0	0	0	0	1	1	1	1
GO:0009795	1	1	100	1.13	0	0	0	0	0	0	0	0	0	1	1	1	1
GO:0009854	1	0	0	0	0	0	0	0	0	0	1	100	28.22	1	1	1	1
GO:0009887	64	58	90.63	1.03	2	3.13	0.89	2	3.13	0.67	2	3.13	0.88	1	1	1	1
GO:0009897	43	19	44.19	0.5	3	6.98	2	15	34.88	7.51	6	13.95	3.94	1	1	3.71E-06	1
GO:0009898	3	3	100	1.13	0	0	0	0	0	0	0	0	0	1	1	1	1
GO:0009948	1	1	100	1.13	0	0	0	0	0	0	0	0	0	1	1	1	1
GO:0009952	2	2	100	1.13	0	0	0	0	0	0	0	0	0	1	1	1	1
GO:0009953	2	2	100	1.13	0	0	0	0	0	0	0	0	0	1	1	1	1
GO:0009954	1	1	100	1.13	0	0	0	0	0	0	0	0	0	1	1	1	1
GO:0009966	1	0	0	0	0	0	0	1	100	21.53	0	0	0	1	1	1	1
GO:0009968	1	0	0	0	0	0	0	1	100	21.53	0	0	0	1	1	1	1
GO:0009986	10	5	50	0.57	0	0	0	3	30	6.46	2	20	5.64	1	1	1	1
GO:0010003	8	5	62.5	0.71	1	12.5	3.58	1	12.5	2.69	1	12.5	3.53	1	1	1	1
GO:0010008	3	2	66.67	0.75	0	0	0	1	33.33	7.18	0	0	0	1	1	1	1
GO:0010165	3	3	100	1.13	0	0	0	0	0	0	0	0	0	1	1	1	1
GO:0012501	1	1	100	1.13	0	0	0	0	0	0	0	0	0	1	1	1	1
GO:0012505	4	4	100	1.13	0	0	0	0	0	0	0	0	0	1	1	1	1
GO:0012506	5	4	80	0.91	0	0	0	1	20	4.31	0	0	0	1	1	1	1
GO:0015008	9	7	77.78	0.88	0	0	0	2	22.22	4.78	0	0	0	1	1	1	1
GO:0015012	1	1	100	1.13	0	0	0	0	0	0	0	0	0	1	1	1	1
GO:0015014	3	3	100	1.13	0	0	0	0	0	0	0	0	0	1	1	1	1
GO:0015017	2	2	100	1.13	0	0	0	0	0	0	0	0	0	1	1	1	1
GO:0015018	5	5	100	1.13	0	0	0	0	0	0	0	0	0	1	1	1	1
GO:0015020	7	5	71.43	0.81	0	0	0	1	14.29	3.08	1	14.29	4.03	1	1	1	1
GO:0015025	2	2	100	1.13	0	0	0	0	0	0	0	0	0	1	1	1	1
GO:0015026	3	0	0	0	2	66.67	19.09	1	33.33	7.18	0	0	0	1	1	1	1
GO:0015027	3	3	100	1.13	0	0	0	0	0	0	0	0	0	1	1	1	1
GO:0015030	5	5	100	1.13	0	0	0	0	0	0	0	0	0	1	1	1	1
GO:0015031	388	377	97.16	1.1	4	1.03	0.3	6	1.55	0.33	1	0.26	0.07	3.36E-08	1	1	1
GO:0015035	7	6	85.71	0.97	0	0	0	1	14.29	3.08	0	0	0	1	1	1	1
GO:0015036	29	28	96.55	1.09	0	0	0	0	0	0	1	3.45	0.97	1	1	1	1
GO:0015056	1	1	100	1.13	0	0	0	0	0	0	0	0	0	1	1	1	1
GO:0015057	6	6	100	1.13	0	0	0	0	0	0	0	0	0	1	1	1	1
GO:0015058	1	0	0	0	0	0	0	0	0	0	1	100	28.22	1	1	1	1

GO:0015065	1	1	100	1.13	0	0	0	0	0	0	0	0	0	1	1	1	1
GO:0015068	3	3	100	1.13	0	0	0	0	0	0	0	0	0	1	1	1	1
GO:0015071	15	14	93.33	1.06	1	6.67	1.91	0	0	0	0	0	0	1	1	1	1
GO:0015072	2	1	50	0.57	1	50	14.32	0	0	0	0	0	0	1	1	1	1
GO:0015073	3	2	66.67	0.75	1	33.33	9.54	0	0	0	0	0	0	1	1	1	1
GO:0015074	10	10	100	1.13	0	0	0	0	0	0	0	0	0	1	1	1	1
GO:0015075	1	1	100	1.13	0	0	0	0	0	0	0	0	0	1	1	1	1
GO:0015076	7	6	85.71	0.97	1	14.29	4.09	0	0	0	0	0	0	1	1	1	1
GO:0015077	3	3	100	1.13	0	0	0	0	0	0	0	0	0	1	1	1	1
GO:0015078	64	61	95.31	1.08	1	1.56	0.45	2	3.13	0.67	0	0	0	1	1	1	1
GO:0015085	1	1	100	1.13	0	0	0	0	0	0	0	0	0	1	1	1	1
GO:0015087	4	4	100	1.13	0	0	0	0	0	0	0	0	0	1	1	1	1
GO:0015097	2	2	100	1.13	0	0	0	0	0	0	0	0	0	1	1	1	1
GO:0015108	3	1	33.33	0.38	0	0	0	1	33.33	7.18	1	33.33	9.41	1	1	1	1
GO:0015111	2	1	50	0.57	0	0	0	0	0	0	1	50	14.11	1	1	1	1
GO:0015116	1	0	0	0	0	0	0	1	100	21.53	0	0	0	1	1	1	1
GO:0015125	3	0	0	0	0	0	0	0	0	0	3	100	28.22	1	1	1	0.21002992
GO:0015132	1	1	100	1.13	0	0	0	0	0	0	0	0	0	1	1	1	1
GO:0015137	1	1	100	1.13	0	0	0	0	0	0	0	0	0	1	1	1	1
GO:0015169	2	1	50	0.57	1	50	14.32	0	0	0	0	0	0	1	1	1	1
GO:0015171	6	5	83.33	0.94	0	0	0	1	16.67	3.59	0	0	0	1	1	1	1
GO:0015174	1	1	100	1.13	0	0	0	0	0	0	0	0	0	1	1	1	1
GO:0015175	3	3	100	1.13	0	0	0	0	0	0	0	0	0	1	1	1	1
GO:0015180	1	1	100	1.13	0	0	0	0	0	0	0	0	0	1	1	1	1
GO:0015181	1	1	100	1.13	0	0	0	0	0	0	0	0	0	1	1	1	1
GO:0015186	1	1	100	1.13	0	0	0	0	0	0	0	0	0	1	1	1	1
GO:0015187	3	3	100	1.13	0	0	0	0	0	0	0	0	0	1	1	1	1
GO:0015189	1	1	100	1.13	0	0	0	0	0	0	0	0	0	1	1	1	1
GO:0015193	1	1	100	1.13	0	0	0	0	0	0	0	0	0	1	1	1	1
GO:0015197	1	1	100	1.13	0	0	0	0	0	0	0	0	0	1	1	1	1
GO:0015198	8	8	100	1.13	0	0	0	0	0	0	0	0	0	1	1	1	1
GO:0015204	1	1	100	1.13	0	0	0	0	0	0	0	0	0	1	1	1	1
GO:0015207	5	5	100	1.13	0	0	0	0	0	0	0	0	0	1	1	1	1
GO:0015227	1	1	100	1.13	0	0	0	0	0	0	0	0	0	1	1	1	1
GO:0015232	2	2	100	1.13	0	0	0	0	0	0	0	0	0	1	1	1	1
GO:0015234	1	1	100	1.13	0	0	0	0	0	0	0	0	0	1	1	1	1
GO:0015238	3	3	100	1.13	0	0	0	0	0	0	0	0	0	1	1	1	1
GO:0015245	1	1	100	1.13	0	0	0	0	0	0	0	0	0	1	1	1	1
GO:0015247	3	2	66.67	0.75	0	0	0	0	0	0	1	33.33	9.41	1	1	1	1
GO:0015250	5	3	60	0.68	1	20	5.73	0	0	0	1	20	5.64	1	1	1	1
GO:0015267	2	2	100	1.13	0	0	0	0	0	0	0	0	0	1	1	1	1

GO:0015269	6	6	100	1.13	0	0	0	0	0	0	0	0	0	1	1	1	1
GO:0015272	2	2	100	1.13	0	0	0	0	0	0	0	0	0	1	1	1	1
GO:0015277	3	3	100	1.13	0	0	0	0	0	0	0	0	0	1	1	1	1
GO:0015278	1	1	100	1.13	0	0	0	0	0	0	0	0	0	1	1	1	1
GO:0015279	2	2	100	1.13	0	0	0	0	0	0	0	0	0	1	1	1	1
GO:0015280	3	2	66.67	0.75	1	33.33	9.54	0	0	0	0	0	0	1	1	1	1
GO:0015285	9	6	66.67	0.75	1	11.11	3.18	0	0	0	2	22.22	6.27	1	1	1	1
GO:0015288	27	26	96.3	1.09	1	3.7	1.06	0	0	0	0	0	0	1	1	1	1
GO:0015290	3	3	100	1.13	0	0	0	0	0	0	0	0	0	1	1	1	1
GO:0015293	41	37	90.24	1.02	2	4.88	1.4	1	2.44	0.53	1	2.44	0.69	1	1	1	1
GO:0015294	4	4	100	1.13	0	0	0	0	0	0	0	0	0	1	1	1	1
GO:0015297	4	3	75	0.85	0	0	0	0	0	0	1	25	7.06	1	1	1	1
GO:0015299	9	7	77.78	0.88	0	0	0	0	0	0	2	22.22	6.27	1	1	1	1
GO:0015302	2	2	100	1.13	0	0	0	0	0	0	0	0	0	1	1	1	1
GO:0015321	2	1	50	0.57	0	0	0	1	50	10.76	0	0	0	1	1	1	1
GO:0015326	1	1	100	1.13	0	0	0	0	0	0	0	0	0	1	1	1	1
GO:0015349	3	3	100	1.13	0	0	0	0	0	0	0	0	0	1	1	1	1
GO:0015355	6	6	100	1.13	0	0	0	0	0	0	0	0	0	1	1	1	1
GO:0015359	5	4	80	0.91	0	0	0	1	20	4.31	0	0	0	1	1	1	1
GO:0015362	1	1	100	1.13	0	0	0	0	0	0	0	0	0	1	1	1	1
GO:0015376	1	1	100	1.13	0	0	0	0	0	0	0	0	0	1	1	1	1
GO:0015377	12	12	100	1.13	0	0	0	0	0	0	0	0	0	1	1	1	1
GO:0015379	1	1	100	1.13	0	0	0	0	0	0	0	0	0	1	1	1	1
GO:0015380	8	8	100	1.13	0	0	0	0	0	0	0	0	0	1	1	1	1
GO:0015384	1	1	100	1.13	0	0	0	0	0	0	0	0	0	1	1	1	1
GO:0015385	7	4	57.14	0.65	0	0	0	0	0	0	3	42.86	12.1	1	1	1	1
GO:0015433	1	1	100	1.13	0	0	0	0	0	0	0	0	0	1	1	1	1
GO:0015450	32	30	93.75	1.06	0	0	0	2	6.25	1.35	0	0	0	1	1	1	1
GO:0015457	1	1	100	1.13	0	0	0	0	0	0	0	0	0	1	1	1	1
GO:0015459	4	4	100	1.13	0	0	0	0	0	0	0	0	0	1	1	1	1
GO:0015467	2	2	100	1.13	0	0	0	0	0	0	0	0	0	1	1	1	1
GO:0015482	1	1	100	1.13	0	0	0	0	0	0	0	0	0	1	1	1	1
GO:0015485	7	6	85.71	0.97	0	0	0	1	14.29	3.08	0	0	0	1	1	1	1
GO:0015499	1	1	100	1.13	0	0	0	0	0	0	0	0	0	1	1	1	1
GO:0015520	2	1	50	0.57	0	0	0	1	50	10.76	0	0	0	1	1	1	1
GO:0015559	1	1	100	1.13	0	0	0	0	0	0	0	0	0	1	1	1	1
GO:0015629	122	112	91.8	1.04	5	4.1	1.17	3	2.46	0.53	2	1.64	0.46	1	1	1	1
GO:0015630	15	15	100	1.13	0	0	0	0	0	0	0	0	0	1	1	1	1
GO:0015631	8	7	87.5	0.99	1	12.5	3.58	0	0	0	0	0	0	1	1	1	1
GO:0015645	3	0	0	0	0	0	0	2	66.67	14.35	1	33.33	9.41	1	1	1	1
GO:0015662	21	19	90.48	1.02	0	0	0	0	0	0	2	9.52	2.69	1	1	1	1

GO:0015671	16	7	43.75	0.5	8	50	14.32	0	0	0	1	6.25	1.76	1	0.000114918	1	1
GO:0015672	3	3	100	1.13	0	0	0	0	0	0	0	0	0	1	1	1	1
GO:0015680	1	1	100	1.13	0	0	0	0	0	0	0	0	0	1	1	1	1
GO:0015694	2	2	100	1.13	0	0	0	0	0	0	0	0	0	1	1	1	1
GO:0015701	2	2	100	1.13	0	0	0	0	0	0	0	0	0	1	1	1	1
GO:0015711	7	7	100	1.13	0	0	0	0	0	0	0	0	0	1	1	1	1
GO:0015718	3	2	66.67	0.75	0	0	0	1	33.33	7.18	0	0	0	1	1	1	1
GO:0015721	3	1	33.33	0.38	0	0	0	0	0	0	2	66.67	18.82	1	1	1	1
GO:0015722	1	1	100	1.13	0	0	0	0	0	0	0	0	0	1	1	1	1
GO:0015724	1	1	100	1.13	0	0	0	0	0	0	0	0	0	1	1	1	1
GO:0015732	1	1	100	1.13	0	0	0	0	0	0	0	0	0	1	1	1	1
GO:0015758	9	8	88.89	1.01	0	0	0	0	0	0	1	11.11	3.14	1	1	1	1
GO:0015780	3	3	100	1.13	0	0	0	0	0	0	0	0	0	1	1	1	1
GO:0015794	2	1	50	0.57	1	50	14.32	0	0	0	0	0	0	1	1	1	1
GO:0015808	1	1	100	1.13	0	0	0	0	0	0	0	0	0	1	1	1	1
GO:0015809	1	1	100	1.13	0	0	0	0	0	0	0	0	0	1	1	1	1
GO:0015816	3	3	100	1.13	0	0	0	0	0	0	0	0	0	1	1	1	1
GO:0015819	1	1	100	1.13	0	0	0	0	0	0	0	0	0	1	1	1	1
GO:0015824	1	1	100	1.13	0	0	0	0	0	0	0	0	0	1	1	1	1
GO:0015833	1	1	100	1.13	0	0	0	0	0	0	0	0	0	1	1	1	1
GO:0015840	1	1	100	1.13	0	0	0	0	0	0	0	0	0	1	1	1	1
GO:0015844	3	3	100	1.13	0	0	0	0	0	0	0	0	0	1	1	1	1
GO:0015858	3	3	100	1.13	0	0	0	0	0	0	0	0	0	1	1	1	1
GO:0015872	1	1	100	1.13	0	0	0	0	0	0	0	0	0	1	1	1	1
GO:0015875	1	0	0	0	1	100	28.63	0	0	0	0	0	0	1	1	1	1
GO:0015888	1	1	100	1.13	0	0	0	0	0	0	0	0	0	1	1	1	1
GO:0015904	2	1	50	0.57	0	0	0	1	50	10.76	0	0	0	1	1	1	1
GO:0015908	2	2	100	1.13	0	0	0	0	0	0	0	0	0	1	1	1	1
GO:0015909	2	2	100	1.13	0	0	0	0	0	0	0	0	0	1	1	1	1
GO:0015917	3	2	66.67	0.75	0	0	0	0	0	0	1	33.33	9.41	1	1	1	1
GO:0015922	3	3	100	1.13	0	0	0	0	0	0	0	0	0	1	1	1	1
GO:0015926	2	2	100	1.13	0	0	0	0	0	0	0	0	0	1	1	1	1
GO:0015934	5	4	80	0.91	1	20	5.73	0	0	0	0	0	0	1	1	1	1
GO:0015935	7	7	100	1.13	0	0	0	0	0	0	0	0	0	1	1	1	1
GO:0015937	5	5	100	1.13	0	0	0	0	0	0	0	0	0	1	1	1	1
GO:0015961	1	1	100	1.13	0	0	0	0	0	0	0	0	0	1	1	1	1
GO:0015979	2	2	100	1.13	0	0	0	0	0	0	0	0	0	1	1	1	1
GO:0015980	1	0	0	0	0	0	0	1	100	21.53	0	0	0	1	1	1	1
GO:0015983	1	1	100	1.13	0	0	0	0	0	0	0	0	0	1	1	1	1
GO:0015986	82	78	95.12	1.08	1	1.22	0.35	3	3.66	0.79	0	0	0	1	1	1	1
GO:0015988	10	10	100	1.13	0	0	0	0	0	0	0	0	0	1	1	1	1

GO:0015991	10	10	100	1.13	0	0	0	0	0	0	0	0	0	1	1	1	1
GO:0015992	78	75	96.15	1.09	1	1.28	0.37	2	2.56	0.55	0	0	0	1	1	1	1
GO:0015999	2	2	100	1.13	0	0	0	0	0	0	0	0	0	1	1	1	1
GO:0016000	2	2	100	1.13	0	0	0	0	0	0	0	0	0	1	1	1	1
GO:0016003	7	6	85.71	0.97	0	0	0	1	14.29	3.08	0	0	0	1	1	1	1
GO:0016005	2	2	100	1.13	0	0	0	0	0	0	0	0	0	1	1	1	1
GO:0016011	2	2	100	1.13	0	0	0	0	0	0	0	0	0	1	1	1	1
GO:0016012	1	1	100	1.13	0	0	0	0	0	0	0	0	0	1	1	1	1
GO:0016019	1	1	100	1.13	0	0	0	0	0	0	0	0	0	1	1	1	1
GO:0016020	1690	1464	86.63	0.98	64	3.79	1.08	77	4.56	0.98	85	5.03	1.42	1	1	1	1
GO:0016021	1641	1428	87.02	0.99	65	3.96	1.13	77	4.69	1.01	71	4.33	1.22	1	1	1	1
GO:0016023	36	34	94.44	1.07	0	0	0	2	5.56	1.2	0	0	0	1	1	1	1
GO:0016025	1	1	100	1.13	0	0	0	0	0	0	0	0	0	1	1	1	1
GO:0016034	1	1	100	1.13	0	0	0	0	0	0	0	0	0	1	1	1	1
GO:0016035	1	1	100	1.13	0	0	0	0	0	0	0	0	0	1	1	1	1
GO:0016042	68	55	80.88	0.92	3	4.41	1.26	6	8.82	1.9	4	5.88	1.66	1	1	1	1
GO:0016044	3	3	100	1.13	0	0	0	0	0	0	0	0	0	1	1	1	1
GO:0016049	11	8	72.73	0.82	1	9.09	2.6	1	9.09	1.96	1	9.09	2.57	1	1	1	1
GO:0016051	7	7	100	1.13	0	0	0	0	0	0	0	0	0	1	1	1	1
GO:0016055	25	24	96	1.09	0	0	0	1	4	0.86	0	0	0	1	1	1	1
GO:0016064	17	12	70.59	0.8	0	0	0	5	29.41	6.33	0	0	0	1	1	1	1
GO:0016066	15	11	73.33	0.83	1	6.67	1.91	2	13.33	2.87	1	6.67	1.88	1	1	1	1
GO:0016070	1	1	100	1.13	0	0	0	0	0	0	0	0	0	1	1	1	1
GO:0016071	5	5	100	1.13	0	0	0	0	0	0	0	0	0	1	1	1	1
GO:0016079	4	3	75	0.85	1	25	7.16	0	0	0	0	0	0	1	1	1	1
GO:0016080	3	3	100	1.13	0	0	0	0	0	0	0	0	0	1	1	1	1
GO:0016081	7	5	71.43	0.81	1	14.29	4.09	1	14.29	3.08	0	0	0	1	1	1	1
GO:0016083	1	1	100	1.13	0	0	0	0	0	0	0	0	0	1	1	1	1
GO:0016126	8	8	100	1.13	0	0	0	0	0	0	0	0	0	1	1	1	1
GO:0016153	1	1	100	1.13	0	0	0	0	0	0	0	0	0	1	1	1	1
GO:0016155	3	3	100	1.13	0	0	0	0	0	0	0	0	0	1	1	1	1
GO:0016165	7	7	100	1.13	0	0	0	0	0	0	0	0	0	1	1	1	1
GO:0016171	11	9	81.82	0.93	2	18.18	5.21	0	0	0	0	0	0	1	1	1	1
GO:0016172	1	1	100	1.13	0	0	0	0	0	0	0	0	0	1	1	1	1
GO:0016175	9	7	77.78	0.88	0	0	0	2	22.22	4.78	0	0	0	1	1	1	1
GO:0016176	1	1	100	1.13	0	0	0	0	0	0	0	0	0	1	1	1	1
GO:0016181	2	2	100	1.13	0	0	0	0	0	0	0	0	0	1	1	1	1
GO:0016183	4	4	100	1.13	0	0	0	0	0	0	0	0	0	1	1	1	1
GO:0016192	64	62	96.88	1.1	0	0	0	2	3.13	0.67	0	0	0	1	1	1	1
GO:0016196	6	6	100	1.13	0	0	0	0	0	0	0	0	0	1	1	1	1
GO:0016197	16	16	100	1.13	0	0	0	0	0	0	0	0	0	1	1	1	1

GO:0016202	1	1	100	1.13	0	0	0	0	0	0	0	0	0	1	1	1	1
GO:0016205	3	3	100	1.13	0	0	0	0	0	0	0	0	0	1	1	1	1
GO:0016206	3	3	100	1.13	0	0	0	0	0	0	0	0	0	1	1	1	1
GO:0016209	7	7	100	1.13	0	0	0	0	0	0	0	0	0	1	1	1	1
GO:0016211	2	1	50	0.57	0	0	0	1	50	10.76	0	0	0	1	1	1	1
GO:0016222	1	1	100	1.13	0	0	0	0	0	0	0	0	0	1	1	1	1
GO:0016228	7	7	100	1.13	0	0	0	0	0	0	0	0	0	1	1	1	1
GO:0016229	5	1	20	0.23	1	20	5.73	2	40	8.61	1	20	5.64	1	1	1	1
GO:0016232	1	1	100	1.13	0	0	0	0	0	0	0	0	0	1	1	1	1
GO:0016233	1	1	100	1.13	0	0	0	0	0	0	0	0	0	1	1	1	1
GO:0016250	1	1	100	1.13	0	0	0	0	0	0	0	0	0	1	1	1	1
GO:0016251	15	14	93.33	1.06	1	6.67	1.91	0	0	0	0	0	0	1	1	1	1
GO:0016252	2	2	100	1.13	0	0	0	0	0	0	0	0	0	1	1	1	1
GO:0016253	4	4	100	1.13	0	0	0	0	0	0	0	0	0	1	1	1	1
GO:0016272	5	5	100	1.13	0	0	0	0	0	0	0	0	0	1	1	1	1
GO:0016273	3	3	100	1.13	0	0	0	0	0	0	0	0	0	1	1	1	1
GO:0016274	7	7	100	1.13	0	0	0	0	0	0	0	0	0	1	1	1	1
GO:0016278	3	3	100	1.13	0	0	0	0	0	0	0	0	0	1	1	1	1
GO:0016279	3	3	100	1.13	0	0	0	0	0	0	0	0	0	1	1	1	1
GO:0016280	1	1	100	1.13	0	0	0	0	0	0	0	0	0	1	1	1	1
GO:0016281	4	4	100	1.13	0	0	0	0	0	0	0	0	0	1	1	1	1
GO:0016286	4	4	100	1.13	0	0	0	0	0	0	0	0	0	1	1	1	1
GO:0016287	4	4	100	1.13	0	0	0	0	0	0	0	0	0	1	1	1	1
GO:0016288	10	9	90	1.02	1	10	2.86	0	0	0	0	0	0	1	1	1	1
GO:0016289	3	3	100	1.13	0	0	0	0	0	0	0	0	0	1	1	1	1
GO:0016290	4	3	75	0.85	0	0	0	1	25	5.38	0	0	0	1	1	1	1
GO:0016291	3	3	100	1.13	0	0	0	0	0	0	0	0	0	1	1	1	1
GO:0016292	1	1	100	1.13	0	0	0	0	0	0	0	0	0	1	1	1	1
GO:0016293	2	2	100	1.13	0	0	0	0	0	0	0	0	0	1	1	1	1
GO:0016298	3	2	66.67	0.75	0	0	0	0	0	0	1	33.33	9.41	1	1	1	1
GO:0016300	3	3	100	1.13	0	0	0	0	0	0	0	0	0	1	1	1	1
GO:0016301	455	433	95.16	1.08	8	1.76	0.5	7	1.54	0.33	7	1.54	0.43	6.82E-05	1	1	1
GO:0016302	1	1	100	1.13	0	0	0	0	0	0	0	0	0	1	1	1	1
GO:0016303	16	15	93.75	1.06	1	6.25	1.79	0	0	0	0	0	0	1	1	1	1
GO:0016304	1	0	0	0	1	100	28.63	0	0	0	0	0	0	1	1	1	1
GO:0016305	3	2	66.67	0.75	1	33.33	9.54	0	0	0	0	0	0	1	1	1	1
GO:0016306	2	1	50	0.57	1	50	14.32	0	0	0	0	0	0	1	1	1	1
GO:0016308	12	12	100	1.13	0	0	0	0	0	0	0	0	0	1	1	1	1
GO:0016310	9	9	100	1.13	0	0	0	0	0	0	0	0	0	1	1	1	1
GO:0016311	2	2	100	1.13	0	0	0	0	0	0	0	0	0	1	1	1	1
GO:0016322	3	3	100	1.13	0	0	0	0	0	0	0	0	0	1	1	1	1

GO:0016323	5	5	100	1.13	0	0	0	0	0	0	0	0	0	1	1	1	1
GO:0016324	9	9	100	1.13	0	0	0	0	0	0	0	0	0	1	1	1	1
GO:0016329	30	29	96.67	1.09	0	0	0	0	0	0	1	3.33	0.94	1	1	1	1
GO:0016331	2	2	100	1.13	0	0	0	0	0	0	0	0	0	1	1	1	1
GO:0016337	27	24	88.89	1.01	2	7.41	2.12	1	3.7	0.8	0	0	0	1	1	1	1
GO:0016338	1	1	100	1.13	0	0	0	0	0	0	0	0	0	1	1	1	1
GO:0016339	4	2	50	0.57	1	25	7.16	1	25	5.38	0	0	0	1	1	1	1
GO:0016343	1	1	100	1.13	0	0	0	0	0	0	0	0	0	1	1	1	1
GO:0016347	1	1	100	1.13	0	0	0	0	0	0	0	0	0	1	1	1	1
GO:0016358	2	2	100	1.13	0	0	0	0	0	0	0	0	0	1	1	1	1
GO:0016363	10	9	90	1.02	1	10	2.86	0	0	0	0	0	0	1	1	1	1
GO:0016401	2	2	100	1.13	0	0	0	0	0	0	0	0	0	1	1	1	1
GO:0016402	3	3	100	1.13	0	0	0	0	0	0	0	0	0	1	1	1	1
GO:0016403	2	2	100	1.13	0	0	0	0	0	0	0	0	0	1	1	1	1
GO:0016404	1	1	100	1.13	0	0	0	0	0	0	0	0	0	1	1	1	1
GO:0016407	1	0	0	0	0	0	0	1	100	21.53	0	0	0	1	1	1	1
GO:0016410	1	0	0	0	0	0	0	0	0	0	1	100	28.22	1	1	1	1
GO:0016423	3	3	100	1.13	0	0	0	0	0	0	0	0	0	1	1	1	1
GO:0016424	3	3	100	1.13	0	0	0	0	0	0	0	0	0	1	1	1	1
GO:0016426	3	3	100	1.13	0	0	0	0	0	0	0	0	0	1	1	1	1
GO:0016427	3	3	100	1.13	0	0	0	0	0	0	0	0	0	1	1	1	1
GO:0016433	3	3	100	1.13	0	0	0	0	0	0	0	0	0	1	1	1	1
GO:0016434	3	3	100	1.13	0	0	0	0	0	0	0	0	0	1	1	1	1
GO:0016435	3	3	100	1.13	0	0	0	0	0	0	0	0	0	1	1	1	1
GO:0016436	3	3	100	1.13	0	0	0	0	0	0	0	0	0	1	1	1	1
GO:0016444	1	1	100	1.13	0	0	0	0	0	0	0	0	0	1	1	1	1
GO:0016448	2	2	100	1.13	0	0	0	0	0	0	0	0	0	1	1	1	1
GO:0016449	2	2	100	1.13	0	0	0	0	0	0	0	0	0	1	1	1	1
GO:0016450	3	3	100	1.13	0	0	0	0	0	0	0	0	0	1	1	1	1
GO:0016451	2	2	100	1.13	0	0	0	0	0	0	0	0	0	1	1	1	1
GO:0016452	2	2	100	1.13	0	0	0	0	0	0	0	0	0	1	1	1	1
GO:0016455	1	1	100	1.13	0	0	0	0	0	0	0	0	0	1	1	1	1
GO:0016459	40	40	100	1.13	0	0	0	0	0	0	0	0	0	1	1	1	1
GO:0016461	1	1	100	1.13	0	0	0	0	0	0	0	0	0	1	1	1	1
GO:0016462	10	10	100	1.13	0	0	0	0	0	0	0	0	0	1	1	1	1
GO:0016467	7	7	100	1.13	0	0	0	0	0	0	0	0	0	1	1	1	1
GO:0016469	79	75	94.94	1.07	1	1.27	0.36	3	3.8	0.82	0	0	0	1	1	1	1
GO:0016471	7	5	71.43	0.81	0	0	0	2	28.57	6.15	0	0	0	1	1	1	1
GO:0016477	15	15	100	1.13	0	0	0	0	0	0	0	0	0	1	1	1	1
GO:0016481	26	25	96.15	1.09	0	0	0	1	3.85	0.83	0	0	0	1	1	1	1
GO:0016485	5	5	100	1.13	0	0	0	0	0	0	0	0	0	1	1	1	1

GO:0016486	6	5	83.33	0.94	1	16.67	4.77	0	0	0	0	0	0	1	1	1	1
GO:0016491	509	439	86.25	0.98	14	2.75	0.79	33	6.48	1.4	23	4.52	1.28	1	1	1	1
GO:0016493	11	2	18.18	0.21	2	18.18	5.21	4	36.36	7.83	3	27.27	7.7	1	1	1	1
GO:0016494	4	1	25	0.28	1	25	7.16	1	25	5.38	1	25	7.06	1	1	1	1
GO:0016505	5	5	100	1.13	0	0	0	0	0	0	0	0	0	1	1	1	1
GO:0016506	3	3	100	1.13	0	0	0	0	0	0	0	0	0	1	1	1	1
GO:0016508	5	5	100	1.13	0	0	0	0	0	0	0	0	0	1	1	1	1
GO:0016509	3	3	100	1.13	0	0	0	0	0	0	0	0	0	1	1	1	1
GO:0016510	5	5	100	1.13	0	0	0	0	0	0	0	0	0	1	1	1	1
GO:0016511	2	2	100	1.13	0	0	0	0	0	0	0	0	0	1	1	1	1
GO:0016512	1	1	100	1.13	0	0	0	0	0	0	0	0	0	1	1	1	1
GO:0016514	1	1	100	1.13	0	0	0	0	0	0	0	0	0	1	1	1	1
GO:0016524	4	4	100	1.13	0	0	0	0	0	0	0	0	0	1	1	1	1
GO:0016525	3	2	66.67	0.75	0	0	0	0	0	0	1	33.33	9.41	1	1	1	1
GO:0016526	1	1	100	1.13	0	0	0	0	0	0	0	0	0	1	1	1	1
GO:0016527	1	1	100	1.13	0	0	0	0	0	0	0	0	0	1	1	1	1
GO:0016529	3	2	66.67	0.75	0	0	0	0	0	0	1	33.33	9.41	1	1	1	1
GO:0016531	3	3	100	1.13	0	0	0	0	0	0	0	0	0	1	1	1	1
GO:0016534	4	4	100	1.13	0	0	0	0	0	0	0	0	0	1	1	1	1
GO:0016537	17	17	100	1.13	0	0	0	0	0	0	0	0	0	1	1	1	1
GO:0016538	35	35	100	1.13	0	0	0	0	0	0	0	0	0	1	1	1	1
GO:0016539	1	1	100	1.13	0	0	0	0	0	0	0	0	0	1	1	1	1
GO:0016554	3	2	66.67	0.75	1	33.33	9.54	0	0	0	0	0	0	1	1	1	1
GO:0016556	1	1	100	1.13	0	0	0	0	0	0	0	0	0	1	1	1	1
GO:0016563	57	49	85.96	0.97	1	1.75	0.5	3	5.26	1.13	4	7.02	1.98	1	1	1	1
GO:0016564	50	49	98	1.11	1	2	0.57	0	0	0	0	0	0	1	1	1	1
GO:0016565	1	1	100	1.13	0	0	0	0	0	0	0	0	0	1	1	1	1
GO:0016566	1	1	100	1.13	0	0	0	0	0	0	0	0	0	1	1	1	1
GO:0016567	12	12	100	1.13	0	0	0	0	0	0	0	0	0	1	1	1	1
GO:0016568	22	22	100	1.13	0	0	0	0	0	0	0	0	0	1	1	1	1
GO:0016571	1	1	100	1.13	0	0	0	0	0	0	0	0	0	1	1	1	1
GO:0016573	1	0	0	0	0	0	0	1	100	21.53	0	0	0	1	1	1	1
GO:0016575	4	4	100	1.13	0	0	0	0	0	0	0	0	0	1	1	1	1
GO:0016579	4	4	100	1.13	0	0	0	0	0	0	0	0	0	1	1	1	1
GO:0016581	12	12	100	1.13	0	0	0	0	0	0	0	0	0	1	1	1	1
GO:0016584	2	2	100	1.13	0	0	0	0	0	0	0	0	0	1	1	1	1
GO:0016585	7	7	100	1.13	0	0	0	0	0	0	0	0	0	1	1	1	1
GO:0016597	3	3	100	1.13	0	0	0	0	0	0	0	0	0	1	1	1	1
GO:0016598	3	3	100	1.13	0	0	0	0	0	0	0	0	0	1	1	1	1
GO:0016599	3	3	100	1.13	0	0	0	0	0	0	0	0	0	1	1	1	1
GO:0016600	4	4	100	1.13	0	0	0	0	0	0	0	0	0	1	1	1	1

GO:0016601	12	12	100	1.13	0	0	0	0	0	0	0	0	0	1	1	1	1
GO:0016603	6	3	50	0.57	0	0	0	3	50	10.76	0	0	0	1	1	1	1
GO:0016604	4	4	100	1.13	0	0	0	0	0	0	0	0	0	1	1	1	1
GO:0016605	1	1	100	1.13	0	0	0	0	0	0	0	0	0	1	1	1	1
GO:0016614	5	4	80	0.91	0	0	0	1	20	4.31	0	0	0	1	1	1	1
GO:0016615	8	8	100	1.13	0	0	0	0	0	0	0	0	0	1	1	1	1
GO:0016616	14	9	64.29	0.73	1	7.14	2.05	3	21.43	4.61	1	7.14	2.02	1	1	1	1
GO:0016619	2	2	100	1.13	0	0	0	0	0	0	0	0	0	1	1	1	1
GO:0016620	7	7	100	1.13	0	0	0	0	0	0	0	0	0	1	1	1	1
GO:0016624	8	6	75	0.85	0	0	0	2	25	5.38	0	0	0	1	1	1	1
GO:0016638	3	1	33.33	0.38	0	0	0	0	0	0	2	66.67	18.82	1	1	1	1
GO:0016641	3	3	100	1.13	0	0	0	0	0	0	0	0	0	1	1	1	1
GO:0016651	1	1	100	1.13	0	0	0	0	0	0	0	0	0	1	1	1	1
GO:0016654	3	3	100	1.13	0	0	0	0	0	0	0	0	0	1	1	1	1
GO:0016656	2	2	100	1.13	0	0	0	0	0	0	0	0	0	1	1	1	1
GO:0016684	4	4	100	1.13	0	0	0	0	0	0	0	0	0	1	1	1	1
GO:0016685	2	2	100	1.13	0	0	0	0	0	0	0	0	0	1	1	1	1
GO:0016686	2	2	100	1.13	0	0	0	0	0	0	0	0	0	1	1	1	1
GO:0016687	2	2	100	1.13	0	0	0	0	0	0	0	0	0	1	1	1	1
GO:0016702	23	21	91.3	1.03	0	0	0	1	4.35	0.94	1	4.35	1.23	1	1	1	1
GO:0016706	15	13	86.67	0.98	0	0	0	2	13.33	2.87	0	0	0	1	1	1	1
GO:0016712	8	0	0	0	1	12.5	3.58	2	25	5.38	5	62.5	17.64	1	1	1	0.016155125
GO:0016730	1	1	100	1.13	0	0	0	0	0	0	0	0	0	1	1	1	1
GO:0016740	874	812	92.91	1.05	16	1.83	0.52	31	3.55	0.76	15	1.72	0.48	0.000318885	1	1	1
GO:0016742	8	8	100	1.13	0	0	0	0	0	0	0	0	0	1	1	1	1
GO:0016743	1	0	0	0	0	0	0	1	100	21.53	0	0	0	1	1	1	1
GO:0016747	3	3	100	1.13	0	0	0	0	0	0	0	0	0	1	1	1	1
GO:0016757	71	67	94.37	1.07	1	1.41	0.4	1	1.41	0.3	2	2.82	0.8	1	1	1	1
GO:0016758	21	12	57.14	0.65	0	0	0	6	28.57	6.15	3	14.29	4.03	1	1	1	1
GO:0016759	4	4	100	1.13	0	0	0	0	0	0	0	0	0	1	1	1	1
GO:0016764	2	2	100	1.13	0	0	0	0	0	0	0	0	0	1	1	1	1
GO:0016766	1	1	100	1.13	0	0	0	0	0	0	0	0	0	1	1	1	1
GO:0016768	4	4	100	1.13	0	0	0	0	0	0	0	0	0	1	1	1	1
GO:0016772	8	8	100	1.13	0	0	0	0	0	0	0	0	0	1	1	1	1
GO:0016773	22	22	100	1.13	0	0	0	0	0	0	0	0	0	1	1	1	1
GO:0016776	5	5	100	1.13	0	0	0	0	0	0	0	0	0	1	1	1	1
GO:0016779	48	43	89.58	1.01	2	4.17	1.19	2	4.17	0.9	1	2.08	0.59	1	1	1	1
GO:0016780	2	2	100	1.13	0	0	0	0	0	0	0	0	0	1	1	1	1
GO:0016787	859	759	88.36	1	24	2.79	0.8	46	5.36	1.15	30	3.49	0.99	1	1	1	1
GO:0016788	12	12	100	1.13	0	0	0	0	0	0	0	0	0	1	1	1	1
GO:0016789	11	5	45.45	0.51	0	0	0	2	18.18	3.91	4	36.36	10.26	1	1	1	1

GO:0016790	1	1	100	1.13	0	0	0	0	0	0	0	0	0	1	1	1	1
GO:0016791	2	1	50	0.57	0	0	0	0	0	0	1	50	14.11	1	1	1	1
GO:0016798	51	43	84.31	0.95	2	3.92	1.12	2	3.92	0.84	4	7.84	2.21	1	1	1	1
GO:0016804	4	4	100	1.13	0	0	0	0	0	0	0	0	0	1	1	1	1
GO:0016805	8	7	87.5	0.99	0	0	0	0	0	0	1	12.5	3.53	1	1	1	1
GO:0016810	7	6	85.71	0.97	0	0	0	1	14.29	3.08	0	0	0	1	1	1	1
GO:0016811	2	1	50	0.57	0	0	0	1	50	10.76	0	0	0	1	1	1	1
GO:0016814	4	4	100	1.13	0	0	0	0	0	0	0	0	0	1	1	1	1
GO:0016818	7	7	100	1.13	0	0	0	0	0	0	0	0	0	1	1	1	1
GO:0016820	36	33	91.67	1.04	1	2.78	0.8	2	5.56	1.2	0	0	0	1	1	1	1
GO:0016829	97	93	95.88	1.09	4	4.12	1.18	0	0	0	0	0	0	1	1	1	1
GO:0016831	26	25	96.15	1.09	1	3.85	1.1	0	0	0	0	0	0	1	1	1	1
GO:0016836	6	6	100	1.13	0	0	0	0	0	0	0	0	0	1	1	1	1
GO:0016844	2	2	100	1.13	0	0	0	0	0	0	0	0	0	1	1	1	1
GO:0016849	2	0	0	0	0	0	0	2	100	21.53	0	0	0	1	1	1	1
GO:0016851	3	3	100	1.13	0	0	0	0	0	0	0	0	0	1	1	1	1
GO:0016852	3	3	100	1.13	0	0	0	0	0	0	0	0	0	1	1	1	1
GO:0016853	93	87	93.55	1.06	1	1.08	0.31	2	2.15	0.46	3	3.23	0.91	1	1	1	1
GO:0016855	3	3	100	1.13	0	0	0	0	0	0	0	0	0	1	1	1	1
GO:0016857	1	1	100	1.13	0	0	0	0	0	0	0	0	0	1	1	1	1
GO:0016868	11	10	90.91	1.03	0	0	0	1	9.09	1.96	0	0	0	1	1	1	1
GO:0016874	133	126	94.74	1.07	2	1.5	0.43	4	3.01	0.65	1	0.75	0.21	1	1	1	1
GO:0016881	1	1	100	1.13	0	0	0	0	0	0	0	0	0	1	1	1	1
GO:0016885	1	1	100	1.13	0	0	0	0	0	0	0	0	0	1	1	1	1
GO:0016887	16	15	93.75	1.06	0	0	0	0	0	0	1	6.25	1.76	1	1	1	1
GO:0016892	1	0	0	0	0	0	0	1	100	21.53	0	0	0	1	1	1	1
GO:0016893	1	1	100	1.13	0	0	0	0	0	0	0	0	0	1	1	1	1
GO:0016908	17	17	100	1.13	0	0	0	0	0	0	0	0	0	1	1	1	1
GO:0016909	17	17	100	1.13	0	0	0	0	0	0	0	0	0	1	1	1	1
GO:0016910	17	17	100	1.13	0	0	0	0	0	0	0	0	0	1	1	1	1
GO:0016911	17	17	100	1.13	0	0	0	0	0	0	0	0	0	1	1	1	1
GO:0016912	17	17	100	1.13	0	0	0	0	0	0	0	0	0	1	1	1	1
GO:0016919	1	1	100	1.13	0	0	0	0	0	0	0	0	0	1	1	1	1
GO:0016922	5	5	100	1.13	0	0	0	0	0	0	0	0	0	1	1	1	1
GO:0016923	7	4	57.14	0.65	0	0	0	3	42.86	9.23	0	0	0	1	1	1	1
GO:0016925	2	2	100	1.13	0	0	0	0	0	0	0	0	0	1	1	1	1
GO:0016932	26	26	100	1.13	0	0	0	0	0	0	0	0	0	1	1	1	1
GO:0016937	3	3	100	1.13	0	0	0	0	0	0	0	0	0	1	1	1	1
GO:0016946	3	3	100	1.13	0	0	0	0	0	0	0	0	0	1	1	1	1
GO:0016954	1	1	100	1.13	0	0	0	0	0	0	0	0	0	1	1	1	1
GO:0016962	3	3	100	1.13	0	0	0	0	0	0	0	0	0	1	1	1	1

GO:0016971	2	2	100	1.13	0	0	0	0	0	0	0	0	0	1	1	1	1
GO:0016978	8	8	100	1.13	0	0	0	0	0	0	0	0	0	1	1	1	1
GO:0016986	1	0	0	0	0	0	0	1	100	21.53	0	0	0	1	1	1	1
GO:0016987	1	1	100	1.13	0	0	0	0	0	0	0	0	0	1	1	1	1
GO:0016992	3	3	100	1.13	0	0	0	0	0	0	0	0	0	1	1	1	1
GO:0016998	4	4	100	1.13	0	0	0	0	0	0	0	0	0	1	1	1	1
GO:0017014	1	1	100	1.13	0	0	0	0	0	0	0	0	0	1	1	1	1
GO:0017015	1	1	100	1.13	0	0	0	0	0	0	0	0	0	1	1	1	1
GO:0017017	15	15	100	1.13	0	0	0	0	0	0	0	0	0	1	1	1	1
GO:0017018	13	12	92.31	1.05	1	7.69	2.2	0	0	0	0	0	0	1	1	1	1
GO:0017022	1	0	0	0	1	100	28.63	0	0	0	0	0	0	1	1	1	1
GO:0017025	8	8	100	1.13	0	0	0	0	0	0	0	0	0	1	1	1	1
GO:0017039	5	5	100	1.13	0	0	0	0	0	0	0	0	0	1	1	1	1
GO:0017040	2	2	100	1.13	0	0	0	0	0	0	0	0	0	1	1	1	1
GO:0017050	3	2	66.67	0.75	1	33.33	9.54	0	0	0	0	0	0	1	1	1	1
GO:0017053	6	6	100	1.13	0	0	0	0	0	0	0	0	0	1	1	1	1
GO:0017057	7	5	71.43	0.81	0	0	0	2	28.57	6.15	0	0	0	1	1	1	1
GO:0017061	2	2	100	1.13	0	0	0	0	0	0	0	0	0	1	1	1	1
GO:0017068	1	1	100	1.13	0	0	0	0	0	0	0	0	0	1	1	1	1
GO:0017070	4	4	100	1.13	0	0	0	0	0	0	0	0	0	1	1	1	1
GO:0017072	4	4	100	1.13	0	0	0	0	0	0	0	0	0	1	1	1	1
GO:0017075	2	1	50	0.57	0	0	0	1	50	10.76	0	0	0	1	1	1	1
GO:0017083	4	4	100	1.13	0	0	0	0	0	0	0	0	0	1	1	1	1
GO:0017095	1	1	100	1.13	0	0	0	0	0	0	0	0	0	1	1	1	1
GO:0017103	1	1	100	1.13	0	0	0	0	0	0	0	0	0	1	1	1	1
GO:0017110	11	11	100	1.13	0	0	0	0	0	0	0	0	0	1	1	1	1
GO:0017113	1	1	100	1.13	0	0	0	0	0	0	0	0	0	1	1	1	1
GO:0017121	2	2	100	1.13	0	0	0	0	0	0	0	0	0	1	1	1	1
GO:0017124	14	13	92.86	1.05	0	0	0	1	7.14	1.54	0	0	0	1	1	1	1
GO:0017127	4	3	75	0.85	0	0	0	1	25	5.38	0	0	0	1	1	1	1
GO:0017128	2	2	100	1.13	0	0	0	0	0	0	0	0	0	1	1	1	1
GO:0017129	2	1	50	0.57	0	0	0	1	50	10.76	0	0	0	1	1	1	1
GO:0017133	3	3	100	1.13	0	0	0	0	0	0	0	0	0	1	1	1	1
GO:0017136	2	2	100	1.13	0	0	0	0	0	0	0	0	0	1	1	1	1
GO:0017139	1	1	100	1.13	0	0	0	0	0	0	0	0	0	1	1	1	1
GO:0017140	3	3	100	1.13	0	0	0	0	0	0	0	0	0	1	1	1	1
GO:0017148	2	1	50	0.57	1	50	14.32	0	0	0	0	0	0	1	1	1	1
GO:0017153	6	5	83.33	0.94	0	0	0	0	0	0	1	16.67	4.7	1	1	1	1
GO:0017154	3	1	33.33	0.38	2	66.67	19.09	0	0	0	0	0	0	1	1	1	1
GO:0017156	1	1	100	1.13	0	0	0	0	0	0	0	0	0	1	1	1	1
GO:0017157	3	3	100	1.13	0	0	0	0	0	0	0	0	0	1	1	1	1

GO:0017158	1	0	0	0	1	100	28.63	0	0	0	0	0	0	1	1	1	1
GO:0017159	2	1	50	0.57	0	0	0	1	50	10.76	0	0	0	1	1	1	1
GO:0017175	1	1	100	1.13	0	0	0	0	0	0	0	0	0	1	1	1	1
GO:0017177	4	4	100	1.13	0	0	0	0	0	0	0	0	0	1	1	1	1
GO:0018008	4	4	100	1.13	0	0	0	0	0	0	0	0	0	1	1	1	1
GO:0018105	1	0	0	0	1	100	28.63	0	0	0	0	0	0	1	1	1	1
GO:0018107	1	0	0	0	1	100	28.63	0	0	0	0	0	0	1	1	1	1
GO:0018108	9	8	88.89	1.01	1	11.11	3.18	0	0	0	0	0	0	1	1	1	1
GO:0018149	9	6	66.67	0.75	2	22.22	6.36	0	0	0	1	11.11	3.14	1	1	1	1
GO:0018169	1	1	100	1.13	0	0	0	0	0	0	0	0	0	1	1	1	1
GO:0018184	1	1	100	1.13	0	0	0	0	0	0	0	0	0	1	1	1	1
GO:0018223	1	1	100	1.13	0	0	0	0	0	0	0	0	0	1	1	1	1
GO:0018224	1	1	100	1.13	0	0	0	0	0	0	0	0	0	1	1	1	1
GO:0018279	1	1	100	1.13	0	0	0	0	0	0	0	0	0	1	1	1	1
GO:0018346	8	8	100	1.13	0	0	0	0	0	0	0	0	0	1	1	1	1
GO:0018347	2	2	100	1.13	0	0	0	0	0	0	0	0	0	1	1	1	1
GO:0018392	4	4	100	1.13	0	0	0	0	0	0	0	0	0	1	1	1	1
GO:0018401	12	12	100	1.13	0	0	0	0	0	0	0	0	0	1	1	1	1
GO:0018421	2	2	100	1.13	0	0	0	0	0	0	0	0	0	1	1	1	1
GO:0018422	2	2	100	1.13	0	0	0	0	0	0	0	0	0	1	1	1	1
GO:0018423	3	3	100	1.13	0	0	0	0	0	0	0	0	0	1	1	1	1
GO:0018451	5	1	20	0.23	1	20	5.73	2	40	8.61	1	20	5.64	1	1	1	1
GO:0018452	5	1	20	0.23	1	20	5.73	2	40	8.61	1	20	5.64	1	1	1	1
GO:0018453	5	1	20	0.23	1	20	5.73	2	40	8.61	1	20	5.64	1	1	1	1
GO:0018707	3	3	100	1.13	0	0	0	0	0	0	0	0	0	1	1	1	1
GO:0018715	4	4	100	1.13	0	0	0	0	0	0	0	0	0	1	1	1	1
GO:0018716	4	4	100	1.13	0	0	0	0	0	0	0	0	0	1	1	1	1
GO:0018717	4	4	100	1.13	0	0	0	0	0	0	0	0	0	1	1	1	1
GO:0018718	4	4	100	1.13	0	0	0	0	0	0	0	0	0	1	1	1	1
GO:0018720	17	17	100	1.13	0	0	0	0	0	0	0	0	0	1	1	1	1
GO:0018721	1	1	100	1.13	0	0	0	0	0	0	0	0	0	1	1	1	1
GO:0018722	1	1	100	1.13	0	0	0	0	0	0	0	0	0	1	1	1	1
GO:0018723	1	1	100	1.13	0	0	0	0	0	0	0	0	0	1	1	1	1
GO:0018724	1	1	100	1.13	0	0	0	0	0	0	0	0	0	1	1	1	1
GO:0018725	1	1	100	1.13	0	0	0	0	0	0	0	0	0	1	1	1	1
GO:0018726	1	1	100	1.13	0	0	0	0	0	0	0	0	0	1	1	1	1
GO:0018727	1	1	100	1.13	0	0	0	0	0	0	0	0	0	1	1	1	1
GO:0018731	2	2	100	1.13	0	0	0	0	0	0	0	0	0	1	1	1	1
GO:0018732	2	2	100	1.13	0	0	0	0	0	0	0	0	0	1	1	1	1
GO:0018733	2	2	100	1.13	0	0	0	0	0	0	0	0	0	1	1	1	1
GO:0018734	2	2	100	1.13	0	0	0	0	0	0	0	0	0	1	1	1	1

GO:0018736	1	1	100	1.13	0	0	0	0	0	0	0	0	0	1	1	1	1
GO:0018737	1	1	100	1.13	0	0	0	0	0	0	0	0	0	1	1	1	1
GO:0018748	2	1	50	0.57	0	0	0	1	50	10.76	0	0	0	1	1	1	1
GO:0018749	2	1	50	0.57	0	0	0	1	50	10.76	0	0	0	1	1	1	1
GO:0018874	1	0	0	0	0	0	0	1	100	21.53	0	0	0	1	1	1	1
GO:0019001	5	5	100	1.13	0	0	0	0	0	0	0	0	0	1	1	1	1
GO:0019004	1	1	100	1.13	0	0	0	0	0	0	0	0	0	1	1	1	1
GO:0019005	4	3	75	0.85	0	0	0	0	0	0	1	25	7.06	1	1	1	1
GO:0019007	2	2	100	1.13	0	0	0	0	0	0	0	0	0	1	1	1	1
GO:0019013	38	36	94.74	1.07	0	0	0	2	5.26	1.13	0	0	0	1	1	1	1
GO:0019028	6	6	100	1.13	0	0	0	0	0	0	0	0	0	1	1	1	1
GO:0019047	2	2	100	1.13	0	0	0	0	0	0	0	0	0	1	1	1	1
GO:0019048	1	1	100	1.13	0	0	0	0	0	0	0	0	0	1	1	1	1
GO:0019069	5	5	100	1.13	0	0	0	0	0	0	0	0	0	1	1	1	1
GO:0019103	1	1	100	1.13	0	0	0	0	0	0	0	0	0	1	1	1	1
GO:0019104	3	2	66.67	0.75	1	33.33	9.54	0	0	0	0	0	0	1	1	1	1
GO:0019111	1	1	100	1.13	0	0	0	0	0	0	0	0	0	1	1	1	1
GO:0019112	4	4	100	1.13	0	0	0	0	0	0	0	0	0	1	1	1	1
GO:0019143	1	1	100	1.13	0	0	0	0	0	0	0	0	0	1	1	1	1
GO:0019166	1	1	100	1.13	0	0	0	0	0	0	0	0	0	1	1	1	1
GO:0019176	1	1	100	1.13	0	0	0	0	0	0	0	0	0	1	1	1	1
GO:0019177	1	1	100	1.13	0	0	0	0	0	0	0	0	0	1	1	1	1
GO:0019187	4	4	100	1.13	0	0	0	0	0	0	0	0	0	1	1	1	1
GO:0019199	17	17	100	1.13	0	0	0	0	0	0	0	0	0	1	1	1	1
GO:0019208	1	1	100	1.13	0	0	0	0	0	0	0	0	0	1	1	1	1
GO:0019210	1	1	100	1.13	0	0	0	0	0	0	0	0	0	1	1	1	1
GO:0019211	4	4	100	1.13	0	0	0	0	0	0	0	0	0	1	1	1	1
GO:0019212	2	2	100	1.13	0	0	0	0	0	0	0	0	0	1	1	1	1
GO:0019217	4	4	100	1.13	0	0	0	0	0	0	0	0	0	1	1	1	1
GO:0019221	10	10	100	1.13	0	0	0	0	0	0	0	0	0	1	1	1	1
GO:0019222	7	7	100	1.13	0	0	0	0	0	0	0	0	0	1	1	1	1
GO:0019229	2	2	100	1.13	0	0	0	0	0	0	0	0	0	1	1	1	1
GO:0019233	6	3	50	0.57	2	33.33	9.54	0	0	0	1	16.67	4.7	1	1	1	1
GO:0019239	1	0	0	0	0	0	0	0	0	0	1	100	28.22	1	1	1	1
GO:0019255	2	2	100	1.13	0	0	0	0	0	0	0	0	0	1	1	1	1
GO:0019276	1	1	100	1.13	0	0	0	0	0	0	0	0	0	1	1	1	1
GO:0019307	2	2	100	1.13	0	0	0	0	0	0	0	0	0	1	1	1	1
GO:0019363	3	3	100	1.13	0	0	0	0	0	0	0	0	0	1	1	1	1
GO:0019370	6	6	100	1.13	0	0	0	0	0	0	0	0	0	1	1	1	1
GO:0019395	1	0	0	0	0	0	0	1	100	21.53	0	0	0	1	1	1	1
GO:0019430	1	1	100	1.13	0	0	0	0	0	0	0	0	0	1	1	1	1

GO:0019432	1	1	100	1.13	0	0	0	0	0	0	0	0	0	1	1	1	1
GO:0019433	1	1	100	1.13	0	0	0	0	0	0	0	0	0	1	1	1	1
GO:0019478	2	2	100	1.13	0	0	0	0	0	0	0	0	0	1	1	1	1
GO:0019531	1	0	0	0	0	0	0	1	100	21.53	0	0	0	1	1	1	1
GO:0019532	1	0	0	0	0	0	0	1	100	21.53	0	0	0	1	1	1	1
GO:0019538	19	17	89.47	1.01	0	0	0	2	10.53	2.27	0	0	0	1	1	1	1
GO:0019605	1	0	0	0	0	0	0	1	100	21.53	0	0	0	1	1	1	1
GO:0019702	3	3	100	1.13	0	0	0	0	0	0	0	0	0	1	1	1	1
GO:0019717	19	18	94.74	1.07	0	0	0	0	0	0	1	5.26	1.49	1	1	1	1
GO:0019722	4	4	100	1.13	0	0	0	0	0	0	0	0	0	1	1	1	1
GO:0019733	1	0	0	0	0	0	0	1	100	21.53	0	0	0	1	1	1	1
GO:0019752	1	1	100	1.13	0	0	0	0	0	0	0	0	0	1	1	1	1
GO:0019770	3	0	0	0	0	0	0	0	0	0	3	100	28.22	1	1	1	0.21002992
GO:0019781	4	4	100	1.13	0	0	0	0	0	0	0	0	0	1	1	1	1
GO:0019789	2	2	100	1.13	0	0	0	0	0	0	0	0	0	1	1	1	1
GO:0019807	1	0	0	0	0	0	0	0	0	0	1	100	28.22	1	1	1	1
GO:0019834	7	7	100	1.13	0	0	0	0	0	0	0	0	0	1	1	1	1
GO:0019835	4	1	25	0.28	0	0	0	3	75	16.15	0	0	0	1	1	1	1
GO:0019838	11	9	81.82	0.93	1	9.09	2.6	0	0	0	1	9.09	2.57	1	1	1	1
GO:0019839	7	6	85.71	0.97	1	14.29	4.09	0	0	0	0	0	0	1	1	1	1
GO:0019843	5	5	100	1.13	0	0	0	0	0	0	0	0	0	1	1	1	1
GO:0019863	6	5	83.33	0.94	0	0	0	0	0	0	1	16.67	4.7	1	1	1	1
GO:0019864	9	3	33.33	0.38	0	0	0	3	33.33	7.18	3	33.33	9.41	1	1	1	1
GO:0019866	47	46	97.87	1.11	0	0	0	1	2.13	0.46	0	0	0	1	1	1	1
GO:0019867	26	26	100	1.13	0	0	0	0	0	0	0	0	0	1	1	1	1
GO:0019882	6	4	66.67	0.75	0	0	0	2	33.33	7.18	0	0	0	1	1	1	1
GO:0019883	7	7	100	1.13	0	0	0	0	0	0	0	0	0	1	1	1	1
GO:0019884	10	5	50	0.57	0	0	0	5	50	10.76	0	0	0	1	1	0.216534029	1
GO:0019885	8	8	100	1.13	0	0	0	0	0	0	0	0	0	1	1	1	1
GO:0019886	11	6	54.55	0.62	0	0	0	5	45.45	9.78	0	0	0	1	1	0.380704074	1
GO:0019897	7	6	85.71	0.97	0	0	0	0	0	0	1	14.29	4.03	1	1	1	1
GO:0019898	1	1	100	1.13	0	0	0	0	0	0	0	0	0	1	1	1	1
GO:0019899	2	2	100	1.13	0	0	0	0	0	0	0	0	0	1	1	1	1
GO:0019900	2	2	100	1.13	0	0	0	0	0	0	0	0	0	1	1	1	1
GO:0019901	2	1	50	0.57	0	0	0	0	0	0	1	50	14.11	1	1	1	1
GO:0019904	16	14	87.5	0.99	0	0	0	2	12.5	2.69	0	0	0	1	1	1	1
GO:0019905	6	6	100	1.13	0	0	0	0	0	0	0	0	0	1	1	1	1
GO:0019911	1	1	100	1.13	0	0	0	0	0	0	0	0	0	1	1	1	1
GO:0019912	17	17	100	1.13	0	0	0	0	0	0	0	0	0	1	1	1	1
GO:0019913	17	17	100	1.13	0	0	0	0	0	0	0	0	0	1	1	1	1
GO:0019914	17	17	100	1.13	0	0	0	0	0	0	0	0	0	1	1	1	1

GO:0019933	1	1	100	1.13	0	0	0	0	0	0	0	0	0	1	1	1	1
GO:0019942	2	2	100	1.13	0	0	0	0	0	0	0	0	0	1	1	1	1
GO:0019955	7	5	71.43	0.81	1	14.29	4.09	0	0	0	1	14.29	4.03	1	1	1	1
GO:0019966	1	1	100	1.13	0	0	0	0	0	0	0	0	0	1	1	1	1
GO:0019984	1	1	100	1.13	0	0	0	0	0	0	0	0	0	1	1	1	1
GO:0019986	1	1	100	1.13	0	0	0	0	0	0	0	0	0	1	1	1	1
GO:0019992	28	27	96.43	1.09	1	3.57	1.02	0	0	0	0	0	0	1	1	1	1
GO:0020037	143	137	95.8	1.08	3	2.1	0.6	3	2.1	0.45	0	0	0	1	1	1	1
GO:0030001	8	7	87.5	0.99	0	0	0	1	12.5	2.69	0	0	0	1	1	1	1
GO:0030016	1	0	0	0	0	0	0	1	100	21.53	0	0	0	1	1	1	1
GO:0030017	2	2	100	1.13	0	0	0	0	0	0	0	0	0	1	1	1	1
GO:0030018	6	6	100	1.13	0	0	0	0	0	0	0	0	0	1	1	1	1
GO:0030020	31	24	77.42	0.88	2	6.45	1.85	5	16.13	3.47	0	0	0	1	1	1	1
GO:0030021	1	0	0	0	0	0	0	0	0	0	1	100	28.22	1	1	1	1
GO:0030022	1	1	100	1.13	0	0	0	0	0	0	0	0	0	1	1	1	1
GO:0030023	2	1	50	0.57	0	0	0	1	50	10.76	0	0	0	1	1	1	1
GO:0030027	25	24	96	1.09	0	0	0	1	4	0.86	0	0	0	1	1	1	1
GO:0030029	1	0	0	0	0	0	0	1	100	21.53	0	0	0	1	1	1	1
GO:0030032	7	7	100	1.13	0	0	0	0	0	0	0	0	0	1	1	1	1
GO:0030033	2	2	100	1.13	0	0	0	0	0	0	0	0	0	1	1	1	1
GO:0030035	1	1	100	1.13	0	0	0	0	0	0	0	0	0	1	1	1	1
GO:0030036	27	26	96.3	1.09	0	0	0	1	3.7	0.8	0	0	0	1	1	1	1
GO:0030041	2	2	100	1.13	0	0	0	0	0	0	0	0	0	1	1	1	1
GO:0030048	7	6	85.71	0.97	0	0	0	1	14.29	3.08	0	0	0	1	1	1	1
GO:0030057	1	1	100	1.13	0	0	0	0	0	0	0	0	0	1	1	1	1
GO:0030060	5	5	100	1.13	0	0	0	0	0	0	0	0	0	1	1	1	1
GO:0030071	3	1	33.33	0.38	0	0	0	2	66.67	14.35	0	0	0	1	1	1	1
GO:0030073	4	1	25	0.28	0	0	0	2	50	10.76	1	25	7.06	1	1	1	1
GO:0030089	3	3	100	1.13	0	0	0	0	0	0	0	0	0	1	1	1	1
GO:0030097	16	14	87.5	0.99	1	6.25	1.79	0	0	0	1	6.25	1.76	1	1	1	1
GO:0030098	6	6	100	1.13	0	0	0	0	0	0	0	0	0	1	1	1	1
GO:0030099	3	2	66.67	0.75	0	0	0	0	0	0	1	33.33	9.41	1	1	1	1
GO:0030100	6	6	100	1.13	0	0	0	0	0	0	0	0	0	1	1	1	1
GO:0030101	1	0	0	0	1	100	28.63	0	0	0	0	0	0	1	1	1	1
GO:0030106	7	7	100	1.13	0	0	0	0	0	0	0	0	0	1	1	1	1
GO:0030111	5	3	60	0.68	0	0	0	1	20	4.31	1	20	5.64	1	1	1	1
GO:0030116	1	1	100	1.13	0	0	0	0	0	0	0	0	0	1	1	1	1
GO:0030117	3	3	100	1.13	0	0	0	0	0	0	0	0	0	1	1	1	1
GO:0030125	28	26	92.86	1.05	1	3.57	1.02	1	3.57	0.77	0	0	0	1	1	1	1
GO:0030127	7	7	100	1.13	0	0	0	0	0	0	0	0	0	1	1	1	1
GO:0030130	2	2	100	1.13	0	0	0	0	0	0	0	0	0	1	1	1	1

GO:0030131	2	2	100	1.13	0	0	0	0	0	0	0	0	0	1	1	1	1
GO:0030134	2	2	100	1.13	0	0	0	0	0	0	0	0	0	1	1	1	1
GO:0030136	2	2	100	1.13	0	0	0	0	0	0	0	0	0	1	1	1	1
GO:0030137	7	7	100	1.13	0	0	0	0	0	0	0	0	0	1	1	1	1
GO:0030139	18	18	100	1.13	0	0	0	0	0	0	0	0	0	1	1	1	1
GO:0030140	1	1	100	1.13	0	0	0	0	0	0	0	0	0	1	1	1	1
GO:0030141	19	17	89.47	1.01	0	0	0	2	10.53	2.27	0	0	0	1	1	1	1
GO:0030145	40	37	92.5	1.05	0	0	0	2	5	1.08	1	2.5	0.71	1	1	1	1
GO:0030148	3	3	100	1.13	0	0	0	0	0	0	0	0	0	1	1	1	1
GO:0030149	4	4	100	1.13	0	0	0	0	0	0	0	0	0	1	1	1	1
GO:0030151	7	2	28.57	0.32	1	14.29	4.09	2	28.57	6.15	2	28.57	8.06	1	1	1	1
GO:0030154	37	35	94.59	1.07	1	2.7	0.77	1	2.7	0.58	0	0	0	1	1	1	1
GO:0030155	6	6	100	1.13	0	0	0	0	0	0	0	0	0	1	1	1	1
GO:0030159	3	2	66.67	0.75	0	0	0	1	33.33	7.18	0	0	0	1	1	1	1
GO:0030161	4	4	100	1.13	0	0	0	0	0	0	0	0	0	1	1	1	1
GO:0030162	2	0	0	0	0	0	0	0	0	0	2	100	28.22	1	1	1	1
GO:0030163	37	37	100	1.13	0	0	0	0	0	0	0	0	0	1	1	1	1
GO:0030165	2	2	100	1.13	0	0	0	0	0	0	0	0	0	1	1	1	1
GO:0030166	1	1	100	1.13	0	0	0	0	0	0	0	0	0	1	1	1	1
GO:0030168	3	2	66.67	0.75	0	0	0	1	33.33	7.18	0	0	0	1	1	1	1
GO:0030170	3	3	100	1.13	0	0	0	0	0	0	0	0	0	1	1	1	1
GO:0030173	10	10	100	1.13	0	0	0	0	0	0	0	0	0	1	1	1	1
GO:0030175	1	1	100	1.13	0	0	0	0	0	0	0	0	0	1	1	1	1
GO:0030176	7	7	100	1.13	0	0	0	0	0	0	0	0	0	1	1	1	1
GO:0030178	2	2	100	1.13	0	0	0	0	0	0	0	0	0	1	1	1	1
GO:0030179	1	1	100	1.13	0	0	0	0	0	0	0	0	0	1	1	1	1
GO:0030182	13	13	100	1.13	0	0	0	0	0	0	0	0	0	1	1	1	1
GO:0030183	8	5	62.5	0.71	0	0	0	2	25	5.38	1	12.5	3.53	1	1	1	1
GO:0030188	2	2	100	1.13	0	0	0	0	0	0	0	0	0	1	1	1	1
GO:0030192	1	1	100	1.13	0	0	0	0	0	0	0	0	0	1	1	1	1
GO:0030195	1	0	0	0	0	0	0	0	0	0	1	100	28.22	1	1	1	1
GO:0030198	3	2	66.67	0.75	0	0	0	0	0	0	1	33.33	9.41	1	1	1	1
GO:0030199	1	1	100	1.13	0	0	0	0	0	0	0	0	0	1	1	1	1
GO:0030201	1	1	100	1.13	0	0	0	0	0	0	0	0	0	1	1	1	1
GO:0030202	2	2	100	1.13	0	0	0	0	0	0	0	0	0	1	1	1	1
GO:0030206	1	1	100	1.13	0	0	0	0	0	0	0	0	0	1	1	1	1
GO:0030210	1	1	100	1.13	0	0	0	0	0	0	0	0	0	1	1	1	1
GO:0030216	9	4	44.44	0.5	2	22.22	6.36	1	11.11	2.39	2	22.22	6.27	1	1	1	1
GO:0030217	3	3	100	1.13	0	0	0	0	0	0	0	0	0	1	1	1	1
GO:0030218	4	4	100	1.13	0	0	0	0	0	0	0	0	0	1	1	1	1
GO:0030224	1	1	100	1.13	0	0	0	0	0	0	0	0	0	1	1	1	1

GO:0030225	3	3	100	1.13	0	0	0	0	0	0	0	0	0	1	1	1	1
GO:0030226	1	1	100	1.13	0	0	0	0	0	0	0	0	0	1	1	1	1
GO:0030234	5	3	60	0.68	1	20	5.73	1	20	4.31	0	0	0	1	1	1	1
GO:0030236	1	0	0	0	0	0	0	0	0	0	1	100	28.22	1	1	1	1
GO:0030238	1	1	100	1.13	0	0	0	0	0	0	0	0	0	1	1	1	1
GO:0030246	6	6	100	1.13	0	0	0	0	0	0	0	0	0	1	1	1	1
GO:0030250	1	1	100	1.13	0	0	0	0	0	0	0	0	0	1	1	1	1
GO:0030261	1	1	100	1.13	0	0	0	0	0	0	0	0	0	1	1	1	1
GO:0030264	1	1	100	1.13	0	0	0	0	0	0	0	0	0	1	1	1	1
GO:0030276	5	5	100	1.13	0	0	0	0	0	0	0	0	0	1	1	1	1
GO:0030278	4	4	100	1.13	0	0	0	0	0	0	0	0	0	1	1	1	1
GO:0030282	3	3	100	1.13	0	0	0	0	0	0	0	0	0	1	1	1	1
GO:0030285	4	3	75	0.85	1	25	7.16	0	0	0	0	0	0	1	1	1	1
GO:0030286	12	12	100	1.13	0	0	0	0	0	0	0	0	0	1	1	1	1
GO:0030288	2	2	100	1.13	0	0	0	0	0	0	0	0	0	1	1	1	1
GO:0030295	1	1	100	1.13	0	0	0	0	0	0	0	0	0	1	1	1	1
GO:0030300	5	5	100	1.13	0	0	0	0	0	0	0	0	0	1	1	1	1
GO:0030304	2	0	0	0	0	0	0	0	0	0	2	100	28.22	1	1	1	1
GO:0030306	2	2	100	1.13	0	0	0	0	0	0	0	0	0	1	1	1	1
GO:0030308	6	6	100	1.13	0	0	0	0	0	0	0	0	0	1	1	1	1
GO:0030311	1	1	100	1.13	0	0	0	0	0	0	0	0	0	1	1	1	1
GO:0030316	2	2	100	1.13	0	0	0	0	0	0	0	0	0	1	1	1	1
GO:0030317	4	3	75	0.85	0	0	0	0	0	0	1	25	7.06	1	1	1	1
GO:0030318	2	2	100	1.13	0	0	0	0	0	0	0	0	0	1	1	1	1
GO:0030320	1	0	0	0	0	0	0	1	100	21.53	0	0	0	1	1	1	1
GO:0030321	1	1	100	1.13	0	0	0	0	0	0	0	0	0	1	1	1	1
GO:0030323	1	1	100	1.13	0	0	0	0	0	0	0	0	0	1	1	1	1
GO:0030324	11	10	90.91	1.03	0	0	0	1	9.09	1.96	0	0	0	1	1	1	1
GO:0030326	4	4	100	1.13	0	0	0	0	0	0	0	0	0	1	1	1	1
GO:0030327	3	2	66.67	0.75	1	33.33	9.54	0	0	0	0	0	0	1	1	1	1
GO:0030328	3	3	100	1.13	0	0	0	0	0	0	0	0	0	1	1	1	1
GO:0030329	2	2	100	1.13	0	0	0	0	0	0	0	0	0	1	1	1	1
GO:0030330	1	1	100	1.13	0	0	0	0	0	0	0	0	0	1	1	1	1
GO:0030332	1	1	100	1.13	0	0	0	0	0	0	0	0	0	1	1	1	1
GO:0030333	3	1	33.33	0.38	0	0	0	2	66.67	14.35	0	0	0	1	1	1	1
GO:0030334	5	3	60	0.68	1	20	5.73	0	0	0	1	20	5.64	1	1	1	1
GO:0030335	3	2	66.67	0.75	0	0	0	0	0	0	1	33.33	9.41	1	1	1	1
GO:0030336	2	2	100	1.13	0	0	0	0	0	0	0	0	0	1	1	1	1
GO:0030337	3	3	100	1.13	0	0	0	0	0	0	0	0	0	1	1	1	1
GO:0030346	1	1	100	1.13	0	0	0	0	0	0	0	0	0	1	1	1	1
GO:0030350	4	4	100	1.13	0	0	0	0	0	0	0	0	0	1	1	1	1

GO:0030355	1	1	100	1.13	0	0	0	0	0	0	0	0	0	1	1	1	1
GO:0030368	1	1	100	1.13	0	0	0	0	0	0	0	0	0	1	1	1	1
GO:0030371	1	1	100	1.13	0	0	0	0	0	0	0	0	0	1	1	1	1
GO:0030374	2	2	100	1.13	0	0	0	0	0	0	0	0	0	1	1	1	1
GO:0030377	3	3	100	1.13	0	0	0	0	0	0	0	0	0	1	1	1	1
GO:0030384	3	3	100	1.13	0	0	0	0	0	0	0	0	0	1	1	1	1
GO:0030409	1	1	100	1.13	0	0	0	0	0	0	0	0	0	1	1	1	1
GO:0030412	1	1	100	1.13	0	0	0	0	0	0	0	0	0	1	1	1	1
GO:0030414	2	1	50	0.57	0	0	0	0	0	0	1	50	14.11	1	1	1	1
GO:0030424	2	1	50	0.57	1	50	14.32	0	0	0	0	0	0	1	1	1	1
GO:0030425	3	3	100	1.13	0	0	0	0	0	0	0	0	0	1	1	1	1
GO:0030426	3	3	100	1.13	0	0	0	0	0	0	0	0	0	1	1	1	1
GO:0030433	9	9	100	1.13	0	0	0	0	0	0	0	0	0	1	1	1	1
GO:0030478	2	1	50	0.57	1	50	14.32	0	0	0	0	0	0	1	1	1	1
GO:0030484	2	2	100	1.13	0	0	0	0	0	0	0	0	0	1	1	1	1
GO:0030497	3	3	100	1.13	0	0	0	0	0	0	0	0	0	1	1	1	1
GO:0030501	2	2	100	1.13	0	0	0	0	0	0	0	0	0	1	1	1	1
GO:0030502	1	1	100	1.13	0	0	0	0	0	0	0	0	0	1	1	1	1
GO:0030506	1	0	0	0	1	100	28.63	0	0	0	0	0	0	1	1	1	1
GO:0030509	1	1	100	1.13	0	0	0	0	0	0	0	0	0	1	1	1	1
GO:0030512	3	2	66.67	0.75	0	0	0	0	0	0	1	33.33	9.41	1	1	1	1
GO:0030513	2	2	100	1.13	0	0	0	0	0	0	0	0	0	1	1	1	1
GO:0030514	5	4	80	0.91	0	0	0	0	0	0	1	20	5.64	1	1	1	1
GO:0030515	1	1	100	1.13	0	0	0	0	0	0	0	0	0	1	1	1	1
GO:0030519	6	4	66.67	0.75	0	0	0	2	33.33	7.18	0	0	0	1	1	1	1
GO:0030520	1	1	100	1.13	0	0	0	0	0	0	0	0	0	1	1	1	1
GO:0030528	16	14	87.5	0.99	2	12.5	3.58	0	0	0	0	0	0	1	1	1	1
GO:0030529	75	70	93.33	1.06	0	0	0	5	6.67	1.44	0	0	0	1	1	1	1
GO:0030532	3	3	100	1.13	0	0	0	0	0	0	0	0	0	1	1	1	1
GO:0030539	2	0	0	0	0	0	0	0	0	0	2	100	28.22	1	1	1	1
GO:0030544	2	2	100	1.13	0	0	0	0	0	0	0	0	0	1	1	1	1
GO:0030552	6	6	100	1.13	0	0	0	0	0	0	0	0	0	1	1	1	1
GO:0030553	1	1	100	1.13	0	0	0	0	0	0	0	0	0	1	1	1	1
GO:0030574	15	5	33.33	0.38	5	33.33	9.54	5	33.33	7.18	0	0	0	1	0.521704658	1	1
GO:0030578	1	1	100	1.13	0	0	0	0	0	0	0	0	0	1	1	1	1
GO:0030593	20	5	25	0.28	4	20	5.73	6	30	6.46	5	25	7.06	1	1	1	1
GO:0030594	6	3	50	0.57	1	16.67	4.77	0	0	0	2	33.33	9.41	1	1	1	1
GO:0030595	3	2	66.67	0.75	0	0	0	1	33.33	7.18	0	0	0	1	1	1	1
GO:0030641	1	1	100	1.13	0	0	0	0	0	0	0	0	0	1	1	1	1
GO:0030643	1	1	100	1.13	0	0	0	0	0	0	0	0	0	1	1	1	1
GO:0030659	2	2	100	1.13	0	0	0	0	0	0	0	0	0	1	1	1	1

GO:0030672	3	3	100	1.13	0	0	0	0	0	0	0	0	0	1	1	1	1
GO:0030674	1	1	100	1.13	0	0	0	0	0	0	0	0	0	1	1	1	1
GO:0030675	1	1	100	1.13	0	0	0	0	0	0	0	0	0	1	1	1	1
GO:0030693	14	12	85.71	0.97	0	0	0	1	7.14	1.54	1	7.14	2.02	1	1	1	1
GO:0030851	4	4	100	1.13	0	0	0	0	0	0	0	0	0	1	1	1	1
GO:0030853	1	0	0	0	0	0	0	1	100	21.53	0	0	0	1	1	1	1
GO:0030856	6	3	50	0.57	0	0	0	1	16.67	3.59	2	33.33	9.41	1	1	1	1
GO:0030866	3	3	100	1.13	0	0	0	0	0	0	0	0	0	1	1	1	1
GO:0030889	4	0	0	0	0	0	0	4	100	21.53	0	0	0	1	1	0.021406639	1
GO:0030890	1	1	100	1.13	0	0	0	0	0	0	0	0	0	1	1	1	1
GO:0030900	1	1	100	1.13	0	0	0	0	0	0	0	0	0	1	1	1	1
GO:0035026	3	1	33.33	0.38	0	0	0	2	66.67	14.35	0	0	0	1	1	1	1
GO:0035035	4	4	100	1.13	0	0	0	0	0	0	0	0	0	1	1	1	1
GO:0040007	8	4	50	0.57	1	12.5	3.58	2	25	5.38	1	12.5	3.53	1	1	1	1
GO:0040008	8	8	100	1.13	0	0	0	0	0	0	0	0	0	1	1	1	1
GO:0040009	5	5	100	1.13	0	0	0	0	0	0	0	0	0	1	1	1	1
GO:0040014	4	3	75	0.85	1	25	7.16	0	0	0	0	0	0	1	1	1	1
GO:0040015	1	1	100	1.13	0	0	0	0	0	0	0	0	0	1	1	1	1
GO:0040016	3	3	100	1.13	0	0	0	0	0	0	0	0	0	1	1	1	1
GO:0040018	3	2	66.67	0.75	0	0	0	1	33.33	7.18	0	0	0	1	1	1	1
GO:0042035	5	5	100	1.13	0	0	0	0	0	0	0	0	0	1	1	1	1
GO:0042036	1	1	100	1.13	0	0	0	0	0	0	0	0	0	1	1	1	1
GO:0042053	1	1	100	1.13	0	0	0	0	0	0	0	0	0	1	1	1	1
GO:0042054	4	3	75	0.85	1	25	7.16	0	0	0	0	0	0	1	1	1	1
GO:0042055	1	1	100	1.13	0	0	0	0	0	0	0	0	0	1	1	1	1
GO:0042060	3	3	100	1.13	0	0	0	0	0	0	0	0	0	1	1	1	1
GO:0042088	3	3	100	1.13	0	0	0	0	0	0	0	0	0	1	1	1	1
GO:0042094	2	2	100	1.13	0	0	0	0	0	0	0	0	0	1	1	1	1
GO:0042098	3	3	100	1.13	0	0	0	0	0	0	0	0	0	1	1	1	1
GO:0042100	3	3	100	1.13	0	0	0	0	0	0	0	0	0	1	1	1	1
GO:0042101	1	0	0	0	0	0	0	0	0	0	1	100	28.22	1	1	1	1
GO:0042102	1	1	100	1.13	0	0	0	0	0	0	0	0	0	1	1	1	1
GO:0042110	8	7	87.5	0.99	0	0	0	0	0	0	1	12.5	3.53	1	1	1	1
GO:0042113	6	3	50	0.57	0	0	0	1	16.67	3.59	2	33.33	9.41	1	1	1	1
GO:0042116	1	0	0	0	1	100	28.63	0	0	0	0	0	0	1	1	1	1
GO:0042127	25	25	100	1.13	0	0	0	0	0	0	0	0	0	1	1	1	1
GO:0042129	1	1	100	1.13	0	0	0	0	0	0	0	0	0	1	1	1	1
GO:0042130	1	0	0	0	0	0	0	0	0	0	1	100	28.22	1	1	1	1
GO:0042132	4	4	100	1.13	0	0	0	0	0	0	0	0	0	1	1	1	1
GO:0042135	2	1	50	0.57	1	50	14.32	0	0	0	0	0	0	1	1	1	1
GO:0042136	3	3	100	1.13	0	0	0	0	0	0	0	0	0	1	1	1	1

GO:0042157	8	7	87.5	0.99	1	12.5	3.58	0	0	0	0	0	0	1	1	1	1
GO:0042162	1	1	100	1.13	0	0	0	0	0	0	0	0	0	1	1	1	1
GO:0042167	2	2	100	1.13	0	0	0	0	0	0	0	0	0	1	1	1	1
GO:0042168	3	3	100	1.13	0	0	0	0	0	0	0	0	0	1	1	1	1
GO:0042169	2	0	0	0	0	0	0	2	100	21.53	0	0	0	1	1	1	1
GO:0042171	2	2	100	1.13	0	0	0	0	0	0	0	0	0	1	1	1	1
GO:0042176	5	5	100	1.13	0	0	0	0	0	0	0	0	0	1	1	1	1
GO:0042246	3	2	66.67	0.75	0	0	0	0	0	0	1	33.33	9.41	1	1	1	1
GO:0042254	1	1	100	1.13	0	0	0	0	0	0	0	0	0	1	1	1	1
GO:0042273	3	3	100	1.13	0	0	0	0	0	0	0	0	0	1	1	1	1
GO:0042287	1	1	100	1.13	0	0	0	0	0	0	0	0	0	1	1	1	1
GO:0042288	1	1	100	1.13	0	0	0	0	0	0	0	0	0	1	1	1	1
GO:0042301	3	3	100	1.13	0	0	0	0	0	0	0	0	0	1	1	1	1
GO:0042306	1	1	100	1.13	0	0	0	0	0	0	0	0	0	1	1	1	1
GO:0042383	2	2	100	1.13	0	0	0	0	0	0	0	0	0	1	1	1	1
GO:0042392	2	2	100	1.13	0	0	0	0	0	0	0	0	0	1	1	1	1
GO:0042393	6	6	100	1.13	0	0	0	0	0	0	0	0	0	1	1	1	1
GO:0042416	1	1	100	1.13	0	0	0	0	0	0	0	0	0	1	1	1	1
GO:0042417	1	1	100	1.13	0	0	0	0	0	0	0	0	0	1	1	1	1
GO:0042420	3	2	66.67	0.75	1	33.33	9.54	0	0	0	0	0	0	1	1	1	1
GO:0042423	2	2	100	1.13	0	0	0	0	0	0	0	0	0	1	1	1	1
GO:0042427	1	1	100	1.13	0	0	0	0	0	0	0	0	0	1	1	1	1
GO:0042462	1	1	100	1.13	0	0	0	0	0	0	0	0	0	1	1	1	1
GO:0042472	3	3	100	1.13	0	0	0	0	0	0	0	0	0	1	1	1	1
GO:0042475	4	4	100	1.13	0	0	0	0	0	0	0	0	0	1	1	1	1
GO:0042487	2	2	100	1.13	0	0	0	0	0	0	0	0	0	1	1	1	1
GO:0042488	2	2	100	1.13	0	0	0	0	0	0	0	0	0	1	1	1	1
GO:0042493	4	4	100	1.13	0	0	0	0	0	0	0	0	0	1	1	1	1
GO:0042535	4	1	25	0.28	0	0	0	0	0	0	3	75	21.17	1	1	1	0.815577835
GO:0042542	2	2	100	1.13	0	0	0	0	0	0	0	0	0	1	1	1	1
GO:0042552	7	5	71.43	0.81	2	28.57	8.18	0	0	0	0	0	0	1	1	1	1
GO:0042554	1	1	100	1.13	0	0	0	0	0	0	0	0	0	1	1	1	1
GO:0042564	1	1	100	1.13	0	0	0	0	0	0	0	0	0	1	1	1	1
GO:0042577	3	1	33.33	0.38	0	0	0	1	33.33	7.18	1	33.33	9.41	1	1	1	1
GO:0042578	4	4	100	1.13	0	0	0	0	0	0	0	0	0	1	1	1	1
GO:0042589	3	3	100	1.13	0	0	0	0	0	0	0	0	0	1	1	1	1
GO:0042590	4	1	25	0.28	0	0	0	0	0	0	3	75	21.17	1	1	1	0.815577835
GO:0042591	14	7	50	0.57	0	0	0	7	50	10.76	0	0	0	1	1	0.006542489	1
GO:0042593	2	2	100	1.13	0	0	0	0	0	0	0	0	0	1	1	1	1
GO:0042598	3	3	100	1.13	0	0	0	0	0	0	0	0	0	1	1	1	1
GO:0042605	6	5	83.33	0.94	0	0	0	1	16.67	3.59	0	0	0	1	1	1	1

GO:0042606	1	1	100	1.13	0	0	0	0	0	0	0	0	0	1	1	1	1
GO:0042607	1	1	100	1.13	0	0	0	0	0	0	0	0	0	1	1	1	1
GO:0042613	4	3	75	0.85	0	0	0	1	25	5.38	0	0	0	1	1	1	1
GO:0042623	9	9	100	1.13	0	0	0	0	0	0	0	0	0	1	1	1	1
GO:0042632	3	3	100	1.13	0	0	0	0	0	0	0	0	0	1	1	1	1
GO:0042692	1	1	100	1.13	0	0	0	0	0	0	0	0	0	1	1	1	1
GO:0042733	2	2	100	1.13	0	0	0	0	0	0	0	0	0	1	1	1	1
GO:0042742	4	2	50	0.57	0	0	0	1	25	5.38	1	25	7.06	1	1	1	1
GO:0042765	1	1	100	1.13	0	0	0	0	0	0	0	0	0	1	1	1	1
GO:0042780	1	1	100	1.13	0	0	0	0	0	0	0	0	0	1	1	1	1
GO:0042787	1	1	100	1.13	0	0	0	0	0	0	0	0	0	1	1	1	1
GO:0042802	3	3	100	1.13	0	0	0	0	0	0	0	0	0	1	1	1	1
GO:0042803	12	10	83.33	0.94	0	0	0	2	16.67	3.59	0	0	0	1	1	1	1
GO:0042804	4	4	100	1.13	0	0	0	0	0	0	0	0	0	1	1	1	1
GO:0042806	1	1	100	1.13	0	0	0	0	0	0	0	0	0	1	1	1	1
GO:0042808	2	2	100	1.13	0	0	0	0	0	0	0	0	0	1	1	1	1
GO:0042809	1	1	100	1.13	0	0	0	0	0	0	0	0	0	1	1	1	1
GO:0042830	1	1	100	1.13	0	0	0	0	0	0	0	0	0	1	1	1	1
GO:0042921	1	1	100	1.13	0	0	0	0	0	0	0	0	0	1	1	1	1
GO:0042981	58	53	91.38	1.03	1	1.72	0.49	2	3.45	0.74	2	3.45	0.97	1	1	1	1
GO:0042989	2	2	100	1.13	0	0	0	0	0	0	0	0	0	1	1	1	1
GO:0042994	2	2	100	1.13	0	0	0	0	0	0	0	0	0	1	1	1	1
GO:0043001	3	1	33.33	0.38	1	33.33	9.54	1	33.33	7.18	0	0	0	1	1	1	1
GO:0043011	2	2	100	1.13	0	0	0	0	0	0	0	0	0	1	1	1	1
GO:0043015	1	1	100	1.13	0	0	0	0	0	0	0	0	0	1	1	1	1
GO:0043021	3	3	100	1.13	0	0	0	0	0	0	0	0	0	1	1	1	1
GO:0043022	5	5	100	1.13	0	0	0	0	0	0	0	0	0	1	1	1	1
GO:0043026	1	1	100	1.13	0	0	0	0	0	0	0	0	0	1	1	1	1
GO:0043029	1	0	0	0	0	0	0	0	0	0	1	100	28.22	1	1	1	1
GO:0043065	3	2	66.67	0.75	0	0	0	1	33.33	7.18	0	0	0	1	1	1	1
GO:0043066	8	8	100	1.13	0	0	0	0	0	0	0	0	0	1	1	1	1
GO:0043071	1	1	100	1.13	0	0	0	0	0	0	0	0	0	1	1	1	1
GO:0043085	2	2	100	1.13	0	0	0	0	0	0	0	0	0	1	1	1	1
GO:0043087	5	5	100	1.13	0	0	0	0	0	0	0	0	0	1	1	1	1
GO:0043123	2	2	100	1.13	0	0	0	0	0	0	0	0	0	1	1	1	1
GO:0045004	3	3	100	1.13	0	0	0	0	0	0	0	0	0	1	1	1	1
GO:0045010	4	3	75	0.85	0	0	0	1	25	5.38	0	0	0	1	1	1	1
GO:0045012	10	5	50	0.57	0	0	0	5	50	10.76	0	0	0	1	1	0.216534029	1
GO:0045026	1	1	100	1.13	0	0	0	0	0	0	0	0	0	1	1	1	1
GO:0045029	1	1	100	1.13	0	0	0	0	0	0	0	0	0	1	1	1	1
GO:0045045	9	9	100	1.13	0	0	0	0	0	0	0	0	0	1	1	1	1

GO:0045055	3	3	100	1.13	0	0	0	0	0	0	0	0	0	1	1	1	1
GO:0045059	3	1	33.33	0.38	0	0	0	1	33.33	7.18	1	33.33	9.41	1	1	1	1
GO:0045079	1	0	0	0	0	0	0	1	100	21.53	0	0	0	1	1	1	1
GO:0045080	1	0	0	0	0	0	0	1	100	21.53	0	0	0	1	1	1	1
GO:0045082	1	1	100	1.13	0	0	0	0	0	0	0	0	0	1	1	1	1
GO:0045084	7	7	100	1.13	0	0	0	0	0	0	0	0	0	1	1	1	1
GO:0045087	2	0	0	0	2	100	28.63	0	0	0	0	0	0	1	1	1	1
GO:0045095	1	0	0	0	0	0	0	0	0	0	1	100	28.22	1	1	1	1
GO:0045098	3	3	100	1.13	0	0	0	0	0	0	0	0	0	1	1	1	1
GO:0045103	3	3	100	1.13	0	0	0	0	0	0	0	0	0	1	1	1	1
GO:0045104	1	1	100	1.13	0	0	0	0	0	0	0	0	0	1	1	1	1
GO:0045116	2	2	100	1.13	0	0	0	0	0	0	0	0	0	1	1	1	1
GO:0045120	7	7	100	1.13	0	0	0	0	0	0	0	0	0	1	1	1	1
GO:0045121	17	17	100	1.13	0	0	0	0	0	0	0	0	0	1	1	1	1
GO:0045123	17	6	35.29	0.4	4	23.53	6.74	5	29.41	6.33	2	11.76	3.32	1	1	1	1
GO:0045127	1	1	100	1.13	0	0	0	0	0	0	0	0	0	1	1	1	1
GO:0045134	1	1	100	1.13	0	0	0	0	0	0	0	0	0	1	1	1	1
GO:0045160	2	2	100	1.13	0	0	0	0	0	0	0	0	0	1	1	1	1
GO:0045165	6	6	100	1.13	0	0	0	0	0	0	0	0	0	1	1	1	1
GO:0045176	2	2	100	1.13	0	0	0	0	0	0	0	0	0	1	1	1	1
GO:0045177	5	5	100	1.13	0	0	0	0	0	0	0	0	0	1	1	1	1
GO:0045182	3	3	100	1.13	0	0	0	0	0	0	0	0	0	1	1	1	1
GO:0045190	1	1	100	1.13	0	0	0	0	0	0	0	0	0	1	1	1	1
GO:0045202	25	25	100	1.13	0	0	0	0	0	0	0	0	0	1	1	1	1
GO:0045210	1	1	100	1.13	0	0	0	0	0	0	0	0	0	1	1	1	1
GO:0045211	3	3	100	1.13	0	0	0	0	0	0	0	0	0	1	1	1	1
GO:0045217	1	1	100	1.13	0	0	0	0	0	0	0	0	0	1	1	1	1
GO:0045226	1	1	100	1.13	0	0	0	0	0	0	0	0	0	1	1	1	1
GO:0045239	1	1	100	1.13	0	0	0	0	0	0	0	0	0	1	1	1	1
GO:0045254	1	1	100	1.13	0	0	0	0	0	0	0	0	0	1	1	1	1
GO:0045255	4	4	100	1.13	0	0	0	0	0	0	0	0	0	1	1	1	1
GO:0045259	2	2	100	1.13	0	0	0	0	0	0	0	0	0	1	1	1	1
GO:0045285	1	1	100	1.13	0	0	0	0	0	0	0	0	0	1	1	1	1
GO:0045294	1	1	100	1.13	0	0	0	0	0	0	0	0	0	1	1	1	1
GO:0045309	6	6	100	1.13	0	0	0	0	0	0	0	0	0	1	1	1	1
GO:0045333	2	2	100	1.13	0	0	0	0	0	0	0	0	0	1	1	1	1
GO:0045348	1	0	0	0	0	0	0	1	100	21.53	0	0	0	1	1	1	1
GO:0045359	1	0	0	0	0	0	0	1	100	21.53	0	0	0	1	1	1	1
GO:0045408	1	1	100	1.13	0	0	0	0	0	0	0	0	0	1	1	1	1
GO:0045409	1	0	0	0	0	0	0	1	100	21.53	0	0	0	1	1	1	1
GO:0045410	3	2	66.67	0.75	0	0	0	1	33.33	7.18	0	0	0	1	1	1	1

GO:0045444	5	5	100	1.13	0	0	0	0	0	0	0	0	0	1	1	1	1
GO:0045445	1	1	100	1.13	0	0	0	0	0	0	0	0	0	1	1	1	1
GO:0045446	2	2	100	1.13	0	0	0	0	0	0	0	0	0	1	1	1	1
GO:0045449	38	35	92.11	1.04	2	5.26	1.51	0	0	0	1	2.63	0.74	1	1	1	1
GO:0045453	4	4	100	1.13	0	0	0	0	0	0	0	0	0	1	1	1	1
GO:0045509	1	1	100	1.13	0	0	0	0	0	0	0	0	0	1	1	1	1
GO:0045523	3	3	100	1.13	0	0	0	0	0	0	0	0	0	1	1	1	1
GO:0045569	1	1	100	1.13	0	0	0	0	0	0	0	0	0	1	1	1	1
GO:0045576	4	1	25	0.28	0	0	0	0	0	0	3	75	21.17	1	1	1	0.815577835
GO:0045579	2	1	50	0.57	0	0	0	1	50	10.76	0	0	0	1	1	1	1
GO:0045580	1	0	0	0	0	0	0	1	100	21.53	0	0	0	1	1	1	1
GO:0045582	4	3	75	0.85	0	0	0	1	25	5.38	0	0	0	1	1	1	1
GO:0045595	1	1	100	1.13	0	0	0	0	0	0	0	0	0	1	1	1	1
GO:0045596	1	1	100	1.13	0	0	0	0	0	0	0	0	0	1	1	1	1
GO:0045597	1	1	100	1.13	0	0	0	0	0	0	0	0	0	1	1	1	1
GO:0045603	2	2	100	1.13	0	0	0	0	0	0	0	0	0	1	1	1	1
GO:0045621	1	0	0	0	0	0	0	1	100	21.53	0	0	0	1	1	1	1
GO:0045637	1	1	100	1.13	0	0	0	0	0	0	0	0	0	1	1	1	1
GO:0045639	1	1	100	1.13	0	0	0	0	0	0	0	0	0	1	1	1	1
GO:0045648	1	0	0	0	0	0	0	1	100	21.53	0	0	0	1	1	1	1
GO:0045656	1	0	0	0	0	0	0	1	100	21.53	0	0	0	1	1	1	1
GO:0045659	1	0	0	0	0	0	0	1	100	21.53	0	0	0	1	1	1	1
GO:0045661	1	1	100	1.13	0	0	0	0	0	0	0	0	0	1	1	1	1
GO:0045663	2	2	100	1.13	0	0	0	0	0	0	0	0	0	1	1	1	1
GO:0045664	2	2	100	1.13	0	0	0	0	0	0	0	0	0	1	1	1	1
GO:0045666	2	2	100	1.13	0	0	0	0	0	0	0	0	0	1	1	1	1
GO:0045668	1	1	100	1.13	0	0	0	0	0	0	0	0	0	1	1	1	1
GO:0045670	5	5	100	1.13	0	0	0	0	0	0	0	0	0	1	1	1	1
GO:0045671	2	0	0	0	0	0	0	2	100	21.53	0	0	0	1	1	1	1
GO:0045672	4	4	100	1.13	0	0	0	0	0	0	0	0	0	1	1	1	1
GO:0045735	6	5	83.33	0.94	1	16.67	4.77	0	0	0	0	0	0	1	1	1	1
GO:0045736	3	3	100	1.13	0	0	0	0	0	0	0	0	0	1	1	1	1
GO:0045745	1	1	100	1.13	0	0	0	0	0	0	0	0	0	1	1	1	1
GO:0045749	4	4	100	1.13	0	0	0	0	0	0	0	0	0	1	1	1	1
GO:0045765	2	2	100	1.13	0	0	0	0	0	0	0	0	0	1	1	1	1
GO:0045766	4	3	75	0.85	0	0	0	0	0	0	1	25	7.06	1	1	1	1
GO:0045773	2	2	100	1.13	0	0	0	0	0	0	0	0	0	1	1	1	1
GO:0045779	3	2	66.67	0.75	0	0	0	1	33.33	7.18	0	0	0	1	1	1	1
GO:0045786	30	30	100	1.13	0	0	0	0	0	0	0	0	0	1	1	1	1
GO:0045806	2	2	100	1.13	0	0	0	0	0	0	0	0	0	1	1	1	1
GO:0045807	1	1	100	1.13	0	0	0	0	0	0	0	0	0	1	1	1	1

GO:0045834	1	0	0	0	0	0	0	0	0	0	1	100	28.22	1	1	1	1
GO:0045859	1	1	100	1.13	0	0	0	0	0	0	0	0	0	1	1	1	1
GO:0045861	1	1	100	1.13	0	0	0	0	0	0	0	0	0	1	1	1	1
GO:0045884	1	1	100	1.13	0	0	0	0	0	0	0	0	0	1	1	1	1
GO:0045892	1	1	100	1.13	0	0	0	0	0	0	0	0	0	1	1	1	1
GO:0045893	6	6	100	1.13	0	0	0	0	0	0	0	0	0	1	1	1	1
GO:0045910	2	2	100	1.13	0	0	0	0	0	0	0	0	0	1	1	1	1
GO:0045926	2	2	100	1.13	0	0	0	0	0	0	0	0	0	1	1	1	1
GO:0045930	1	1	100	1.13	0	0	0	0	0	0	0	0	0	1	1	1	1
GO:0045941	6	6	100	1.13	0	0	0	0	0	0	0	0	0	1	1	1	1
GO:0045944	42	39	92.86	1.05	0	0	0	2	4.76	1.03	1	2.38	0.67	1	1	1	1
GO:0046032	2	2	100	1.13	0	0	0	0	0	0	0	0	0	1	1	1	1
GO:0046208	3	3	100	1.13	0	0	0	0	0	0	0	0	0	1	1	1	1
GO:0046328	3	3	100	1.13	0	0	0	0	0	0	0	0	0	1	1	1	1
GO:0046330	1	1	100	1.13	0	0	0	0	0	0	0	0	0	1	1	1	1
GO:0046332	1	1	100	1.13	0	0	0	0	0	0	0	0	0	1	1	1	1
GO:0046340	1	1	100	1.13	0	0	0	0	0	0	0	0	0	1	1	1	1
GO:0046398	1	1	100	1.13	0	0	0	0	0	0	0	0	0	1	1	1	1
GO:0046488	6	6	100	1.13	0	0	0	0	0	0	0	0	0	1	1	1	1
GO:0046513	3	3	100	1.13	0	0	0	0	0	0	0	0	0	1	1	1	1
GO:0046514	1	1	100	1.13	0	0	0	0	0	0	0	0	0	1	1	1	1
GO:0046527	1	1	100	1.13	0	0	0	0	0	0	0	0	0	1	1	1	1
GO:0046540	2	2	100	1.13	0	0	0	0	0	0	0	0	0	1	1	1	1
GO:0046581	3	2	66.67	0.75	0	0	0	0	0	0	1	33.33	9.41	1	1	1	1
GO:0046592	3	3	100	1.13	0	0	0	0	0	0	0	0	0	1	1	1	1
GO:0046604	4	4	100	1.13	0	0	0	0	0	0	0	0	0	1	1	1	1
GO:0046626	7	7	100	1.13	0	0	0	0	0	0	0	0	0	1	1	1	1
GO:0046651	3	3	100	1.13	0	0	0	0	0	0	0	0	0	1	1	1	1
GO:0046652	7	7	100	1.13	0	0	0	0	0	0	0	0	0	1	1	1	1
GO:0046653	1	1	100	1.13	0	0	0	0	0	0	0	0	0	1	1	1	1
GO:0046685	2	1	50	0.57	0	0	0	1	50	10.76	0	0	0	1	1	1	1
GO:0046709	2	2	100	1.13	0	0	0	0	0	0	0	0	0	1	1	1	1
GO:0046716	4	3	75	0.85	0	0	0	0	0	0	1	25	7.06	1	1	1	1
GO:0046777	15	14	93.33	1.06	1	6.67	1.91	0	0	0	0	0	0	1	1	1	1
GO:0046831	2	2	100	1.13	0	0	0	0	0	0	0	0	0	1	1	1	1
GO:0046854	1	1	100	1.13	0	0	0	0	0	0	0	0	0	1	1	1	1
GO:0046872	44	43	97.73	1.11	0	0	0	1	2.27	0.49	0	0	0	1	1	1	1
GO:0046873	6	6	100	1.13	0	0	0	0	0	0	0	0	0	1	1	1	1
GO:0046879	2	2	100	1.13	0	0	0	0	0	0	0	0	0	1	1	1	1
GO:0046907	1	1	100	1.13	0	0	0	0	0	0	0	0	0	1	1	1	1
GO:0046933	71	67	94.37	1.07	1	1.41	0.4	3	4.23	0.91	0	0	0	1	1	1	1

GO:0046935	3	3	100	1.13	0	0	0	0	0	0	0	0	0	1	1	1	1
GO:0046961	71	67	94.37	1.07	1	1.41	0.4	3	4.23	0.91	0	0	0	1	1	1	1
GO:0046967	1	1	100	1.13	0	0	0	0	0	0	0	0	0	1	1	1	1
GO:0046979	1	1	100	1.13	0	0	0	0	0	0	0	0	0	1	1	1	1
GO:0046980	1	1	100	1.13	0	0	0	0	0	0	0	0	0	1	1	1	1
GO:0046982	7	7	100	1.13	0	0	0	0	0	0	0	0	0	1	1	1	1
GO:0047237	1	1	100	1.13	0	0	0	0	0	0	0	0	0	1	1	1	1
GO:0047238	1	1	100	1.13	0	0	0	0	0	0	0	0	0	1	1	1	1
GO:0047290	1	1	100	1.13	0	0	0	0	0	0	0	0	0	1	1	1	1
GO:0047499	4	4	100	1.13	0	0	0	0	0	0	0	0	0	1	1	1	1
GO:0047598	1	1	100	1.13	0	0	0	0	0	0	0	0	0	1	1	1	1
GO:0047760	1	0	0	0	0	0	0	1	100	21.53	0	0	0	1	1	1	1
GO:0048002	4	3	75	0.85	0	0	0	1	25	5.38	0	0	0	1	1	1	1
GO:0048004	2	2	100	1.13	0	0	0	0	0	0	0	0	0	1	1	1	1
GO:0048005	7	4	57.14	0.65	0	0	0	3	42.86	9.23	0	0	0	1	1	1	1
GO:0048008	3	3	100	1.13	0	0	0	0	0	0	0	0	0	1	1	1	1
GO:0048011	1	1	100	1.13	0	0	0	0	0	0	0	0	0	1	1	1	1
GO:0048037	3	3	100	1.13	0	0	0	0	0	0	0	0	0	1	1	1	1
GO:0048066	4	4	100	1.13	0	0	0	0	0	0	0	0	0	1	1	1	1
GO:0048147	1	1	100	1.13	0	0	0	0	0	0	0	0	0	1	1	1	1
GO:0048168	3	3	100	1.13	0	0	0	0	0	0	0	0	0	1	1	1	1
GO:0048227	1	1	100	1.13	0	0	0	0	0	0	0	0	0	1	1	1	1
GO:0048255	1	1	100	1.13	0	0	0	0	0	0	0	0	0	1	1	1	1
GO:0048256	3	3	100	1.13	0	0	0	0	0	0	0	0	0	1	1	1	1
GO:0048265	1	1	100	1.13	0	0	0	0	0	0	0	0	0	1	1	1	1
GO:0048268	1	1	100	1.13	0	0	0	0	0	0	0	0	0	1	1	1	1
GO:0048270	1	1	100	1.13	0	0	0	0	0	0	0	0	0	1	1	1	1
GO:0050031	1	1	100	1.13	0	0	0	0	0	0	0	0	0	1	1	1	1
GO:0050220	2	2	100	1.13	0	0	0	0	0	0	0	0	0	1	1	1	1
GO:0050508	1	1	100	1.13	0	0	0	0	0	0	0	0	0	1	1	1	1
GO:0050613	1	1	100	1.13	0	0	0	0	0	0	0	0	0	1	1	1	1
GO:0050653	1	1	100	1.13	0	0	0	0	0	0	0	0	0	1	1	1	1
GO:0050655	2	2	100	1.13	0	0	0	0	0	0	0	0	0	1	1	1	1
GO:0050660	2	2	100	1.13	0	0	0	0	0	0	0	0	0	1	1	1	1
GO:0050708	1	1	100	1.13	0	0	0	0	0	0	0	0	0	1	1	1	1
GO:0050714	2	2	100	1.13	0	0	0	0	0	0	0	0	0	1	1	1	1
GO:0050728	1	0	0	0	0	0	0	0	0	0	1	100	28.22	1	1	1	1
GO:0050729	1	1	100	1.13	0	0	0	0	0	0	0	0	0	1	1	1	1
GO:0050730	6	6	100	1.13	0	0	0	0	0	0	0	0	0	1	1	1	1
GO:0050766	12	6	50	0.57	0	0	0	3	25	5.38	3	25	7.06	1	1	1	1
GO:0050776	4	1	25	0.28	0	0	0	0	0	0	3	75	21.17	1	1	1	0.815577835

GO:0050777	4	0	0	0	0	0	0	4	100	21.53	0	0	0	1	1	0.021406639	1
GO:0050778	3	2	66.67	0.75	0	0	0	1	33.33	7.18	0	0	0	1	1	1	1
GO:0050808	1	1	100	1.13	0	0	0	0	0	0	0	0	0	1	1	1	1
GO:0050819	11	11	100	1.13	0	0	0	0	0	0	0	0	0	1	1	1	1
GO:0050821	4	4	100	1.13	0	0	0	0	0	0	0	0	0	1	1	1	1
GO:0050828	1	1	100	1.13	0	0	0	0	0	0	0	0	0	1	1	1	1
GO:0050869	1	0	0	0	0	0	0	1	100	21.53	0	0	0	1	1	1	1
GO:0050892	1	1	100	1.13	0	0	0	0	0	0	0	0	0	1	1	1	1
GO:0050896	5	5	100	1.13	0	0	0	0	0	0	0	0	0	1	1	1	1
GO:0050897	1	1	100	1.13	0	0	0	0	0	0	0	0	0	1	1	1	1
GO:0050909	2	2	100	1.13	0	0	0	0	0	0	0	0	0	1	1	1	1
GO:0050930	6	5	83.33	0.94	0	0	0	1	16.67	3.59	0	0	0	1	1	1	1
GO:0050983	1	1	100	1.13	0	0	0	0	0	0	0	0	0	1	1	1	1
GO:0051014	1	1	100	1.13	0	0	0	0	0	0	0	0	0	1	1	1	1
GO:0051016	4	3	75	0.85	0	0	0	1	25	5.38	0	0	0	1	1	1	1
GO:0051018	1	1	100	1.13	0	0	0	0	0	0	0	0	0	1	1	1	1

Library ID	Total	#A	%A	Change A	#B	%B	Change B	#C	%C	Change C	#D	%D	Change D	p-value A	p-value B	p-value C	p-value D
6	598	529	88.46	1	22	3.68	1.05	35	5.85	1.26	12	2.01	0.57	1	1	1	1
7	74	67	90.54	1.03	4	5.41	1.55	1	1.35	0.29	2	2.7	0.76	1	1	1	1
9	24	21	87.5	0.99	1	4.17	1.19	2	8.33	1.79	0	0	0	1	1	1	1
10	30	28	93.33	1.06	2	6.67	1.91	0	0	0	0	0	0	1	1	1	1
11	584	513	87.84	0.99	18	3.08	0.88	34	5.82	1.25	19	3.25	0.92	1	1	1	1
12	200	191	95.5	1.08	1	0.5	0.14	6	3	0.65	2	1	0.28	0.022153176	1	1	1
13	197	172	87.31	0.99	4	2.03	0.58	14	7.11	1.53	7	3.55	1	1	1	1	1
15	269	246	91.45	1.04	12	4.46	1.28	8	2.97	0.64	3	1.12	0.31	1	1	1	1
16	72	59	81.94	0.93	2	2.78	0.8	6	8.33	1.79	5	6.94	1.96	1	1	1	1
17	294	253	86.05	0.97	11	3.74	1.07	14	4.76	1.03	16	5.44	1.54	1	1	1	1
18	431	379	87.94	1	8	1.86	0.53	33	7.66	1.65	11	2.55	0.72	1	1	1	1
20	253	219	86.56	0.98	7	2.77	0.79	14	5.53	1.19	13	5.14	1.45	1	1	1	1
22	158	140	88.61	1	2	1.27	0.36	10	6.33	1.36	6	3.8	1.07	1	1	1	1
23	498	438	87.95	1	16	3.21	0.92	27	5.42	1.17	17	3.41	0.96	1	1	1	1
24	448	421	93.97	1.06	11	2.46	0.7	15	3.35	0.72	1	0.22	0.06	0.000820283	1	1	1
25	144	123	85.42	0.97	10	6.94	1.99	8	5.56	1.2	3	2.08	0.59	1	1	1	1
26	617	562	91.09	1.03	24	3.89	1.11	26	4.21	0.91	5	0.81	0.23	0.604831935	1	1	1
27	218	197	90.37	1.02	6	2.75	0.79	9	4.13	0.89	6	2.75	0.78	1	1	1	1
28	446	409	91.7	1.04	14	3.14	0.9	15	3.36	0.72	8	1.79	0.51	0.624479222	1	1	1
29	145	127	87.59	0.99	6	4.14	1.18	6	4.14	0.89	6	4.14	1.17	1	1	1	1
30	82	75	91.46	1.04	2	2.44	0.7	1	1.22	0.26	4	4.88	1.38	1	1	1	1
31	175	154	88	1	8	4.57	1.31	7	4	0.86	6	3.43	0.97	1	1	1	1
32	66	61	92.42	1.05	1	1.52	0.43	1	1.52	0.33	3	4.55	1.28	1	1	1	1

33	56	47	83.93	0.95	3	5.36	1.53	4	7.14	1.54	2	3.57	1.01	1	1	1	1
34	16	14	87.5	0.99	1	6.25	1.79	1	6.25	1.35	0	0	0	1	1	1	1
35	10	10	100	1.13	0	0	0	0	0	0	0	0	0	1	1	1	1
36	34	31	91.18	1.03	1	2.94	0.84	2	5.88	1.27	0	0	0	1	1	1	1
37	31	30	96.77	1.1	1	3.23	0.92	0	0	0	0	0	0	1	1	1	1
38	104	87	83.65	0.95	3	2.88	0.83	6	5.77	1.24	8	7.69	2.17	1	1	1	1
39	10	10	100	1.13	0	0	0	0	0	0	0	0	0	1	1	1	1
40	86	76	88.37	1	3	3.49	1	6	6.98	1.5	1	1.16	0.33	1	1	1	1
41	7	6	85.71	0.97	0	0	0	1	14.29	3.08	0	0	0	1	1	1	1
42	7	5	71.43	0.81	2	28.57	8.18	0	0	0	0	0	0	1	1	1	1
44	41	37	90.24	1.02	2	4.88	1.4	0	0	0	2	4.88	1.38	1	1	1	1
46	149	126	84.56	0.96	4	2.68	0.77	9	6.04	1.3	10	6.71	1.89	1	1	1	1
47	326	285	87.42	0.99	16	4.91	1.41	16	4.91	1.06	9	2.76	0.78	1	1	1	1
48	228	196	85.96	0.97	7	3.07	0.88	11	4.82	1.04	14	6.14	1.73	1	1	1	1
49	775	668	86.19	0.98	31	4	1.15	49	6.32	1.36	27	3.48	0.98	1	1	1	1
50	106	90	84.91	0.96	1	0.94	0.27	8	7.55	1.62	7	6.6	1.86	1	1	1	1
51	17	16	94.12	1.07	1	5.88	1.68	0	0	0	0	0	0	1	1	1	1
52	12	10	83.33	0.94	2	16.67	4.77	0	0	0	0	0	0	1	1	1	1
53	233	209	89.7	1.02	6	2.58	0.74	13	5.58	1.2	5	2.15	0.61	1	1	1	1
54	90	70	77.78	0.88	3	3.33	0.95	10	11.11	2.39	7	7.78	2.2	1	1	1	1
55	17	12	70.59	0.8	2	11.76	3.37	3	17.65	3.8	0	0	0	1	1	1	1
56	5	4	80	0.91	1	20	5.73	0	0	0	0	0	0	1	1	1	1
57	590	544	92.2	1.04	16	2.71	0.78	20	3.39	0.73	10	1.69	0.48	0.030483418	1	1	1
58	243	208	85.6	0.97	14	5.76	1.65	10	4.12	0.89	11	4.53	1.28	1	1	1	1
59	105	95	90.48	1.02	3	2.86	0.82	5	4.76	1.03	2	1.9	0.54	1	1	1	1
60	259	235	90.73	1.03	11	4.25	1.22	6	2.32	0.5	7	2.7	0.76	1	1	1	1
61	2	1	50	0.57	0	0	0	1	50	10.76	0	0	0	1	1	1	1
62	40	36	90	1.02	2	5	1.43	1	2.5	0.54	1	2.5	0.71	1	1	1	1
63	323	276	85.45	0.97	16	4.95	1.42	17	5.26	1.13	14	4.33	1.22	1	1	1	1
64	277	248	89.53	1.01	15	5.42	1.55	11	3.97	0.85	3	1.08	0.31	1	1	1	1
65	68	60	88.24	1	3	4.41	1.26	3	4.41	0.95	2	2.94	0.83	1	1	1	1
66	9	5	55.56	0.63	1	11.11	3.18	3	33.33	7.18	0	0	0	1	1	1	1
67	199	180	90.45	1.02	10	5.03	1.44	5	2.51	0.54	4	2.01	0.57	1	1	1	1
68	137	120	87.59	0.99	6	4.38	1.25	9	6.57	1.41	2	1.46	0.41	1	1	1	1
70	13	8	61.54	0.7	2	15.38	4.41	1	7.69	1.66	2	15.38	4.34	1	1	1	1
71	94	84	89.36	1.01	2	2.13	0.61	4	4.26	0.92	4	4.26	1.2	1	1	1	1
72	2	2	100	1.13	0	0	0	0	0	0	0	0	0	1	1	1	1
73	13	10	76.92	0.87	1	7.69	2.2	0	0	0	2	15.38	4.34	1	1	1	1
74	114	106	92.98	1.05	3	2.63	0.75	5	4.39	0.94	0	0	0	1	1	1	1
75	23	19	82.61	0.94	1	4.35	1.24	2	8.7	1.87	1	4.35	1.23	1	1	1	1
76	2	0	0	0	0	0	0	1	50	10.76	1	50	14.11	1	1	1	1

	77	1	0	0	0	0	0	0	0	0	0	1	100	28.22	1	1	1	1
	79	1	1	100	1.13	0	0	0	0	0	0	0	0	0	1	1	1	1
	80	87	77	88.51	1	5	5.75	1.65	3	3.45	0.74	2	2.3	0.65	1	1	1	1
	81	3	3	100	1.13	0	0	0	0	0	0	0	0	0	1	1	1	1
	82	3	1	33.33	0.38	0	0	0	2	66.67	14.35	0	0	0	1	1	1	1
	84	72	58	80.56	0.91	5	6.94	1.99	5	6.94	1.49	4	5.56	1.57	1	1	1	1
	85	3	3	100	1.13	0	0	0	0	0	0	0	0	0	1	1	1	1
	90	135	103	76.3	0.86	9	6.67	1.91	10	7.41	1.59	13	9.63	2.72	1	1	1	0.562755353
	91	157	127	80.89	0.92	5	3.18	0.91	10	6.37	1.37	15	9.55	2.7	1	1	1	0.2954558
	92	52	38	73.08	0.83	4	7.69	2.2	2	3.85	0.83	8	15.38	4.34	1	1	1	0.196353872
	93	212	182	85.85	0.97	10	4.72	1.35	12	5.66	1.22	8	3.77	1.07	1	1	1	1
	94	204	182	89.22	1.01	5	2.45	0.7	8	3.92	0.84	9	4.41	1.25	1	1	1	1
	95	224	189	84.38	0.96	8	3.57	1.02	16	7.14	1.54	11	4.91	1.39	1	1	1	1
	96	234	202	86.32	0.98	11	4.7	1.35	11	4.7	1.01	10	4.27	1.21	1	1	1	1
	98	219	172	78.54	0.89	14	6.39	1.83	20	9.13	1.97	13	5.94	1.68	1	1	1	1
	99	143	116	81.12	0.92	8	5.59	1.6	9	6.29	1.35	10	6.99	1.97	1	1	1	1
A0		43	38	88.37	1	1	2.33	0.67	0	0	0	4	9.3	2.63	1	1	1	1
A1		215	169	78.6	0.89	12	5.58	1.6	23	10.7	2.3	11	5.12	1.44	1	1	0.106395474	1
A2		289	253	87.54	0.99	12	4.15	1.19	8	2.77	0.6	16	5.54	1.56	1	1	1	1
A3		206	176	85.44	0.97	13	6.31	1.81	6	2.91	0.63	11	5.34	1.51	1	1	1	1
A4		291	239	82.13	0.93	7	2.41	0.69	18	6.19	1.33	27	9.28	2.62	1	1	1	0.007513119
A5		288	212	73.61	0.83	20	6.94	1.99	14	4.86	1.05	42	14.58	4.12	1	1	1	4.98E-11
A6		378	324	85.71	0.97	14	3.7	1.06	23	6.08	1.31	17	4.5	1.27	1	1	1	1
A7		278	242	87.05	0.99	18	6.47	1.85	10	3.6	0.77	8	2.88	0.81	1	1	1	1
A8		237	204	86.08	0.97	21	8.86	2.54	7	2.95	0.64	5	2.11	0.6	1	0.045636663	1	1
A9		239	214	89.54	1.01	5	2.09	0.6	9	3.77	0.81	11	4.6	1.3	1	1	1	1
AA		18	16	88.89	1.01	1	5.56	1.59	0	0	0	1	5.56	1.57	1	1	1	1
AB		14	12	85.71	0.97	1	7.14	2.05	1	7.14	1.54	0	0	0	1	1	1	1
AC		9	9	100	1.13	0	0	0	0	0	0	0	0	0	1	1	1	1
AD		14	12	85.71	0.97	0	0	0	1	7.14	1.54	1	7.14	2.02	1	1	1	1
AE		22	22	100	1.13	0	0	0	0	0	0	0	0	0	1	1	1	1
AF		12	11	91.67	1.04	0	0	0	1	8.33	1.79	0	0	0	1	1	1	1
AG		12	10	83.33	0.94	1	8.33	2.39	0	0	0	1	8.33	2.35	1	1	1	1
AH		20	19	95	1.08	1	5	1.43	0	0	0	0	0	0	1	1	1	1
AI		15	15	100	1.13	0	0	0	0	0	0	0	0	0	1	1	1	1
AJ		13	13	100	1.13	0	0	0	0	0	0	0	0	0	1	1	1	1
AK		13	13	100	1.13	0	0	0	0	0	0	0	0	0	1	1	1	1
AL		6	6	100	1.13	0	0	0	0	0	0	0	0	0	1	1	1	1
AM		17	17	100	1.13	0	0	0	0	0	0	0	0	0	1	1	1	1
AN		20	20	100	1.13	0	0	0	0	0	0	0	0	0	1	1	1	1
AO		13	11	84.62	0.96	1	7.69	2.2	1	7.69	1.66	0	0	0	1	1	1	1

AP	6	6	100	1.13	0	0	0	0	0	0	0	0	0	1	1	1	1
AQ	4	4	100	1.13	0	0	0	0	0	0	0	0	0	1	1	1	1
AR	16	13	81.25	0.92	0	0	0	3	18.75	4.04	0	0	0	1	1	1	1
AS	9	9	100	1.13	0	0	0	0	0	0	0	0	0	1	1	1	1
AT	6	3	50	0.57	0	0	0	2	33.33	7.18	1	16.67	4.7	1	1	1	1
AU	13	13	100	1.13	0	0	0	0	0	0	0	0	0	1	1	1	1
AV	11	9	81.82	0.93	0	0	0	0	0	0	2	18.18	5.13	1	1	1	1
AW	15	14	93.33	1.06	1	6.67	1.91	0	0	0	0	0	0	1	1	1	1
AX	11	10	90.91	1.03	0	0	0	1	9.09	1.96	0	0	0	1	1	1	1
AY	6	6	100	1.13	0	0	0	0	0	0	0	0	0	1	1	1	1
AZ	28	27	96.43	1.09	0	0	0	1	3.57	0.77	0	0	0	1	1	1	1
B0	95	85	89.47	1.01	3	3.16	0.9	4	4.21	0.91	3	3.16	0.89	1	1	1	1
B1	211	193	91.47	1.04	14	6.64	1.9	4	1.9	0.41	0	0	0	1	1	1	1
B2	426	369	86.62	0.98	23	5.4	1.55	23	5.4	1.16	11	2.58	0.73	1	1	1	1
B3	22	19	86.36	0.98	0	0	0	1	4.55	0.98	2	9.09	2.57	1	1	1	1
B4	137	115	83.94	0.95	6	4.38	1.25	8	5.84	1.26	8	5.84	1.65	1	1	1	1
B5	10	9	90	1.02	0	0	0	1	10	2.15	0	0	0	1	1	1	1
B6	18	17	94.44	1.07	0	0	0	1	5.56	1.2	0	0	0	1	1	1	1
B7	11	11	100	1.13	0	0	0	0	0	0	0	0	0	1	1	1	1
B8	48	43	89.58	1.01	2	4.17	1.19	2	4.17	0.9	1	2.08	0.59	1	1	1	1
B9	169	161	95.27	1.08	2	1.18	0.34	6	3.55	0.76	0	0	0	0.120333538	1	1	1
BA	10	9	90	1.02	1	10	2.86	0	0	0	0	0	0	1	1	1	1
BB	13	11	84.62	0.96	0	0	0	2	15.38	3.31	0	0	0	1	1	1	1
BC	6	6	100	1.13	0	0	0	0	0	0	0	0	0	1	1	1	1
BD	19	16	84.21	0.95	2	10.53	3.01	0	0	0	1	5.26	1.49	1	1	1	1
BE	18	12	66.67	0.75	1	5.56	1.59	2	11.11	2.39	3	16.67	4.7	1	1	1	1
BF	8	5	62.5	0.71	0	0	0	2	25	5.38	1	12.5	3.53	1	1	1	1
BG	16	15	93.75	1.06	1	6.25	1.79	0	0	0	0	0	0	1	1	1	1
BH	14	13	92.86	1.05	1	7.14	2.05	0	0	0	0	0	0	1	1	1	1
BI	29	27	93.1	1.05	0	0	0	1	3.45	0.74	1	3.45	0.97	1	1	1	1
BK	8	7	87.5	0.99	1	12.5	3.58	0	0	0	0	0	0	1	1	1	1
BL	16	16	100	1.13	0	0	0	0	0	0	0	0	0	1	1	1	1
BM	3	3	100	1.13	0	0	0	0	0	0	0	0	0	1	1	1	1
BN	33	32	96.97	1.1	0	0	0	1	3.03	0.65	0	0	0	1	1	1	1
BO	50	48	96	1.09	0	0	0	2	4	0.86	0	0	0	1	1	1	1
BP	40	35	87.5	0.99	1	2.5	0.72	1	2.5	0.54	3	7.5	2.12	1	1	1	1
BQ	47	44	93.62	1.06	0	0	0	1	2.13	0.46	2	4.26	1.2	1	1	1	1
BR	41	39	95.12	1.08	2	4.88	1.4	0	0	0	0	0	0	1	1	1	1
BS	82	79	96.34	1.09	1	1.22	0.35	1	1.22	0.26	1	1.22	0.34	1	1	1	1
BT	43	42	97.67	1.11	1	2.33	0.67	0	0	0	0	0	0	1	1	1	1
BU	19	18	94.74	1.07	1	5.26	1.51	0	0	0	0	0	0	1	1	1	1

BY	170	159	93.53	1.06	2	1.18	0.34	9	5.29	1.14	0	0	0	1	1	1	1
BZ	207	200	96.62	1.09	3	1.45	0.41	4	1.93	0.42	0	0	0	0.000928342	1	1	1
C0	65	57	87.69	0.99	4	6.15	1.76	1	1.54	0.33	3	4.62	1.3	1	1	1	1
C1	376	323	85.9	0.97	23	6.12	1.75	12	3.19	0.69	18	4.79	1.35	1	1	1	1
C2	239	221	92.47	1.05	8	3.35	0.96	3	1.26	0.27	7	2.93	0.83	1	1	1	1
C3	204	191	93.63	1.06	7	3.43	0.98	3	1.47	0.32	3	1.47	0.42	0.673618946	1	1	1
C4	158	151	95.57	1.08	1	0.63	0.18	3	1.9	0.41	3	1.9	0.54	0.108522028	1	1	1
C5	166	153	92.17	1.04	4	2.41	0.69	6	3.61	0.78	3	1.81	0.51	1	1	1	1
C6	182	161	88.46	1	6	3.3	0.94	6	3.3	0.71	9	4.95	1.4	1	1	1	1
C7	291	197	67.7	0.77	14	4.81	1.38	30	10.31	2.22	50	17.18	4.85	1	1	0.031259706	2.51E-16
C8	84	76	90.48	1.02	4	4.76	1.36	1	1.19	0.26	3	3.57	1.01	1	1	1	1
C9	102	84	82.35	0.93	4	3.92	1.12	6	5.88	1.27	8	7.84	2.21	1	1	1	1
CA	84	79	94.05	1.06	2	2.38	0.68	2	2.38	0.51	1	1.19	0.34	1	1	1	1
CB	80	78	97.5	1.1	0	0	0	0	0	0	2	2.5	0.71	0.38693388	1	1	1
CC	137	134	97.81	1.11	1	0.73	0.21	2	1.46	0.31	0	0	0	0.003428601	1	1	1
CD	67	62	92.54	1.05	1	1.49	0.43	3	4.48	0.96	1	1.49	0.42	1	1	1	1
CE	132	126	95.45	1.08	2	1.52	0.43	2	1.52	0.33	2	1.52	0.43	0.383764482	1	1	1
CF	167	152	91.02	1.03	4	2.4	0.69	4	2.4	0.52	7	4.19	1.18	1	1	1	1
CG	70	65	92.86	1.05	1	1.43	0.41	4	5.71	1.23	0	0	0	1	1	1	1
CI	1	1	100	1.13	0	0	0	0	0	0	0	0	0	1	1	1	1
CR	2	2	100	1.13	0	0	0	0	0	0	0	0	0	1	1	1	1
CS	1	1	100	1.13	0	0	0	0	0	0	0	0	0	1	1	1	1
CT	3	3	100	1.13	0	0	0	0	0	0	0	0	0	1	1	1	1
CV	1	1	100	1.13	0	0	0	0	0	0	0	0	0	1	1	1	1
D0	223	193	86.55	0.98	13	5.83	1.67	8	3.59	0.77	9	4.04	1.14	1	1	1	1
D1	328	288	87.8	0.99	15	4.57	1.31	17	5.18	1.12	8	2.44	0.69	1	1	1	1
D2	149	128	85.91	0.97	12	8.05	2.31	6	4.03	0.87	3	2.01	0.57	1	1	1	1
D3	222	198	89.19	1.01	12	5.41	1.55	4	1.8	0.39	8	3.6	1.02	1	1	1	1
D4	138	122	88.41	1	2	1.45	0.41	9	6.52	1.4	5	3.62	1.02	1	1	1	1
D5	52	43	82.69	0.94	6	11.54	3.3	1	1.92	0.41	2	3.85	1.09	1	1	1	1
D6	134	113	84.33	0.95	3	2.24	0.64	10	7.46	1.61	8	5.97	1.68	1	1	1	1
D7	79	65	82.28	0.93	3	3.8	1.09	2	2.53	0.54	9	11.39	3.22	1	1	1	0.82744797
D8	78	58	74.36	0.84	7	8.97	2.57	5	6.41	1.38	8	10.26	2.89	1	1	1	1
D9	147	130	88.44	1	7	4.76	1.36	5	3.4	0.73	5	3.4	0.96	1	1	1	1
DC	4	4	100	1.13	0	0	0	0	0	0	0	0	0	1	1	1	1
DE	1	0	0	0	0	0	0	1	100	21.53	0	0	0	1	1	1	1
DG	56	53	94.64	1.07	2	3.57	1.02	1	1.79	0.38	0	0	0	1	1	1	1
DP	2	2	100	1.13	0	0	0	0	0	0	0	0	0	1	1	1	1
E0	184	153	83.15	0.94	8	4.35	1.24	7	3.8	0.82	16	8.7	2.45	1	1	1	0.551527511
E1	333	308	92.49	1.05	4	1.2	0.34	9	2.7	0.58	12	3.6	1.02	0.479831088	1	1	1
E2	100	83	83	0.94	4	4	1.15	6	6	1.29	7	7	1.98	1	1	1	1

E3	153	132	86.27	0.98	6	3.92	1.12	7	4.58	0.98	8	5.23	1.48	1	1	1	1
E4	694	630	90.78	1.03	23	3.31	0.95	25	3.6	0.78	16	2.31	0.65	0.792092257	1	1	1
E5	20	18	90	1.02	0	0	0	1	5	1.08	1	5	1.41	1	1	1	1
E6	3	3	100	1.13	0	0	0	0	0	0	0	0	0	1	1	1	1
E8	14	14	100	1.13	0	0	0	0	0	0	0	0	0	1	1	1	1
E9	36	33	91.67	1.04	0	0	0	3	8.33	1.79	0	0	0	1	1	1	1
F4	191	161	84.29	0.95	12	6.28	1.8	8	4.19	0.9	10	5.24	1.48	1	1	1	1
F5	89	74	83.15	0.94	2	2.25	0.64	5	5.62	1.21	8	8.99	2.54	1	1	1	1
F6	1390	1227	88.27	1	30	2.16	0.62	83	5.97	1.29	50	3.6	1.02	1	1	1	1
F7	289	244	84.43	0.96	10	3.46	0.99	19	6.57	1.42	16	5.54	1.56	1	1	1	1
F8	803	683	85.06	0.96	25	3.11	0.89	60	7.47	1.61	35	4.36	1.23	1	1	0.266688663	1
F9	225	199	88.44	1	8	3.56	1.02	13	5.78	1.24	5	2.22	0.63	1	1	1	1
G1	4	4	100	1.13	0	0	0	0	0	0	0	0	0	1	1	1	1
G2	163	153	93.87	1.06	7	4.29	1.23	1	0.61	0.13	2	1.23	0.35	1	1	1	1
G3	152	138	90.79	1.03	5	3.29	0.94	6	3.95	0.85	3	1.97	0.56	1	1	1	1
G4	11	11	100	1.13	0	0	0	0	0	0	0	0	0	1	1	1	1
G5	260	238	91.54	1.04	5	1.92	0.55	11	4.23	0.91	6	2.31	0.65	1	1	1	1
G6	11	10	90.91	1.03	0	0	0	0	0	0	1	9.09	2.57	1	1	1	1
G7	292	274	93.84	1.06	7	2.4	0.69	4	1.37	0.29	7	2.4	0.68	0.056378085	1	1	1
G8	173	156	90.17	1.02	6	3.47	0.99	8	4.62	1	3	1.73	0.49	1	1	1	1
G9	124	115	92.74	1.05	1	0.81	0.23	7	5.65	1.22	1	0.81	0.23	1	1	1	1
I0	7	6	85.71	0.97	0	0	0	1	14.29	3.08	0	0	0	1	1	1	1
I1	317	303	95.58	1.08	4	1.26	0.36	7	2.21	0.48	3	0.95	0.27	0.000149846	1	1	1
I4	441	409	92.74	1.05	8	1.81	0.52	18	4.08	0.88	6	1.36	0.38	0.04985844	1	1	1
I5	11	11	100	1.13	0	0	0	0	0	0	0	0	0	1	1	1	1
I6	1	1	100	1.13	0	0	0	0	0	0	0	0	0	1	1	1	1
I7	16	15	93.75	1.06	0	0	0	1	6.25	1.35	0	0	0	1	1	1	1
I8	1023	936	91.5	1.04	14	1.37	0.39	46	4.5	0.97	27	2.64	0.74	0.004847755	1	1	1
I9	50	47	94	1.06	0	0	0	2	4	0.86	1	2	0.56	1	1	1	1
K0	10	9	90	1.02	0	0	0	1	10	2.15	0	0	0	1	1	1	1
K1	2	1	50	0.57	0	0	0	1	50	10.76	0	0	0	1	1	1	1
K2	56	51	91.07	1.03	2	3.57	1.02	2	3.57	0.77	1	1.79	0.5	1	1	1	1
K3	1	1	100	1.13	0	0	0	0	0	0	0	0	0	1	1	1	1
K4	32	27	84.38	0.96	0	0	0	2	6.25	1.35	3	9.38	2.65	1	1	1	1
K5	57	55	96.49	1.09	0	0	0	1	1.75	0.38	1	1.75	0.5	1	1	1	1
K8	1	1	100	1.13	0	0	0	0	0	0	0	0	0	1	1	1	1
K9	2	2	100	1.13	0	0	0	0	0	0	0	0	0	1	1	1	1
L2	1	1	100	1.13	0	0	0	0	0	0	0	0	0	1	1	1	1
L9	1	1	100	1.13	0	0	0	0	0	0	0	0	0	1	1	1	1
M4	5	5	100	1.13	0	0	0	0	0	0	0	0	0	1	1	1	1
M5	113	102	90.27	1.02	5	4.42	1.27	4	3.54	0.76	2	1.77	0.5	1	1	1	1
